# A Computational Approach to Identifying Gene-microRNA Modules in Cancer

**DOI:** 10.1371/journal.pcbi.1004042

**Published:** 2015-01-22

**Authors:** Daeyong Jin, Hyunju Lee

**Affiliations:** 1 School of Information and Communications, Gwangju Institute of Science and Technology, Gwangju, South Korea; Thomas Jefferson University, UNITED STATES

## Abstract

MicroRNAs (miRNAs) play key roles in the initiation and progression of various cancers by regulating genes. Regulatory interactions between genes and miRNAs are complex, as multiple miRNAs can regulate multiple genes. In addtion, these interactions vary from patient to patient and even among patients with the same cancer type, as cancer development is a heterogeneous process. These relationships are more complicated because transcription factors and other regulatory molecules can also regulate miRNAs and genes. Hence, it is important to identify the complex relationships between genes and miRNAs in cancer.

In this study, we propose a computational approach to constructing modules that represent these relationships by integrating the expression data of genes and miRNAs with gene-gene interaction data. First, we used a biclustering algorithm to construct modules consisting of a subset of genes and a subset of samples to incorporate the heterogeneity of cancer cells. Second, we combined gene-gene interactions to include genes that play important roles in cancer-related pathways. Then, we selected miRNAs that are closely associated with genes in the modules based on a Gaussian Bayesian network and Bayesian Information Criteria. When we applied our approach to ovarian cancer and glioblastoma (GBM) data sets, 33 and 54 modules were constructed, respectively. In these modules, 91% and 94% of ovarian cancer and GBM modules, respectively, were explained either by direct regulation between genes and miRNAs or by indirect relationships via transcription factors. In addition, 48.4% and 74.0% of modules from ovarian cancer and GBM, respectively, were enriched with cancer-related pathways, and 51.7% and 71.7% of miRNAs in modules were ovarian cancer-related miRNAs and GBM-related miRNAs, respectively. Finally, we extensively analyzed significant modules and showed that most genes in these modules were related to ovarian cancer and GBM.

## Author Summary

A microRNA (miRNA) is a small RNA molecule that regulates the expression of mRNA genes. A miRNA can regulate multiple genes, and a gene can be regulated by multiple miRNAs. The regulation of genes by miRNAs may vary from patient to patient, even if they suffer from the same type of cancer. In this study, we identify the relationships between genes and miRNAs in cancer patients using expression data. Because these relationships are complicated by the involvement of transcription factors, which are among the most influential regulators of genes, we also attempt to explain the triple relationship among genes, miRNAs, and transcription factors. We constructed modules consisting of a set of genes and miRNAs, in which the expression levels are highly correlated. In most of these modules, genes and miRNAs are related to specific cancer types; their relationships are explained both by direct regulation of genes by miRNAs and by indirect relationships via transcription factors.

## Introduction

Cancer is one of the leading causes of death worldwide. Although remarkable progress has been achieved in cancer therapies, the molecular mechanisms of cancer have not yet been fully identified. Among various regulations of cancer-related genes and pathways in several stages, the regulation of genes by microRNAs (miRNAs) in cancer cells has drawn particular attention, because many miRNAs are located in chromosomal regions that are frequently altered in cancer [1]. MiRNAs are small RNAs, known as important regulators of genes through binding to 3’ UTR regions of target genes [2]. In many cancer types, miRNAs have been studied as important biomarkers for diagnosis and prognosis of cancer, as many miRNAs function as oncogenes or tumor suppressors by regulating other oncogenes or tumor suppressor genes [1, 3].

Because miRNAs regulate genes by binding to the 3’ UTR regions of genes, many methods were developed to identify conserved sequence regions between miRNAs and mRNAs [4]. However, sequence-based approaches generate many false positive bindings sites and cannot identify functional changes of genes. Hence, the expressions of genes and miRNAs were also integrated to address possible negative correlations between the two sets of expression data [5, 6]. With the advances in high throughput technologies, large-scale mRNA expression and miRNA expression data sets from the same tumor samples have become available, due to collaborative efforts such as The Cancer Genome Atlas (TCGA) project. [7, 8]. These data sets enable researchers to apply computational approaches to identify relationships between mRNAs and miRNAs and help understand their effects in cancer.

Another important approach to understanding relationships between mRNAs and miRNAs is to analyze multiple genes and miRNAs simultaneously by constructing modules of them rather than analyzing each gene-miRNA pair separately [5, 9, 10]. It is widely known that a miRNA can regulate multiple genes [11], and a gene can be targeted by multiple miRNAs [12]. Changes in these numerous relationships can significantly alter the biological functions or signaling pathways associated with a specific cancer [13]. Although it is known that several pathways, such as the p53 and TGF-beta signaling pathways, are related to ovarian cancer [14, 15], the functions of miRNAs in these pathways have not yet been fully explained.

Although a few algorithms for finding gene-miRNA modules have been proposed, improvements are still needed. Peng et al. [5] proposed a bi-clique approach based on a gene-miRNA correlation matrix; however, most of the modules contained only one miRNA, and a few modules contained at most three miRNAs. Hence, it may be difficult to address multiple relationships between genes and miRNAs. Zhang et al. [6] integrated miRNAs, gene expression and gene-gene interactions based on a non-negative matrix factorization (NMF) framework [16]. The decomposed matrix components were considered as gene-miRNA regulatory modules. Although many modules were enriched with known pathways, the relationships between genes and miRNAs were not explained.

Relationships between genes and miRNAs become even more complicated because molecules such as transcription factors or signal transducers regulate genes and miRNAs. For example, p53, the most frequently mutated gene in cancer, regulates hundreds of genes and a set of miRNAs, including miR-24 family, miR-145, miR-107, and miR-192 [17, 18]. In [19], the authors constructed modules that contain highly correlated genes and miRNAs in their expression levels and found that miR-200a regulates the transcription factor ZEB1, which regulates genes contained in the same module as miR-200a.

To enhance the understanding of relationships between genes and miRNAs, we propose a framework that combines a biclustering approach and a Gaussian Bayesian network. Using the biclustering approach, gene-sample modules are first constructed based on gene expression and gene-gene interaction data sets. Here, a subset of genes that are correlated with each other in a subset of samples is clustered, because gene aberrations are different among patients, even if cancer occurs in the same organ or tissue type [20]. Next, using a Gaussian Bayesian network, gene-miRNA modules are constructed to identify miRNAs that regulate genes in gene-sample modules. Here, we use the expression data on genes and miRNA. When we applied our approach to ovarian cancer data sets and glioblastoma (GBM) data sets from TCGA, we identified several modules consisting of genes and miRNAs related to ovarian cancer and GBM. In many modules, relationships between genes and miRNAs were explained either by direct regulations of genes by miRNAs or by indirect relationships via transcription factors. In addition, functional pathway enrichment tests using several biological and signaling pathways demonstrated that these modules were biologically coherent. Based on ratios of cancer-related genes and cancer-related miRNAs, we extensively analyzed several significant modules and performed network analyses of these modules to demonstrate the regulation of genes by miRNAs.

## Materials and Methods

### Materials

**Figure 1:**
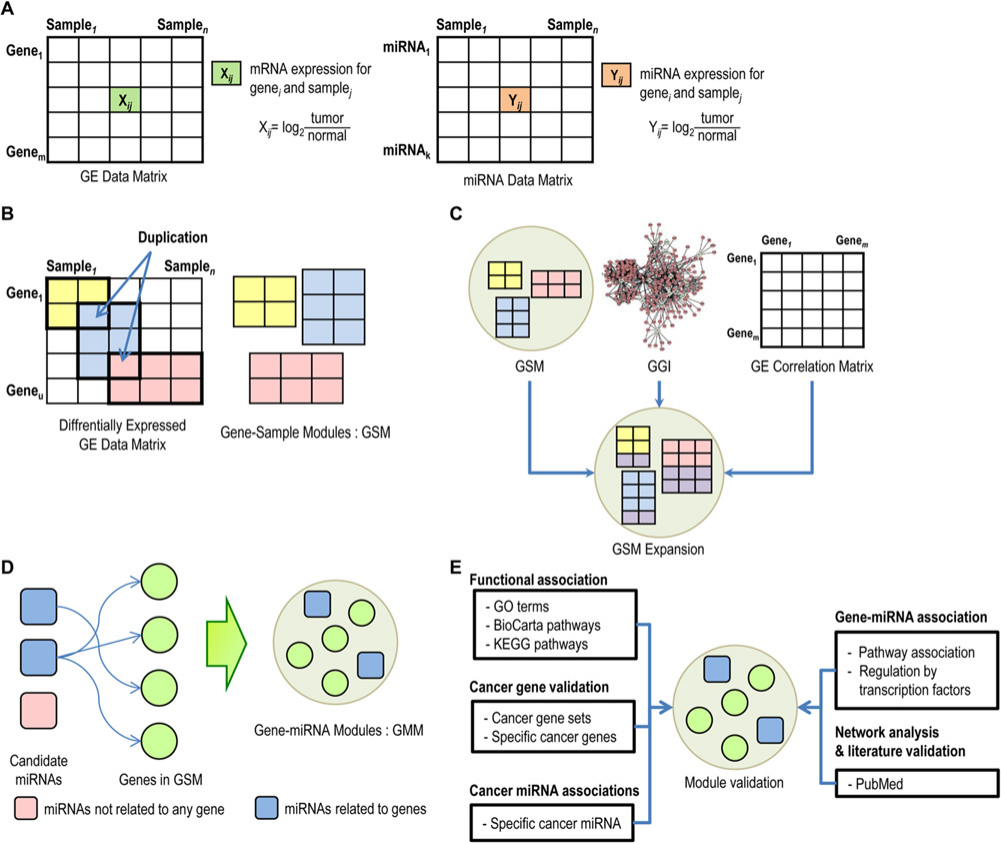
Overview of the proposed approach. (A) Collect gene expression and miRNA expression data sets from paired tumor samples, and calculate log2 ratios between tumor samples and normal samples. (B) Construct gene-sample modules (GSM) from a differentially expressed gene expression matrix using a biclustering algorithm, which allows duplications of genes and samples in multiple modules. (C) Add genes to GSM using gene-gene interactions, if the included genes increase the average PCC values among genes in the module. (D) Construct gene-miRNA modules (GMM) by selecting gene-regulating miRNAs in GSM. Use a Gaussian Bayesian network and the BIC score to evaluate the relationship between genes and miRNAs. (E) To determine the functional relevance of the modules, test whether the genes from the modules are enriched for specific biological functions or signaling pathways. To validate that modules are related to a specific cancer, check that the genes and miRNAs are related to the specific cancer.

**Ovarian cancer.** We collected mRNA expression and miRNA expression data sets for 587 tumor samples and 8 unmatched normal samples for ovarian cancer from TCGA [8]; mRNA and miRNA expression data were generated using an Affymetrix HG-U133A microarray and an Agilent H-miRNA_8X15K microarray, respectively. We normalized the expression levels of 12,042 genes using log2 ratios between tumor samples and the average of normal samples for each gene, and then selected 2,933 differentially expressed genes using a *t*-test (*p*-value < 0.001). Similarly, we normalized the expression levels of 479 miRNAs using the log2 ratios between tumor samples and the average of normal samples for each miRNA (Fig. 1 (A)).

**Glioblastoma.** We collected mRNA expression and miRNA expression data sets for 482 tumor samples and 10 unmatched normal samples for GBM [7]. These data sets were generated using the same microarray platforms used in the ovarian cancer study. After normalization, we selected 4,059 differentially expressed genes using a *t*-test (Bonferroni corrected *p*-value < 0.05). We used the expression levels of 423 miRNAs normalized using normal samples.

**Selecting a p-value threshold for a t-test.** The degree of expression changes depending on the cancer type. In this study, the number of differentially expressed genes was small in ovarian cancer compared to GBM. Hence, we used a less strict threshold for ovarian cancer.

**Gene-gene interactions.** We collected gene-gene interaction data from the HPRD database [21].

### Constructing gene-sample modules

In this study, we first hypothesized that if a group of genes has similar expression tendencies in a subset of samples, and they are differentially expressed in these samples, then these genes might be related to similar functions or pathways in the development of cancer. We also hypothesized that a gene might have multiple functions and could function in several pathways. To incorporate these hypotheses, we use a biclustering algorithm to allow the duplication of genes and samples in multiple clusters. First, we construct a matrix of differentially expressed genes and samples, and then we normalize the expression values for each gene using a z-score to determine the tendency toward changes of gene expression in the samples. Next, we apply a SAMBA biclustering algorithm [22] to the normalized matrix to construct modules in which genes and samples are highly correlated (Fig. 1 (B)). The SAMBA biclustering algorithm models gene expression data in a bipartite graph *G* = (*U*,*V*,*E*), where genes in *V* are represented as nodes on one side and samples in *U* on the other side. There is an edge in *E* between a gene *v* in *V* and a sample *u* in *U* if the expression value of gene *v* changes significantly in sample *u*, having high absolute expression values. The biclustering algorithm generates subgraphs from the bipartite graph, in which most of the genes are connected to most of the samples as edges. These subgraphs represent highly correlated gene-sample clusters, where the tendency toward gene expression changes is similar for a subset of samples. Additional details are provided in Fig. S1. We calculate the statistical significance of each module based on a null hypothesis that the expression level of a gene is independent of the expression level of other genes for samples in a module, assessing that the average Pearson correlation coefficients (PCCs) of gene expression levels for genes in the module are higher than the ones from random modules for selected samples. For each module, we conduct the following test.

**(Step 1)** Construct a random module by randomly selecting the same numbers of genes and samples from the normalized matrix.
**(Step 2)** Calculate the PCC matrix of expression level values of genes in the module across a subset of samples. Then, calculate the average value of the PCC matrix, excluding diagonal elements.
**(Step 3)** Repeat Steps 1 and 2 *N* times, letting the average value from the *i*-th permutation serve as the random_*avg*_(*i*).
**(Step 4)** Let the average PCC value of genes in the observed module be the module_*avg*_.
**(Step 5)** Calculate the *p*-value of the observed module using the following equation, where I is an indicator function.

$$p-value=\frac{\sum_{i=1}^{N}I(module_{avg}<random_{avg}(i))}{N}$$

When we calculate the *p*-value, we try to take into account that observed modules are not independent of each other as genes overlap among modules. Hence, we construct random modules where genes in the modules share the same overlap ratio as the observed modules.

Recent research has shown that not all of the genes in cancer-related pathways undergo expression or genomic changes [23]. Consequently, certain genes that play important roles in cancer-related pathways might not be differentially expressed. To include functionally related genes in the gene-sample modules, we expand the gene-sample modules using a gene-gene interaction network. If a gene interacts directly with at least one gene in a module, then this gene can be regarded as a candidate gene for the module. For each module, we collect candidate genes and calculate the average PCC values of expressions between a candidate gene and the genes in the module. We add candidate genes to the module in descending order from the gene having the highest PCC value until the average PCC values of the expressions of genes in the module do not increase.

### Constructing gene-miRNA modules

Because a set of genes with similar expression changes might be regulated by common miRNAs, we construct gene-miRNA modules by including regulating miRNAs in the gene-sample modules. For this task, we employ a Bayesian network model. Bayesian networks have been extensively used for analyzing gene expression patterns [24]. They are useful in modeling local dependencies and causal influences among variables. Hence, we estimate dependencies between expression values of genes and expression values of miRNAs based on a Bayesian network model. A joint distribution of genes *X* = {*X*_1_,*X*_2_, …, *X*_*n*_} and miRNAs *Y* = {*Y*_1_,*Y*_2_, … *Y*_*m*_} is represented by a Gaussian Bayesian network. If *X*_*i*_ is normally distributed around a mean that linearly depends on its parents, then the conditional probability of *X*_*i*_ given its parents *Pa* ^*G*^(*X*_*i*_) = {*Y*_*j*_, … *Y*_*k*_} can be represented by

$$P\left(X_{i}|Pa^{G}\left(X_{i}\right)\right)=P\left(X_{i}|Y_{j},\ldots ,Y_{k}\right)∼N\left(a_{0}+\sum_{j^{'}}a_{j^{'}}\cdot Y_{j^{'}},\sigma^{2}\right)$$ (1)

Then, the likelihood of *X* and *Y* can be represented by

$$L\left(X,Y\right)=P\left(X_{1},X_{2},\ldots ,X_{n},Y_{1},Y_{2},\ldots Y_{m}\right)=\prod_{i=1}^{n}P\left(X_{i}|Pa^{G}\left(X_{i}\right)\right)$$ (2)

To determine which sets of miRNAs explain the expression levels of genes in gene-sample modules, we use a Bayesian information criterion (BIC) as a measure for determining a Bayesian network structure between genes and miRNAs, which can be represented by

$$BIC=log\left(L\right)-\frac{logM}{2}+O\left(1\right),$$ (3)

where *M* is the sum of the number of genes and miRNAs. To determine the parents *Pa* ^*G*^(*X*_*i*_) of a gene *X*_*i*_ yielding the optimal BIC score, we should consider all combinations of miRNAs; however, this approach is highly time-consuming. To reduce the search space, we select candidate miRNAs whose average of absolute Spearman’s rank correlation coefficient (SCC) values for genes in a given module are within the top *T*% among all miRNAs. Note that we use SCC values for selecting candidate miRNAs to reduce the effects of possible outliers in the PCC. From candidate miRNAs, we first add a miRNA with the highest SCC value as a regulator and calculate the BIC score. Then, we add miRNAs with the next highest SCC values, until adding more miRNAs no longer improves the BIC score. After adding miRNAs to gene-sample modules using the above approach, modules with fewer than two miRNAs are filtered out because these modules cannot represent the combinatorial effects of genes and miRNAs. Finally, gene-miRNA modules are obtained.

### Module validation

To validate the relationships between genes and miRNAs in the modules, we consider four cases of gene regulations. In the first case, genes are directly bound and regulated by miRNAs. To validate this case, we select gene-miRNA pairs from miRTarbase [25] and MicroCosm (http://www.ebi.ac.uk/enright-srv/microcosm/htdocs/targets/v5/). Interacting pairs in miRTarbase are validated by various molecular experiments. Among them, reporter assays and western blot analysis confirm direct interactions. We compare the gene-miRNA pairs in our modules with these direct interactions in miRTarbase. MicroCosm provides computationally predicted binding sites for miRNAs in genomic sequences. Among these pairs, we select only gene-miRNA pairs with a negative correlation in expression values. From this process, we collect target genes for each miRNA, which we use for validation. Then, we perform a hypergeometric test for each miRNA in the modules to check for enrichment of genes in a module against the target genes of a miRNA.

However, certain genes in the modules are not directly regulated by miRNAs, even though the expressions of the genes and the miRNAs are highly correlated. To investigate this indirect relationship, we introduce transcription factors (TFs). We confirm relationships between miRNAs and TFs by manually searching the literature for evidence of cases where miRNAs are regulated by TFs or TFs are regulated by miRNAs. In the second case, we consider a relationship in which the miRNAs in a module regulate TFs, and these TFs regulate genes in the module. Here, it is not necessary that TFs be members of the module. We identify relationships between TFs and genes using the ChIP-X database [26]. For each TF in the database, we perform a hypergeometric test to determine if there is enrichment of genes in a module against the target genes of the TF. Here, the correlation of expression values between the miRNA and the TF must be negative, and the correlation values between the TF and the mRNA can be either positive or negative.

In the third case, genes and miRNAs are regulated by a common TF. In this case, correlations of expression values between gene-TF and miRNA-TF should be both positive or both negative.

In the fourth case, interacting pairs in miRTarbase [25], experimentally validated by the coexpression of miRNA and mRNA, are used to validate gene-miRNA pairs in our module. Molecular experiments for this case include quantitative real-time PCR (qPCR), microarrays, stable isotope labeling with amino acids in culture (SILAC) and pulsed SILAC.

To determine the functional relevance of the modules, we test whether the genes from the modules are enriched for specific biological functions or signaling pathways. We perform a pathway enrichment test using gene ontology (GO) biological process terms [27], KEGG pathways [28], and BioCarta pathways (http://www.biocarta.com). First, we download these pathways from GSEA (http://www.broadinstitute.org/gsea) and apply a hypergeometric test to each module, obtaining the *p*-values. We exclude biological functions or signaling pathways containing more than 300 genes, as such functions are too general. Supplementary Fig. S2 shows the distribution of GO biological functions as well as KEGG and BioCarta pathways. It can be seen that 51 of 825 GO terms contain more than 300 genes. To address any issues with multiple comparisons, we compute the *q*-values from the *p*-values based on a Benjamini & Hochberg correction. Then, we use a *q*-value < 0.05 for the enrichment threshold.

To validate that modules are related to the specific cancer, we first examine whether enriched pathways are related to the cancer being evaluated. For this task, we collect 2,032 cancer genes from the allOnco database (http://www.bushmanlab.org/links/genelists), which is a collection of list of cancer genes from several databases [29–32], 379 ovarian cancer genes from the Dragon Database for Exploration of Ovarian Cancer Genes (DDOC [33]), and 98 GBM genes from the literature ([34, 35]). Then, we calculate the ratios of these cancer genes in the modules. We also collect 100 ovarian cancer miRNAs and 92 GBM miRNAs from the Human miRNA & Disease Database (HMDD [36]). Then, we calculate the ratios of ovarian cancer-related miRNAs in the modules.

### Associating modules with cancer subtype

Genes involved in the development of cancer vary depending on cancer subtypes. In several papers [8, 37–39], the expression levels of marker genes are used to determine the subtype. For example, GBM samples were classified as a proneural subtype if marker genes DLL3, NKX2–2, SOX2, ERBB3, and OLIG2 were overexpressed [8]. Similarly, we check whether modules identified by our approach are related to a specific subtype of cancers using marker genes.

For this task, we perform the following two steps. In the first step, we cluster all samples into subtypes using hierarchical clustering with a dynamic tree cut [40]. For clustering, we use genes with high variability across the samples. Then, we assign each cluster to a subtype of cancers if known marker genes of cancer subtypes are overexpressed or underexpressed. If a cluster is not related to any subtype or is related to more than one subtype, that cluster is not assigned to any subtype. In the second step, for each module, we use marker genes of the subtype to compare the expression levels of the marker genes of samples in a module to the expression levels of samples in the other subtype clusters using the *t*-test. If the *p*-values of markers genes of the subtype are significant, we consider the module to be related to the given subtype.

## Results

### Gene-Sample modules for ovarian cancer and GBM

To construct gene-sample modules, we applied the SAMBA biclustering algorithm to the gene expression matrix, allowing duplication of genes and samples in modules using an overlap factor of 0.5 in [0, 1], where 1 represents non-overlap. For ovarian cancer and GBM, we identified 90 and 135 modules, respectively, that represent similar tendencies of gene expression changes for a subset of samples. After performing 1000 permutation tests, we selected 58 and 88 modules with a *q*-value < 0.05 for ovarian cancer and GBM, respectively. Then, we enlarged these modules by adding genes using gene-gene interactions. On average, we added 15 and 33 genes to each module for ovarian cancer and GBM, respectively.

### Gene-miRNA modules for ovarian cancer and GBM

**Figure 2:**
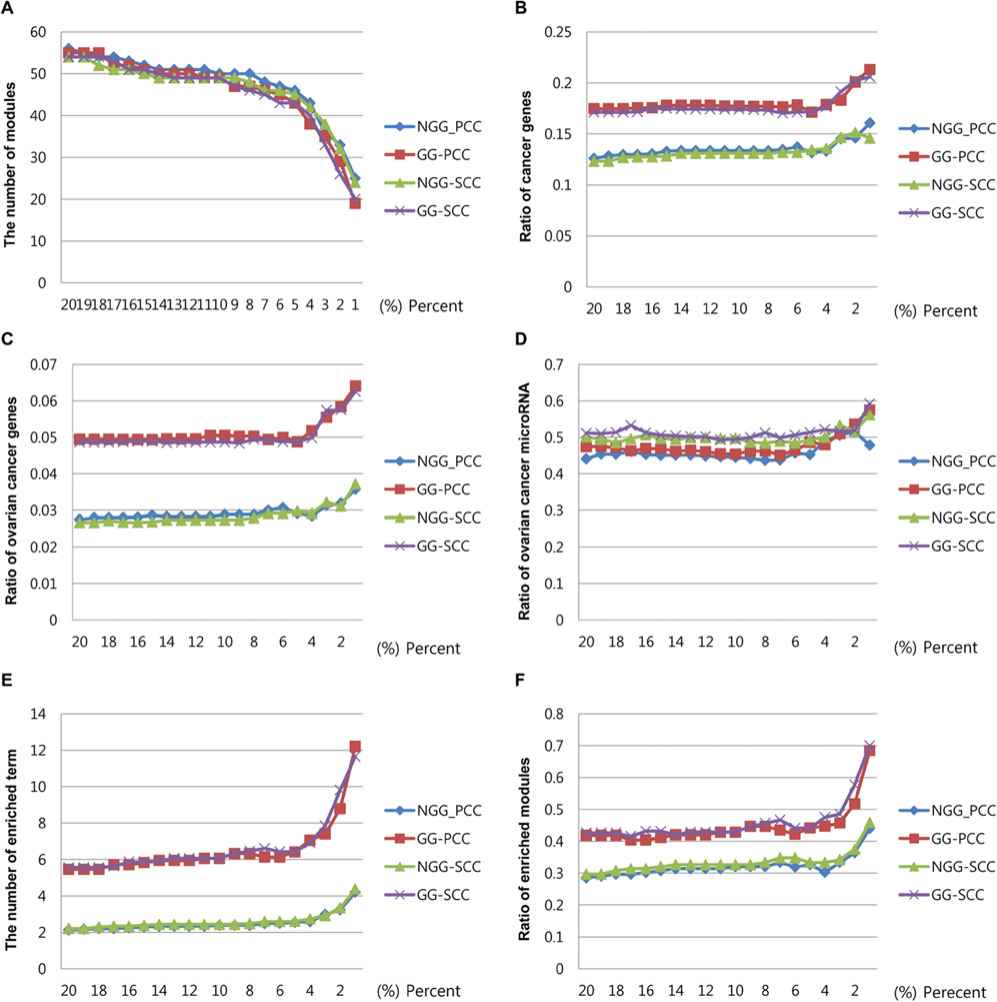
Performance comparison of gene-miRNA modules for ovarian cancer. For ovarian cancer, we compared the performance of gene-miRNA modules generated from four cases: SCC with GGI information, SCC without GGI information, PCC with GGI information, and PCC without GGI information. For all cases, the *x*-axis presents different percentages of candidate miRNAs (*T*%) among all miRNAs when constructing gene-miRNA modules. For each case, the number of modules (A), the ratios of cancer genes (B), the ratios of ovarian cancer genes (C), the ratios of ovarian cancer miRNAs (D), the average number of enriched pathways (E), and the ratios of modules enriched with at least one pathway (F) are shown.

We constructed gene-miRNA modules from gene-sample modules by including miRNAs. As described in the Methods section, we pre-selected the candidate miRNAs based on the SCC values between the genes and the miRNAs and then added miRNAs to the module, which increased the BIC score. As shown in Fig. 2, we applied 20 different SCC thresholds (*T*% in [1%, 20%] of candidate miRNAs among all miRNAs) to reduce the search space. In Fig. 2 (A), the number of modules for ovarian cancer decreased as the thresholds decreased. We observed similar trends when the PCC was used instead of the SCC or when we did not integrate the gene-gene interaction data. Fig. 2 also shows that the ratios of cancer genes, ovarian cancer genes, and ovarian cancer miRNAs were similar for various SCC thresholds > 5%, and that these ratios increased when SCC thresholds decreased. Fig. S3 shows similar results for GBM. Note that we filtered out modules with fewer than two miRNAs, as such modules cannot represent the combinatorial effects of genes and miRNAs.

Among the various thresholds for candidate miRNAs, we selected a value of 3% (SCC value = 0.157 for ovarian cancer and 0.194 for GBM) for further analysis and constructed 33 and 54 modules for ovarian cancer and GBM, respectively. Tables S1, S2, S3, and S4 present lists of genes and miRNAs for the modules. For ovarian cancer, the average size of the modules was 34 genes and 10 miRNAs. On average, 19.1% of genes were cancer genes, 5.7% were ovarian cancer genes, and 51.7% of miRNAs were ovarian cancer-related miRNAs in the ovarian cancer modules. When combining genes and miRNAs from all modules, 18.6% (145 out of 777) of genes were cancer genes, 6.0% (47 out of 777 genes) were ovarian cancer genes, and 43.5% (47 out of 108) of miRNAs were ovarian cancer-related miRNAs. Based on the pathway enrichment test, 48.4% of the modules were enriched with biological functions or signaling pathways, and most of the modules contained at least one ovarian cancer gene. Table 1 shows ovarian cancer genes and miRNAs for the selected modules. Table S5 presents lists of cancer genes, ovarian cancer genes, and ovarian cancer miRNAs for all of the ovarian cancer modules. For GBM, the average numbers of genes and miRNAs for each module were 66 genes and 14 miRNAs. In the GBM modules, on average, 23.2% of the genes were cancer genes, 1.2% were GBM-related genes, and 71.7% of the miRNAs were GBM-related miRNAs. For all genes and miRNAs in the GBM modules, 20.6% (386 out of 1867) of the genes were cancer genes, 1.7% (32 out of 1867 genes) were GBM-related genes, and 48.4% (46 out of 95) of the miRNAs were GBM-related miRNAs. Table S6 presents lists of cancer genes, GBM genes, and GBM miRNAs for all of the GBM modules. Based on the pathway enrichment test, 74.0% of the modules were enriched in biological functions or signaling pathways.

**Table 1:** Cancer genes, ovarian cancer genes and ovarian cancer miRNAs for selected modules.

Module ID	Cancer Genes	Num1^*a*^	Ovarian Cancer Genes	Num2^*b*^	Ovarian Cancer miRNAs	Num3^*c*^
2	CD44, MMP9, PLAUR, LTB, GBP1, CTSH, EPB41L3, POU2AF1, VAV1, CXCL10, MEF2C, HCK, BTK, CASP1, CD74, LCK, LYN, FGR, SPP1	19/60	CD44, DPYD, IL18, MMP9, PLAUR	5/60	miR-125b, miR-146a, miR-155, miR-17, miR-20a, miR-21, miR-218, miR-22, miR-223, miR-224, miR-335	11/24
3	CDK2, E2F1, PLK1, MCM2, CDC6, EZH2, ASPM, BUB1	8/35	CDK2, E2F1	2/35	miR-106b, miR-130b, miR-18a, miR-19a, miR-25, miR-29a, miR-93	7/14
6	BARD1, CDC25A, CDK2, MSH6, MCM2, BUB1, FEN1, PCNA, CDKN3	9/34	BARD1, CDC25A, CDK2, MKI67, MSH6	5/34	miR-101, miR-106b, miR-130b, miR-17, miR-18a, miR-19a, miR-20b, miR-25, miR-29a, miR-93	10/20
8	PLAUR, MMP11, BGN, COL16A1, THBS2, THBS1, VCAN, COL1A1, TIMP3, PDGFRB, COL1A2	11/39	FN1, LGALS1, PLAU, PLAUR, SERPINE1	5/39	miR-152, miR-199a, miR-214, miR-22	4/8
12	E2F3, MCM2, FEN1, DEK, PALB2, PSMA5	6/33	E2F3, NBN	2/33	miR-93	1/2
13	CDC42, PLK1, CDC6, BUB1, PCNA, UCHL5, FANCE, SMARCB1, FANCG, EIF4EBP1, ECT2	11/78	CDC42	1/78	miR-18a, miR-25, miR-29a, miR-93	4/8
18	MCM2, FEN1, FOXM1, DEK, FANCG, WHSC1	6/31	MKI67	1/31	miR-18a, miR-25, miR-29a, miR-93	4/7
20	AURKA, CDC20, MAD2L1, TOP2A, PLK1, ASPM, BUB1, FOXM1, MYBL2, KIF14, CCNA2, CCNB1, BUB1B	13/44	AURKA, CDC20, MAD2L1, TOP2A	4/44	miR-101, miR-17, miR-18a, miR-19a, miR-29a, miR-93	6/13
21	HCK, BTK, LCK, IL2RG, IL2RB, ITK, CCR1, LAPTM5	8/30		0/30	miR-146a, miR-155, miR-21, miR-218, miR-22, miR-223, miR-224	7/17
22	MMP2, MMP11, THBS2, VCAN, COL1A1, LOXL2, ADAM12, DPT, ECM1	9/27	FN1, MMP1, MMP2, PLAU, SPARC	5/27	miR-152, miR-214, miR-22	3/6
25	MCM2, FEN1, PCNA, MYBL2, FBXO5	5/29		0/29	miR-18a, miR-25, miR-29a, miR-93	4/8
26	MAD2L1, PLK1, FEN1, PCNA, UCHL5, CCNA2, CCNB1, FBXO5, RAP1GDS1, RAN	10/44	MAD2L1	1/44	let-7b, miR-101, miR-17, miR-18a, miR-19a, miR-25, miR-29a, miR-93	8/17
27	MMP14, MMP2, MMP11, BGN, COL16A1, THBS2, THBS1, VCAN, COL1A1, PDGFRB, COL1A2, LOXL2, ADAM12, ECM1, COL11A1, TWIST1, SFRP4, LOX, TAGLN, LHFP	20/55	FN1, MMP14, MMP2, PLAU, SERPINF1, SPARC	6/55	miR-127, miR-145, miR-152, miR-199a, miR-214, miR-22	6/12
31	CD82, CTSB, STAT3, TNFSF10, GBP1, EPB41L3, CXCL10, CASP1, LYN, SPP1, LAPTM5, IRF1, CTSL1, TACC1, S100A13, CAPG	16/65	ACVR2B, CD82, CTSB, CTSD, DPYD, RAB25, SERPINF1, STAT3, TNFSF10	9/65	miR-125b, miR-130a, miR-146a, miR-155, miR-17, miR-183, miR-20a, miR-20b, miR-21, miR-218, miR-22, miR-223, miR-224, miR-335	14/23
33	AURKA, CDC20, TOP2A, PLK1, ASPM, BUB1, FOXM1, EfCT2, KIF14, CCNA2, BUB1B, FBXO5, UBE2C, TK1, CENPF, TACC3, CKS2	17/57	AURKA, CDC20, MKI67, TOP2A	4/57	let-7b, miR-101, miR-106b, miR-130b, miR-146b, miR-16, miR-17, miR-18a, miR-19a, miR-20b, miR-25, miR-29a, miR-93	13/31

^*a*^Num1 represents the number of cancer genes / the number of all genes in a module,

^*b*^Num2 the number of ovarian cancer genes / the number of all genes in a module, and

^*c*^Num3 the number of ovarian cancer miRNAs / the number of all miRNAs in a module.

Because our approach includes genes belonging to multiple modules, we calculated the overlap ratios of genes and miRNAs among the modules. The overlap ratio is defined as ∣*m*_1_ ∩ *m*_2_∣/∣*m*_1_ ∪ *m*_2_∣, where *m*_1_ and *m*_2_ are the number of genes or miRNAs in module 1 and module 2, respectively. Figs. S4 and S5 show the overlap ratios among the modules. The average overlap ratios of genes were 1.6% and 2.0% for ovarian cancer and GBM, respectively, and the average overlap ratios of miRNAs were 7.3% and 14.2% for ovarian cancer and GBM, respectively. The overlap ratios of miRNAs are higher than the overlap ratio of genes, indicating that a miRNA regulates many genes involved in several pathways.

### Relationship among genes, miRNAs and TFs in modules

As described in the Methods section, we examined the direct relationships between genes and miRNAs and their indirect relationships through TFs in the identified modules, as well as experimentally validated interactions between genes and miRNAs. For the ovarian cancer modules, we tested the direct relationship based on whether potential targets of a miRNA in the module were enriched for the genes in the same module using MicroCosm. Table 2 shows 8 miRNAs and their target genes in 12 ovarian cancer modules. For example, in Table 2, let-7b may directly regulate several genes (ESPL1, DEPDC1, BUB1B, AURKB and UBE2C) in module 33. Additionally, 19 gene-miRNA direct interaction pairs that were experimentally confirmed in miRTarbase are shown in Table 3. Previously, it was confirmed using a luciferase reporter assay and the western blot method that miR-93 targets E2F1. Also, it was confirmed using a luciferase reporter assay that miR-125b targets BCL3 in ovarian cancer cell [41]. All 156 gene-miRNA interaction pairs experimentally validated in miRTarbase are shown in Table S7, which includes both direct and coexpression based interactions.

**Table 2:** miRNAs regulate genes in ovarian cancer modules.

Module ID	miRNA	m^*a*^	k^*b*^	x^*c*^	*p*-value^*d*^	Genes
2	miR-185	757	60	9	1.21E-02	RASSF4,CTSH,POU2AF1,PSCD4,AIM2,LCK
3	miR-7	997	35	7	2.26E-02	BUB1,ASPM,SEC61A2,CDK2,COQ7,SYT17
6	miR-7	997	34	7	1.94E-02	BUB1,POLE2,KIF23,CDK2,MCM6
7	miR-331	892	25	5	3.38E-02	PYCRL,SHARPIN,PLEC1
13	miR-7	997	78	12	2.60E-02	BUB1,POLE2,MCM6,BXDC2,RBBP9,SMARCB1,GAD1
15	miR-9	863	35	6	3.62E-02	MXD3,C6orf134,DDX25
15	miR-29b	1266	35	9	8.60E-03	DNAH7,COL4A6,DDX25
17	miR-29a	1038	29	7	1.00E-02	MYBL2,TDG,PPIE,MSH2
17	miR-29b	1266	29	7	2.75E-02	TIMELESS,TDG,FAF1
25	miR-7	997	29	8	1.91E-03	FBXO5,POLE2,KIF23,MCM6
26	miR-29b	1266	44	10	1.41E-02	CHEK1,TIMELESS,RIT1,DYNLT1
29	miR-93	946	23	5	3.03E-02	CDCA8,MED8,RLF
30	let-7b	1050	26	6	2.19E-02	EHMT2,RNF5,RGL2
33	let-7b	1050	57	11	9.28E-03	ESPL1,DEPDC1,BUB1B,UBE2C,AURKB

^*a*^m,

^*b*^k, and

^*c*^x represent the number of genes regulated by the miRNA collected from MicroCosm, the number of genes in the module, and the number of genes regulated by the miRNA in the module, respectively. The significant numbers of genes in each module are regulated by the miRNA, and the significances are shown in

^*d*^ *p*-value.

**Table 3:** Experimentally validated gene-miRNA interactions with strong evidence from miRTarbase in ovarian cancer modules.

Module ID	Gene	miRNA	Validation Method	PubMed ID
2	EPB41L3	miR-223	Luciferase reporter assay, Western blot	21628394
2	MEF2C	miR-223	Luciferase reporter assay	18278031
2	MEF2C	miR-21	Immunofluorescence, In situ hybridization, Luciferase reporter assay	21170291
3	E2F1	miR-93	Luciferase reporter assay, Western blot	19486339
3	E2F1	miR-106b	Luciferase reporter assay, Western blot	19486339
3	EZH2	miR-25	Luciferase reporter assay, qRT-PCR, Western blot	22399519
5	CREBZF	miR-221	Reporter assay, Microarray	20018759
6	CCNE2	miR-26a	Luciferase reporter assay, Western blot	19524505
13	CDC42	miR-29a	Luciferase reporter assay, Western blot	19079265
14	BCL3	miR-125b	Luciferase reporter assay	20658525
14	HK2	miR-125b	Luciferase reporter assay, qRT-PCR	22593586
18	NASP	miR-29a	Luciferase reporter assay, Western blot	22080513
26	CCNA2	let-7b	Immunoblot, Immunofluorescence, Luciferase reporter assay, qRT-PCR	18379589
27	TWIST1	miR-214	Luciferase reporter assay, qRT-PCR, Western blot	22540680
27	MMP14	miR-145	Reporter assay, Microarray	21351259
31	STAT3	miR-21	Western blot, Other	20048743
31	STAT3	miR-20b	qRT-PCR, ELISA, ChIP, Western blot	20232316
31	EPB41L3	miR-223	Luciferase reporter assay, Western blot	21628394
31	TNFSF10	miR-222	Western blot	18246122
32	TOB1	miR-218	Luciferase reporter assay	23060446
33	CCNA2	let-7b	Immunoblot, Immunofluorescence, Luciferase reporter assay, qRT-PCR	18379589

**Figure 3:**
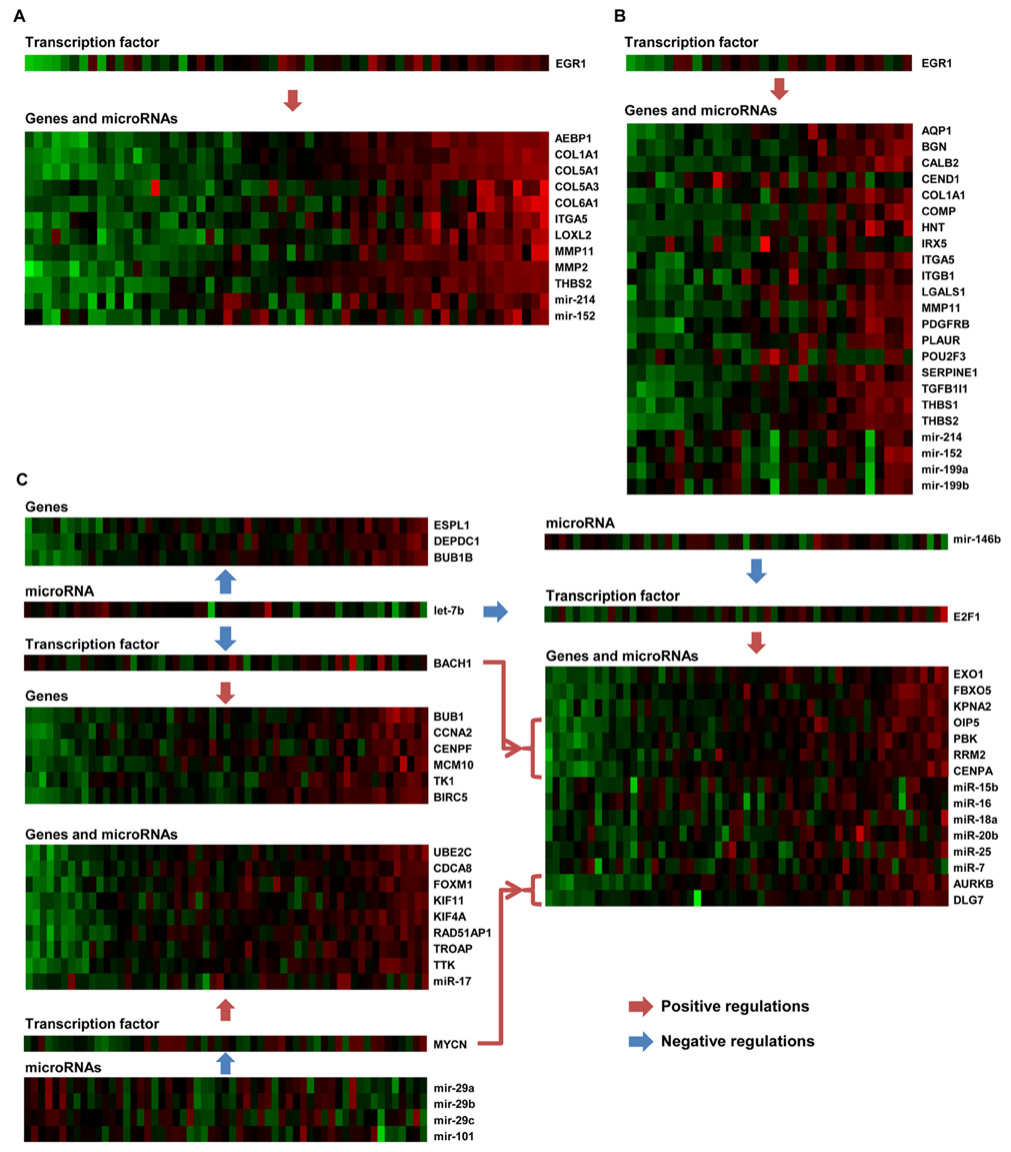
Regulations among genes, miRNAs, and TFs in ovarian cancer modules. For three ovarian cancer modules-22 (A), 8 (B), and 33 (C)-the expression values of genes, miRNAs, and TFs are shown. Arrows represent genes and miRNAs regulated by TFs or other miRNAs. Genes and miRNAs are members of each module, but TFs do not belong to the modules.

Table S8 shows the indirect relationships in 19 ovarian cancer modules, where genes and miRNAs are co-regulated by the same TF. Note that some TFs are not members of the modules. Regulation of miRNAs by TFs is validated by literature evidence (PubMed IDs are shown in the table), and the significance of the regulations of the genes in the modules by TFs was demonstrated using *p*-values that were obtained based on the ChIP-X database [26]. In many modules, one TF regulates multiple miRNAs and multiple genes. For example, Fig. 3 (A) shows ovarian cancer module 22, in which the TF EGR1 positively regulates several genes (AEBP1, COL1A1, COL5A1, COL5A3, COL6A1, ITGA5, LOXL2, MMP11, MMP2 and THBS2) and miRNAs (miR-214 and miR-152). Fig. 3 (B) shows ovarian cancer module 8, in which EGR1 positively regulates several genes (AQP1, BGN, CALB2, CEND1, COL1A1, COMP, HNT, IRX5, ITGA5 and ITGB1) and miRNAs (miR-214, miR-152, miR-199a and miR-199b) in the module at the same time. In both cases, we can infer that the genes and miRNAs are indirectly related via EGR1.

Table S9 shows another type of indirect relationship in ovarian cancer, where miRNAs regulate TFs, and the TFs regulate genes in 14 ovarian cancer modules. Regulation of TFs by miRNAs was found in the literature, and is shown in the third column of the table. One example of this relationship is shown in Fig. 3 (C): let-7b directly regulates the TF BACH1, and BACH1 regulates several genes (BUB1, CCNA2, CENPF, MCM10, BIRC5, TK1, OIP5, KIF11, RRM2 and CENPA); miR-156b and let-7b regulate the TF E2F1, which regulates several genes and other miRNAs in the modules; and miR-101, miR-29a, miR-29b and miR-29c regulate the TF MYCN, which regulates genes in the module. This module is related to ovarian cancer-related pathways such as those involved in mitosis and the cell cycle.

**Figure 4:**
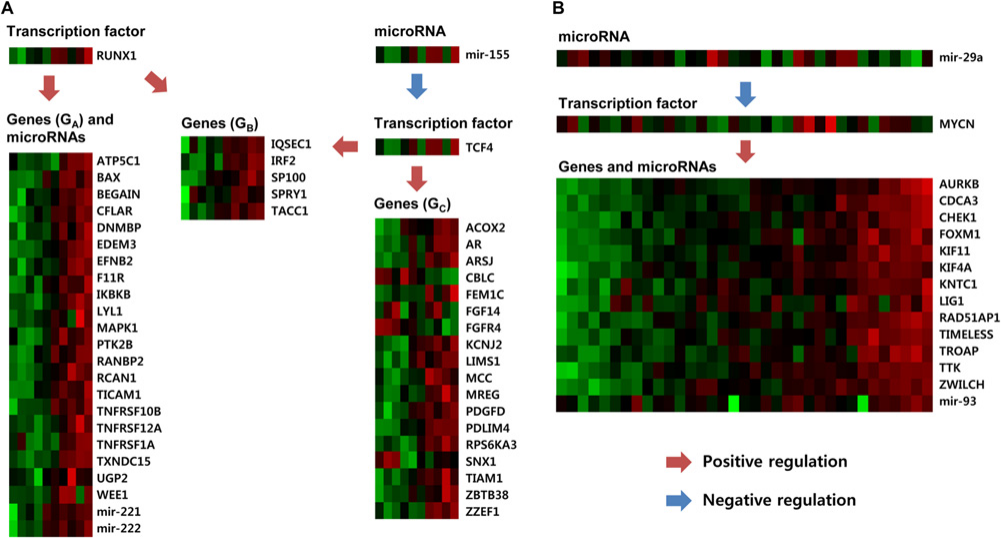
Regulations among genes, miRNAs, and TFs in GBM modules. For two GBM modules, 11 (A) and 5 (B), the expression values of genes, miRNAs, and TFs are shown. Arrows represent genes and miRNAs regulated by TFs or other miRNAs. Genes and miRNAs are members of each module, but TFs do not belong to the modules.

Similarly, relationships among genes, miRNAs, and TFs in GBM modules are shown in Fig. 4 and in Tables S10, S11, and S12. Table S10 shows 8 miRNAs and their target genes in 12 GBM modules. Genes targeted by miRNAs were highly enriched in these modules. In addition, Tables S11 and S12 show indirect relationships between genes and miRNAs through TFs. Fig. 4 (A) shows one example of an indirect relationship in GBM module 11, where even though genes might not be directly regulated by miRNAs, they are indirectly related via two TFs: RUNX1 and TCF4. For ease of reference, the genes in module 11 were divided into three groups (*G*_*A*_, *G*_*B*_ and *G*_*C*_): the TF RUNX1 positively regulates miR-221, miR-222, and genes in *G*_*A*_ and *G*_*B*_; miR-155 negatively regulates the TF TCF4; and TCF4 positively regulates genes in *G*_*B*_ and *G*_*C*_. Similarly, Fig. 4 (B) shows that miR-29a regulates the TF MYCN, which regulates several genes and miR-93 in GBM module 5. Experimentally validated 438 gene-miRNA interactions from the miRTarbase are shown in Table S13, including 112 direct interactions. In addition, we verified in the literature that miR-21 interacts with BMPR2 and miR-222 interacts with ICAM1 in GBM cell [42].

Fig. S6 summarizes these direct and indirect relationships in the ovarian cancer and GBM modules. These analyses show that, in total, 91% (30 out of 33) of ovarian cancer modules and 94% (51 out of 54) of GBM modules can be explained by direct regulations or indirect relationships, which allows us to understand how genes are regulated in modules.

### Pathway enrichment tests and network analysis for ovarian cancer

To determine the functional relevance of modules identified in ovarian cancer, we performed pathway enrichment tests for GO biological processes, KEGG pathways, and BioCarta pathways. We found that 16 out of 33 modules (48.4%) were enriched in at least one function. Table 4 presents enriched functions or signaling pathways for selected modules. Several modules have many enriched functions or pathways related to ovarian cancer, such as the p53 signaling pathway [43], ECM receptor interactions [44], and cell cycles [45]. Tables S14, S15, and S16 present lists of all enriched pathways. As mentioned previously, on average, 19.1% of genes in our modules were cancer genes and 5.7% were ovarian cancer genes. Our further manual literature search revealed that most of the cancer genes in several modules are also ovarian cancer-related genes, suggesting that cancer genes in the modules have a high potential to be ovarian cancer-related genes. In addition, most of the enriched modules had at least one ovarian cancer gene, supporting the idea that all enriched modules might be related to ovarian cancer. Therefore, we extensively analyzed modules 22 and 8 because module 22 has a relatively high fraction of ovarian cancer genes (12.8%) and cancer genes (28.2%) and is enriched for important pathways in ovarian cancer, and module 8 also contains a high fraction of ovarian cancer genes (18.5%), cancer genes (33.3%), and three enriched pathways related to ovarian cancer.

**Table 4:** Ovarian cancer modules with enriched pathways.

Module ID^*a*^	Pathways^*b*^	Related genes^*c*^	# of genes	*q*-values
2	Cytokine-Cytokine Receptor Interaction	CXCL13, LTB, CXCL11, IL18, CXCL9, CD27, CXCL10, CCR5	8	7.16E-04
2	Chemokine Signaling Pathway	CXCL13, CXCL11, CXCL9, VAV1, CXCL10, HCK, DOCK2, CCR5, LYN, FGR	10	8.02E-07
2	Cell Adhesion Molecules Cams	ICOS, SIGLEC1, ITGB2, CD4	4	4.45E-02
2	Toll-Like Receptor Signaling Pathway	CXCL11, CXCL9, CXCL10, SPP1	4	1.92E-02
2	Natural Killer Cell Mediated Cytotoxicity	VAV1, LCP2, ITGB2, TYROBP, LCK	5	6.05E-03
2	T-Cell Receptor Signaling Pathway	ICOS, VAV1, LCP2, CD4, LCK	5	5.81E-03
2	B-Cell Receptor Signaling Pathway	BLNK, VAV1, BTK, LYN	4	9.50E-03
2	Defense Response	CXCL11, CXCL9, BLNK, CXCL10, CLEC5A, LSP1, CCR5, TYROBP	8	1.93E-03
2	Immune Response	CXCL13, IL18, BLNK, CD96, POU2AF1, AIM2, PSMB10, LCP2, CCR5, ARHGDIB, CD74	11	1.41E-06
2	T-Cell Activation	IL18, CD4, LCK	3	3.49E-02
2	Response to Wounding	CXCL11, CXCL9, BLNK, CXCL10, CCR5	5	4.65E-02
2	Phosphorylation	HCK, ITGB2, BTK, LCK, LYN, FGR	6	4.80E-02
2	Cellular Defense Response	CXCL9, CLEC5A, LSP1, CCR5, TYROBP	5	7.74E-04
6	Cell Cycle	CHEK1, CDC7, CCNE2, MCM4, CDK2, MCM6, CDC25A, MCM2, PCNA, BUB1	10	2.97E-11
6	p53-Signaling Pathway	CHEK1, CCNE2, CDK2	3	1.95E-02
6	MCM Pathway	MCM4, CDK2, MCM6, MCM2	4	3.42E-05
6	Cell Cycle Process	CHEK1, CDC7, TIMELESS, CDK2, KIF15, KNTC1, KIF23, BUB1, RACGAP1, CDKN3	10	9.74E-09
6	Mitotic Cell Cycle	CDC7, CDK2, KIF15, KNTC1, KIF23, BUB1, CDKN3	7	1.04E-05
6	Response to DNA Damage Stimulus	CHEK1, POLE2, FEN1, MSH6	4	3.44E-02
6	Regulation of Cell Cycle	CHEK1, CDC7, CCNE2, TIMELESS, CDK2, KNTC1, CDC25A, BUB1, CDKN3	9	9.44E-08
6	Regulation of Cell Proliferation	CHEK1, CDC7, TIMELESS, CDK2, CDKN3	5	3.44E-02
8	TGF-Beta Signaling Pathway	INHBA, COMP, THBS2, THBS1	4	6.99E-03
8	Focal Adhesion	MYLK, COMP, ITGB1, THBS2, THBS1, COL3A1, COL1A1, FN1, PDGFRB, COL1A2, ITGA5	11	8.00E-10
8	ECM-Receptor Interaction	COMP, ITGB1, THBS2, THBS1, COL3A1, COL1A1, FN1, COL1A2, ITGA5	9	4.33E-10
8	Complement and Coagulation Cascades	SERPINE1, PLAU, PLAUR	3	4.44E-02
22	Focal Adhesion	COL5A3, COL1A1, COL6A1, COL5A1, THBS2, FN1, ITGA5, COL3A1	8	3.94E-07
22	ECM-Receptor Interaction	COL5A3, COL1A1, COL6A1, COL5A1, THBS2, FN1, ITGA5, COL3A1	8	8.02E-10
22	Proteolysis	MMP11, MMP1, CTSK, PLAU, MMP2	5	2.24E-02
26	G2 Pathway	PLK1, CCNB1, CHEK1	3	8.31E-03
26	Regulation of Cell Cycle	FBXO5, BIRC5, CCNA2, MAD2L1, TIMELESS, CHEK1, GMNN, CDC7	8	1.14E-05
33	Cell Cycle	BUB1, TTK, ESPL1, PLK1, BUB1B, CCNA2, CDC20, CCNB2	8	8.53E-06
33	Microtubule Based Process	TTK, KIF11, KIF23, PRC1, NUSAP1, KIF4A, KPNA2	7	2.64E-06
33	Regulation of Cell Cycle	BUB1, FBXO5, TTK, BUB1B, UBE2C, NUSAP1, CCNA2, CKS2, BIRC5	9	2.51E-06

^*a*^Several ovarian cancer modules are shown with enriched

^*b*^pathways and

^*c*^cancer genes. We selected these modules based on the importance of terms and the ratios of cancer genes and ovarian cancer genes.

**Figure 5:**
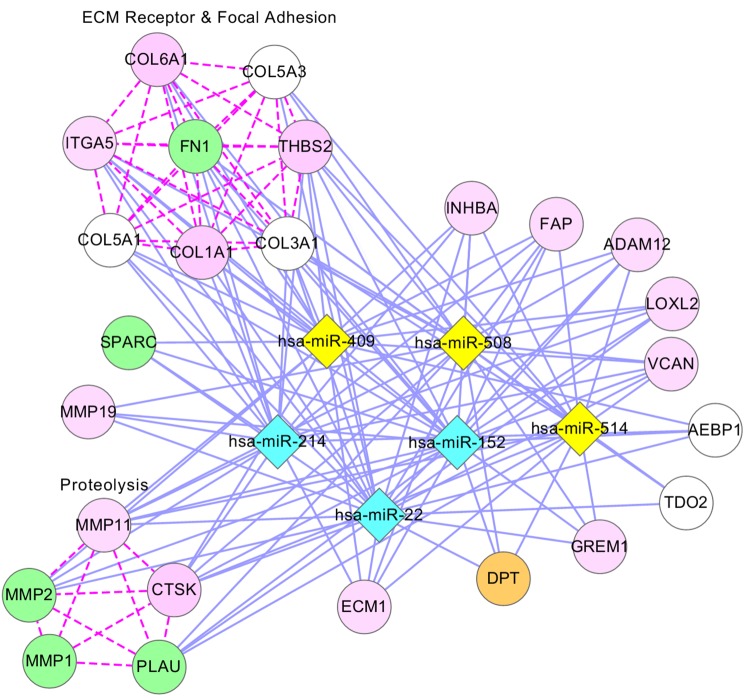
Network presentation of module 22 in ovarian cancer. In this network, diamonds represent miRNAs: sky-blue nodes for ovarian cancer miRNAs from the HMDD database, pink nodes for ovarian cancer miRNAs supported by the literature, and yellow nodes for the remaining miRNAs. Genes are represented by circles: pink nodes for ovarian cancer genes validated by the literature, green nodes for ovarian cancer genes validated by the DDOC database, orange nodes for cancer genes, and white nodes for the remaining genes. A blue solid line indicates that the MCC value between a gene and a miRNA is larger than 0.3. A purple line indicates that the linked genes are enriched together with at least one function. For example, COL6A1, COL5A3, THBS2, FN1, COL1A1, COL5A1, COLA1A, and COL3A1 are enriched with at least one function together (ECM receptor pathway or Focal adhesion pathway). Table S17 presents PubMed identifiers for ovarian cancer genes in pink nodes.

Fig. 5 shows a network representation of module 22, where 25 genes (2 genes are not shown) and 6 miRNAs are presented as nodes. In this module, 5 genes (FN1, MMP2, MMP1, PLAU, and SPARC), colored in green, were identified as ovarian cancer-related genes in the DDOC database. Moreover, the literature showed that 14 genes (ITGA5, COL6A1, THBS2, COL1A1, MMP19, MMP11, CTSK, ECM1, GREM1, VCAN, LOXL2, ADAM12, FAP, and INHBA), colored in pink, are ovarian cancer genes (shown in Table S17) and that these genes have high-average SCC values with at least one miRNA colored in sky blue. Most of the genes enriched in ECM receptor interaction, focal adhesion and proteolysis pathways are green or pink nodes, suggesting that these pathways are closely related to ovarian cancer. The literature confirms that these pathways are related to ovarian cancer [44, 46, 47]. In this module, COL3A1 might be related to ovarian cancer, as it is a known cancer gene targeted by all ovarian cancer miRNAs and belongs to ECM receptor and focal adhesion pathways. COL5A1 and COL5A3 are also likely to be ovarian cancer genes: they are targeted by ovarian cancer miRNAs and enriched in the above pathways, although they are not known cancer genes. Similarly, DPT also might be an ovarian cancer gene, as it is a cancer gene and is targeted by all ovarian cancer miRNAs. Evidence in the literature shows that the previously known ovarian cancer-related miRNAs miR-152, miR-22, and miR-214 are also related to enriched pathways in this module: miR-152 is involved in ECM-receptor-interaction [48, 49], and miR-22 and miR-214 regulate the AKT/PTEN pathway and the p53 signaling pathway [50, 51], which are highly related to the ECM-receptor, focal adhesion and proteolysis pathways [52–55]. These observations support the idea that genes and miRNAs interact with each other and play critical roles at the pathway level.

**Figure 6:**
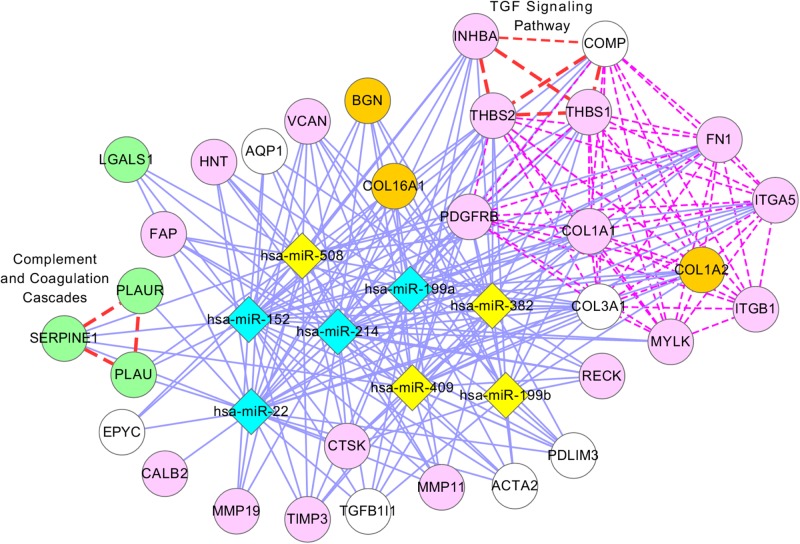
Network presentation of module 8 in ovarian cancer. The description of this network is the same as in Fig. 5 except that red lines are used to represent two enriched pathways (complement and coagulation cascades pathway, and TGF signaling pathway).

Fig. 6 illustrates module 8, which contains 34 genes and 8 miRNAs (5 genes are not shown). Because several genes and miRNAs are duplicated in module 22, the same pathways (ECM receptor and focal adhesion) are enriched. However, other important pathways in ovarian cancer, such as the TGF-beta signaling pathway and the complement and coagulation cascades pathway, are also enriched [56]. From this module, COL16A1, COL3A1, and COL1A2 are likely to be ovarian cancer genes, as they are cancer genes and are enriched with at least one pathway containing ovarian cancer genes. For miRNAs, several articles support that miR-199a, miR-199b, miR-214, and miR-382 are involved in the TGF-beta signaling pathway [57–60], and that miR-22 regulates the AKT/PTEN pathway [50, 51], which is closely related to the TGF-beta signaling pathway in several cancers [50, 61].

### Pathway enrichment tests and network analysis for GBM

We performed pathway enrichment tests for modules identified from the GBM data set. Of 54 modules tested, 40 (74%) were enriched with at least one function. Several modules had many enriched functions or pathways related to GBM, such as the p53 signaling pathway [62], the ERBB signaling pathway [63], and the MAPK signaling pathway [64]. Tables S18, S19, and S20 present lists of enriched pathways. As mentioned above, on average, 23.2% of genes in the modules were cancer genes, and 1.2% were GBM genes. A list of GBM genes was extracted from two articles [34, 35]. Similarly to ovarian cancer, the literature results demonstrated that most of the cancer genes in our modules were also GBM-related genes, suggesting that cancer genes in the modules are likely to be related to GBM. We extensively analyzed module 11 because this module contained many GBM-related genes and pathways.

**Figure 7:**
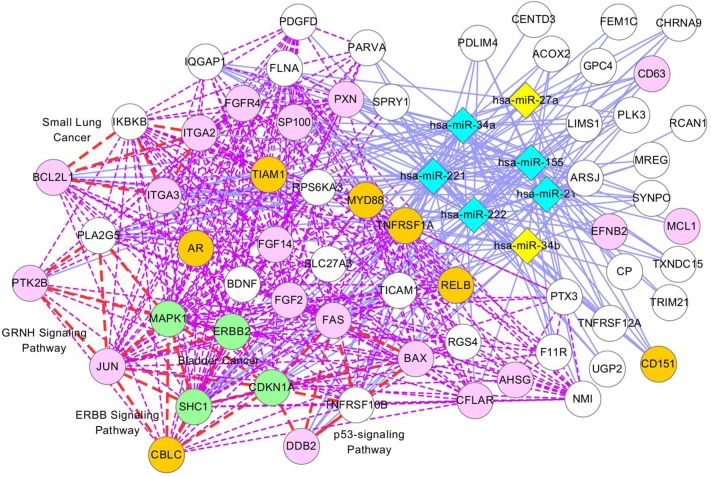
Module 11 in GBM. The description of this network is the same as in Fig. 5, except that green nodes indicate GBM genes validated by two articles [34, 35], and pink nodes indicate GBM genes validated by the literature in PubMed. Table S17 presents PubMed identifiers for GBM genes.

Fig. 7 illustrates a network presentation of module 11, where 74 genes (15 genes are not shown) and 7 miRNAs are presented as nodes. In this module, 4 genes (MAPK1, CDKN1A, SHC1, and ERBB2), colored in green, are GBM genes that were validated by the literature. Most of the genes on the left side of Fig. 7 are cancer genes and are enriched with at least one pathway, including the p53, ERBB, and GRNH signaling pathways. CBLC might be involved in the development of GBM because it is a cancer gene and is contained in the ERBB signaling pathway, an important GBM-related pathway that includes four GBM genes in this module. Additionally, the literature shows that miRNAs in this module function together in the enriched pathways: miR-34a, miR-135, miR-21, mi-222, miR-221, miR-27a, and miR-34b are involved in the p53 signaling pathway [65–71] and the MAPK signaling pathway [71–75], and miR-34a, miR-135, miR-21, miR-222, and miR-221 are involved in the ERBB signaling pathway [76–79].

### Cancer subtypes of modules

In Bell *et al.* [8], ovarian cancer was classified into four ovarian cancer subtypes depending on the expression levels of marker genes: “immunoreactive,” “proliferative,” “differentiated,” and “mesenchymal.” The immunoreactive subtype was identified by the chemokine receptor CXCR3 and its ligands CXCL11 and CXCL10, indicating that considerable expression changes of these genes are important markers for identifying the subtype. The proliferative subtype was identified by the overexpression of transcription factors HMGA2 and SOX11, proliferation marker genes such as MCM2 and PCNA, and underexpression of MUC1 and MUC16, which are known ovarian tumor marker genes. The differentiated subtype was identified by overexpression of MUC16, MUC1 and SLPI. Finally, the mesenchymal subtype was identified by overexpression of FAP and ANGPTL2.

**Figure 8:**
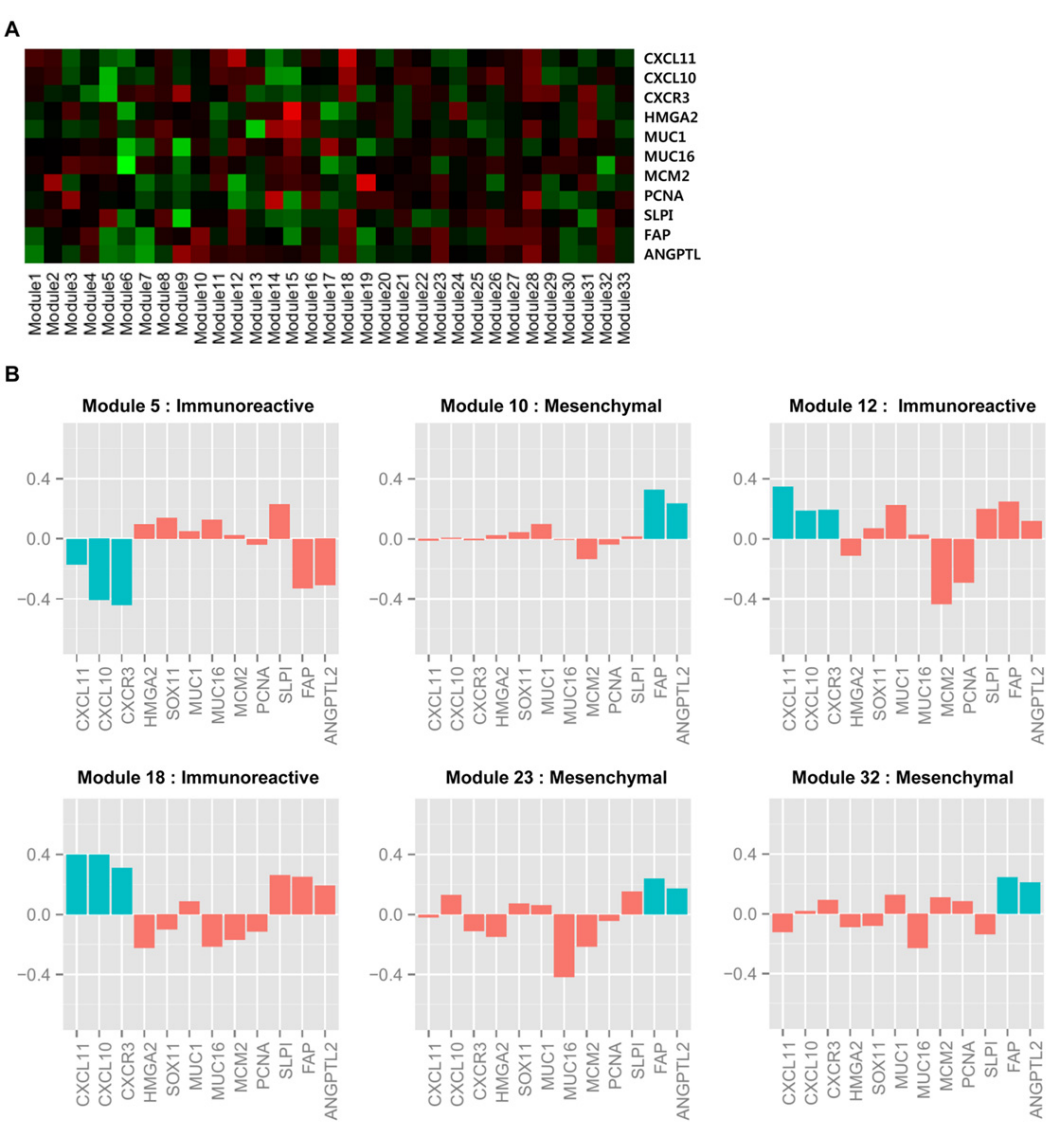
Expression levels of ovarian cancer subtype marker genes. (A) Heat map of the means of marker gene expression levels for 32 ovarian cancer modules. Red indicates overexpression of genes, and green indicates underexpression of genes. (B) Expression levels of marker genes of selected modules. Blue bars represent marker genes that determine the subtype and red bars represent other subtype marker genes.

In this study, we used the marker genes described above to determine which subtype was related to the majority of samples in the modules. First, we calculated the average expression level of the marker gene in the samples belonging to the module. Fig. 8 (A) represents the average expression levels of the 12 subtype marker genes across 33 ovarian cancer modules, showing that the expression levels of marker genes vary depending on the modules. As explained in the Methods section, we identified the cancer subtypes of samples by performing a hierarchical clustering with a dynamic tree cut (minModuleSize = 30) using gene expression data, and then we calculated the *p*-values of marker genes for the identified modules. As shown in Fig. 8 (B), among marker genes in the immunoreactive subtype, CXCL10 is underexpressed in module 5 (*p*-value: 0.08), and all of the marker genes (CXCL10, CXCL11 and CXCR3) are overexpressed in module 18 (*p*-values: 0.04, 0.02 and 0.67). Marker genes of the mesenchymal subtype are overexpressed in module 10 (*p*-values: 0.0003 and 0.0002), module 23 (*p*-values: 0.03 and 0.66), and module 32 (*p*-values: 0.02 and 0.09).

In Verhaak *et al.* [37], GBM was classified into four subtypes depending on the marker genes: “proneural,” “neural,” “classical,” and “mesenchymal.” It was observed that marker genes DLL3, NKX2–2, SOX2, ERBB3, and OLIG2 were overexpressed in the proneural subtype; marker genes FBXO3, GABRB2, SNCG and MBP were overexpressed in the neural subtype; FGFR3, PDGFA, EGFR, AKT2, and NES were overexpressed in the classical subtype; and CASP1, CASP4, CASP5, CASP8, ILR4, CHI3L1, TRADD, TLR2, TLR4, and RELB were overexpressed in the mesenchymal subtype. Note that marker genes of the GBM subtype were overexpressed in samples belonging to that subtype, while marker genes of other GBM subtypes were underexpressed in those samples.

**Figure 9:**
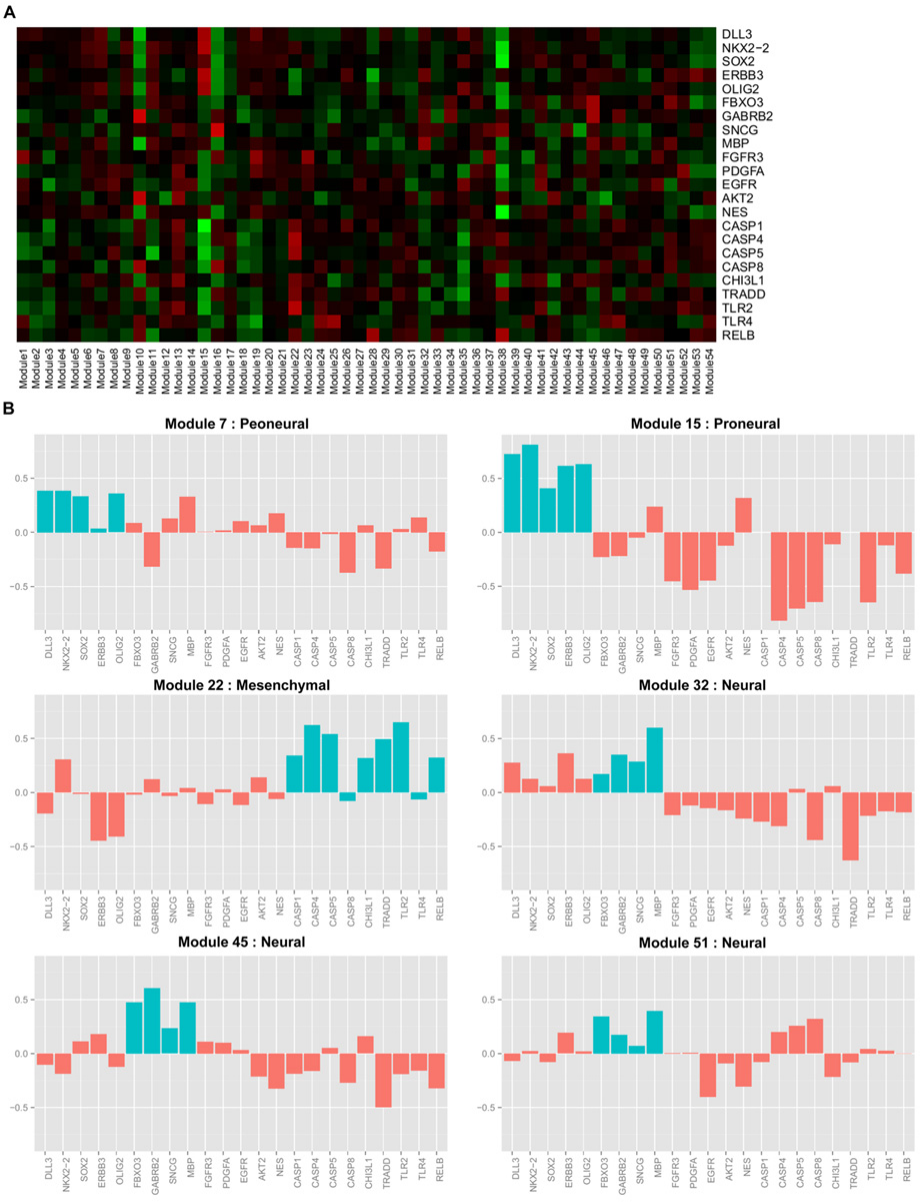
Expression levels of GBM subtype marker genes. (A) Heat map of the means of marker gene expression levels for 54 GBM modules. Red indicates overexpression of genes, and green indicates underexpression of genes. (B) Expression levels of marker genes of selected modules. Blue bars represent marker genes that determine the subtype, and red bars represent other subtype marker genes.

For GBM, we first calculated the average expression levels of marker genes. Fig. 9 (A) presents the average expression levels of the 23 subtype marker genes across 54 GBM modules, and shows the distinct expression levels of marker genes depending on the modules. Fig. 9 (B) shows 6 modules related to GBM marker genes. Marker genes in the proneural subtype (DLL3, NKX2–2, SOX2, ERBB3 and OLIG2) are overexpressed in module 7 (*p*-values: 0.01, 0.001, 0.0002, 0.07 and 0.004) and module 15 (*p*-values: 0.001, 0.00003, 0.002, 0.017 and 0.007). All of the marker genes in the mesenchymal subtype (CASP1, CASP4, CASP5, CASP8, ILR4, CHI3L1, TRADD, TLR2 and RELB), except TLR4, are overexpressed in module 22 (*p*-values: 0.001, 0.001, 0.003, 0.022, 0.048, 0.001, 0.036 and 0.0004). Two marker genes (SNCG and MBP) in the neural subtype are overexpressed in module 32 (*p*-values: 0.07 and 0.0001), all of the marker genes in the neural subtype (FBXO3, GABRB2, SNCG and MBP) are overexpressed in module 45 (*p*-values: 0.02, 0.02, 0.11 and 0.02), and two marker genes in the neural subtype (FBXO and MBP) are overexpressed in module 51 (*p*-values: 0.05 and 0.03). In addition, we obtained the subtype classification of GBM samples from Carro *et al.* [80], which shares 162 samples in common with our study (proneural: 62, neural: 22, classical: 35 and mesenchymal: 53). When we used these subtypes of samples for the enrichment of a particular subtype in our modules through a hypergeometric test, we confirmed that modules 32 and 45 are closely related to the neural subtype (*p*-values: 0.053 and 0.018).

### Performance comparisons

**Figure 10:**
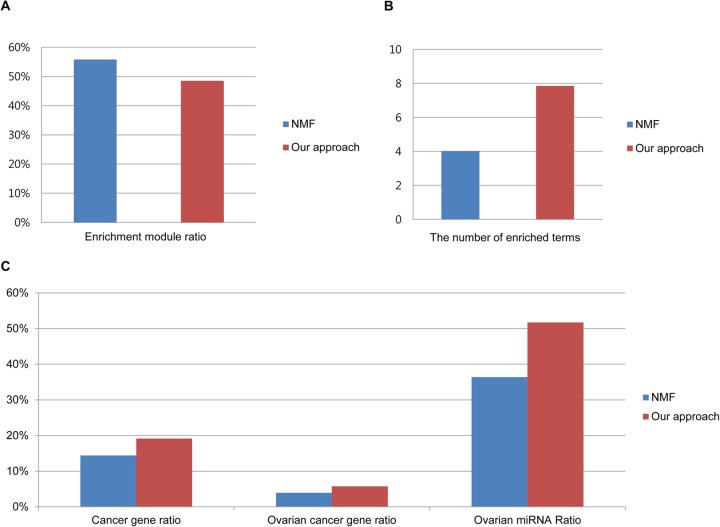
Performance comparisons. Comparison of modules identified using our approach and the NMF approach using ovarian cancer data. (A) The ratio of modules with at least one enriched function or pathway. (B) The average number of enriched functions in the identified modules. (C) The average ratios of cancer genes, ovarian cancer genes, and ovarian cancer miRNAs in the modules.

Zhang *et al.* [6] previously showed that their NMF approach outperformed the bi-clique algorithm proposed by Peng *et al.* [5]. Hence, we assessed the performance of our approach by comparing it with the NMF approach using TCGA ovarian cancer data. By applying our criteria to the modules generated from their approach, we selected modules having at least one gene and two human miRNAs. As a result, we removed 7 out of 50 modules. Fig. 10 shows that the ratio of modules containing enriched pathways in the NMF approach was slightly higher than the ratios of our modules. However, the average number of enriched pathways in our modules was larger than that in the NMF approach.

When we compared enriched pathways, two approaches had 43 common pathways, including ovarian cancer-related pathways such as the immune response, ECM-receptor, and TGF-Beta signaling pathways. In addition, 71 pathways were enriched only in our modules and 67 pathways only in the NMF modules, indicating that the two approaches most likely complement each other and capture different pathways related to ovarian cancer. Table S21 lists the common pathways and pathways enriched in each approach.

Additionally, modules identified by our approach contain more differentially expressed genes and cancer-related genes, because we primarily used differentially expressed genes, which provide more chances to incorporate cancer type-specific genes. In Zhang et al. [6], the modules contain a small fraction of differentially expressed genes and cancer-related genes, because 12,456 genes were used after filtering out genes with small absolute values and little variation. When we computed the overlap ratios of differentially expressed gene, most genes in our modules (79.4%, 617 out of 777 genes) were differentially expressed. However, modules generated by Zhang et al. [6] contained 28.3% (462 out of 1630 genes) differentially expressed genes on average. When we compared ratios of cancer genes, ovarian cancer genes, and ovarian cancer miRNAs in modules, our approach outperformed the NMF approach, as shown in Fig. 10.

The difference between the NMF approach and ours from a methodological viewpoint is that our approach can be more flexibly generalized to incorporate other regulatory components. In our approach, gene-sample modules are first constructed, and then miRNAs regulating genes are added to the modules (generating gene-miRNA modules). To demonstrate the range of our approach, we incorporated DNA copy number aberrations (CNAs) as another type of regulators in gene-sample modules. As a result, 23 out of 58 ovarian cancer gene-sample modules were explained by the regulation of CNAs, and 15 ovarian cancer gene-sample modules were explained by both miRNAs and CNAs. A detailed analysis regarding regulations by CNAs is provided in the Discussion section. By contrast, the NMF approach simultaneously incorporates gene-expression, miRNA expression, gene-gene interaction, and gene-miRNA sequence prediction information. Hence, when other regulators are included, they generate modules, where correlations between genes and regulators are simultaneously high. Indeed, in another paper from the same authors [81], they extended their NMF model to incorporate miRNAs, genes, and methylation of genes. In the generated modules, correlations of the expression levels of these three data sets were coordinately high due to a common basis matrix. Although it is a good approach, it omits modules representing the regulation of genes by a single type of regulators when incorporating multiple regulators.

Additionally, we compared our approach with the Context-Specific MicroRNA analysis (COSMIC) algorithm [82] using TCGA ovarian cancer data. COSMIC combines gene-miRNA target prediction information, mRNA expression, and miRNA expression data. The modules constructed by the COSMIC algorithm consisted of a single miRNA and genes, which indicated that several genes are regulated by the miRNA. When we applied a *q*-value threshold of < 0.05 to 479 identified modules, 102 modules were obtained. Since COSMIC generates modules consisting of a single miRNA, it is difficult to directly compare COSMIC with our approach. Hence, we applied pathway enrichment tests using GO biological processes and BioCarta and KEGG pathways with a *q*-value threshold of < 0.05 to these 102 modules, and observed that 25.5% (27 out of 102) of the modules were significantly enriched. This enrichment ratio is lower than the value obtained using our approach (48.4%). However, we need to consider that the higher enrichment ratio in our approach is partially because two studies developed algorithms using different data sets and different assumptions. We incorporated gene-gene interactions and indirect interactions among genes and miRNAs based on mRNA expressions and miRNA expressions, while COSMIC incorporated direct interactions using sequence information of genes and miRNAs, which might reduce false positive interactions. In spite of the differences, the two approaches had 26 common pathways, including ovarian cancer-related pathways such as the ECM-receptor, DNA replication, and the G2 pathway. In addition, 88 and 38 pathways were enriched only in our modules and only in the COSMIC algorithm, respectively. Table S22 lists the common pathways as well as the pathways enriched in each approach.

## Discussion

In this study, we developed an approach to constructing gene-miRNA modules by integrating genes and miRNAs. We applied our approach to ovarian cancer and GBM data sets from the TCGA project. Finally, we constructed 33 modules for ovarian cancer and 54 modules for GBM. We employed gene-gene interactions to include genes with high absolute correlations with genes in the modules, because some important cancer-related genes might not be clustered together by the biclustering algorithm or might not be differentially expressed. Fig. 2 shows that incorporating gene-gene interactions increased the performance in terms of the average number of enriched terms, the number of modules with at least one enriched pathway, and the ratios of cancer-related genes and cancer-related miRNAs. Although we used gene-gene interactions to add biologically relevant genes to modules in the proposed approach, gene-gene interactions can be used to filter out biologically irrelevant genes from modules to reduce false positives. However, because the currently available human gene-gene interactions are not complete, closely related but unidentified genes might also be filtered out. It is an important challenge to incorporate gene-gene interactions to reduce false positive genes in modules, while true relevant genes still remain. We will address this issue in our future work.

Because the identified modules might miss relevant interactions, we measured a potential false negative rate using miRTarbase. Let *N*_*G*_ be the number of common genes in the modules and miRTarbase, and let *N*_*G*_*interaction*_ be the number of common genes that interact with the same miRNAs in the modules and miRTarbase. Then, 1 - *N*_*G*_*interaction*_ / *N*_*G*_ might be a potential false negative rate. As a result, the rates of false negative were 0.789 (1–118/559) for ovarian cancer and 0.775 (1–316/1405) for GBM, respectively. However, the false negative rate should be adjusted when more accurate miRNA-gene interaction data become available, as this ratio is estimated based on all gene-miRNA interactions from miRTarbase and is not based on the specific cancer type and miRTarbase, which itself contains only a fraction of the gene-miRNA interactions.

In the Results section, we described a functional enrichment test of genes in modules using GO terms, KEGG, and BioCarta pathways. Although we employed a widely used approach in the enrichment test, a hypergeometric test followed by a Benjamini & Hochberg method for multiple comparison correction, several issues that require further improvement still remain. For the first issue, the Benjamini & Hochberg method hypothesizes independence of the terms, while the biological processes in various ontologies represent a hierarchical structure and inter-correlation. Thus, we performed an additional enrichment test for ovarian cancer and GBM modules using TANGO [83], which considers dependencies among biological pathways. It corrects *p*-values by computing the distribution of enrichment *p*-values in a large number of randomly generated gene sets of the same size. For ovarian cancer, 16 of 33 modules (48%) were enriched with at least one GO biological process term. For GBM, 28 out of 54 modules (48%) were enriched with at least one term. Tables S23 and S24 list all pathways enriched in each cancer. Further, Fig. S7 shows a comparison of the two approaches (a Benjamini & Hochberg method and TANGO) in terms of the ratio of enriched modules and the number of enriched terms. Although there are small differences in the two approaches, both approaches confirm that a large fraction of our identified modules were enriched with biologically relevant terms. For the second issue, because annotated pathways in GO terms, KEGG, and BioCarta pathways are still incomplete, validations on these pathways might miss biologically related sets of genes. An approach to reveal the pathways unannotated in GO, KEGG and BioCarta is to search for evidence about gene functions in the literature, and then to analyze them collectively. As part of such efforts, we manually searched scientific articles on ovarian cancer-related genes and GBM-related genes (Table S17), and relationships among genes, microRNAs, and TFs (Tables S8, S9, S11, and S12). However, this approach only solves the above problem partially so a more systematic approach is called for. Very few efforts, including LitVan (http://www.c2b2.columbia.edu/danapeerlab/html/software.html), have been developed to carry out an automatic literature search to connect genes with over-represented biological terms in millions of scientific articles. Although we attempted to analyze our modules using such tools, either there are no currently available tools or websites are not connected. Hence, we will further analyze modules for functional enrichments in the future.

Certain oncogenes and tumor-suppressor genes such as P53 and PTEN may play important roles in many cancer types rather than only in specific cancer type. Hence, we examined how many genes in the identified modules were specific to ovarian cancer or GBM. We collected 1393 genes from five cancer type specific databases: the DDOC [33], GBM genes from the literature [34, 35], the Cervical Cancer gene Database (CCDB) [84], the Dragon Database of Genes associated with Prostate Cancer (DDPC) [85], and Lung Cancer Gene Database (LUGEND). We refer to genes contained only in the DDOC as potentially ovarian cancer specific genes. Although these genes are not compared with genes from all types of cancers, it might helpful to remove common cancer genes. Among the 47 DDOC genes included in our ovarian cancer modules, 18 genes were potentially ovarian cancer specific genes. Similarly, among the 32 GBM genes included in our GBM modules, 7 genes were potentially GBM cancer specific genes. Lists of these cancer type specific genes are shown in Table S25.

The accuracy of the identified modules might be largely dependent on the quality of the data sets. In this study, we used TCGA microarray data sets, as in many previous reports they have been used to identify core genes and pathways significantly related to ovarian cancer and GBM. Additionally, when TCGA microarray data sets were compared to RNA-Seq data from the same samples, their expression values were highly correlated in most cases [86] confirming that these data sets are less dependent on a particular platform.

The proposed approach can be generalized to incorporate other regulatory components. To demonstrate the range of applicability of our approach and to provide additional support of biological relevance to the modules, we incorporated somatic DNA copy numbers from the paired patients of gene expression data. For this task, we downloaded TCGA level 3 data sets that provide segmented copy number ratio data compared to normal samples. We first recalculated the copy number aberration ratios for every 1 MB region and filtered out regions whose absolute copy number ratio values are less than 0.2, corresponding to 99.9% among all ratio values. Then, CNA regions were incorporated into gene-sample modules based on correlations between genes in modules and CNA regions. As a result, for the ovarian cancer modules, 23 out of 58 gene-sample modules were explained by the regulation of CNAs, and genes in 15 out of 33 gene-miRNA modules (45%) were also regulated by CNAs, as shown in Table S26. In particular, genes in several modules were located in the regulating CNA regions, indicating that the expression of genes in the modules might be directly affected by CNAs. DNA copy numbers in the chr 1: 32.1 MB - 53.4 MB region were highly correlated with genes in ovarian cancer module 9 with a PCC value of 0.301, and 13 out of 18 genes in the module (CDCA8, C1orf109, AK2, SNIP1, GNL2, RLF, TRIT1, YRDC, RRAGC, PPIE, PSMB2, MED8 and COL9A2) were located in this CNA region. Similarly, the DNA copy numbers in the chr 1: 180.6 MB - 247.9 MB region were highly correlated with genes in ovarian cancer module 23 with a PCC value of 0.319, and most of genes (14 out of 19 genes) in this module were located in this region. Additionally, for ovarian cancer module 29, DNA copy numbers in chr 1: 31.9 MB - 59.1 MB regions have a high correlation value (0.345) with gene in the module, and 78.3% of the genes are located in this region. For GBM, 26 out of 88 gene-sample modules were explained by regulation of the DNA copy numbers shown in Table S27, and 19 out of 54 gene-miRNA modules (35%) were commonly regulated by CNAs and miRNAs.

## Supporting Information

S1 Fig The SAMBA biclustering algorithm. In a SAMBA biclustering algorithm, it models gene expression data into a bipartite graph *G* = (*U*,*V*,*E*). In this graph, U is a set of samples, *V* is a set of genes and *E* is a set of edges between *U* and *V*. Nodes in one side are genes and nodes in the other side are samples. An edge is linked if the expression value of gene for sample is high or low. This means that gene expression level of *v* significantly changes in sample of *u*. In this model, we try to find a subgraph *G* ^′^ = (*U* ^′^,*V* ^′^,*E* ^′^) of *G*, where expression values of most genes in *V* ^′^ significantly change in most of samples in *U* ^′^, representing low values or high values. For example, genes *G*3, *G*4, and *G*5 in *V* ^′^ and *S*3 and *S*4 samples *U* ^′^ are constructed as a module (a circle colored in grey). For gene expression data normalized by a z-score, the SAMBA biclustering algorithm generates highly correlated gene-sample clusters that represent similar tendencies of gene expression changes for a subset of samples.
(TIF)

S2 Fig Distribution of the number of pathway terms. (TIF)

S3 Fig Performance comparison of gene-miRNAs modules for GBM. Performances of gene-miRNA modules generated from four cases (SCC with GGI information, SCC without GGI information, PCC with GGI information, and PCC without GGI information) are compared. For all cases, *x*-axis presents different percentages of candidate miRNAs (*T*%) among all miRNAs when constructing gene-miRNA modules. For each case, ratios of modules enriched with at least one pathway, the average number of enriched pathways, and ratios of cancer genes, GBM genes, and GBM miRNAs are shown.
(TIF)

S4 Fig Overlap ratio of genes in ovarian cancer modules. For every pairs of ovarian cancer modules, the overlap ratios of genes are defined as ∣*m*_1_ ∩ *m*_2_∣/∣*m*_1_ ∪ *m*_2_∣, where *m*_1_ and *m*_2_ are numbers of genes in module 1 and module 2, respectively.
(TIF)

S5 Fig Overlap ratio of genes in GBM modules. The description of the overlap ratios is the same as in Fig. S4.
(TIF)

S6 Fig Ratios of modules having four types of gene-miRNA relationships in ovarian cancer and GBM modules. *y*-axis represents the fraction of modules containing at least one corresponding relationship in the modules. ‘microCosm’ represents gene-miRNA interactions based on gene-miRNA sequences from the microCosm database, and ‘miRTarbase’ represents experimentally confirmed gene-miRNA relationships from miRTarbase. ‘TF regulates genes and miRNAs’ represents that genes and miRNAs are co-regulated by the same TF. ‘MiRNA regulates genes via TFs’ represents that miRNA regulates transcription factors and transcription factors regulates genes. ‘Union’ represents all four types of relationships.
(TIF)

S7 Fig Functional enrichment tests using a Benjamini & Hochberg method and a TANGO tool. GO biological terms were tested for functional enrichment of genes in ovarian cancer and GBM modules. Two multiple comparison correction approaches, a Benjamini & Hochberg (BH) method and a TANGO tool, were used after a hypergeometric test. GO terms employed in the two approaches were not exactly same, because TANGO, which were included in an EXPANDER software, uses its own collection of GO terms, and filters out redundant terms by computing an intersection between genes in two terms. However, in both approaches, ovarian cancer and GBM modules were enriched with GO terms. (A) and (C) show the ratios of modules enriched with at least one term for ovarian cancer and for GBM, respectively, and (B) and (D) represent the average numbers of enriched terms in identifiied modules for ovarian cancer and GBM, respectively.
(TIF)

S1 Table Genes in ovarian cancer modules. (PDF)

S2 Table MiRNAs in ovarian cancer modules. (PDF)

S3 Table Genes in GBM modules. (PDF)

S4 Table MiRNAs in GBM modules. (PDF)

S5 Table Cancer genes, ovarian cancer genes and ovarian cancer miRNAs in modules. ‘Num’ represents the number of ovarian cancer genes (or ovarian cancer miRNAs) / the number of all genes (or all miRNAs) in a module.
(PDF)

S6 Table Cancer genes, GBM genes and GBM miRNAs in modules. ‘Num’ represents the number of GBM genes (or GBM miRNAs) / the number of all genes (or all miRNAs) in a module.
(PDF)

S7 Table Exprementally validated gene-miRNA interactions in ovarian cancers. (PDF)

S8 Table Genes and miRNAs are co-regulated by the same TF in ovarian cancer modules. (PDF)

S9 Table MiRNA regulates TFs and the TFs regulate genes in the ovarian cancer modules. (PDF)

S10 Table MiRNAs regulate genes in GBM modules. The significant numbers of genes in each module are regulated by miRNAs, and the significances are shown in ‘p-value’. ‘m’, ‘k’, and ‘x’ represent the number of genes regulated by the miRNA collected from microCosm, the number of genes in the module, and the number of genes regulated by the miRNA in the module, respectively.
(PDF)

S11 Table Genes and miRNAs are co-regulated by the same TF in GBM modules. (PDF)

S12 Table MiRNA regulates TFs and the TFs regulate genes in GBM modules. (PDF)

S13 Table Exprementally validated gene-miRNA interactions in GBM. (PDF)

S14 Table Ovarian cancer modules with enriched GO terms. The significant numbers of genes in each module are enriched in gene ontology biological process, and the significance is shown in ‘p-value’. ‘m’, ‘k’, and ‘x’ represent the number of genes in the corresponding GO term, the number of genes in the module, and the number of genes belonging to the GO term in the module, respectively.
(PDF)

S15 Table Ovarian cancer modules with enriched pathways in KEGG. The significant numbers of genes in each module are enriched in KEGG pathway, and the significance is shown in ‘p-value’. ‘m’, ‘k’, and ‘x’ represent the number of genes in the corresponding KEGG pathway, the number of genes in the module, and the number of genes belonging to the pathway in the module, respectively.
(PDF)

S16 Table Ovarian cancer modules with enriched pathways in BioCarta. The significant numbers of genes in each module are enriched in BioCarta pathways, and the significance is shown in ‘p-value’. ‘m’, ‘k’, and ‘x’ represent the number of genes in the corresponding BioCarta pathway, the number of genes in the module, and the number of genes belonging to the BioCarta pathway in the module, respectively.
(PDF)

S17 Table Literature evidences for ovarian cancer-related genes from ovarian cancer modules 22 and 8, and GBM-related genes from GBM module 22. (PDF)

S18 Table GBM modules with enriched GO terms. The significant numbers of genes in each module are enriched in GO biological process, and the significance is shown in ‘p-value’. ‘m’, ‘k’, and ‘x’ represent the number of genes in the corresponding GO term, the number of genes in the module, and the number of genes belonging to the GO term in the module, respectively.
(PDF)

S19 Table GBM modules with enriched pathways in KEGG. The significant numbers of genes in each module are enriched in KEGG pathways, and the significance is shown in ‘p-value’. ‘m’, ‘k’, and ‘x’ represent the number of genes in the corresponding pathway, the number of genes in the module, and the number of genes belonging to the pathway in the module, respectively.
(PDF)

S20 Table GBM modules with enriched pathways in BioCarta. The significant numbers of genes in each module are enriched in BioCarta pathways, and the significance is shown in ‘p-value’. ‘m’, ‘k’, and ‘x’ represent the number of genes in the corresponding pathway, the number of genes in the module, and the number of genes belonging to the pathway in the module, respectively.
(PDF)

S21 Table Comparisons of enriched pathways from the NMF approach and our approach. (PDF)

S22 Table Comparisons of enriched pathways from the COSMIC algorithm and our approach. (PDF)

S23 Table Ovarian cancer modules enriched with GO terms using a TANGO tool. (PDF)

S24 Table GBM modules enriched with GO terms using a TANGO tool. (PDF)

S25 Table Ovarian cancer specific genes and GBM specific genes in identified modules. (PDF)

S26 Table DNA copy number aberration regions that regulate gene expressions in ovarian cancer modules. (PDF)

S27 Table DNA copy number aberration regions that regulate gene expressions in GBM modules. (PDF)

## References

[1] Croce C (2009) Causes and consequences of microRNA dysregulation in cancer. Nat Rev Genet 10: 704–14. 10.1038/nrg2634 19763153 PMC3467096

[2] Bartel D (2004) Micrornas: genomics, biogenesis, mechanism, and function. Cell 116: 281–297. 10.1016/S0092-8674(04)00045-5 14744438

[3] Kent O, Mendell J (2006) A small piece in the cancer puzzle: microRNAs as tumor suppressors and oncogenes. Oncogene 25: 6188–96. 10.1038/sj.onc.1209913 17028598

[4] Lewis BP, Burge CB, Bartel DP (2005) Conserved seed pairing, often flanked by adenosines, indicates that thousands of human genes are microrna targets. Cell 120: 15–20. 10.1016/j.cell.2004.12.035 15652477

[5] Peng X, Li Y, Walters K, Rosenzweig E, Lederer S, et al (2009) Computational identification of hepatitis c virus associated microrna-mrna regulatory modules in human livers. Bmc Genomics 10: 373 10.1186/1471-2164-10-373 19671175 PMC2907698

[6] Zhang S, Li Q, Liu J, Zhou X (2011) A novel computational framework for simultaneous integration of multiple types of genomic data to identify microrna-gene regulatory modules. Bioinformatics 27: i401–i409. 10.1093/bioinformatics/btr206 21685098 PMC3117336

[7] McLendon R, Friedman A, Bigner D, Van Meir E, Brat D, et al (2008) Comprehensive genomic characterization defines human glioblastoma genes and core pathways. Nature 455: 1061–1068. 10.1038/nature07385 18772890 PMC2671642

[8] Bell D, Berchuck A, Birrer M, Chien J, Cramer D, et al (2011) Integrated genomic analyses of ovarian carcinoma. Nature 474: 609–615. 10.1038/nature10166 21720365 PMC3163504

[9] Joung J, Hwang K, Nam J, Kim S, Zhang B (2007) Discovery of microrna–mrna modules via population-based probabilistic learning. Bioinformatics 23: 1141–1147. 10.1093/bioinformatics/btm045 17350973

[10] Tran D, Satou K, Ho T (2008) Finding microrna regulatory modules in human genome using rule induction. BMC bioinformatics 9: S5 10.1186/1471-2105-9-S12-S5 19091028 PMC2638145

[11] Lim L, Lau N, Garrett-Engele P, Grimson A, Schelter J, et al (2005) Microarray analysis shows that some micrornas downregulate large numbers of target mrnas. Nature 433: 769–773. 10.1038/nature03315 15685193

[12] Krek A, Grün D, Poy M, Wolf R, Rosenberg L, et al (2005) Combinatorial microrna target predictions. Nature genetics 37: 495–500. 10.1038/ng1536 15806104

[13] Liu D, Liu J, Lin B, Liu S, Hou R, et al (2012) Lewis y regulate cell cycle related factors in ovarian carcinoma cell rmg-i in vitro via erk and akt signaling pathways. International journal of molecular sciences 13: 828–839. 10.3390/ijms13010828 22312289 PMC3269723

[14] Kulkarni A, Kingsbury S, Tudzarova S, Hong H, Loddo M, et al (2009) Cdc7 kinase is a predictor of survival and a novel therapeutic target in epithelial ovarian carcinoma. Clinical Cancer Research 15: 2417–2425. 10.1158/1078-0432.CCR-08-1276 19318489

[15] Jia L, Jin H, Zhou J, Chen L, Lu Y, et al (2013) A potential anti-tumor herbal medicine, corilagin, inhibits ovarian cancer cell growth through blocking the tgf-beta signaling pathways. BMC Complementary and Alternative Medicine 13: 33 10.1186/1472-6882-13-33 23410205 PMC3598193

[16] Devarajan K (2008) Nonnegative matrix factorization: an analytical and interpretive tool in computational biology. PLoS computational biology 4: e1000029 10.1371/journal.pcbi.1000029 18654623 PMC2447881

[17] Yamakuchi M, Lowenstein C (2009) MiR-34, SIRT1 and p53: the feedback loop. Cell Cycle 8: 712–5. 10.4161/cc.8.5.7753 19221490

[18] Feng Z, Zhang C, Wu R, Hu W (2011) Tumor suppressor p53 meets microRNAs. J Mol Cell Biol 3: 44–50. 10.1093/jmcb/mjq040 21278451 PMC3030969

[19] Bonnet E, Tatari M, Joshi A, Michoel T, Marchal K, et al (2010) Module network inference from a cancer gene expression data set identifies microRNA regulated modules. PLoS One 5: e10162 10.1371/journal.pone.0010162 20418949 PMC2854686

[20] Visvader J (2011) Cells of origin in cancer. Nature 469: 314–22. 10.1038/nature09781 21248838

[21] Prasad TK, Goel R, Kandasamy K, Keerthikumar S, Kumar S, et al (2009) Human protein reference database2009 update. Nucleic acids research 37: D767–D772. 10.1093/nar/gkn892 18988627 PMC2686490

[22] Tanay A, Sharan R, Shamir R (2002) Discovering statistically significant biclusters in gene expression data. Bioinformatics 18: S136–S144. 10.1093/bioinformatics/18.suppl_1.S136 12169541

[23] Vandin F, Upfal E, Raphael B (2012) De novo discovery of mutated driver pathways in cancer. Genome Res 22: 375–85. 10.1101/gr.120477.111 21653252 PMC3266044

[24] Friedman N, Linial M, Nachman I, Pe’er D (2000) Using bayesian networks to analyze expression data. Journal of computational biology 7: 601–620. 10.1089/106652700750050961 11108481

[25] Hsu SD, Tseng YT, Shrestha S, Lin YL, Khaleel A, et al (2014) mirtarbase update 2014: an information resource for experimentally validated mirna-target interactions. Nucleic acids research 42: D78–D85. 10.1093/nar/gkt1266 24304892 PMC3965058

[26] Lachmann A, Xu H, Krishnan J, Berger SI, Mazloom AR, et al (2010) Chea: transcription factor regulation inferred from integrating genome-wide chip-x experiments. Bioinformatics 26: 2438–2444. 10.1093/bioinformatics/btq466 20709693 PMC2944209

[27] Ashburner M, Ball C, Blake J, Botstein D, Butler H, et al (2000) Gene ontology: tool for the unification of biology. Nature genetics 25: 25 10.1038/75556 10802651 PMC3037419

[28] Kanehisa M, Goto S, Sato Y, Furumichi M, Tanabe M (2012) Kegg for integration and interpretation of large-scale molecular data sets. Nucleic acids research 40: D109–D114. 10.1093/nar/gkr988 22080510 PMC3245020

[29] Huret J, Le Minor S, Dorkeld F, Dessen P, Bernheim A (2000) Atlas of genetics and cytogenetics in oncology and haematology, an interactive database. Nucleic acids research 28: 349–351. 10.1093/nar/28.1.349 10592271 PMC102493

[30] Sjöblom T, Jones S, Wood L, Parsons D, Lin J, et al (2006) The consensus coding sequences of human breast and colorectal cancers. science 314: 268–274. 10.1126/science.1133427 16959974

[31] Akagi K, Suzuki T, Stephens R, Jenkins N, Copeland N (2004) Rtcgd: retroviral tagged cancer gene database. Nucleic acids research 32: D523–D527. 10.1093/nar/gkh013 14681473 PMC308748

[32] Futreal P, Coin L, Marshall M, Down T, Hubbard T, et al (2004) A census of human cancer genes. Nature Reviews Cancer 4: 177–183. 10.1038/nrc1299 14993899 PMC2665285

[33] Kaur M, Radovanovic A, Essack M, Schaefer U, Maqungo M, et al (2009) Database for exploration of functional context of genes implicated in ovarian cancer. Nucleic acids research 37: D820–D823. 10.1093/nar/gkn593 18790805 PMC2686485

[34] Furnari F, Fenton T, Bachoo R, Mukasa A, Stommel J, et al (2007) Malignant astrocytic glioma: genetics, biology, and paths to treatment. Genes & development 21: 2683–2710. 10.1101/gad.1596707 17974913

[35] Parsons DW, Jones S, Zhang X, Lin JCH, Leary RJ, et al (2008) An integrated genomic analysis of human glioblastoma multiforme. Science Signalling 321: 1807 10.1126/science.1164382 18772396 PMC2820389

[36] Lu M, Zhang Q, Deng M, Miao J, Guo Y, et al (2008) An analysis of human microrna and disease associations. PLoS One 3: e3420 10.1371/journal.pone.0003420 18923704 PMC2559869

[37] Verhaak RG, Hoadley KA, Purdom E, Wang V, Qi Y, et al (2010) An integrated genomic analysis identifies clinically relevant subtypes of glioblastoma characterized by abnormalities in pdgfra, idh1, egfr and nf1. Cancer cell 17: 98 10.1016/j.ccr.2009.12.020 20129251 PMC2818769

[38] Mischel PS, Shai R, Shi T, Horvath S, Lu KV, et al (2003) Identification of molecular subtypes of glioblastoma by gene expression profiling. Oncogene 22: 2361–2373. 10.1038/sj.onc.1206344 12700671

[39] Liang Y, Diehn M, Watson N, Bollen AW, Aldape KD, et al (2005) Gene expression profiling reveals molecularly and clinically distinct subtypes of glioblastoma multiforme. Proceedings of the National Academy of Sciences of the United States of America 102: 5814–5819. 10.1073/pnas.0402870102 15827123 PMC556127

[40] Langfelder P, Zhang B, Horvath S (2008) Defining clusters from a hierarchical cluster tree: the dynamic tree cut package for r. Bioinformatics 24: 719–720. 10.1093/bioinformatics/btm563 18024473

[41] Guan Y, Yao H, Zheng Z, Qiu G, Sun K (2011) Mir-125b targets bcl3 and suppresses ovarian cancer proliferation. International Journal of Cancer 128: 2274–2283. 10.1002/ijc.25575 20658525

[42] Karsy M, Arslan E, Moy F (2012) Current progress on understanding micrornas in glioblastoma multiforme. Genes & cancer 3: 3–15. 10.1177/1947601912448068 22893786 PMC3415667

[43] Kupryjańczyk J, Thor A, Beauchamp R, Merritt V, Edgerton S, et al (1993) p53 gene mutations and protein accumulation in human ovarian cancer. Proceedings of the National Academy of Sciences 90: 4961–4965. 10.1073/pnas.90.11.4961 PMC46633 8506342

[44] Kwon Y, Cukierman E, Godwin A (2011) Differential expressions of adhesive molecules and pro-teases define mechanisms of ovarian tumor cell matrix penetration/invasion. Plos one 6: e18872 10.1371/journal.pone.0018872 21526198 PMC3079735

[45] Wei N, Liu S, Chan K, Ngan H (2012) Tumour suppressive function and modulation of programmed cell death 4 (pdcd4) in ovarian cancer. PloS one 7: e30311 10.1371/journal.pone.0030311 22272332 PMC3260274

[46] Sood AK, Coffin JE, Schneider GB, Fletcher MS, DeYoung BR, et al (2004) Biological significance of focal adhesion kinase in ovarian cancer: role in migration and invasion. The American journal of pathology 165: 1087–1095. 10.1016/S0002-9440(10)63370-6 15466376 PMC1618649

[47] Devine KM, Smicun Y, Hope JM, Fishman DA (2008) S1p induced changes in epithelial ovarian cancer proteolysis, invasion, and attachment are mediated by gi and rac. Gynecologic oncology 110: 237–245. 10.1016/j.ygyno.2008.04.013 18513786 PMC3984540

[48] Bandyopadhyay S, Friedman RC, Marquez RT, Keck K, Kong B, et al (2011) Hepatitis c virus infection and hepatic stellate cell activation downregulate mir-29: mir-29 overexpression reduces hepatitis c viral abundance in culture. Journal of Infectious Diseases 203: 1753–1762. 10.1093/infdis/jir186 21606534 PMC3143452

[49] Mancini M, Saintigny G, Mahé C, Annicchiarico-Petruzzelli M, Melino G, et al (2012) Microrna-152 and-181a participate in human dermal fibroblasts senescence acting on cell adhesion and remodeling of the extra-cellular matrix. Aging (Albany NY) 4: 843 23238588 10.18632/aging.100508 PMC3560438

[50] Bar N, Dikstein R (2010) mir-22 forms a regulatory loop in pten/akt pathway and modulates signaling kinetics. PloS one 5: e10859 10.1371/journal.pone.0010859 20523723 PMC2877705

[51] Yang H, Kong W, He L, Zhao JJ, O’Donnell JD, et al (2008) Microrna expression profiling in human ovarian cancer: mir-214 induces cell survival and cisplatin resistance by targeting pten. Cancer Research 68: 425–433. 10.1158/0008-5472.CAN-07-2488 18199536

[52] Tamura M, Gu J, Danen EH, Takino T, Miyamoto S, et al (1999) Pten interactions with focal adhesion kinase and suppression of the extracellular matrix-dependent phosphatidylinositol 3-kinase/akt cell survival pathway. Journal of Biological Chemistry 274: 20693–20703. 10.1074/jbc.274.29.20693 10400703

[53] Hu Z, Lee IH, Wang X, Sheng H, Zhang L, et al (2007) Pten expression contributes to the regulation of muscle protein degradation in diabetes. Diabetes 56: 2449–2456. 10.2337/db06-1731 17623817

[54] Wen S, Stolarov J, Myers MP, Su JD, Wigler MH, et al (2001) Pten controls tumor-induced angiogenesis. Proceedings of the National Academy of Sciences 98: 4622–4627. 10.1073/pnas.081063798 PMC31884 11274365

[55] Ilić D, Almeida EA, Schlaepfer DD, Dazin P, Aizawa S, et al (1998) Extracellular matrix survival signals transduced by focal adhesion kinase suppress p53-mediated apoptosis. The Journal of cell biology 143: 547–560. 10.1083/jcb.143.2.547 9786962 PMC2132850

[56] Wang X, Wang E, Kavanagh JJ, Freedman RS (2005) Ovarian cancer, the coagulation pathway, and inflammation. Journal of translational medicine 3: 25 10.1186/1479-5876-3-25 15969748 PMC1182397

[57] Cardenas CLL, Henaoui IS, Courcot E, Roderburg C, Cauffiez C, et al (2013) mir-199a-5p is upregulated during fibrogenic response to tissue injury and mediates tgfbeta-induced lung fibroblast activation by targeting caveolin-1. PLOS Genetics 9: e1003291 10.1371/journal.pgen.1003291 23459460 PMC3573122

[58] Zhang Y, Fan KJ, Sun Q, Chen AZ, Shen WL, et al (2012) Functional screening for mirnas targeting smad4 identified mir-199a as a negative regulator of tgf-*β* signalling pathway. Nucleic Acids Research. 10.1093/nar/gks667 PMC3467063 22821565

[59] Kriegel AJ, Fang Y, Liu Y, Tian Z, Mladinov D, et al (2010) Microrna-target pairs in human renal epithelial cells treated with transforming growth factor *β*1: a novel role of mir-382. Nucleic acids research 38: 8338–8347. 10.1093/nar/gkq718 20716515 PMC3001085

[60] Denby L, Ramdas V, McBride MW, Wang J, Robinson H, et al (2011) mir-21 and mir-214 are consistently modulated during renal injury in rodent models. The American journal of pathology 179: 661–672. 10.1016/j.ajpath.2011.04.021 21704009 PMC3157202

[61] Chow JY, Cabral JA, Chang J, Carethers JM (2008) Tgf*β* modulates pten expression independently of smad signaling for growth proliferation in colon cancer cells. Cancer biology & therapy 7: 1694–1699. 10.4161/cbt.7.10.6665 18769113 PMC2820113

[62] Stegh AH, Brennan C, Mahoney JA, Forloney KL, Jenq HT, et al (2010) Glioma oncoprotein bcl2l12 inhibits the p53 tumor suppressor. Genes & development 24: 2194–2204. 10.1101/gad.1924710 20837658 PMC2947771

[63] Clark PA, Iida M, Treisman DM, Kalluri H, Ezhilan S, et al (2012) Activation of multiple erbb family receptors mediates glioblastoma cancer stem-like cell resistance to egfr-targeted inhibition. Neoplasia (New York, NY) 14: 420 22745588 10.1596/neo.12432 PMC3384429

[64] Dong Y, Han Q, Zou Y, Deng Z, Lu X, et al (2012) Long-term exposure to imatinib reduced cancer stem cell ability through induction of cell differentiation via activation of mapk signaling in glioblastoma cells. Molecular and Cellular Biochemistry: 1–14. 10.1007/s11010-012-1401-0 22829019

[65] Tsuchiya N, Izumiya M, Ogata-Kawata H, Okamoto K, Fujiwara Y, et al (2011) Tumor suppressor mir-22 determines p53-dependent cellular fate through post-transcriptional regulation of p21. Cancer research 71: 4628–4639. 10.1158/0008-5472.CAN-10-2475 21565979 PMC7425979

[66] Tarasov V, Jung P, Verdoodt B, Lodygin D, Epanchintsev A, et al (2007) Differential regulation of micrornas by p53 revealed by massively parallel sequencing: mir-34a is a p53 target that induces apoptosis and g1-arrest. Cell Cycle 6: 1586–1593. 10.4161/cc.6.13.4436 17554199

[67] Gironella M, Seux M, Xie MJ, Cano C, Tomasini R, et al (2007) Tumor protein 53-induced nuclear protein 1 expression is repressed by mir-155, and its restoration inhibits pancreatic tumor development. Proceedings of the National Academy of Sciences 104: 16170–16175. 10.1073/pnas.0703942104 17911264 PMC2042180

[68] Si M, Zhu S, Wu H, Lu Z, Wu F, et al (2006) mir-21-mediated tumor growth. Oncogene 26: 2799–2803. 10.1038/sj.onc.1210083 17072344

[69] Chen L, Zhang J, Han L, Zhang A, Zhang C, et al (2011) Downregulation of mir-221/222 sensitizes glioma cells to temozolomide by regulating apoptosis independently of p53 status. Oncology reports. 10.3892/or.2011.1535 22075712

[70] Corney DC, Flesken-Nikitin A, Godwin AK, Wang W, Nikitin AY (2007) Microrna-34b and microrna-34c are targets of p53 and cooperate in control of cell proliferation and adhesion-independent growth. Cancer research 67: 8433–8438. 10.1158/0008-5472.CAN-07-1585 17823410

[71] Yang S, Wang K, Qian C, Song Z, Pu P, et al (2012) A predicted mir-27a-mediated network identifies a signature of glioma. Oncology reports. 10.3892/or.2012.1955 22895821

[72] Lal A, Thomas MP, Altschuler G, Navarro F, O’Day E, et al (2011) Capture of microrna–bound mrnas identifies the tumor suppressor mir-34a as a regulator of growth factor signaling. PLoS Genetics 7: e1002363 10.1371/journal.pgen.1002363 22102825 PMC3213160

[73] Zhu J, Chen T, Yang L, Li Z, Wong MM, et al (2012) Regulation of microrna-155 in atherosclerotic inflammatory responses by targeting map3k10. PloS one 7: e46551 10.1371/journal.pone.0046551 23189122 PMC3506618

[74] Zhou X, Ren Y, Moore L, Mei M, You Y, et al (2010) Downregulation of mir-21 inhibits egfr pathway and suppresses the growth of human glioblastoma cells independent of pten status. Laboratory investigation 90: 144–155. 10.1038/labinvest.2009.126 20048743

[75] Cardinali B, Castellani L, Fasanaro P, Basso A, Alemà S, et al (2009) Microrna-221 and microrna-222 modulate differentiation and maturation of skeletal muscle cells. PLoS One 4: e7607 10.1371/journal.pone.0007607 19859555 PMC2762614

[76] Yan K, Gao J, Yang T, Ma Q, Qiu X, et al (2012) Microrna-34a inhibits the proliferation and metastasis of osteosarcoma cells both in vitro and in vivo. PloS one 7: e33778 10.1371/journal.pone.0033778 22457788 PMC3310405

[77] Rokah OH, Granot G, Ovcharenko A, Modai S, Pasmanik-Chor M, et al (2012) Downregulation of mir-31, mir-155, and mir-564 in chronic myeloid leukemia cells. PloS one 7: e35501 10.1371/journal.pone.0035501 22511990 PMC3325224

[78] Barker A, Giles KM, Epis MR, Zhang PM, Kalinowski F, et al (2010) Regulation of erbb receptor signalling in cancer cells by microrna. Current Opinion in Pharmacology 10: 655–661. 10.1016/j.coph.2010.08.011 20864407

[79] Xin F, Li M, Balch C, Thomson M, Fan M, et al (2009) Computational analysis of microrna profiles and their target genes suggests significant involvement in breast cancer antiestrogen resistance. Bioinformatics 25: 430–434. 10.1093/bioinformatics/btn646 19091772 PMC2642642

[80] Carro MS, Lim WK, Alvarez MJ, Bollo RJ, Zhao X, et al (2009) The transcriptional network for mesenchymal transformation of brain tumours. Nature 463: 318–325. 10.1038/nature08712 20032975 PMC4011561

[81] Zhang S, Liu CC, Li W, Shen H, Laird PW, et al (2012) Discovery of multi-dimensional modules by integrative analysis of cancer genomic data. Nucleic acids research 40: 9379–9391. 10.1093/nar/gks725 22879375 PMC3479191

[82] Ben-Moshe NB, Avraham R, Kedmi M, Zeisel A, Yitzhaky A, et al (2012) Context-specific microrna analysis: identification of functional micrornas and their mrna targets. Nucleic acids research 40: 10614–10627. 10.1093/nar/gks841 22977182 PMC3505984

[83] Ulitsky I, Maron-Katz A, Shavit S, Sagir D, Linhart C, et al (2010) Expander: from expression microarrays to networks and functions. nature protocols 5: 303–322. 10.1038/nprot.2009.230 20134430

[84] Agarwal SM, Raghav D, Singh H, Raghava G (2011) Ccdb: a curated database of genes involved in cervix cancer. Nucleic acids research 39: D975–D979. 10.1093/nar/gkq1024 21045064 PMC3013652

[85] Maqungo M, Kaur M, Kwofie SK, Radovanovic A, Schaefer U, et al (2011) Ddpc: dragon database of genes associated with prostate cancer. Nucleic acids research 39: D980–D985. 10.1093/nar/gkq849 20880996 PMC3013759

[86] Guo Y, Sheng Q, Li J, Ye F, Samuels DC, et al (2013) Large scale comparison of gene expression levels by microarrays and rnaseq using tcga data. PloS one 8: e71462 10.1371/journal.pone.0071462 23977046 PMC3748065

