# Supplementary figures and images for: A Computational Approach to Identifying Gene-microRNA Modules in Cancer
Source: PLoS Comput Biol. 2015 Jan 22;11(1):e1004042. doi: 10.1371/journal.pcbi.1004042 (PMC4303261; doi:10.1371/journal.pcbi.1004042)

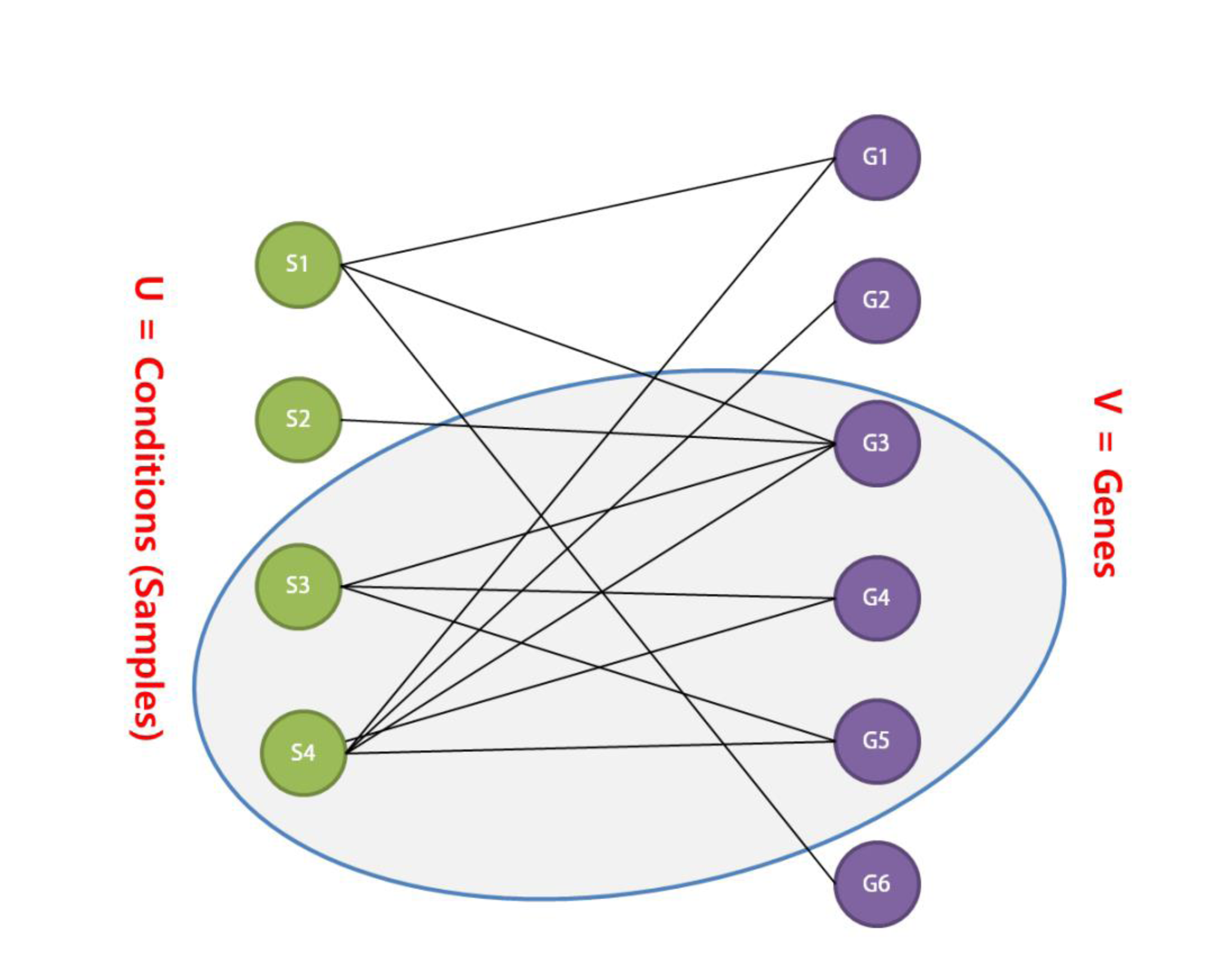

Supplement: S1 Fig — In a SAMBA biclustering algorithm, it models gene expression data into a bipartite graph G = (U,V,E). In this graph, U is a set of samples, V is a set of genes and E is a set of edges between U and V. Nodes in one side are genes and nodes in the other side are samples. An edge is linked if the expression value of gene for sample is high or low. This means that gene expression level of v significantly changes in sample of u. In this model, we try to find a subgraph G ′ = (U ′,V ′,E ′) of G, where expression values of most genes in V ′ significantly change in most of samples in U ′, representing low values or high values. For example, genes G3, G4, and G5 in V ′ and S3 and S4 samples U ′ are constructed as a module (a circle colored in grey). For gene expression data normalized by a z-score, the SAMBA biclustering algorithm generates highly correlated gene-sample clusters that represent similar tendencies of gene expression changes for a subset of samples. (TIF) [file pcbi.1004042.s001.tif]

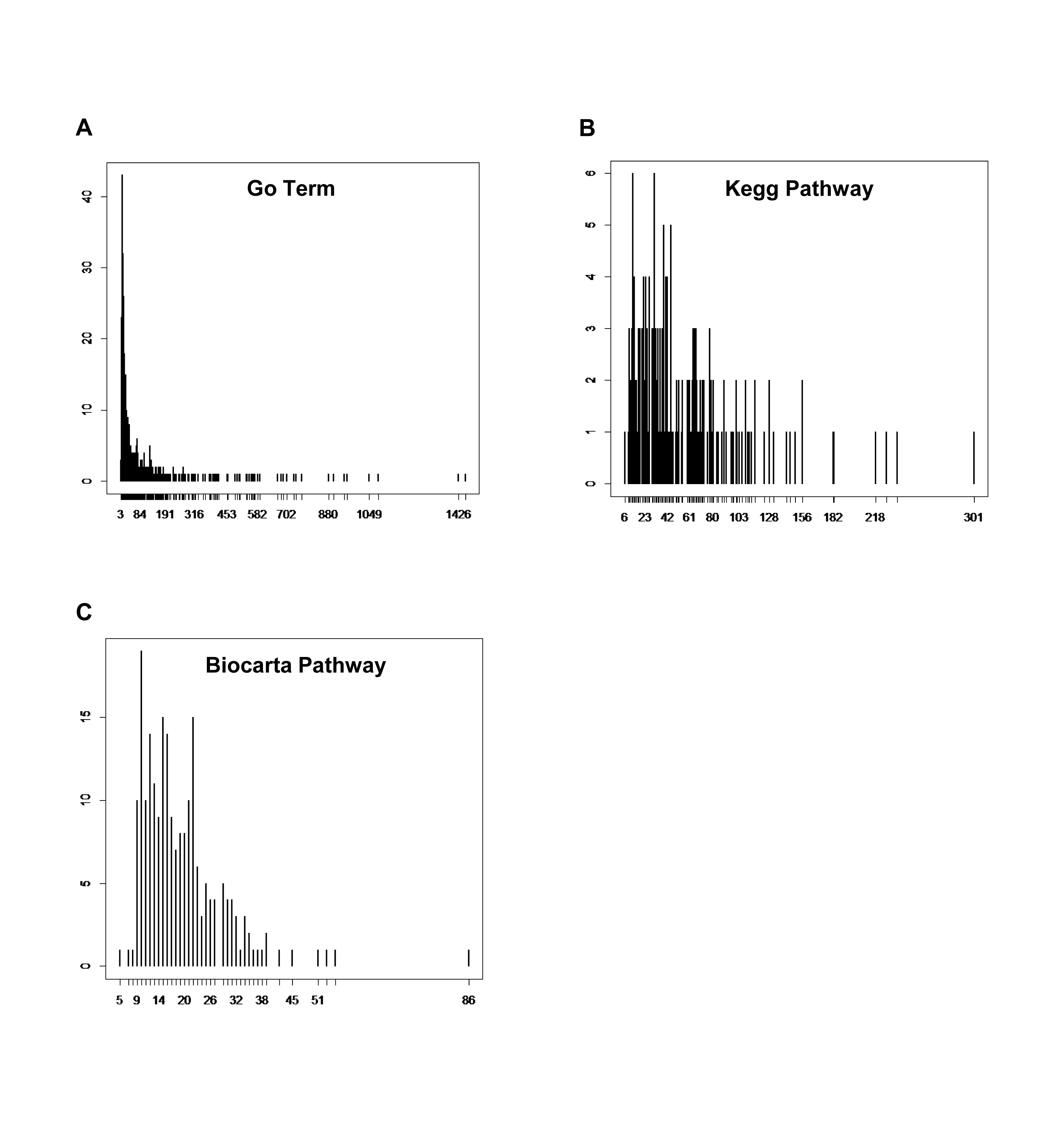

Supplement: S2 Fig — (TIF) [file pcbi.1004042.s002.tif]

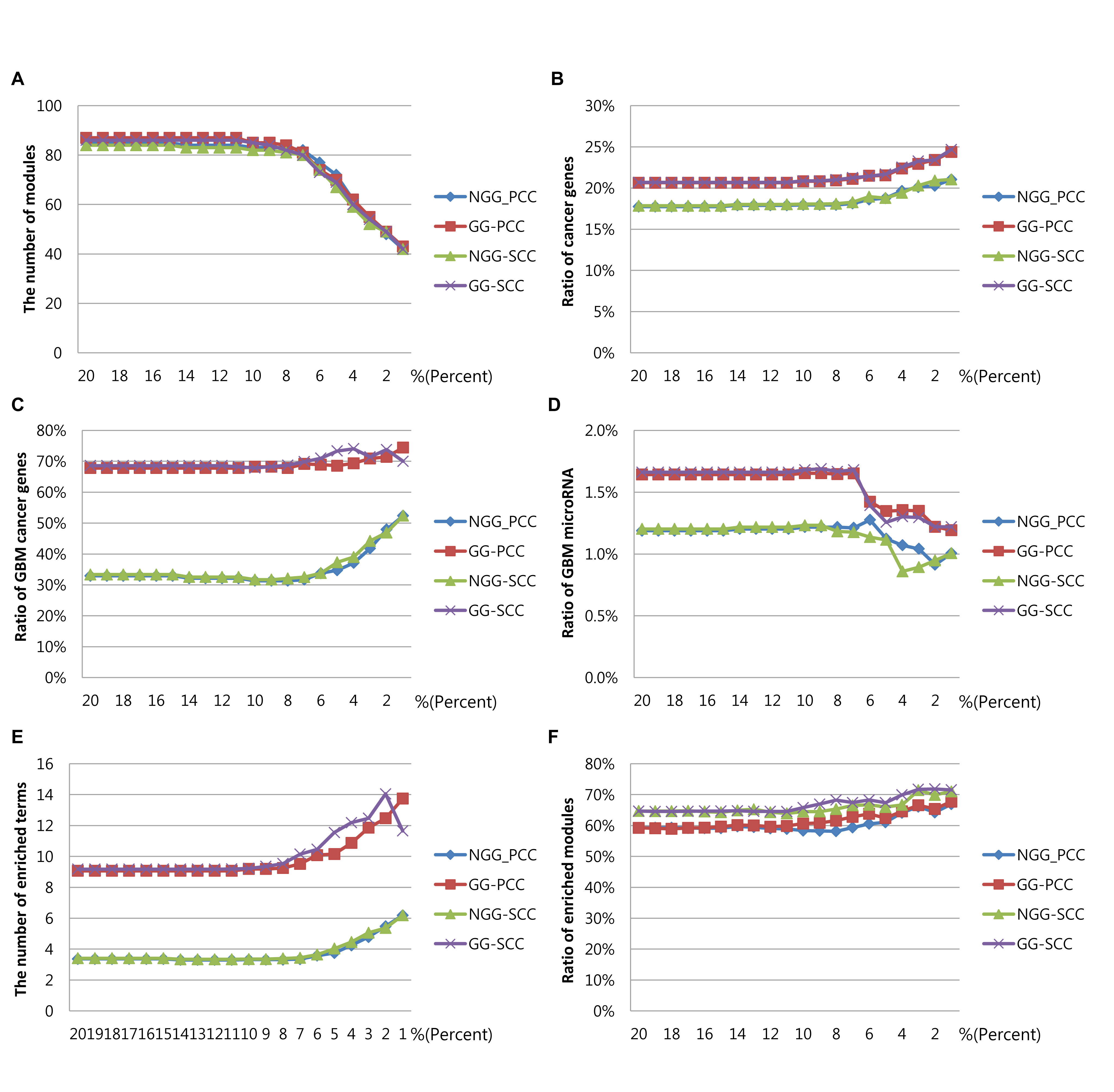

Supplement: S3 Fig — Performances of gene-miRNA modules generated from four cases (SCC with GGI information, SCC without GGI information, PCC with GGI information, and PCC without GGI information) are compared. For all cases, x-axis presents different percentages of candidate miRNAs (T%) among all miRNAs when constructing gene-miRNA modules. For each case, ratios of modules enriched with at least one pathway, the average number of enriched pathways, and ratios of cancer genes, GBM genes, and GBM miRNAs are shown. (TIF) [file pcbi.1004042.s003.tif]

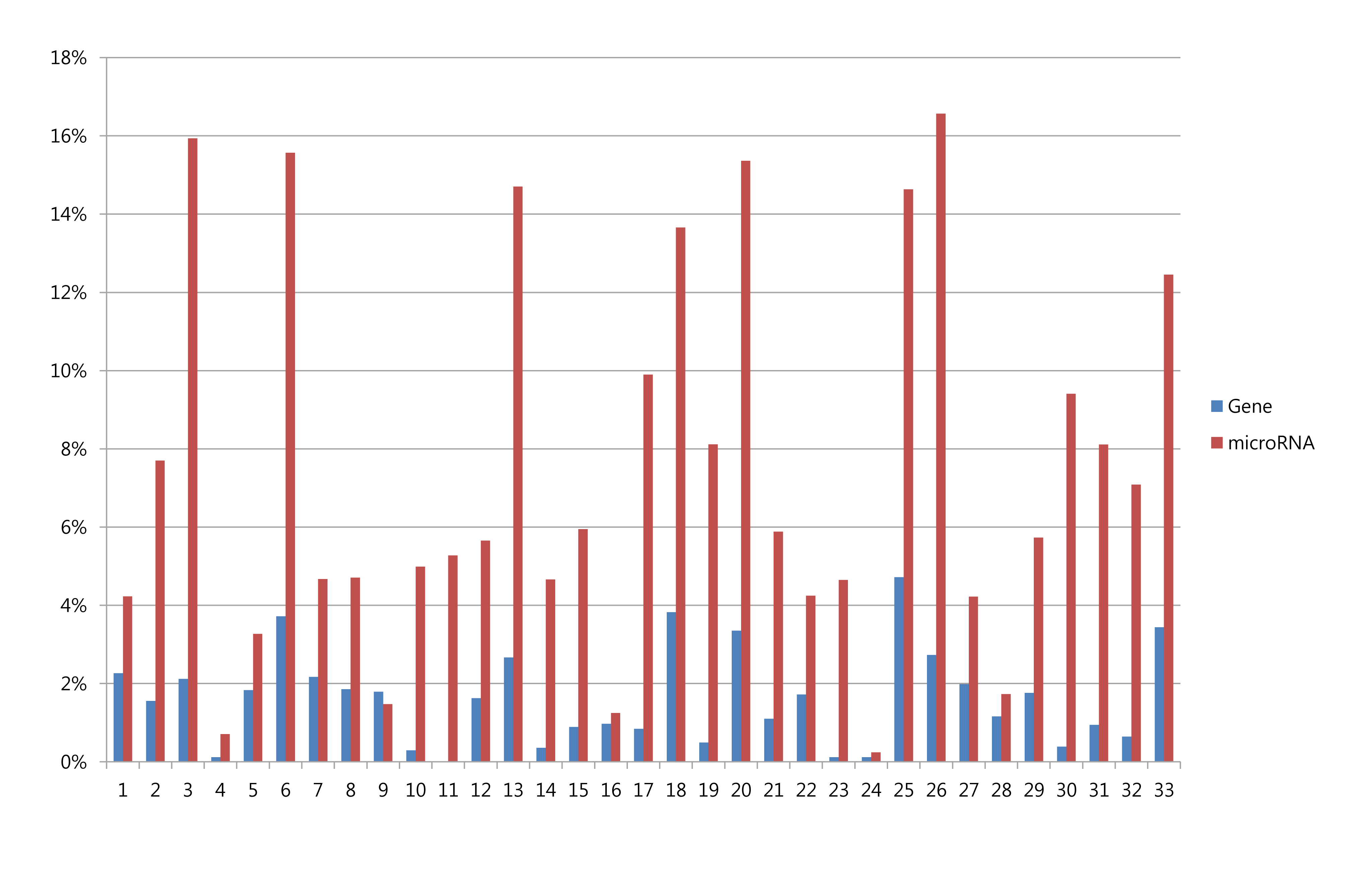

Supplement: S4 Fig — For every pairs of ovarian cancer modules, the overlap ratios of genes are defined as ∣m 1 ∩ m 2∣/∣m 1 ∪ m 2∣, where m 1 and m 2 are numbers of genes in module 1 and module 2, respectively. (TIF) [file pcbi.1004042.s004.tif]

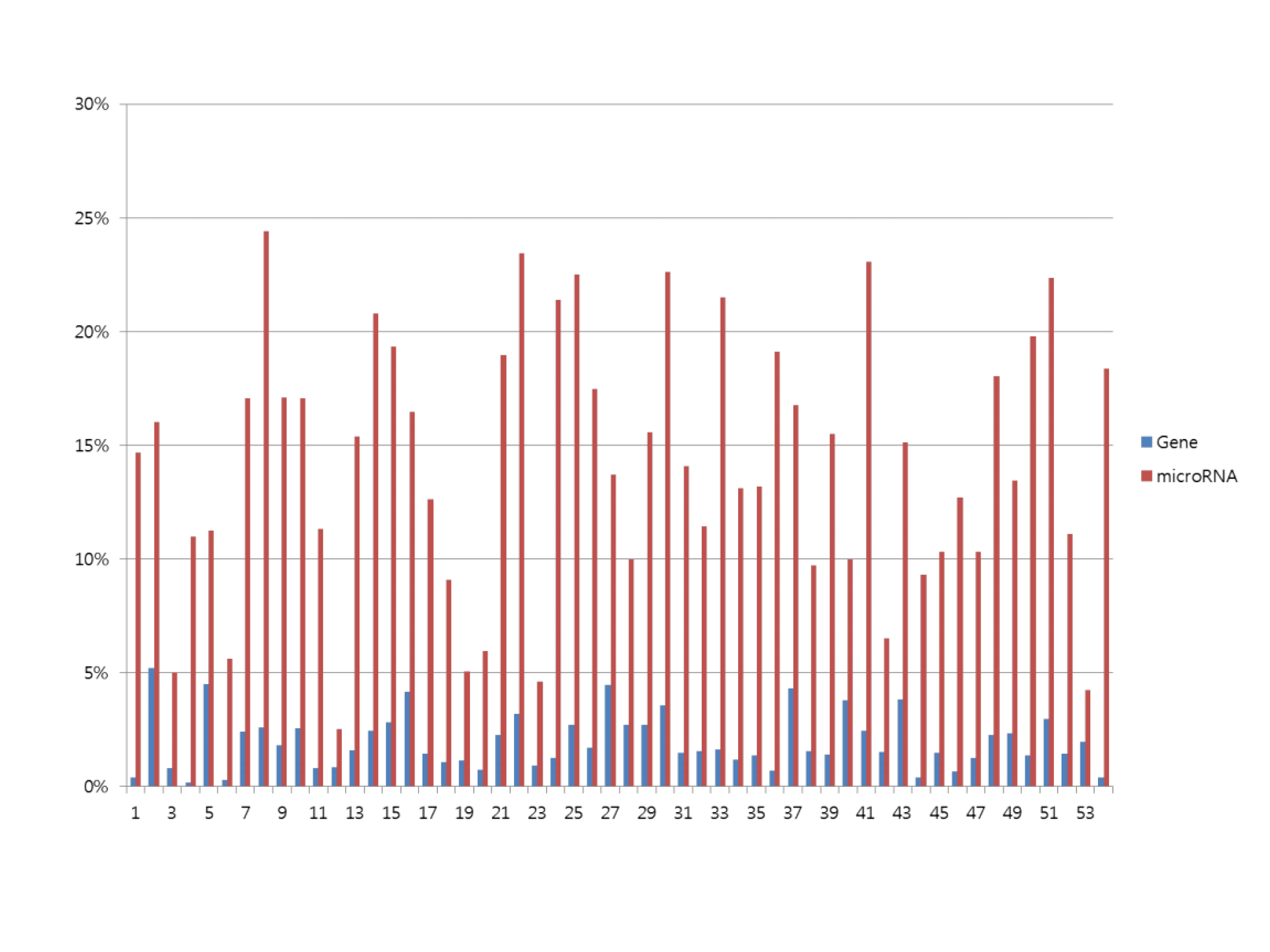

Supplement: S5 Fig — The description of the overlap ratios is the same as in Fig. S4. (TIF) [file pcbi.1004042.s005.tif]

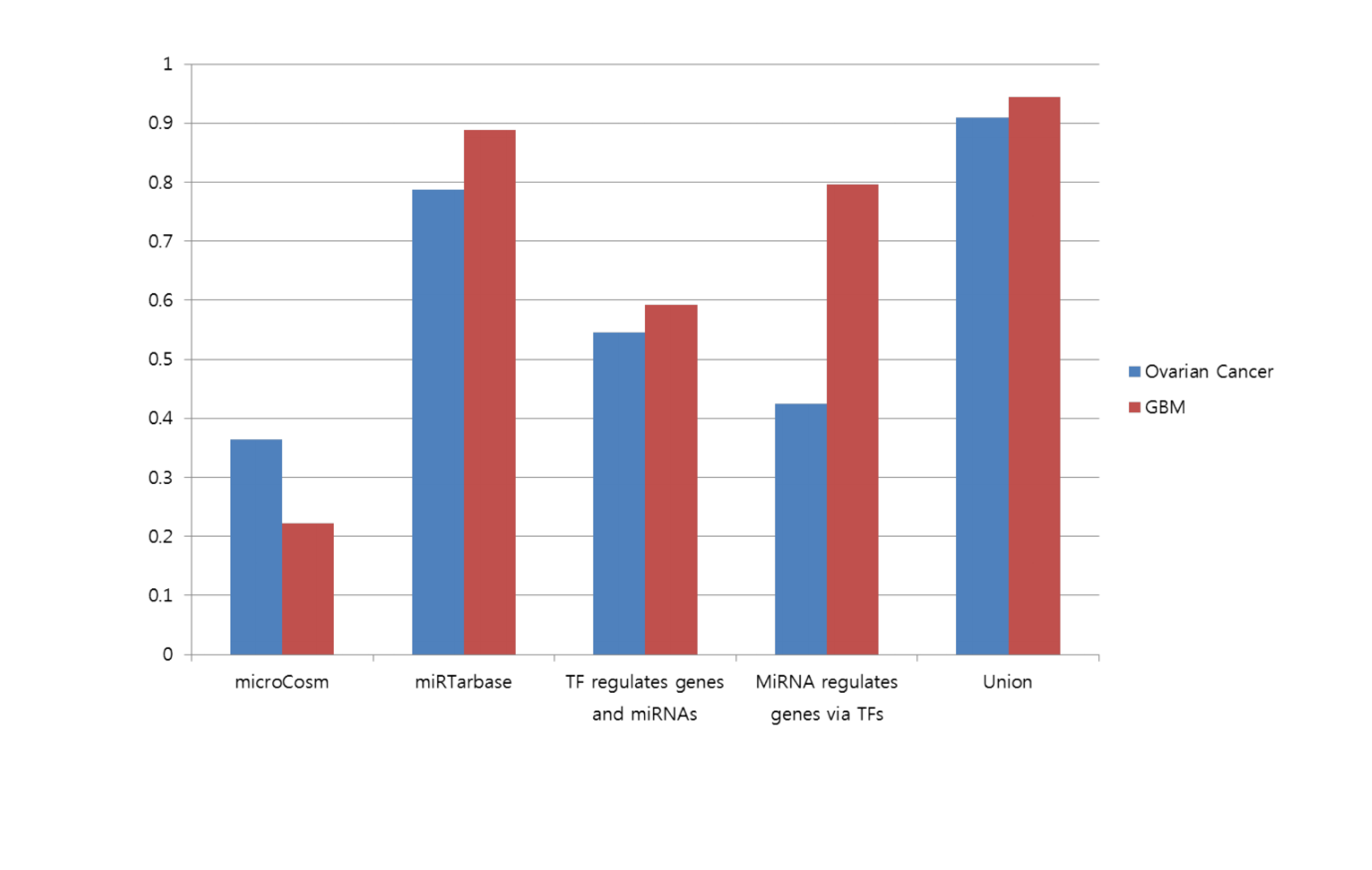

Supplement: S6 Fig — y-axis represents the fraction of modules containing at least one corresponding relationship in the modules. ‘microCosm’ represents gene-miRNA interactions based on gene-miRNA sequences from the microCosm database, and ‘miRTarbase’ represents experimentally confirmed gene-miRNA relationships from miRTarbase. ‘TF regulates genes and miRNAs’ represents that genes and miRNAs are co-regulated by the same TF. ‘MiRNA regulates genes via TFs’ represents that miRNA regulates transcription factors and transcription factors regulates genes. ‘Union’ represents all four types of relationships. (TIF) [file pcbi.1004042.s006.tif]

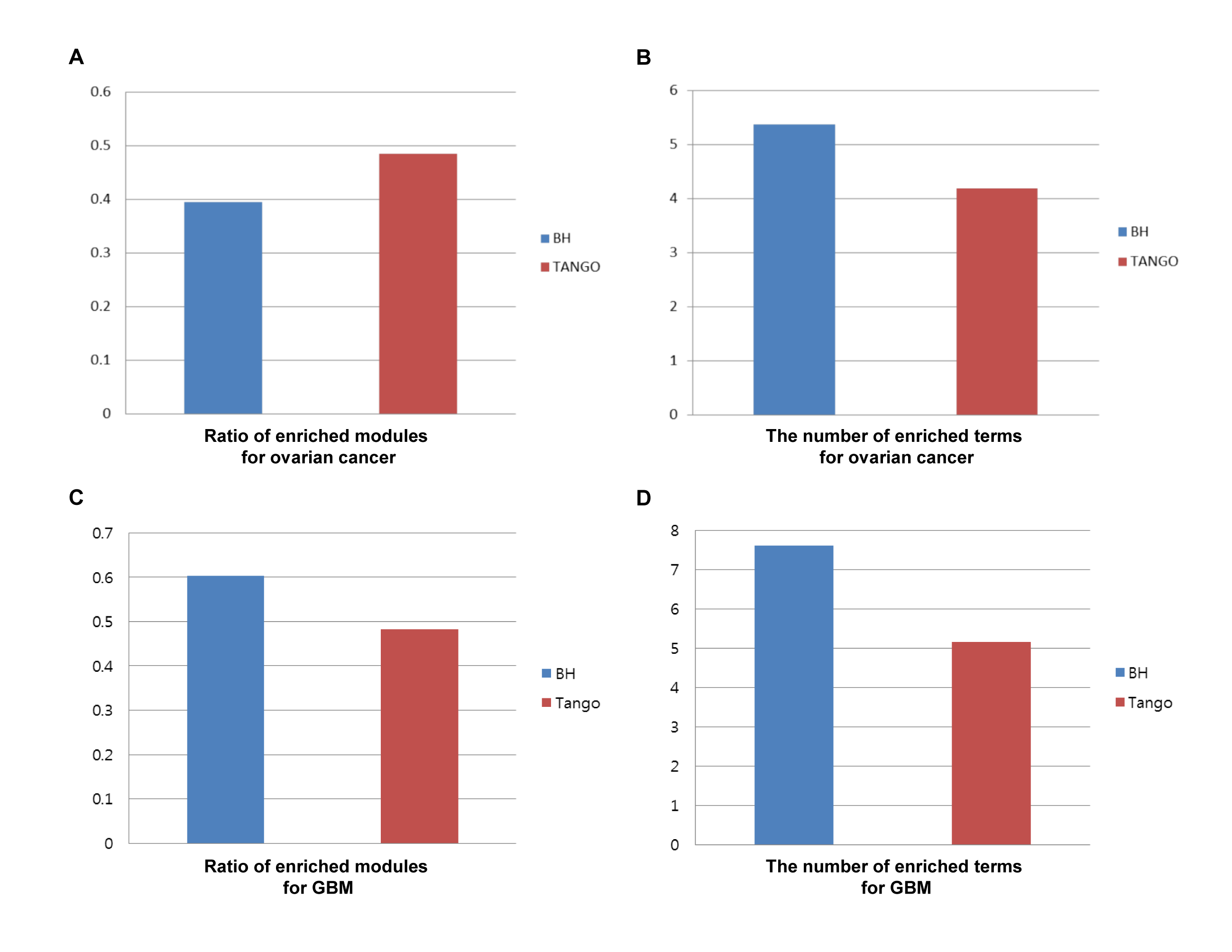

Supplement: S7 Fig — GO biological terms were tested for functional enrichment of genes in ovarian cancer and GBM modules. Two multiple comparison correction approaches, a Benjamini & Hochberg (BH) method and a TANGO tool, were used after a hypergeometric test. GO terms employed in the two approaches were not exactly same, because TANGO, which were included in an EXPANDER software, uses its own collection of GO terms, and filters out redundant terms by computing an intersection between genes in two terms. However, in both approaches, ovarian cancer and GBM modules were enriched with GO terms. (A) and (C) show the ratios of modules enriched with at least one term for ovarian cancer and for GBM, respectively, and (B) and (D) represent the average numbers of enriched terms in identifiied modules for ovarian cancer and GBM, respectively. (TIF) [file pcbi.1004042.s007.tif]
