# Supplementary material for: A Computational Approach to Identifying Gene-microRNA Modules in Cancer
Source: PLoS Comput Biol. 2015 Jan 22;11(1):e1004042. doi: 10.1371/journal.pcbi.1004042 (PMC4303261; doi:10.1371/journal.pcbi.1004042)
Supplement: S1 Table — (PDF) [file pcbi.1004042.s008.pdf]

Table S1. Genes in ovarian cancer modules.

Module ID	Genes
1	CYC1, OPLAH, ZNF250, C8orf55, GPR172A, ZNF623, BOP1, PYCRL, C8orf30A, GSDMDC1, FBXL6, ZNF7, HSF1, ZC3H3, SCRIB, DGAT1, SHARPIN, EXOSC4, GRINA, PTK2, ZNF16, LRRC14, TSTA3, ZNF696, GPAA1
2	CXCL13, ACP5, SQRDL, MMP9, LTB, CXCL11, RASSF4, EMP3, ITM2A, IL18, CD44, GBP1, TFEC, KLRB1, CXCL9, GZMH, BLNK, CTSH, FAP, FPRL2, SNX10, ICOS, IGKV1-5, EPB41L3, CD96, DPYD, SIGLEC1, CD27, POU2AF1, KCNMB1, PSCD4, VAV1, CXCL10, AIM2, CLEC5A, MEF2C, PSMB10, FLVCR2, LSP1, HCK, LCP2, DOCK2, SLA, LPXN, CCR5, ITGB2, BTK, TYROBP, SRGN, ARHGDIB, CD4, PLAUR, CASP1, CD74, PTPN22, LCK, LYN, PSMB9, FGR, SPP1
3	MCM10, CCNE2, PLK1, MCM2, GINS2, SEC61A2, GINS1, GPR19, E2F1, KIF22, LRRC20, SNX26, ARL6IP1, LOC400506, MXD3, IQCK, COQ7, TRMT11, SYT17, CDC6, TUBG1, PRC1, CDC45L, CEP55, MCM4, CEP70, POLA2, EZH2, ASPM, BUB1, TFDP2, CDK2, MCM7, DMP1, CCNB2
4	C6, TPPP3, NME5, TCTN1, LRRC23, PACRG, EFHC1, DNAI2, C9orf116, FLJ23049, LRRC48, EF-CAB1, DNAH7, DLEC1, C11orf16, C14orf45, FOXJ1, GSTA3, LRP2BP, CCDC81, DNAI1, LRRC50, DUOX1, C1orf114, EFHC2, TSGA10, CCDC19, SPEF1, ZMYND10, FLJ22167, LOC200383, DNAH3, FLJ21062, CALML4, RAB36, MAK, CASC1, SRD5A2, EFCAB6, WDR78, AGBL2, TEKT2, RTDR1, LRRC6, C11orf63, MNS1, SPAG8, TSNAXIP1
5	CYC1, VPS28, PPP1R14B, PYCRL, RANBP1, HTF9C, EXOSC4, TSTA3, UQCR, ATP5D, C1orf61, TRMT12, DFFB, MRPL40, CREBZF, DGAT1, GPAA1, C2orf18, SCRIB, BOP1, HSF1, PTK2, CPSF1, CIB1, SAT1, ZNF7, SHARPIN, NOX4, CASP7, PEX19
6	CHEK1, MKI67, TYRP1, CDC7, POLE2, CCNE2, MCM4, DONSON, TIMELESS, CDK2, KIF15, LMNB1, DSN1, MCM6, KNTC1, RFC5, GPSM2, BARD1, CDC25A, PRIM1, FEN1, MCOLN3, KIF4A, CBX5, MCM2, CEP55, MSH6, PCNA, KIF23, PARP2, BUB1, RACGAP1, CDKN3, MAPK8IP3
7	GPR172A, BOP1, PYCRL, C8orf30A, GSDMDC1, FBXL6, RECQL4, DGAT1, SHARPIN, RAD21, TSTA3, NCKAP1, STIP1, PRKCE, GPAA1, CHORDC1, TTC35, ZNF7, HSF1, CPSF1, SCRIB, ZNF250, FLT4, PLEC1, PTK2
8	C1orf116, MMP11, BGN, IRX5, INHBA, ACTA2, EPYC, MYLK, CCL21, MMP19, HNT, SERPINE1, RBPMS, CEND1, POU2F3, COMP, TUBA4A, ITGB1, PDLIM3, COL16A1, AQP1, FAP, PLAU, THBS2, CTSK, THBS1, VCAN, TGFB1I1, COL3A1, COL1A1, FN1, TIMP3, CALB2, PDGFRB, COL1A2, LGALS1, ITGA5, RECK, PLAUR
9	CDCA8, C1orf109, AK2, C1orf108, SNIP1, GNL2, RLF, EIF2C3, TRIT1, YRDC, RRAGC, AGTPBP1, PPIE, PSMB2, ACVR2A, MED8, COL9A2, CACNA1E
10	NCAPD2, MLF2, FOXM1, NOL1, CLSTN3, ATN1, ITFG2, LRP6, RAD52, USP5, FAM90A1, PEX5, CD74
11	THOC6, KREMEN2, E4F1, CLUAP1, TELO2, IFT140, CLDN6, C16orf59, TBX2, NTHL1, SHFM1, UBE2I, DNAJA3
12	PAK1IP1, PECI, E2F3, CCDC90A, RPP40, FAM8A1, DEK, GNL3, CDYL, EEF1E1, BPHL, NUP153, KC-NMB4, JARID2, GMNN, FXR1, PALB2, PRIM1, SNRPE, EXOSC5, LSM2, RFC5, FEN1, NBN, MCM2, RACGAP1, PSMA5, STAG2, TFDP2, CDC5L, LSM3, GNAI3, MCM10
13	BUB1, MCM10, DEPDC1, CCNE2, NCAPH, SNTB1, CDC6, ACOT7, CDC45L, FAM29A, EXO1, UCHL5, NUP155, BXDC2, SENP5, APOBEC3A, RIF1, ARNTL2, HEATR3, RAD1, RANBP1, WDR8, PEX10, NADK, AURKAIP1, CDC42, POLE2, FANCE, GINS2, DOM3Z, CDCA8, TFDP2, KIF4A, HDAC1, MCM7, SMARCB1, POLL, DDX49, SCNM1, ORC2L, GAD1, PKN1, FANCG, PLK1, BIRC5, RRAGB, DBF4, LSM5, MPZL1, PCNA, TMPO, CHEK1, RBBP9, MCM6, SUB1, CEBPG, DSN1, ORC5L, ZNF165, EIF4EBP1, AURKB, TIMELESS, SMC2, RPP21, ARL6IP1, CBX3, MELK, SMC4, PSMB4, TFAM, ORC1L, EXOSC10, RPP30, ECT2, PDCD5, GMNN, BUB3, C1orf156
14	TMEFF1, SOX12, CAMTA1, DBN1, RP4-691N24.1, PTBP2, CEP170, C1orf114, THNSL1, SOX11, TUBB4, SPAST, FNBP1L, POLR3F, PLCG1, BCORL1, LCN2, POU2F2, BCL3, HK2, OASL, RFXANK, MAP3K4, PIGT, MAPK7,
15	EFNA4, COL2A1, TAF1A, GPATCH2, ZNF692, SPAG6, VASH2, DNAH7, C1orf114, SOX11, PRR3, COLEC11, COL9A3, LAMA2, C6orf134, DDX25, NFYA, RHOBTB1, LSM7, NDRG1, COPS3, BNIP1, TUBB, BCL3, LSM2, DOM3Z, COL4A6, SP100, INSR, MXD3, SCRIB, CSNK2B, MIS12, TSN, PFDN6
16	STX10, ILVBL, MYO9B, CHERP, SLC35E1, TRMT1, EIF3G, FARSA, GADD45GIP1, ZNF136, GTPBP3, WIZ, GIPC1, C19orf62, LOC90379, PPAN, EPS15L1, PKN1, C19orf58, GCDH, FAM32A, TMEM161A, AKAP8, DDX49, CDC37
17	TIMELESS, TMEM118, PHOX2A, DYRK2, KIAA0286, SHMT2, TDG, NAB2, XPOT, C12orf41, FAM130A1, YEATS4, ZBTB39, MARS, LMBR1L, NUP107, CAND1, PRKRIR, RRAGC, PPFIBP1, C11orf30, FAF1, CTSH, PPIE, MSH2, NFYC, NCAPD2, RLF, MYBL2

18	CHEK1, CCNE2, MCM4, DONSON, WHSC1, DSN1, MCM6, KNTC1, RFC5, CASP8AP2, RNGTT, SLBP, MCM3, DEK, DMC1, NASP, MCM2, TIMELESS, FEN1, MKI67, SMC4, KIF23, TOPBP1, EXO1, RACGAP1, MELK, MCM7, FANCG, CDC7, FOXM1, AURKB
19	C1orf116, ZDHHC18, VGLL1, ALS2CL, SMARCA2, ETV6, KIF13B, KRT7, CFHR2, ADAM28, RHBDF2, PRKCD, VPS39, LCN2, LMNA, GORASP1, MAPKAPK2, RAD54L2, MAP3K3, TFEB, IFNAR2, CD82, PML, CAPN1, CTSB
20	BUB1, GTSE1, SPC25, CDCA3, DEPDC1, PLK1, CEP55, CENPE, KIF14, POLQ, ASF1B, FOXM1, NEIL3, ERCC6L, EXO1, CCNA2, RAD51, E2F8, MAD2L1, SPAG5, AURKB, NEK2, CCNB1, EXOSC9, PLK4, HMGB2, KIF4A, BIRC5, NCAPH, MELK, TOP2A, BUB1B, MYBL2, CCNB2, KIF23, CDCA8, KIF15, CDC20, RACGAP1, ASPM, RAD51AP1, GINS2, CENPA, AURKA
21	CXCL13, CXCL11, ITM2A, TFEC, KLRB1, CXCL9, GZMH, FPRL2, ICOS, CD96, CD27, AIM2, CCR5, IL2RG, GZMA, IL2RB, GZMB, LCP2, PTPRCAP, ITK, LCK, MNDA, SLA, BTK, CCR1, LPXN, LAPTM5, DOCK2, HCK, C1QA
22	GREM1, MMP11, MMP1, BCAT1, LOXL2, TDO2, ADAM12, CCRL1, MMP19, COL5A3, COL1A1, FAP, DPT, CTSK, ECM1, VCAN, PLAU, COL6A1, COL5A1, INHBA, THBS2, SPARC, FN1, ITGA5, COL3A1, AEBP1, MMP2
23	RBBP5, TAF1A, TBCE, KIAA0133, TSNAX, RRP15, GNPAT, FBXO28, SNRPE, CAMSAP1L1, ARID4B, CXXC4, RAB4A, C17orf48, PARP1, NUP133, NSL1, KRI1, H3F3A
24	SPARCL1, DLK1, STAR, C4orf31, ABCA8, SFRP1, GPRASP1, OGN, COLEC11, PID1, PCDH21, LAMA2, SNED1, GSTM5, RASL12, TCF21, GATA4, ENG, PDGFRA
25	CHEK1, FBXO5, CDC7, POLE2, CCNE2, MCM2, KIAA0101, MCM6, PCNA, TRMT11, TIPIN, MELK, FEN1, MCM7, DSN1, CCNB2, KIF15, SMC4, KIF4A, MCM10, KPNA2, MYBL2, TIMELESS, MCM4, PRC1, RACGAP1, KIF23, EXO1, NDC80
26	CENPA, MCM10, FBXO5, DEPDC1, PLK1, FAM64A, CENPN, BIRC5, CCNA2, MAD2L1, SFT2D2, H2AFZ, RAP1GDS1, C16orf61, DYNLT1, HIST1H3G, PLK4, STK17B, NSUN3, FEN1, PCNA, TIMELESS, EXOSC9, GINS2, CCNB1, KPNA2, MCM4, SPC25, RIT1, CHEK1, CSTF1, RPA3, UCHL5, XRCC4, GMNN, SLC25A11, HMMR, RAN, PSMA7, CDC7, RFC5, RANBP1, DSN1, RFC4
27	GREM1, MMP11, COL11A1, C7orf10, BGN, LOXL2, TMEM158, INHBA, TWIST1, ACTA2, COL5A2, PLAU, EPYC, ADAM12, SLC12A8, ACTG2, DCN, SRPX2, TNFAIP6, MMP19, MMP14, HNT, PMP22, COL1A1, VCAN, C1QTNF3, SFRP4, SERPINF1, LAMB1, COMP, FAP, C10orf56, PDLIM3, ECM2, COL16A1, LOX, FSTL1, COL5A1, COL4A1, TAGLN, LHFP, XYLT1, FBN1, THBS2, SPARC, CTSK, ECM1, AEBP1, COL3A1, fMMP2, PDGFRB, THBS1, FN1, COL1A2, TGFB1I1
28	MYO9B, CHERP, COPS7B, TRMT1, EIF3G, FARSA, AKAP8L, ZNF136, WIZ, FABP6, ARMC6, PPAN, DDX49, SFRS14, ZNF426, TMEM161A, MRPL4, ZNF14, AKAP8, CDC37, GTPBP3, RFXANK, POLE, UPF1, ILF3, HMGA1, JARID2, GREM1, PRKRIP1, LGALS1, FAS, DDX27, NRF1, SLC35E1, SMARCA4, DNMT1, SMPD4
29	MYCL1, CDCA8, C1orf109, C1orf108, YBX1, MED8, MRPS15, ELOVL1, SNIP1, GNL2, DPH2, CTPS, PPIE, RLF, EIF2C3, JMJD2A, TRIT1, RRAGC, TRAPPC3, PPIH, MYCBP, PSMB2, YRDC
30	GTF2H4, MRS2L, CENPQ, TAF11, PAK1IP1, LSM2, MED20, WDR46, ZKSCAN4, ZSCAN16, SNRPC, EEF1E1, ABT1, RNF5, RDBP, RGL2, TBCC, RXRB, CDC5L, PBX2, CSNK2B, DAXX, EHMT2, SKIV2L, ZBTB22, PFDN6
31	PHF11, SQRDL, SLC15A3, TRIM22, STAT1, RAB25, SMARCA2, OAS2, SERPINF1, RARRES3, HERC5, STAT3, DPYD, HCCS, PYCARD, CTSO, TPST2, STOM, BAZ1A, OAS3, OAS1, CXCL10, DBI, AIM1, PSMB9, CASP1, ISG15, CTSD, SIGLEC1, IRF1, OASL, IRF9, GBP1, CXCL11, WASF1, SAMSN1, SPP1, PTAFR, SP100, EPB41L3, KLF12, CTSB, TNFSF10, IRF2, MAPK8IP1, ACVR2B, PSMB10, CD82, USP18, CTSL1, TACC1, LAPTM5, C1QA, LCP2, S100A13, CAPG, RFXAP, ITGB2, LYN, CCL5, CXCL9, PSME2, RAB27A, SLA, EYA2
32	C1orf116, TMPRSS4, ETV4, S100A2, PPAP2C, TRIM29, ANXA1, REEP5, KRT7, S100A14, S100A4, PLLP, RAB11FIP1, S100P, KIF15, CAPN1, RAB25, SFN, TOB1, S100A11, ITGB4, CASP7, PLEKHF2, CD82, PRKCH
33	BUB1, CENPA, SPC25, NCAPG, MCM10, FBXO5, MKI67, TROAP, KIFC1, TTK, DKFZp762E1312, ESPL1, ASPM, DEPDC1, PLK1, CEP55, KIF14, NDC80, FAM64A, AURKA, RACGAP1, KIF11, RAD51AP1, FOXM1, KIF23, OIP5, SMC4, FANCI, ECT2, KIF20A, PRC1, TPX2, BUB1B, KIF15, UBE2C, TK1, CENPF, MELK, NUSAP1, EXO1, TOP2A, CCNA2, DLG7, CDC20, SPAG5, PBK, AURKB, TACC3, RAD54L, RRM2, CKS2, KIF4A, CCNB2, NCAPH, CDCA8, BIRC5, KPNA2
