# Supplementary material for: A Computational Approach to Identifying Gene-microRNA Modules in Cancer
Source: PLoS Comput Biol. 2015 Jan 22;11(1):e1004042. doi: 10.1371/journal.pcbi.1004042 (PMC4303261; doi:10.1371/journal.pcbi.1004042)
Supplement: S2 Table — (PDF) [file pcbi.1004042.s009.pdf]

Table S2. MiRNAs in ovarian cancer modules.

Module ID	microRNAs
1	miR-30d, miR-151, miR-30b, miR-221, miR-23b, miR-331, miR-222, miR-9, miR-24, miR-378, miR-338
2	miR-142, miR-150, miR-146a, miR-155, miR-223, miR-452, miR-185, miR-22, miR-21, miR-224, miR-218, miR-140, miR-342, miR-147b, miR-335, miR-20a, miR-514, miR-125b, miR-96, miR-507, miR-29b, miR-29c, miR-506, miR-17
3	miR-18b, miR-29a, miR-18a, miR-93, miR-768, miR-25, miR-15b, miR-7, miR-106b, miR-301a, miR-19a, miR-29b, miR-130b, miR-301b,
4	miR-34c, miR-449a, miR-449b, miR-34b, miR-508, miR-509
5	miR-30d, miR-151, miR-30b, miR-221
6	miR-29a, miR-18b, miR-768, miR-18a, miR-93, miR-29b, miR-15b, miR-7, miR-25, miR-301a, miR-29c, miR-101, miR-19a, miR-20b, miR-130b, miR-106b, miR-26a, miR-17, miR-301b, miR-934
7	miR-30d, miR-30b, miR-151, miR-221, miR-23b, miR-331, miR-222
8	miR-22, miR-152, miR-214, miR-409, miR-199a, miR-382, miR-199b, miR-508
9	miR-30e, miR-324
10	miR-200c, miR-141, let-7b, miR-142, miR-21, miR-452, miR-224, miR-146a, miR-106b
11	miR-130a, miR-22, let-7b
12	miR-18b, miR-93
13	miR-18b, miR-18a, miR-93, miR-768, miR-29a, miR-7, miR-301a, miR-25
14	miR-29b, miR-149, miR-218, miR-142, miR-130a, miR-125b
15	let-7b, miR-9, miR-142, miR-20a, miR-29b
16	miR-27a, miR-23a, miR-181d, miR-24, miR-629
17	miR-29a, let-7b, miR-93, miR-29b, miR-148b, miR-301a, miR-301b
18	miR-18b, miR-29a, miR-768, miR-93, miR-18a, miR-15b, miR-25
19	miR-222, let-7b, miR-20a, miR-218, miR-17, miR-934, miR-301a, miR-130a
20	miR-18b, miR-18a, miR-29a, miR-768, miR-15b, miR-19a, miR-7, miR-93, miR-29b, miR-26a, miR-17, miR-301a, miR-101
21	miR-142, miR-150, miR-146a, miR-155, miR-452, miR-223, miR-185, miR-224, miR-22, miR-21, miR-140, miR-342, miR-218, miR-514, miR-507, miR-506, miR-96
22	miR-22, miR-152, miR-214, miR-409, miR-508, miR-514
23	miR-17, let-7b
24	miR-154, miR-376c, miR-376a, miR-377, miR-299, miR-381, miR-495, miR-127, miR-337, miR-411, miR-410, miR-376b, miR-543, miR-432, miR-145, miR-136
25	miR-18b, miR-29a, miR-18a, miR-768, miR-93, miR-15b, miR-25, miR-7
26	miR-18b, miR-18a, miR-29a, miR-768, miR-7, miR-93, miR-26a, miR-19a, miR-301a, miR-564, miR-29b, miR-15b, miR-101, miR-25, miR-139, let-7b, miR-17
27	miR-22, miR-214, miR-152, miR-409, miR-199a, miR-382, miR-199b, miR-127, miR-431, miR-379, miR-508, miR-145
28	miR-452, miR-27a, miR-23a
29	miR-30e, miR-30c, miR-324, miR-93, miR-768
30	miR-219, let-7b, miR-106b, miR-107, miR-93, miR-301a, miR-25, miR-324, miR-564, miR-18b, miR-139, miR-20b, miR-301b
31	miR-142, miR-150, miR-146a, miR-155, miR-218, miR-452, miR-185, miR-224, miR-22, miR-335, miR-20a, miR-29c, miR-21, miR-125b, miR-223, miR-17, miR-222, miR-140, miR-29b, miR-149, miR-130a, miR-20b, miR-183
32	miR-222, miR-25, let-7b, miR-934, miR-221, miR-218
33	miR-18b, miR-18a, miR-29a, miR-768, miR-15b, miR-7, miR-19a, miR-93, miR-29b, miR-26a, miR-101, miR-301a, miR-17, miR-16, miR-25, miR-130b, miR-29c, miR-20b, miR-564, miR-628, miR-425, miR-106b, miR-139, miR-301b, miR-934, miR-512, miR-455, let-7b, miR-663, miR-146b, miR-548c
