# Supplementary material for: A Computational Approach to Identifying Gene-microRNA Modules in Cancer
Source: PLoS Comput Biol. 2015 Jan 22;11(1):e1004042. doi: 10.1371/journal.pcbi.1004042 (PMC4303261; doi:10.1371/journal.pcbi.1004042)
Supplement: S3 Table — (PDF) [file pcbi.1004042.s010.pdf]

Table S3. Genes in GBM modules.

Module ID	Genes
1	ZNF22, SEC61A2, MCM10, RSU1, CORO1C, TMPO, C10orf18, IDI1, GPSM2, WAC, DCLRE1C, ATP5C1, ZMYND11, KIN, ARHGAP12, SEPHS1, RAP1GDS1, PITRM1, ACTR1A, PDSS1, PPP2R2D, RPP38, BUB3, EEF1G, HSF2, PPP1CC, PPP2R5D, KRAS, DDX21, PHB2, TFAM, DEDD, NBN
2	MYBL1, NDC80, GTSE1, NCAPG, CENPE, STIL, MKI67, CENPF, DKFZp762E1312, KIF2C, TTK, FANCI, ASF1B, TOP2A, ESPL1, ASPM, KIF15, CDC45L, KIF11, FOXM1, NCAPH, KIF20A, AURKA, RACGAP1, BUB1, CDCA3, AP3B2, MCM10, FBXO5, KIF23, CENPM, KIF4A, SPAG5, MXD3, RAD51, KNTC1, CDT1, CDCA8, PTTG3, CDC7, POLQ, EXO1, MSH5, LMNB1, TROAP, KIF14, ORC6L, DDX11, RAD54L, E2F8, ZNF248, WDHD1, CUTL2, FGF9, PAK7, CCDC15, CELSR3, TRAIP, BCOR, BUB1B, KPNA2, PLK1, TACC3, ZWINT, CHEK1, AURKB, TPX2, EZH2, CCNA2, BRCA1, MCM2, HMGB2, MCM4, POLA2, ATAD2, PRC1, NEK2, CDC6, MELK, CDC25C, MCM3, TUBG1, CEP55, RAD51AP1, SPC25, ECT2, SMC2, BUB3, MYBL2, MCM7, CDC20, SNRPA1, MAD2L1, BIRC5, MCM6, GOT1, ZWILCH, SFRS2
3	LIG1, SPAG1, SFRS12, C6orf26, NASP, ZNF292, CREBZF, FAM111A, CCDC93, GATAD1, NFATC3, KNTC1, MSH5, SETD6, CCDC41, ZNF83, MNS1, C16orf53, SMPD4, PRPF3, CCNL2, C21orf66, FLJ10213, ULK1, PARP6, PHKA2, LRRC48, RCOR3, NPFF, SRRM1, CDK5RAP3, C9orf39, ARHGEF4, KIAA1009, RBBP6, NFATC2IP, DST, SEMA4C, SPG7, EZH1, PPIG, TCERG1, GTPBP3, RAD9A, POLR1B, ZNF44, AKAP9, INSR, VPS39, TYK2, ZNF451, PRPF4B, CHFR, CEP70, DVL3, ATM, TERF1, SPEN, SENP6, TOP2B, XRCC2, PIK3R1, MDC1, ZNF337, SIN3B, XPO6, TSC2, SFRS18, NCOR1, CTBP1, PNN, DVL2, CLK1, ATR, C11orf61, BCL6, CYLD, OFD1, CLK2, PRKAR2A, TOPBP1, XPO1, MAPKBP1, PLD2, SCAPER, GSK3B, TRA2A, ZNF426, CUTC, DFFB, ITGA2, DCP1A, AMOTL2, PCNT, DCLRE1C, ZHX2, DHX30, ATXN1, SFRS2IP, PAXIP1, CHD4
4	SP110, IFI44L, TRIM6-TRIM34, OAS3, CXCL11, SIGLEC1, SP100, IFI35, GBP1, NMI, OAS1, HERC5, IRF7, FLJ20035, IFIT3, IFI44, IFIH1, STAT1, USP18, TAP1, UBE1L, RTP4, BTN3A1, IRF9, BTN3A3, OAS2, SAMD9, TLR3, APOL6, TRIM21, PARP12, CXCL10, PSME2, APOL3, EIF2AK2, RSAD2, XAF1, MX2, HCP5, MX1, TNFSF10
5	GTSE1, CENPE, DKFZp762E1312, LIG1, CDC45L, CHEK1, NASP, MCM7, FANCC, TIMELESS, CHAF1A, SPAG5, RAD51, EXO1, TRIM28, ZNF235, ZNF551, XRCC2, MYBL2, DPF1, LMNB1, FANCI, KIF2C, NCAPH, KIF14, TPX2, CENPF, KIF15, ASPM, CDK2, MCM2, MCM4, KIF11, NDC80, TMPO, FOXM1, MCM3, PRC1, MKI67, KIF4A, KNTC1, TTK, TACC3, TROAP, PLK1, CDCA3, TOP2A, NCAPG, ZWILCH, FBXO5, AURKB, EZH2, SNRPA1, BUB1B, RAD51AP1, KIF23
6	RAB40B, GRM3, TF, FBXO2, QDPR, SH3GL3, MAL, S100A1, MBP, CENTA1, NPY, MOBP, RAPGEF5, ENPP2, SPOCK3, 04-Sep, DBNDD2, MAG, LHPP, PIP4K2A, LDB3, SEC14L5, PLP1
7	OIP5, FAM64A, VAX2, GINS2, SPC25, CENPM, MXD3, NUDT1, AURKB, TRAF4, KCNQ2, DTYMK, FANCE, TRAIP, CD320, CECR5, H2AFX, ISG20L1, BCL7C, RAD51AP1, LSM4, BIRC5, SFRS3, MAD2L1, CEP72, DBF4, SNRPD1, CENPA, CCNB1, AURKA, GMNN, MOBKL3, PCNA, CDCA3, PPP1CC, ORC4L, SFRS2, BUB1B, RACGAP1, RAD54B, STIL, NCAPH, NDC80
8	STEAP3, PLA2G2A, TREM1, PTX3, SOD2, CXCL5, G0S2, UBD, PDLIM3, SLC39A8, SYNPO, IL32, STC1, IL1R2, CCL20, CHI3L2, PBEF1, CD163, STAB1, RNASE2, SLC11A1, ADFP, BIRC3, SLC39A14, IL10RA, SPSB1, TCIRG1, CP, SIGLEC9, EMR1, THBD, FBLN5, CPD, RGS16, ICAM1, HP, HSPA6, TNFRSF1B, HCK, FOSL2, CCR5, IL1B, CD14, IL4R, NCF1, PLAUR, PTPN6, SERPINA1, FCGR2B, TLR1, WAS, CEBPB, SLA, CTSB, JUNB, SPI1, MMP7, SAT1, SYK, BTK, MAP3K8, PLCG2, CCR1, CCL2, ITGAM, FAS, LYN, SIGLEC7, PTPN2, PTPRC, CD44, FCGR2A, IL13RA1
9	BASP1, STMN2, OPCML, INA, DYNC1I1, SOX11, INSM1, TUBB4, STMN4, HMP19, PAK3, DNM3, LRRTM2, VAX2, C1orf106, SOX4, WASF1, RAP2A, PPP1R16B, PGRMC1, RIMS2, DUSP26, SATB1, PEG3, SH3GL3, TMSL8, C20orf42, C1QL1, ACAN, MBP, ELMO1, TMEM16C, PROM1, RAB33A, GSTA4, ST8SIA3, SOX10, ALG6, HSPB3, DGKB, SLC1A1, HDAC2, PAK7, TTC3, CA10, SCN3A, MAG, GNAI1, NRXN2, FHOD3, OLIG2, PLCB1, SH3BP4, NCAM1, KIF21B, ICK, FLRT1, FGF12, MMP17, ERBB3, TRIP6, RBPJ, AMOTL2, CRMP1, DPYSL4, G3BP2, SNAP91, TSPYL4, MAP3K4, CDK5R1
10	MKI67, GART, NONO, CHEK1, HNRPF, ORC2L, HN1L, NCBP1, CDK2, HEATR1, SERBP1, C13orf34, PLK1, KNTC1, CDT1, AGPS, POLQ, LMNB1, TMPO, SFRS1, BUB1B, CUL4B, NCAPD3, NUDT21, MSH6, CSTF2, DARS2, SKP2, RFWD3, ERLIN1, FEN1, UCK2, HIST1H2BH, PRPF4, TAF5L, TFDP2, MCM3, NDC80, NCAPG, CEP55, UPF2, SRPK1, SFRS2, CPSF6, EIF4A1, STIL, ATAD2, TPX2, GMPS, EEF1G, RAB8A, MAPK14, ZWINT, NFKB1, FANCG, HDAC1, MCM4, KIF2C, CCNA2, LYN, SUPT16H, CDCA8, MELK, IK, BAZ1A, AURKA, CDKN3, NUP155, PFN1, ZWILCH, SMC2, KIF20A, MCM2

11	ARSJ, WEE1, PDGFD, ELOVL2, CENTD3, BAX, ITGA2, RGS4, ACOX2, SHC1, SPRY1, PLA2G5, EFTUD1, LIMS1, UGP2, DNMBP, TXNDC15, EDEM3, BEGAIN, CD63, RAB8B, ITGA3, PXN, PCYOX1L, TNFRSF12A, PTK2B, SNX1, FEM1C, CHRNA9, SLC27A3, MCC, MREG, BDNF, IQSEC1, PDLIM4, FGF14, TNFRSF10B, NMI, CFLAR, F11R, AHSG, CD151, FGF2, GPC4, IQGAP1, FAS, IKBKB, TRIM21, RANBP2, JUN, CP, PLK3, RCAN1, FGFR4, BCL2L1, PARVA, SRPK3, DDB2, ERBB2, AR, ZZEF1, TACC1, ATP5C1, TNFRSF1A, CD46, TICAM1, IRF2, MYD88, RPS6KA3, KCNJ2, HTN1, SYNPO, NEK9, MCL1, RELB, CBLC, MAPK1, FLNA, KRT15, SP110, SP100, TIAM1, EFNB2, PTX3, CDKN1A, LYL1, MMP17, ZBTB38, SPAG1
12	NID1, COL5A1, EDNRA, NOX4, DOCK6, TBX2, ANGPT2, COL4A1, LAMC1, NID2, ELTD1, PLVAP, COL18A1, COL1A1, ENPEP, COL4A2, PXDN, MYO1B, COL5A2, TBXA2R, MCAM, LAMB1, OLFML2A, BGN, NDUFA4L2, CD93, LAMC3, GJA4, PDGFRB, ITGA5, IGFBP4, CD248, PAPSS2, TAGLN, COL6A3, PCDH12, NOTCH3, ITGB1, SMTN, MYH9, CD34, COL1A2, CALD1, COL3A1, FN1, PDLIM1, CHFR
13	RP2, RFC2, FLJ22222, SPC25, CENPQ, RNASEH2A, MXD3, CCDC101, PTTG3, RHOC, TIMM44, ERCC8, SPCS3, ALG6, TARBP2, PHB, LIG3, CTPS2, PIN1, AP1S1, WDR18, PSMA7, PPFIA3, DDX49, HADH, KLHDC3, CD320, RFXANK, CECR5, ATPAF2, SSR2, USP39, DCI, LSM2, ILVBL, BAK1, MEF2B, CDK5, UQCRC1, FBXO5, ILF2, NUP93, RFC5, SUMO2, SFRS3, RAD1, DHX9, EXOSC5, ZNF250, SEPHS1, POLR1C, RAD51AP1, RACGAP1, EIF6, EXOSC9, SUB1, SLC35E1, FANCF, NCKAP1, STIL, GPS1, COPS6, SUMO4, SNRPD1, PARK7, NDC80, BYSL, PCNA, NUP153, NUDT21, NEK2, MED14, SMAD4, RAD51, C2orf44, HTRA2, OIP5, SP3, FANCI, BRMS1, KIF23, POP7, CASK, KIF11, BUB1, LSM4, CDKN2AIP, GNAI3, MOBKL3, NCAPH, CDYL
14	LOX, CAV1, PLAU, TREM1, SRPX2, CA12, VEGFA, GBE1, ABCC3, ARSJ, NRP1, RNASE4, IQGAP1, THBS1, FAM46A, CAST, MMP19, AIM1, FNDC3B, CA9, TGFB1I1, DPP4, CCL20, C5AR1, PTPN12, ZCCHC6, ITGA5, IL1RAP, TPM4, SERPINE1, WWTR1, CHRNA9, ADAM12, GLIPR1, ATP13A3, HSPA6, PLAUR, CD163, SRPR, FLNA, THBD, SHC1, TGFBI, LAMC1, TIMP1, NRP2, CSNK1D, HSPA5, LAMB1, ANXA2, TSPAN4, IL4R, SOCS3, WIPI1, CTSB, ANG, P4HB, WIPF1, CTSL1, IL13RA1, STAB1, EMILIN1, PDIA3, GRN, C1RL
15	BASP1, OPCML, REEP1, NEFL, SNAP91, MYBL1, HOXA7, INA, GTSE1, HOXC10, GNG3, EEF1A2, GRIA2, CENPE, SOX11, MKI67, MLF1IP, CENPF, FANCI, STMN4, TOP2A, HMP19, SCN2A, DTL, REV3L, VAX2, ELAVL4, PLAT, LY6H, TUSC3, ChGn, AP3B2, CSPG4, APOD, RAP1GAP, UBE2C, RIMS2, PHLDA1, CENPM, LMNB2, SCAMP5, COTL1, DUSP26, SATB1, PEG3, SPAG5, CHIC2, PLK1, ACTL6B, TMSL8, CDT1, ACAN, NT5DC2, WSCD1, TEX11, ELMO1, PHF16, GRIA3, CRMP1, LMNB1, TROAP, TRAF4, KIAA0574, CACNA1A, KCNQ2, UGDH, BUB1B, PDGFRA, NOTCH1, ATP1A3, SOX10, SLC17A6, MCF2L, HNRNPA2B1, SYP, ANKS1B, SLC1A1, NRXN1, EVL, BAT1, NTRK3, PAK7, KCNB1, NOL4, HEY2, TTC3, HN1, NDN, SORBS2, PSD, KCNK3, CA10, SLC38A1, SOX12, CELSR3, GFRA2, SYN3, TFAP2A, JPH3, NRXN2, TRIB2, SPRY4, OLIG2, STK32B, ZBED4, GRM5, SMTN, SYNE1, GPR162, CENTG2, NTSR1, CCND1, GABRB3, GABRA3, CASK, MAPK8IP3, AFF3, H2AFX, KIF21B, AGRN, DPF1, FLRT1, SMARCB1, CACNA1G, FGF14, PCDHGC3, CDH7, CAMKK2, HMGB2, MYT1, CDCA3, DDX11, KIAA1009, PSMA2, TACC3, BRCA2, SKP2, DNM1, NKX2-2, COPS4, POLR2A, MMP16, NCAPH, CBX5, WASF1, NLGN3, CDC25A, TRIP13, ATP5J2, E2F3, SFRS1, MGST3, NCAPG, CHAF1A, DOK1, POLR2J, KIF15, ASPM, KIF4A, MAP3K4, MMP15, PSMC2, CUL1, PSIP1, RAP1A, E2F1, MAD2L1, ASL, FBN2, ECT2, SFPQ, NDUFB3, TCERG1, KIF2C, LIG1, MAP3K1, PIK3CG, FTH1, KIF20A, CDC20, CDC6, KIF11, TSPYL4, EXOSC9, C11orf61, MYO9B, ASF1B, CDCA8, TAF1C, UXT, EIF4G3, MAN2A2, MAPKBP1, AURKB, XRCC2, TTK, FUBP1, CEP55, LPHN1, FNTA, TMPO, RPL21, KIF14, RAD51AP1, LRP5, ZWINT, HERPUD1, DPYSL4, STK16, FOXM1, PRC1, MYBL2, ORC6L, IGFBP7, RHOA, CDC45L, LYL1, TRPC3, SAFB, C13orf34, ANG, BBOX1, COPS6, DAPP1, BAT3, ATP6V0E1, HSPB1, SIX1, SLC9A3R1, PCNT, HDAC2
16	GTSE1, NCAPG, CEP55, MKI67, HMMR, CCNA2, DKFZp762E1312, KIF2C, ASF1B, ESPL1, NEK2, CDC6, DLG7, CDC45L, TK1, CHEK1, NEIL3, NCAPH, CENPA, ERCC6L, AURKA, BUB1, KIAA0922, GPR126, CDCA3, ANP32E, MCM10, KIF23, CHEK2, CENPN, C13orf34, SPAG5, PLK1, RAD51, CDT1, CDCA8, MCM4, GEMIN4, POLQ, EXO1, AURKB, NUP155, TROAP, KIF14, TMPO, BUB1B, DDX11, C1orf112, TMEM48, SLC6A15, RAD54L, BRCA2, LIG3, P2RX5, BRCA1, CSTF2, C12orf48, ATAD2, DARS2, FUBP1, SKP2, MYBL2, RFWD3, UCK2, ORC1L, POLG2, SNRPF, CDC25C, POLD1, RP6-213H19.1, ASPM, NDC80, CCNB2, KIF11, KIF15, LMNB1, TRAF5, STIL, POLA2, OIP5, TTK, SPC25, KNTC1, CDK2, TPX2, CDC20, FANCI, CDC25A, FEN1, CENPE, SMC2, TTF2, LMNB2
17	LOX, PLAU, SRPX2, COL5A1, TRAM2, COL6A2, MXRA5, MMP9, THBS1, PRSS23, MMP19, AIM1, COL6A1, NID2, COL18A1, COL1A1, COL4A2, LOXL2, PXDN, STC1, TPM2, ASPN, MYO1B, SPON2, COL5A2, TGFBI, BMP1, LOXL1, MICAL2, CNN2, LAMB1, LUM, PCOLCE, OLFML2A, FBN1, BGN, FN1, LAMC3, MYL9, PDGFRB, ITGA5, IGFBP4, CD248, TAGLN, COL6A3, OLFML2B, ITGB1, ADAM12, MYH9, THBD, COL1A2, COL3A1, IL1R1, CALD1, ITGA4, TPM4, SLC9A1, CD93, SERPINE1

18	SH3GL2, ALDOC, SLC22A17, RAP2A, EFNB3, MAPT, ENAH, C20orf42, PALM, GALNT1, THRA, NTRK2, ADCY2, PGF, MPP2, NRXN2, MAPK8IP1, MCM3APAS, CTNND2, KIF21B, ARHGEF4, FLRT1, CREBBP, AKAP1, NCAM1, EPHB1, DNM3, SIRT1, ZHX2, CTBP2, AMOTL2, SATB1, LRRC1, PIK3R3
19	SLC17A7, GABRA1, DYNC1I1, AK5, SYN1, MYT1L, SLC12A5, CCK, NTSR2, RALYL, NEFM, TAC3, CHGA, ARPP-21, MSTN, PIP3-E, CACNG3, PCSK2, DLG2, ATRNL1, GAD2, NEFH, NPY, SLC8A2, PCP4, SPOCK3, CRHBP, KIAA1324, CLSTN2, DLX2, DVL3, SYN2, SYT1, RAB3A, TSPYL2, PAK3, EPHB6, SNAP25, SH3GL2, NRXN3, KIAA0182, GLS2, ELAVL4, SNCB, STX1A, GNAO1, DNM1, STMN2
20	VSNL1, SNAP25, RUNDC3A, SLC17A7, NEFL, SNAP91, SYT1, HPCAL4, NRGN, GNG3, GABRA1, SERPINI1, AK5, SV2B, CRYM, SYN1, MYT1L, NRIP3, SLC12A5, SNCA, CA11, CCK, KIAA1107, A2BP1, NEFM, PRKCB1, DNM1, FBXO2, CHGA, KCNK1, SNCB, ARPP-21, PPP1R16B, CPNE6, DOC2A, PHYHIP, PIP3-E, CDH18, EPB49, SYN2, SH3GL3, SULT4A1, HTR2A, CACNG3, CCKBR, RAB3A, DLG2, DRD1IP, ARHGDIG, HPCA, TAC1, NCDN, AAK1, RYR2, NPY, DDN, EPHB6, SLC8A2, BSN, KIAA1045, CAMK2A, HTR1E, CNKSR2, NEUROD2, CUTL2, SYP, ICAM5, TRHDE, PSD, CRHBP, GABRA5, CABP1, DLGAP2, GAD2
21	LOX, PLAU, PTX3, SRPX2, CA12, ADAM9, ZYX, BCAT1, BACH1, LOXL2, LIMS1, DNMBP, STK10, PTPN12, GPC1, RAB8B, SERPINB6, PODXL, IL1RAP, FZD5, TPM4, SERPINE1, FEM1C, WWTR1, CHRNA9, ADAM12, CHSY1, ICAM1, GALNT2, FOSL2, ITPKC, ITGA5, PLAUR, CALD1, HSPA5, IQGAP1, TGFBI, LAMC1, SRPR, CAST, MCL1, FLNA, LGALS1, CXCR4, WIPI1, CDKN1A, C5AR1, IL13RA1, NEDD9, SDCBP, FAS, TNFRSF12A, NMI, SHC1, CSNK1D, CALU, GBE1, TNFRSF10B, TREM1, CTNNA1, ACTN1, PARVA, DUSP5, LAMB1, CTSD, DUSP1, COL5A2, GRN, DNAJB1, SWAP70
22	ERC2, SOD2, MAN2B1, FYB, NCF4, FLVCR2, PTPN2, SLAMF8, CD300A, LAIR1, DSE, CD4, SYK, CD86, CTSS, ALOX5, NAGA, MFSD1, MMP19, NCKAP1L, TLR5, NPL, SLC16A3, ARPC1B, COL1A1, DNASE2, CSTA, SERPINA1, IL18, MAP3K8, MS4A6A, ASPN, TRIM38, GRN, CCR5, TLR1, PSCDBP, CPM, COL15A1, FLJ20273, GAL3ST4, C5AR1, CTSL1, PTPRC, CFD, GLT25D1, LHFPL2, NCF2, SLC7A7, C2, ITGB2, DOCK2, RNASE2, RIN3, LILRB1, SCIN, DAB2, SLC11A1, GMIP, RPS6KA1, GJA4, TBXAS1, SIPA1, FXYD5, ARSB, ITGA5, IL10RA, SAMSN1, C1orf38, CYBB, NCF1, TCIRG1, RAB20, GYPC, MSR1, CXorf9, SLCO2B1, SYNGR2, OLFML2B, NOD2, PYCARD, MYO1F, SIGLEC9, TLR2, CENTA2, IFI30, LGALS9, CTSZ, IBSP, CSF1R, WIPF1, GMFG, MGAT4A, SLC15A3, GIMAP4, ALDH3B1, SLC2A5, PLCG2, TNFRSF1B, HCK, BMP2K, VAV1, F13A1, GIMAP6, WIPI1, FCGR2A, HK3, SLA, CD14, TRAF1, MPP1, CTSB, SPTLC2, HLA-C, C1QA, HCLS1, SERPING1, IL4R, CTSD, HERPUD1, FCER1G, ARSA, ALOX5AP, SPP1, FGR, HLA-G, CTSC, SOCS3, MYD88, PLAUR, C1RL, GAA, SPINT2, ITGAM, HLA-B, C1QB, STAT6, CREM, STAT1, BRSK2, NPFFR1, ELF1, BRD8, CEBPB, AP1B1, TIMP1, MERTK, DUSP6, INCENP, TRADD, AAMP, CD81, CSF1, CCR1, CD53, ST14, PRKCD, RAD52, PSAP, YWHAH, THBD, MAPK13, CLIC5, SH3GLB1, TNFRSF10B, IL13RA1
23	SCN3B, MYT1L, PAK3, GNAO1, D4S234E, TAC3, ELAVL4, TRIM36, ACTL6B, BCL11A, DUSP8, SLC17A6, LASS6, FGF9, NOL4, GNAZ, RIMS3, DLX2, ZNF536, KIF21B, DCX, SH3GL2, STMN2, BSN, PCSK2, SYN1, ELMO1, SNAP25, STXBP1, CHGA, ATRNL1, CHGB, INA, NRXN3
24	LAIR1, BACH1, RIPK1, AGPS, TGFBR1, SNAP23, LCP1, GM2A, CYBB, TGFBR2, DDX3X, MSR1, OSMR, SLCO2B1, ADAM10, FLI1, EML4, P2RX4, TMOD3, NFKB1, PRKCD, HLA-DQB1, PICALM, CTSS, WIPF1, NAGA, PTPRC, CSF2RB, SNX2, LPXN, MPP1, GRN, JAK2, IL13RA1, HEXA, NCF2, HCK, TLR2
25	LOX, PLAU, PLA2G2A, TREM1, PTX3, C1RL, VEGFA, SOD2, RCAN1, PRRX1, LTF, CLIC4, TGFB2, MMP19, KLF6, CD44, TWSG1, PBEF1, ZFP36L2, ZFP36L1, EXOC5, STAT3, FAS, SPSB1, TPM4, SERPINE1, OSMR, IBSP, FBLN5, CPD, BCL6, HP, GALNT2, FOSL2, F13A1, ACTN1, C5AR1, FCGR2A, CALD1, IL13RA1, FLII, TLR2, SLA, LAIR1, CAST, CTSB, LCP2, TGFBI, IQGAP1, BIRC3, SRGN, FCGR2B, SQSTM1, FLNA, CP, MAN2B1, WIPI1, FTH1, DUSP3, THBS1, SLC16A3, ITGA5, TNFRSF1B, SLC7A7, SOCS3, MPP1, JUNB, ICAM1, CTSD, SYNPO, PLAUR, HCLS1, SKAP2, TGOLN2, CTSL1
26	RTN3, GNAO1, LRRTM2, FAIM2, ELAVL4, CYFIP2, AP3B2, EHD3, MAPT, KIF1B, APBA2, SATB1, BZRAP1, MXI1, RABGAP1, KIAA0888, SRGAP3, PHF16, KCNC1, CDK5R1, GRIA3, C11orf2, PPP1R12B, PALM, ATP1A3, CLASP2, THRA, ZNF248, BCAN, ALDH5A1, LRP1B, NTRK3, FAM130A2, KCNB1, INPP5A, HERC1, ZMYND11, TMEM59L, SCN3A, OLIG2, KIF3A, PCGF2, MADD, PPP1R9A, GPR162, NCAM1, DLGAP1, MAP2, FRY, DAPK1, SPTBN1, RAPGEF2, GDAP1L1, ABLIM1, FLRT1, SCAPER, DHTKD1, FCHSD2, TSPYL4, SCG3, RAPGEF4, MPDZ, RNF2, NLGN1, NOTCH1, APC, ARHGEF10L, GRIA2, RBM8A, PIK3R1, CTNND2, TCF12, QKI, LPHN1, CRMP1, RAP2A, ICAM1, KIF3C, CSNK1E, TERF2, TAOK3, MCF2L, CLDN4, NRXN2, ABAT, RAB3D, EDNRB, ATP6V1G2, PEA15, NEK1, PTPN22, TERF1, NKX2-2, GOLM1, MYST2, SLIT1, PAWR, NRXN1, CHD9, SMARCC2, TRIM9, CEACAM1, TTC3, ALDOC, TNIK, HIPK2, COL13A1, HDAC4, SHC2, EIF4G3, WIPF2, SIAH1, SPINT1, SNCAIP, SPOP, BMPR2, F11R, C1orf61, DBN1

27	BIRC5, NDC80, NCAPG, NUSAP1, CENPE, CDC2, MLF1IP, CENPF, CCNA2, DKFZp762E1312, PRC1, KIF2C, TTK, FANCI, TOP2A, OIP5, ASPM, PBK, MELK, KIAA0101, DLG7, CCNB2, KIF11, FAM64A, FOXM1, TPX2, GINS2, KIF20A, CENPA, AURKA, RACGAP1, RAD51AP1, SPC25, UBE2C, CDC20, MCM2, CENPN, CCNB1, SPAG5, RAD51, CDCA8, PTTG3, TROAP, MAD2L1, BUB1B, PTTG1, CDC25C, UBE2S, KIF4A, DTL, AURKB, KIF23, CDCA3
28	ARPC1B, HEXB, GRN, CTSA, FLJ20273, SLC7A7, ITGB2, TGFB1, LRP10, MGAT1, SIPA1, FXYD5, IL10RA, PLD3, TCIRG1, CYBA, MYO1F, S100A11, CTSB, GNAI2, IFI30, CTSZ, SH2B3, HMOX1, TRADD, CECR1, NAGA, SERPINA1, LAIR1, ITGAM, FCGR2B, F13A1, RHOG, PLAUR, HCK, FBP1, LILRB1, CAPG, RNASE2, SIGLEC7, CXCR4, CTSS, TLN1, GM2A, HK3, MSR1, CYBB, CSTA, TLR1, RAP2B, TNFRSF1B, VAMP8, CD4, SYK, C5AR1
29	TCF3, POLE2, DCC1, DLG7, CDC45L, NEIL3, RNASEH2A, CDC7, CDKN2C, ORC6L, TRAIP, KIF15, CENPA, RAD51AP1, OIP5, BUB1B, KIF2C, NEK2, MLF1IP, PCNA, AURKB, BIRC5, SFRS3, CDKN3, TTK, SPC25, EZH2, MCM2, CCNB2, FANCI
30	GPR65, FYB, NCF4, SLAMF8, CASP1, CD300A, LAIR1, CD4, SYK, CD86, IL13RA1, CTSS, ALOX5, MFSD1, NCKAP1L, CAPG, TLR5, NPL, ARPC1B, EVI2B, C1S, CSTA, CPVL, SERPINA1, IL18, SELL, MS4A6A, GRN, CCR5, TLR1, FLJ20273, C5AR1, CTSL1, SERPINB1, PTPRC, CFD, CD163, C3, STAB1, LHFPL2, MS4A4A, C1QA, NCF2, SLC7A7, HLA-DQB1, C2, ITGB2, DOCK2, RNASE2, CD37, LILRB1, HLA-DMA, CTSC, DAB2, FTL, LCP1, SLC11A1, SERPINF1, UCP2, CD74, TBXAS1, NPC2, RNASE6, FCGR2A, FXYD5, TREM2, HLA-DPB1, IL10RA, FCGR2B, SAMSN1, GM2A, MFNG, C1orf38, CYBB, FUCA1, LYN, GYPC, MSR1, CXorf9, CYBA, ARHGDIB, SLCO2B1, SYNGR2, PYCARD, MYO1F, CTSB, TLR2, IFI30, CTSZ, FCER1G, CSF1R, LYZ, NINJ1, C1QB, GMFG, ADORA3, MAFB, SAT1, MGAT4A, VSIG4, HLA-DPA1, GIMAP4, VAMP8, SLC2A5, HLA-DRA, PLCG2, LAPTM5, TNFRSF1B, HCK, VAV1, F13A1, MNDA, SLA, AIF1, CD53, C3AR1, LCP2, CCR1, PLEK, HCLS1, SRGN, PTPN6, ITGAM, ALOX5AP, SPI1, CORO1A, FCGR1A, ZNF74, PTAFR, HLA-DMB, GPSM3
31	CAST, CAPG, FER1L3, ARPC1B, CPVL, LEPREL1, COPZ2, ZMYM6, SQRDL, RAB32, LTBP1, FAM26B, TSPO, ANG, S100A4, RRAS, CASP4, CCDC109B, CLIC1, ICAM3, REXO2, CIB1, TRADD, S100A11, SERPINB1, LY96, SERPINA1, HEXB, PROCR, CASP1, NPC2, ASL, RHOG, LYN, ANXA1, NAGA, ANXA11
32	WHSC1, SMARCA5, NFATC2IP, SERBP1, HNRPH3, DDX27, SMC6, DHX29, EWSR1, DNMT1, ZC-CHC6, TNPO3, THOC2, HNRNPA2B1, STAG1, SCRIB, EIF3B, PDPK1, AHCTF1, TPR, CHD4, TOP1, MADD, SMC3, ESF1, MED1, ABCF1, MTR, KHDRBS1, NCL, FBXW2, GPATCH8, NFIC, HNRPM, UBXD7, TRRAP, AKAP13, RBBP6, NIPBL, EIF3A, KPNB1, RBM10, RPS6KB1, EIF4G3, SON, SPEN, SART3, RARS, DCP1A, PNN, SFRS2IP, NCOA6, ZCCHC14, CHD1, FXR1, PAXIP1, OXSR1, NUP153, SMC1A, MBD1, KPNA6, SMARCA4, LUC7L2, DVL3, RFC1, RANBP2, MSH3, MAML1, DEK, MAP4K4, YLPM1
33	PYGL, PTX3, ABCC3, SLC43A3, G0S2, DYNLT3, SLC2A10, PDPN, TRIM5, PTRF, EMP3, DRAM, FLJ21963, CLDN10, TRIP4, MSN, TAGLN2, LOC26010, C1S, RBP1, CSTA, LEPREL1, EFEMP2, TRIP6, SLC25A20, ANXA1, COPZ2, UPP1, DDB2, FAM26B, ANXA2P2, RRBP1, GNG12, CD63, SWAP70, ANXA5, tcag7.1314, KLHL26, FAS, CSRP1, PARP12, TNFRSF1A, TMBIM1, CD151, C13orf18, OSBPL3, LGALS3, CCDC109B, PGCP, SLC27A3, LDHA, TNFAIP6, CLIC1, GSTK1, CXCL14, ANXA2, KIAA0323, RGN, TIMP1, PDLIM4, CAST, CBR1, ANG, PLA2G5, RCAN1, STEAP3, HEXB, SORT1, CTNNA1, IGFBP2, NUCB1, IQGAP1, PLAUR, S100A13, CA12, BCAP31, TNFRSF12A, VAV3, NUAK2, PDGFA, PLAU, VIM, DAG1
34	NASP, NOL8, HNRPH3, SMC1A, EWSR1, DNMT1, ATF7IP, RFC1, NIPBL, TOP1, PHF2, HNRNPA1, KHDRBS1, TRRAP, SART3, RBBP6, CPSF6, ILF3, TLK2, GPATCH8, MDC1, SPEN, ZNF638, STAG1, XPO6, EP400, RBM10
35	PYGL, STEAP3, CA12, PLOD2, VEGFA, P4HB, CALU, IGFBP2, SERPINH1, PDIA4, TRIP6, IGFBP3, PLOD3, GPI, SMS, SERPINB6, CANX, CD151, FEM1C, YKT6, CCDC46, GNB2, PDIA3, NSUN5, LAMC1, HSPA5, RIMS2, ZYX, IL1RAP, ITGA5, AEBP1, COL4A2, ADAM12, SPRY4, PRKCSH, KHDRBS2, ATP6V1G2, SNAP91, IQGAP1, CAPNS1, SPRY1, PLAU, PLAUR, NDN
36	VAX2, RALGPS1, SEC61A2, ZNF571, MXD3, ACTL6B, SPAST, ELF2, RTN2, ZFP37, ZBTB5, SLC17A6, REXO4, PAK7, PDCL, SH3GLB2, C14orf104, ZNF250, LOC81691, COQ7, C9orf7, C9orf91, CP110, PRR3, AGTPBP1, PAIP2B, C5orf30, RNF5, CCDC101, YWHAQ, PAIP1, STMN1, SIRT1, BGLAP, H3F3A, STAM, BMI1, RPS6KA5, MATR3, SUMO2, VPS72, RB1CC1, SEPHS1, FANCE, BUB3, POLD3, YEATS4, MIS12, ING1, PSIP1, POLA2, RP2, MBIP, PMF1, GMNN, TOPORS, PPP1R8, DYNC1LI1, CACYBP, MCRS1, CEP72, APTX, HSF2, EED, RAB4A, CDKN1B, RACGAP1, NCAPD3, ENSA, TFAM, PPM1D, SMC2, NOC4L, SSNA1, AIP, SRPK1, C1D, RBMX, UBE2E3, SFRS2B, RPS3
37	ASF1B, CDC6, CHEK1, NEIL3, NCAPH, SMCHD1, CDK2, KIF23, RFC5, KIF14, TMPO, RAD54L, E2F8, WDHD1, BRCA1, RIF1, ATAD2, CENPA, KIF15, KIF4A, BUB1, AURKB, TTK, KIF2C, CCNA2, STIL, ASPM, PLK4, BIRC5, FANCI, NCAPG, MXD3, NEK2, CDCA3, ECT2, RAD51AP1, KIF11, CENPF, EZH2, BUB1B, CENPE, HMMR, OIP5, CDCA8, MAD2L1, SPAG5, TPX2, TRIP13

38	REEP1, DDAH1, SOX11, WASF3, FLJ10781, NDRG4, PCDH9, SLC6A1, RAB6B, MAPT, NUDT11, KLHDC8A, C1orf21, WSCD1, PFTK1, CRMP1, TTYH1, NCALD, CLASP2, CDH2, BCAN, NOL4, DPP6, PSRC1, NOVA1, NCAN, MAP2, CTNND2, ITM2C, GRIA2, NKX2-2, PLP1, DTNA, APBA2, TRIM9, NCAM1, MAGI2, APC, SCG3, ABAT, TSC22D4, GRIA3, ATP6V1G2, ANK2, STMN4, BBOX1, S100B, GAP43, AQP4, TNIK, NFASC, NES
39	GABBR1, RALGPS1, ProSAPiP1, PID1, PALM, THRA, ALDH5A1, NTRK3, ADCY2, RNF208, TMEM59L, NRXN2, GPR162, CRY2, MAPK8IP3, SCAPER, ATP6V1G2, MAPT, NRXN1, SRGAP3, NCAM1, CTNND2, SCG3, SALL2, NCOA1, TAOK3, MAPK10, AKAP6, DPP6, KIF3A, SLC6A1, FAIM2, APC2, APBA2, SORBS1, GNAO1, HIPK2, SCN2A, NEK1, GRIA2, ACP5, CDK5R1
40	EZH2, NCAPG, CCNE2, MKI67, CA3, DKFZp762E1312, FANCI, TOP2A, WHSC1, KIF15, NCAPH, MYB, SOX4, BUB1, NASP, FANCC, TIMELESS, KNTC1, CDT1, EXO1, MSH5, LMNB1, TACC3, RAD54L, E2F8, BAT1, HDAC2, FZD2, LOC81691, SKP2, C9orf39, POLD1, KIF11, CHEK1, DTL, BRCA2, CHAF1A, BRCA1, MCM2, KIF2C, SPAG5, CDCA8, MCM7, TPX2, PLK4, TTF1, CBX5, NCAPD3, MCM4, PRPF4, KIF4A, CDK2, CENPF, TLK2, TMPO, RFC5, NUP93, ILF3, BUB1B, FEN1, SMC3, SFRS1, SFPQ, MUS81, KIF5B, TAF5, CDC7, DEK, NDC80, POLD3, TTK, TUBG1, PRC1, MCM3, SSRP1, ASPM, WAC, RFC4, DNMT1, BUB3, PLK1, TRIM28, PRPF3, MELK
41	LOX, PLA2G2A, TREM1, SOD2, CXCL5, SLAMF8, DSE, MFSD1, AIM1, SLC16A3, STC1, MAP3K8, IL1R2, CCL20, C5AR1, CD163, C2, RIN3, CTSC, SLC11A1, ADFP, BIRC3, SLC39A14, MBD4, IL10RA, CDCP1, C1orf38, SPSB1, OLFML2B, IBSP, MAFB, SAT1, GLIPR1, HP, SLC2A5, HMOX1, TNFRSF1B, FOSL2, F13A1, PLAUR, CTSB, S100A8, THBD, FPR1, FCGR2A, CD14, VSIG4, STAB1, CEBPB, TLR2, IL4R, MAPK13, CSTA, PTPN2, IER3, ALOX5, CTSL1, S100A11, TIMP1, NOD2, SYNPO, IL6, SLA, JUNB, TGFBI, SRGN, DAB2, TNFAIP3
42	PYGL, G0S2, RNASE4, PDPN, EMP3, IGFBP2, CAPG, SNX10, CD44, EFEMP2, TRIP6, UPP1, PLP2, ANG, TMBIM1, LGALS3, CCDC109B, FABP5, HRH1, NUPR1, S100A13, TIMP1, PLAU, LGALS1, CBR1, CLIC1, ANXA2, MSN, FZD7, ANXA1, COPZ2, PTRF, S100A11, PLAUR, EPHB1, CXCR4
43	BIRC5, MLF1IP, CENPF, OIP5, CCNB2, ECT2, DTL, GINS2, FBXO5, KIF23, MCM2, TYMS, PCNA, CCNB1, RNASEH2A, GINS1, H2AFZ, MAD2L1, DTYMK, DBF4, RFC4, CKS2, C9orf46, HN1, PTTG1, CKS1B, DEPDC1, ZWILCH, TRMT5, LSM2, TTK, PBK, MELK, RAD51AP1, UBE2C, TOP2A, NDC80, CDC20, BUB1B, CDKN3, NCAPG, CENPA, SPC25, AURKB, FANCI, RACGAP1, KPNA2, STIL, KIF4A, ASPM, AURKA, EZH2, KIF2C, RAD51
44	POSTN, ELOVL2, SLC25A15, JAG1, ELAVL1, PRPF31, ZNF227, ZNF313, ZNF473, HMG20B, TH1L, RAE1, POFUT1, ITCH, DDX49, PDCD5, DYNLRB1, RALY, EIF6, CPSF6, CSNK2A1, TSC2, CTNNBL1, VPS72, PPP2R2D, PA2G4, PTPN22, NUP93, DHFR, FSD1, ZNF76, AATF, MAD1L1, CSTF2, AES, ARHGDIA, ADRM1, DHX30, DHX38, PRCC, NONO, CR1, ZFP64
45	SMARCA5, PDLIM5, YES1, NFATC2IP, HIP1, AKT1, DHX29, LUC7L2, TRIM44, DDX3X, ZNF532, ZNF12, RPS6KA2, BAZ1B, TOP1, SMC3, ESF1, ABCF1, KIAA0329, PHF2, NCL, TRIM26, AATF, UBXD7, TRRAP, AKAP13, STAG1, CPSF6, RBBP6, KHDRBS1, SPAG9, EIF5B, GTF2I, TRIM27, FBXW2, TCF4, TOP2B, ZW10, NBR1, KIDINS220, DHX30, ARHGEF7, KPNB1, TNPO3, BPTF, RFC1, STX6, ANP32B, RBM10, NCOA6, XPC, SFRS2IP, SND1, MBD1, MAP4K4, ZCCHC14, ANAPC1, ZNF638, ARIH2, NXF1, DVL3, SART3, XPO6, RBM8A, DCP1A, SPEN, HGS, SFRS1, MAML1, SERTAD2, ATG4B, CSNK1A1, SPG7, TAF6, RBM14, PDXDC1, TOPBP1, ZNF148, EWSR1, FBXW11, NIPBL, MED1, ERCC5, CTBP2, CLASP1, U2AF1, GTF2F1, POLR3E, GNA11, FXR1, TPR, SSB, PABPC4, DNMT1, GPATCH8
46	SMARCC1, ATAD2B, ZNF426, NASP, GOLGA1, SPAST, PRPF4B, PHF16, ZFP37, DIS3, FAM48A, RBM12, CASP8AP2, RBM4, PDCL, DHX35, RBM4B, RFWD3, ZER1, ZBED4, CEP110, MSL2L1, WDR68, HMG20A, ILF3, TLK2, PTK2, SRPK1, ORC2L, STX5, SMG1, NUP155, ANP32A, SUZ12, NCL, EXOSC2, C11orf30, USP39, SETDB1, TTF1, DEDD, GSPT1, CPSF6, WBP11, REV1, BCOR, PCBP2, DDX18, RAD1, SAFB, CSTF1, UBTF, RIF1, STRN3, MDC1, NFYC, TSC2, FUS, NFRKB, RRN3
47	PRKD2, CHAF1A, RPN2, HNRNPA2B1, SSRP1, SLC1A4, KEAP1, CPSF6, ILF3, TOP1, SMC3, DVL3, KHDRBS1, NFIC, AATF, HNRPM, AKAP13, XPO6, SMARCA4, GTF2F1, CHD4, PABPN1, OS9, TNPO3, HCFC1, CSNK2A1, KPNB1, DCP1A, KPNA6, CKAP4, EIF3B, NUDC, NCOA6, ABCF1, SEMA4C, RBM10, GATAD2A, TGFBRAP1, MED1, PRKCSH, GNA11, NCL, POLR3E, EIF4G1, BRD4, ABL1, YLPM1, WNK1, ATF7IP, DDX27, ZC3H11A, GOSR1, DHX30, TESK1, SRRM2, EPRS, TRRAP, GTF2I, GOLGA1
48	HNMT, DPYD, CTBS, GBP2, SP100, CASP1, IFI35, PLTP, NMI, CAPG, FAH, CSTA, CYBRD1, ANXA1, COPZ2, TMEM140, TRIM38, ZMYM6, HLA-B, ANXA4, APOBEC3G, SERPINB1, HOMER3, ITGB2, LRP10, ANG, EFEMP1, NPC2, HLA-E, FAS, YAP1, TMBIM1, S100A10, RRAS, LYN, RARRES3, LAMP2, PGCP, CASP8, ECM2, STEAP3, NAGA, LY96, FTL, BMP2K, SAMSN1, S100A13, TSPO, RHOG, IL10RA, CCR1, BLVRB, C1RL, TLR1, RNASE2, LAIR1, PROCR, S100A11, EMP3, ZNF74, TLR5, PAK7, TRIP6, VAMP8, GM2A, CD86, RNASE6, FYB

49	FYB, NCF4, CD4, SYK, CD86, KIAA0143, TLR5, EVI2B, IL18, LHFPL2, GMIP, TBXAS1, PTPN18, SAMSN1, RAB20, MYO1F, TLR7, GIMAP4, SLC7A7, LAIR1, CTSS, IL10RA, CYBB, HCK, ITGB2, LCP2, MNDA, PTPRC, TLR2, TLR1, LILRB1, HLA-DMA, HCLS1, SPI1, SLA, RNASE6, CSF3R, CCR1, ITGAM, CSF1R, MPP1
50	SOX11, BCHE, ANKRD46, ASCL1, TSPAN12, BMP7, PROM1, KCNQ2, BCAN, DGKB, SCN3A, PLA2G6, OLIG2, SEZ6L, DLGAP1, MAP2, GDAP1L1, GTF2I, DPF1, ARHGEF7, RAPGEF4, FCHSD2, MAPT, TOP2B, RBM4B, GRIA2, AP3B2, MARK1, ZFP2, RBM12, IL1R1, NOVA1, DBN1, SCG3, TCL1A, TRIM37, CRMP1, TRIM24, PAFAH1B3, MYST2, DPP6, NCAN, REV3L, NKX2-2, CUL3, APBA2, IPO9, APBB2, CLASP2
51	ERC2, TFEC, PLAC8, FYB, CD300A, SYK, CD86, ALOX5, NCKAP1L, IL18, MS4A6A, SERPINB1, PTPRC, TSPO, C1QA, SLC7A7, ITGB2, DOCK2, RNASE2, DLGAP2, SCIN, RPS6KA1, TBXAS1, TREM2, RNASE3, SAMSN1, HLA-DQA1, CXorf9, IGSF6, CYBA, PYCARD, LY96, TLR2, LGALS9, CD69, FCER1G, C1QB, ADORA3, VSIG4, VAMP8, HCK, MNDA, LCP2, CASP1, SRGN, LAIR1, CCR1, TLR1, CD4, BCL2A1, ARHGAP15, AIF1, FCGR1A, FCGR2A, CD14, CTSS, SERPINA1, C3AR1, CSTA, NCF2, TLR7, LILRB1, CEBPA, CORO1A, RNASE6, IL10RA, CD53, MYO1F, CYBB, MSR1, NCF4, MAFB, LPXN, HLA-DMB, PLEK, GMFG, LYN, CSF2RB, HMOX1, CAPG, LYZ, PIK3CG, CSF1R, HLA-DPB1, RAC2, LAPTM5, SLA, APOBEC3G
52	RUNDC3A, TAGLN3, STMN2, OPCML, PFN2, UCHL1, DYNC1I1, HMP19, ELAVL4, PGRMC1, HOXC6, B4GALNT1, DUSP26, NMNAT2, HOXC4, TMEM16C, PROM1, RAB33A, GSTA4, PAK7, CA10, SPTBN2, HIST1H1C, FGF12, SOX10, TUBB4, PAK3, CLASP2, INA, DNM3, MYT1, DCX, MBP, TTC3, CDK5R1, MAGI1, SNAP91, SMPD4, CYFIP2, PODXL2, RAP2A, TFAP2A, STMN4, TF, AMPH, YAP1, NCAM1, PLCB1, SCAMP5, NOL4, POLR2D, WASF1, PPFIA2, GNG4, SOX11, CLIC1, SFPQ
53	OMG, ALDOC, NDRG2, RND2, ASCL1, DNAJC6, MAPT, SCG3, THRA, BCAN, NTRK2, ABCC8, SCN3A, NRXN2, OLIG2, CTNND2, DPP6, APBA2, GRIA2, ABAT, SLC6A1, ATP1A2, GRIA3, NCAM1, CLASP2, TSC22D4, NOVA1, ATP6V1G2, KIF5C, SCN2A, KIF1B, MAGI2, PLP1, NKX2-2, CRMP1, RUFY3, SALL2, CTNNA2, MAP2
54	PPIC, HEXB, ZMYM6, RAB32, SLC25A24, LUM, TEK, SLIT3, PROCR, C1orf78, CLIC2, GNG11, TNFSF10, CASP4, IMPA2, LEPR, CSF3R, LILRB2, MALL, ERG, MME, IL7R, NPC2, PLA2G1B, RBPMS, CITED2, TSPAN4, SOSTDC1, GATA6, STAT4, TGM2, FBP1, GZMA, TGFBR2, CST7
