# Supplementary material for: A Computational Approach to Identifying Gene-microRNA Modules in Cancer
Source: PLoS Comput Biol. 2015 Jan 22;11(1):e1004042. doi: 10.1371/journal.pcbi.1004042 (PMC4303261; doi:10.1371/journal.pcbi.1004042)
Supplement: S4 Table — (PDF) [file pcbi.1004042.s011.pdf]

Table S4. MiRNAs in GBM modules.

Module ID	microRNAs
1	miR-19a, miR-19b, miR-222, miR-17, miR-34b, miR-454, miR-130b, miR-22
2	miR-130b, miR-93, miR-106b, miR-15b, miR-29a, miR-19b, miR-17, miR-19a, miR-106a, miR-29b, miR-25, miR-18a, miR-20b, miR-20a, miR-29c, miR-92, miR-22, miR-454, miR-18b, miR-34b, miR-34a, miR-222, miR-152, miR-10b, miR-223, miR-550, miR-363, miR-30a, miR-16
3	miR-22, miR-483
4	miR-146b, miR-155, miR-142, miR-34a, miR-193a, miR-19b
5	miR-130b, miR-93, miR-106b, miR-29a
6	miR-219, miR-338, miR-330, miR-124a, miR-139, miR-128b, miR-128a, miR-137, miR-218, miR-29c, miR-129, miR-138
7	miR-130b, miR-93, miR-106b, miR-15b, miR-17, miR-19b, miR-106a, miR-19a, miR-25, miR-18a, miR-29a, miR-20a, miR-29b
8	miR-223, miR-21, miR-142, miR-22, miR-9, miR-146b, miR-222, miR-155, miR-34a, miR-181d, miR-33, miR-181a, miR-193a, miR-34b, miR-221, miR-19b, miR-181c, miR-17, miR-301, miR-19a, miR-128b, miR-181b, miR-95, miR-93
9	miR-338, miR-128b, miR-222, miR-27a, miR-124a, miR-23a, miR-128a, miR-219, miR-138, miR-152, miR-155, miR-139, miR-221, miR-204, miR-340, miR-34a, miR-10b, miR-181a, miR-7, miR-342, miR-577, miR-504, miR-34b, miR-17, miR-146b, miR-19b, miR-106a, miR-20b, miR-301, miR-93, miR-181b, miR-22, miR-223, miR-363, miR-18a, miR-501, miR-20a, miR-181d, miR-19a, miR-324, miR-193a, miR-153, miR-9
10	miR-130b, miR-15b, miR-93, miR-19b, miR-19a, miR-17, miR-106b, miR-29a, miR-29b, miR-29c, miR-106a, miR-18a, miR-20a, miR-454, miR-92, miR-20b, miR-25, miR-22
11	miR-34a, miR-222, miR-221, miR-155, miR-21, miR-34b, miR-27a
12	miR-214, miR-199a, miR-145, miR-143, miR-422b, miR-128a, miR-129
13	miR-130b, miR-93, miR-15b, miR-19b, miR-106b, miR-19a, miR-17, miR-106a, miR-20a
14	miR-21, miR-222, miR-155, miR-223, miR-128b, miR-210, miR-128a, miR-221, miR-9, miR-34a, miR-181c, miR-142, miR-33, miR-22, miR-146b, miR-181a, miR-95, miR-181d, miR-214, miR-139, miR-23a, miR-488, miR-34b, miR-187
15	miR-130b, miR-93, miR-17, miR-19b, miR-152, miR-29a, miR-22, miR-106a, miR-222, miR-19a, miR-106b, miR-20a
16	miR-130b, miR-93, miR-15b, miR-106b, miR-19b, miR-29a, miR-17, miR-19a, miR-29b, miR-106a, miR-18a, miR-25, miR-29c, miR-20a, miR-92
17	miR-214, miR-199a, miR-21, miR-9, miR-128b, miR-128a, miR-181c, miR-223, miR-422b, miR-95, miR-488, miR-155, miR-101, miR-145, miR-222, miR-33, miR-129, miR-30c, miR-199b, miR-210, miR-598, miR-181d, miR-181a, miR-769, miR-143
18	miR-222, miR-128b, miR-155, miR-221
19	miR-124a, miR-129, miR-7, miR-139, miR-128b, miR-218
20	miR-124a, miR-129, miR-7, miR-139, miR-218, miR-137, miR-128b, miR-128a, miR-203, miR-433, miR-132, miR-323, miR-329, miR-338, miR-485, miR-410, miR-219, miR-504, miR-29c, miR-330, miR-138, miR-383, miR-769, miR-214, miR-629, miR-517c, miR-210, miR-422b, miR-381, miR-23a, miR-155
21	miR-21, miR-222, miR-155, miR-221, miR-34a, miR-223, miR-128b, miR-128a, miR-210, miR-181c, miR-139, miR-181a, miR-146b, miR-142, miR-504, miR-27a, miR-22, miR-129, miR-33, miR-23a, miR-9, miR-34b, miR-95, miR-301, miR-101, miR-187, miR-181d, miR-214, miR-422b, miR-181b, miR-99a, miR-124a, miR-199a
22	miR-142, miR-223, miR-21, miR-22, miR-146b, miR-34a, miR-181d, miR-9, miR-222, miR-155, miR-33, miR-34b, miR-181c, miR-181a, miR-193a, miR-19b, miR-221, miR-93, miR-17, miR-130b, miR-301, miR-106b, miR-181b, miR-92, miR-19a, miR-106a, miR-95, miR-20a, miR-185, miR-25
23	miR-124a, miR-7, miR-128b, miR-139
24	miR-142, miR-223, miR-21, miR-34a, miR-146b, miR-155, miR-181d, miR-22, miR-181c, miR-222, miR-34b, miR-181a, miR-221, miR-9
25	miR-21, miR-223, miR-142, miR-155, miR-222, miR-22, miR-34a, miR-9, miR-146b, miR-33, miR-221, miR-181a, miR-128b, miR-210, miR-34b, miR-128a, miR-181d, miR-193a, miR-181c, miR-301, miR-17, miR-181b, miR-95, miR-19b, miR-23a, miR-488, miR-19a, miR-93, miR-20b, miR-363, miR-106a, miR-153, miR-27a
26	miR-9, miR-128b, miR-181d, miR-128a, miR-181a, miR-21, miR-222, miR-155, miR-22, miR-95, miR-23a, miR-193a, miR-98, miR-223, miR-301, miR-340, miR-181c
27	miR-130b, miR-93, miR-106b, miR-15b, miR-17, miR-29a
28	miR-142, miR-21, miR-223, miR-34a
29	miR-130b, miR-93, miR-106b, miR-15b, miR-17, miR-19b, miR-29a

30	miR-142, miR-223, miR-21, miR-22, miR-181d, miR-146b, miR-34a, miR-9, miR-34b, miR-33, miR-222, miR-193a, miR-130b, miR-19b, miR-155, miR-181a, miR-181c, miR-92
31	miR-142, miR-21, miR-222, miR-155, miR-22
32	miR-29a, miR-22, miR-19a
33	miR-222, miR-34a, miR-155, miR-221, miR-34b, miR-21, miR-128b, miR-27a, miR-128a, miR-146b, miR-23a, miR-22, miR-504, miR-181a, miR-223, miR-142, miR-17, miR-340, miR-139, miR-19b, miR-204, miR-301, miR-193a, miR-19a, miR-181b, miR-152, miR-92, miR-181d, miR-181c, miR-9, miR-106a, miR-342, miR-338, miR-33, miR-93, miR-20b, miR-20a, miR-148a
34	miR-22, miR-29a, miR-19a, miR-19b
35	miR-155, miR-128b, miR-222, miR-128a, miR-139, miR-504, miR-221, miR-21, miR-34a, miR-129, miR-27a, miR-210, miR-219, miR-124a, miR-338, miR-23a, miR-101, miR-223, miR-204, miR-491, miR-422b, miR-342, miR-34b, miR-181c, miR-340
36	miR-19a, miR-19b, miR-93, miR-17, miR-22, miR-222, miR-20a, miR-130b, miR-301, miR-106a, miR-9, miR-20b, miR-181d, miR-223, miR-34b
37	miR-130b, miR-93, miR-106b, miR-15b, miR-19b, miR-29a, miR-17, miR-19a, miR-29b, miR-25, miR-106a, miR-18a, miR-29c, miR-20a
38	miR-9, miR-181d, miR-128b, miR-128a, miR-181a, miR-21
39	miR-128b, miR-128a, miR-9, miR-21, miR-155, miR-95, miR-181d, miR-181a, miR-598, miR-214, miR-98, miR-124a, miR-139, miR-181c, miR-222, miR-193a, miR-187, miR-23a, miR-488, miR-199a, miR-22
40	miR-130b, miR-93, miR-29a
41	miR-21, miR-223, miR-142, miR-9, miR-22, miR-181d, miR-155, miR-146b, miR-222, miR-33, miR-181c, miR-181a, miR-34a, miR-193a, miR-488, miR-95, miR-210, miR-34b, miR-301, miR-128b, miR-221, miR-128a, miR-19b, miR-17, miR-181b
42	miR-222, miR-155
43	miR-130b, miR-93, miR-106b, miR-15b, miR-17, miR-19b, miR-25, miR-29a, miR-18a
44	miR-130b, miR-17, miR-93, miR-20a
45	miR-22, miR-29a
46	miR-19b, miR-19a, miR-22, miR-17, miR-29a
47	miR-29a, miR-22
48	miR-142, miR-34a, miR-22, miR-223, miR-21, miR-222, miR-146b, miR-155
49	miR-142, miR-223, miR-21, miR-22, miR-34a
50	miR-9, miR-181d, miR-22, miR-181a, miR-222, miR-21, miR-93, miR-17, miR-223, miR-152, miR-20a, miR-128b, miR-20b, miR-142, miR-155, miR-193a, miR-181b
51	miR-142, miR-223, miR-21, miR-22, miR-34a, miR-146b, miR-181d, miR-9, miR-34b, miR-222, miR-130b, miR-33, miR-193a, miR-19b, miR-181a, miR-155, miR-92
52	miR-124a, miR-128b, miR-128a, miR-23a, miR-27a, miR-338, miR-222, miR-139, miR-138, miR-155, miR-7, miR-219, miR-10b, miR-152, miR-221, miR-340, miR-204, miR-34a, miR-504, miR-181a, miR-137
53	miR-128b, miR-9
54	miR-142, miR-181d, miR-223, miR-21, miR-9, miR-181a, miR-22, miR-222, miR-199a, miR-155
