# Supplementary material for: A Computational Approach to Identifying Gene-microRNA Modules in Cancer
Source: PLoS Comput Biol. 2015 Jan 22;11(1):e1004042. doi: 10.1371/journal.pcbi.1004042 (PMC4303261; doi:10.1371/journal.pcbi.1004042)
Supplement: S5 Table — ‘Num’ represents the number of ovarian cancer genes (or ovarian cancer miRNAs) / the number of all genes (or all miRNAs) in a module. (PDF) [file pcbi.1004042.s012.pdf]

Table S5. Cancer genes, ovarian cancer genes and ovarian cancer miRNAs in modules.

Module ID	Cancer Genes	Num	Ovarian Cancer Genes	Num	Ovarian Cancer microRNAs	Num
1	BOP1	1/25	PTK2	1/25	miR-151, miR-30b, miR-30d, miR-338, miR-9	5/11
2	CD44, MMP9, PLAUR, LTB, GBP1, CTSH, EPB41L3, POU2AF1, VAV1, CXCL10, MEF2C, HCK, BTK, CASP1, CD74, LCK, LYN, FGR, SPP1	19/60	CD44, DPYD, IL18, MMP9, PLAUR	5/60	miR-125b, miR-146a, miR-155, miR-17, miR-20a, miR-21, miR-218, miR-22, miR-223, miR-224, miR-335	11/24
3	CDK2, E2F1, PLK1, MCM2, CDC6, EZH2, ASPM, BUB1	8/35	CDK2, E2F1	2/35	miR-106b, miR-130b, miR-18a, miR-19a, miR-25, miR-29a, miR-93	7/14
4		0/48	DLEC1	1/48	miR-34b, miR-34c, miR-449a, miR-449b	4/6
5	BOP1, PPP1R14B	2/30	PTK2	1/30	miR-151, miR-30b, miR-30d	3/4
6	BARD1, CDC25A, CDK2, MSH6, MCM2, BUB1, FEN1, PCNA, CDKN3	9/34	BARD1, CDC25A, CDK2, MKI67, MSH6	5/34	miR-101, miR-106b, miR-130b, miR-17, miR-18a, miR-19a, miR-20b, miR-25, miR-29a, miR-93	10/20
7	BOP1, RECQL4	2/25	PTK2	1/25	miR-151, miR-30b, miR-30d	3/7
8	PLAUR, MMP11, BGN, COL16A1, THBS2, THBS1, VCAN, COL1A1, TIMP3, PDGFRB, COL1A2	11/39	FN1, LGALS1, PLAU, PLAUR, SERPINE1	5/39	miR-152, miR-199a, miR-214, miR-22	4/8
9	ACVR2A, RLF	2/18	ACVR2A	1/18		0/2
10	CD74, NCAPD2, FOXM1, RAD52	4/13		0/13	let-7b, miR-106b, miR-141, miR-146a, miR-200c, miR-21, miR-224	7/9
11	TBX2	1/13		0/13	let-7b, miR-130a, miR-22	3/3
12	E2F3, MCM2, FEN1, DEK, PALB2, PSMA5	6/33	E2F3, NBN	2/33	miR-93	1/2
13	CDC42, PLK1, CDC6, BUB1, PCNA, UCHL5, FANCE, SMARCB1, FANCG, EIF4EBP1, ECT2	11/78	CDC42	1/78	miR-18a, miR-25, miR-29a, miR-93	4/8
14	CAMTA1, DBN1, SOX11, POU2F2, BCL3, MAPK7	6/25	MAP3K4	1/25	miR-125b, miR-130a, miR-218	3/6
15	SOX11, BCL3, RHOBTB1, NDRG1	4/35	INSR	1/35	let-7b, miR-20a, miR-9	3/5
16	GADD45GIP1, EPS15L1	2/25		0/25	miR-27a, miR-629	2/5
17	C11orf30, MSH2, CTSH, RLF, NCAPD2, MARS, PPFIBP1, MYBL2	8/29	C11orf30, MSH2	2/29	let-7b, miR-148b, miR-29a, miR-93	4/7
18	MCM2, FEN1, FOXM1, DEK, FANCG, WHSC1	6/31	MKI67	1/31	miR-18a, miR-25, miR-29a, miR-93	4/7
19	CD82, CTSB, ZDHHC18, ALS2CL, ETV6, PRKCD, MAPKAPK2, TFEB, PML	9/25	CD82, CTSB, MAP3K3	3/25	let-7b, miR-130a, miR-17, miR-20a, miR-218	5/8
20	AURKA, CDC20, MAD2L1, TOP2A, PLK1, ASPM, BUB1, FOXM1, MYBL2, KIF14, CCNA2, CCNB1, BUB1B	13/44	AURKA, CDC20, MAD2L1, TOP2A	4/44	miR-101, miR-17, miR-18a, miR-19a, miR-29a, miR-93	6/13
21	HCK, BTK, LCK, IL2RG, IL2RB, ITK, CCR1, LAPTM5	8/30		0/30	miR-146a, miR-155, miR-21, miR-218, miR-22, miR-223, miR-224	7/17
22	MMP2, MMP11, THBS2, VCAN, COL1A1, LOXL2, ADAM12, DPT, ECM1	9/27	FN1, MMP1, MMP2, PLAU, SPARC	5/27	miR-152, miR-214, miR-22	3/6
23	RBBP5, NUP133	2/19	ARID4B	1/19	let-7b, miR-17	2/2
24	PDGFRA	1/19	PDGFRA	1/19	miR-127, miR-145, miR-376c	3/16
25	MCM2, FEN1, PCNA, MYBL2, FBXO5	5/29		0/29	miR-18a, miR-25, miR-29a, miR-93	4/8

26	MAD2L1, PLK1, FEN1, PCNA, UCHL5, CCNA2, CCNB1, FBXO5, RAP1GDS1, RAN	10/44	MAD2L1	1/44	let-7b, miR-101, miR-17, miR-18a, miR-19a, miR-25, miR-29a, miR-93	8/17
27	MMP14, MMP2, MMP11, BGN, COL16A1, THBS2, THBS1, VCAN, COL1A1, PDGFRB, COL1A2, LOXL2, ADAM12, ECM1, COL11A1, TWIST1, SFRP4, LOX, TAGLN, LHFP	20/55	FN1, MMP14, MMP2, PLAU, SERPINF1, SPARC	6/55	miR-127, miR-145, miR-152, miR-199a, miR-214, miR-22	6/12
28	DNMT1, HMGA1, NRF1, SMARCA4	4/37	DNMT1, HMGA1, LGALS1	3/37	miR-27a	1/3
29	MYCL1, RLF, YBX1	3/23	MYCL1	1/23	miR-30c, miR-93	2/5
30	PBX2, DAXX	2/26		0/26	let-7b, miR-106b, miR-20b, miR-219, miR-25, miR-93	6/13
31	CD82, CTSB, STAT3, TNFSF10, GBP1, EPB41L3, CXCL10, CASP1, LYN, SPP1, LAPTM5, IRF1, CTSL1, TACC1, S100A13, CAPG	16/65	ACVR2B, CD82, CTSB, CTSD, DPYD, RAB25, SERPINF1, STAT3, TN-FSF10	9/65	miR-125b, miR-130a, miR-146a, miR-155, miR-17, miR-183, miR-20a, miR-20b, miR-21, miR-218, miR-22, miR-223, miR-224, miR-335	14/23
32	CD82, TMPRSS4, ETV4, ANXA1, S100A14, S100P, ITGB4	7/25	CD82, RAB25	2/25	let-7b, miR-218, miR-25	3/6
33	AURKA, CDC20, TOP2A, PLK1, ASPM, BUB1, FOXM1, ECT2, KIF14, CCNA2, BUB1B, FBXO5, UBE2C, TK1, CENPF, TACC3, CKS2	17/57	AURKA, CDC20, MKI67, TOP2A	4/57	let-7b, miR-101, miR-106b, miR-130b, miR-146b, miR-16, miR-17, miR-18a, miR-19a, miR-20b, miR-25, miR-29a, miR-93	13/31
