# Supplementary material for: A Computational Approach to Identifying Gene-microRNA Modules in Cancer
Source: PLoS Comput Biol. 2015 Jan 22;11(1):e1004042. doi: 10.1371/journal.pcbi.1004042 (PMC4303261; doi:10.1371/journal.pcbi.1004042)
Supplement: S6 Table — ‘Num’ represents the number of GBM genes (or GBM miRNAs) / the number of all genes (or all miRNAs) in a module. (PDF) [file pcbi.1004042.s013.pdf]

Table S6. Cancer genes, GBM genes and GBM miRNAs in modules.

Module ID	Cancer Genes	Num	GBM Genes	Num	GBM microRNAs	Num
1	KRAS, RAP1GDS1, CORO1C, PPP1CC	4/33	KRAS	1/33	miR-19a, miR-19b, miR-22, miR-222	4/9
2	BUB1, FBXO5, ASPM, PLK1, KIF14, MYBL2, AURKA, MCM2, FOXM1, ECT2, CDC6, BUB1B, CDC25C, STIL, CENPF, TOP2A, CCNA2, EZH2, CDC20, MAD2L1, MYBL1, TACC3, FGF9, BCOR, CDT1, BRCA1	26/98	CDC25C, BRCA1	2/98	miR-106a, miR-106b, miR-10b, miR-15b, miR-16, miR-18a, miR-18b, miR-19a, miR-19b, miR-20a, miR-20b, miR-22, miR-222, miR-25, miR-29a, miR-29c, miR-34a, miR-93	18/30
3	PRPF4B, ATM, DST, ARHGEF4, AKAP9, ATR, CHFR, PIK3R1, RAD9A, GSK3B, TOP2B, RBBP6, NFATC3, BCL6, CYLD, TSC2	16/101	ATM, PIK3R1, GSK3B, TSC2	4/101	miR-22	1/2
4	EIF2AK2, GBP1, CXCL10, TAP1, XAF1, SP110, TNFSF10	7/41		0/41	miR-146b, miR-155, miR-193a, miR-19b, miR-34a	5/7
5	FBXO5, ASPM, PLK1, KIF14, MYBL2, MCM2, FOXM1, CDK2, BUB1B, CENPF, TOP2A, EZH2, TACC3, FANCC	14/56	CDK2	1/56	miR-106b, miR-29a, miR-93	3/4
6	ENPP2, MAL, NPY	3/23		0/23	miR-137, miR-139, miR-218, miR-29c	4/12
7	AURKA, BUB1B, STIL, MAD2L1, FANCE, CCNB1, PCNA, H2AFX, TRAF4, PPP1CC, SFRS3, CECR5	12/43		0/43	miR-106a, miR-106b, miR-15b, miR-18a, miR-19a, miR-19b, miR-20a, miR-25, miR-29a, miR-93	10/13
8	CD44, PLAUR, ICAM1, FOSL2, LYN, PLA2G2A, FCGR2B, PTPN6, MAP3K8, IL4R, BIRC3, SPI1, CCR1, BTK, CTSB, CEBPB, TNFRSF1B, SERPINA1, IL1B, HCK, JUNB, SYK, WAS, TCIRG1	24/73	PLCG2	1/73	miR-146b, miR-155, miR-181a, miR-181b, miR-181c, miR-181d, miR-193a, miR-19a, miR-19b, miR-21, miR-22, miR-221, miR-222, miR-34a, miR-9, miR-93, miR-95	17/26
9	SOX4, SATB1, SOX11, HDAC2, OPCML, ELMO1, PEG3, PPP1R16B, FGF12, SOX10, OLIG2, NRXN2, PLCB1, CDK5R1, RAP2A, ERBB3	16/70	ERBB3	1/70	miR-106a, miR-10b, miR-139, miR-146b, miR-153, miR-155, miR-181a, miR-181b, miR-181d, miR-18a, miR-193a, miR-19a, miR-19b, miR-20a, miR-20b, miR-22, miR-221, miR-222, miR-342, miR-34a, miR-7, miR-9, miR-93	23/43
10	PLK1, AURKA, MCM2, CDK2, BUB1B, STIL, CDKN3, CCNA2, SKP2, FEN1, FANCG, GMPS, CDT1, MSH6, LYN, MAPK14, CUL4B, NFKB1, PFN1, RAB8A, NONO	21/73	CDK2, MSH6	2/73	miR-106a, miR-106b, miR-15b, miR-18a, miR-19a, miR-19b, miR-20a, miR-20b, miR-22, miR-25, miR-29a, miR-29c, miR-93	13/19
11	TACC1, CBLC, FGF14, FGF2, CFLAR, BCL2L1, CDKN1A, SHC1, AR, DDB2, ERBB2, CD46, FGFR4, MCL1, RELB, SP110, PXN, TIAM1, RANBP2, MYD88, MCC, JUN, TNFRSF1A, LYL1, MAPK1, CD151, BAX	27/89	CDKN1A, SHC1, ERBB2, MAPK1	4/89	miR-155, miR-21, miR-221, miR-222, miR-34a	5/7
12	BGN, COL1A1, TBX2, CHFR, TAGLN, CD248, COL1A2, PDGFRB, MYH9, NOTCH3	10/47		0/47	miR-143, miR-145	2/7

13	BUB1, FBXO5, BAK1, STIL, USP39, PCNA, BRMS1, CDK5, MEF2B, PHB, FANCF, ERCC8, RHOC, SMAD4, SFRS3, DHX9, CECR5	17/91		0/91	miR-106a, miR-106b, miR-15b, miR-19a, miR-19b, miR-20a, miR-93	7/9
14	ADAM12, VEGFA, CAV1, GRN, ANG, LOX, SHC1, PLAUR, TIMP1, TPM4, THBS1, CTSL1, IL4R, TGFBI, CTSB, HSPA5	16/65	SHC1	1/65	miR-139, miR-146b, miR-155, miR-181a, miR-181c, miR-181d, miR-21, miR-22, miR-221, miR-222, miR-34a, miR-9, miR-95	13/25
15	TFAP2A, ASPM, PLK1, KIF14, MYBL2, FOXM1, ECT2, CDC6, BUB1B, BRCA2, UBE2C, IGFBP7, CENPF, EEF1A2, TOP2A, SORBS2, E2F1, CDC20, MAD2L1, MYBL1, TACC3, SKP2, HSPB1, SATB1, E2F3, CDC25A, H2AFX, SYNE1, CDT1, ANG, SOX11, FGF14, RHOA, HDAC2, SLC38A1, HOXA7, PDGFRA, SFPQ, HERPUD1, NTRK3, HNRNPA2B1, SLC9A3R1, SIX1, TRAF4, RAP1GAP, OPCML, ELMO1, CUL1, ASL, PEG3, RAP1A, SMARCB1, NOTCH1, CAMKK2, CCND1, EVL, CSPG4, LRP5, COTL1, SOX10, LYL1, CHIC2, OLIG2, NRXN2, NKX2-2	65/231	BRCA2, E2F1, CDC25A, PDGFRA, CCND1, PIK3CG	6/231	miR-106a, miR-106b, miR-19a, miR-19b, miR-20a, miR-22, miR-222, miR-29a, miR-93	9/12
16	BUB1, ASPM, PLK1, KIF14, MYBL2, AURKA, CDC6, CDK2, BUB1B, BRCA2, CDC25C, STIL, TK1, CCNA2, CDC20, CHEK2, SKP2, CDC25A, FEN1, CDT1, BRCA1	21/93	CDK2, BRCA2, CDC25C, CHEK2, CDC25A, BRCA1	6/93	miR-106a, miR-106b, miR-15b, miR-18a, miR-19a, miR-19b, miR-20a, miR-25, miR-29a, miR-29c, miR-93	11/15
17	BGN, LOXL2, ADAM12, MMP9, COL1A1, LOX, TAGLN, CD248, TPM4, COL1A2, THBS1, PDGFRB, MYH9, TGFBI	14/59		0/59	miR-101, miR-143, miR-145, miR-155, miR-181a, miR-181c, miR-181d, miR-21, miR-222, miR-30c, miR-9, miR-95	12/25
18	SATB1, THRA, ARHGEF4, CREBBP, ENAH, SIRT1, PGF, SH3GL2, CTNND2, NTRK2, NRXN2, RAP2A	12/34		0/34	miR-155, miR-221, miR-222	3/4
19	EPHB6, NPY, SH3GL2, CHGA, KIAA0182	5/48		0/48	miR-139, miR-218, miR-7	3/6
20	EPHB6, NPY, PPP1R16B, CHGA, TAC1, ICAM5	6/74		0/74	miR-137, miR-139, miR-155, miR-218, miR-29c, miR-381, miR-7	7/31
21	LOXL2, ADAM12, GRN, LOX, CDKN1A, SHC1, PLAUR, ICAM1, FOSL2, MCL1, TPM4, ADAM9, CTNNA1, SWAP70, TGFBI, HSPA5	16/70	CDKN1A, SHC1	2/70	miR-101, miR-139, miR-146b, miR-155, miR-181a, miR-181b, miR-181c, miR-181d, miR-21, miR-22, miR-221, miR-222, miR-34a, miR-9, miR-95	15/34
22	COL1A1, GRN, RAD52, MERTK, VAV1, CSTA, PLAUR, SIPA1, MAPK13, TIMP1, DAB2, HERPUD1, FXYD5, CSF1, TRAF1, MPP1, MAP3K8, RPS6KA1, MYD88, SPP1, CTSL1, CD53, IL4R, PRKCD, SLC16A3, IFI30, CCR1, ERC2, CTSB, CEBPB, TNFRSF1B, FGR, SERPINA1, HCK, PSAP, ALOX5, SYK, CSF1R, TCIRG1	39/166	RPS6KA1, PRKCD, PLCG2	3/166	miR-106a, miR-106b, miR-146b, miR-155, miR-181a, miR-181b, miR-181c, miR-181d, miR-185, miR-193a, miR-19a, miR-19b, miR-20a, miR-21, miR-22, miR-221, miR-222, miR-25, miR-34a, miR-9, miR-93, miR-95	22/32

23	FGF9, ELMO1, SH3GL2, CHGA, BCL11A, SCN3B	6/34		0/34	miR-139, miR-7	2/4
24	EML4, GRN, TGFBR2, FLI1, NFKB1, TGFBR1, MPP1, JAK2, PICALM, PRKCD, LCP1, HCK	12/38	PRKCD	1/38	miR-146b, miR-155, miR-181a, miR-181c, miR-181d, miR-21, miR-22, miR-221, miR-222, miR-34a, miR-9	11/15
25	VEGFA, CD44, STAT3, LOX, PLAUR, ICAM1, FOSL2, TPM4, CLIC4, THBS1, PLA2G2A, FCGR2B, MPP1, CTSL1, FLII, BIRC3, SLC16A3, TGFBI, TGFB2, ZFP36L1, ZFP36L2, CTSB, TNFRSF1B, BCL6, KLF6, JUNB	26/75		0/75	miR-106a, miR-146b, miR-153, miR-155, miR-181a, miR-181b, miR-181c, miR-181d, miR-193a, miR-19a, miR-19b, miR-20b, miR-21, miR-22, miR-221, miR-222, miR-34a, miR-9, miR-93, miR-95	20/35
26	DBN1, SATB1, THRA, PAWR, CLDN4, SRGAP3, PIK3R1, ICAM1, MXI1, MAP2, NTRK3, BZRAP1, DLGAP1, HIPK2, DAPK1, CTNND2, CEACAM1, NOTCH1, CHD9, APC, HDAC4, TCF12, SPINT1, OLIG2, NRXN2, CDK5R1, RAP2A, NKX2-2	28/119	SHC2, PIK3R1, HIPK2	3/119	miR-155, miR-181a, miR-181c, miR-181d, miR-193a, miR-21, miR-22, miR-222, miR-9, miR-95	10/17
27	ASPM, AURKA, MCM2, FOXM1, BUB1B, CDC25C, UBE2C, CENPF, TOP2A, CCNA2, CDC20, MAD2L1, CCNB1, PTTG1	14/53	CDC25C	1/53	miR-106b, miR-15b, miR-29a, miR-93	4/6
28	GRN, RAP2B, CAPG, CSTA, PLAUR, SIPA1, VAMP8, GNAI2, FXYD5, FCGR2B, IFI30, RHOG, CTSB, TNFRSF1B, SERPINA1, HCK, TGFB1, SYK, TCIRG1	19/55		0/55	miR-21, miR-34a	2/5
29	MCM2, BUB1B, CDKN3, EZH2, PCNA, TCF3, CDKN2C, SFRS3	8/30	CDKN2C	1/30	miR-106b, miR-15b, miR-19b, miR-29a, miR-93	5/7
30	GRN, CAPG, VAV1, CSTA, SERPINB1, VAMP8, DAB2, LYN, CORO1A, FXYD5, CASP1, FCGR2B, PTPN6, CD74, CTSL1, MAFB, CD53, IFI30, SPI1, CCR1, LCP1, CTSB, TNFRSF1B, SERPINA1, HCK, ALOX5, LAPTM5, SYK, CSF1R, C3AR1	30/131	PLCG2	1/131	miR-146b, miR-155, miR-181a, miR-181c, miR-181d, miR-193a, miR-19b, miR-21, miR-22, miR-222, miR-34a, miR-9	12/19
31	ANG, ANXA1, RRAS, CAPG, SERPINB1, LYN, CASP1, ASL, RHOG, SERPINA1	10/37		0/37	miR-155, miR-21, miR-22, miR-222	4/5
32	WHSC1, DEK, DNMT1, EWSR1, TOP1, MSH3, NIPBL, NFIC, HNRNPA2B1, RBBP6, TPR, RANBP2, STAG1, SMARCA4, EIF3A, NCOA6, AKAP13	17/71	RPS6KB1, PDPK1	2/71	miR-19a, miR-22, miR-29a	3/3
33	LGALS3, VAV3, OSBPL3, IGFBP2, ANG, ANXA1, CSTA, DDB2, PLAUR, PDGFA, TIMP1, CTNNA1, SWAP70, S100A13, TNFRSF1A, MSN, CD151	17/83	PDGFA	1/83	miR-106a, miR-139, miR-146b, miR-148a, miR-155, miR-181a, miR-181b, miR-181c, miR-181d, miR-193a, miR-19a, miR-19b, miR-20a, miR-20b, miR-21, miR-22, miR-221, miR-222, miR-342, miR-34a, miR-9, miR-93	22/40
34	DNMT1, EWSR1, ATF7IP, TOP1, NIPBL, RBBP6, EP400, STAG1	8/27		0/27	miR-19a, miR-19b, miR-22, miR-29a	4/4
35	ADAM12, VEGFA, IGFBP2, PLAUR, IGFBP3, CD151, HSPA5	7/44		0/44	miR-101, miR-139, miR-155, miR-181c, miR-21, miR-221, miR-222, miR-342, miR-34a, miR-491	10/25

36	FANCE, RB1CC1, BMI1, PPM1D, TOPORS, SIRT1, CDKN1B	7/81	CDKN1B	1/81	miR-106a, miR-181d, miR-19a, miR-19b, miR-20a, miR-20b, miR-22, miR-222, miR-9, miR-93	10/16
37	BUB1, ASPM, KIF14, ECT2, CDC6, CDK2, BUB1B, STIL, CENPF, CCNA2, EZH2, MAD2L1, BRCA1	13/48	CDK2, BRCA1	2/48	miR-106a, miR-106b, miR-15b, miR-18a, miR-19a, miR-19b, miR-20a, miR-25, miR-29a, miR-29c, miR-93	11/14
38	SOX11, MAP2, CTNND2, APC, S100B, NKX2-2	6/52		0/52	miR-181a, miR-181d, miR-21, miR-9	4/6
39	THRA, SRGAP3, MAPK10, APC2, NTRK3, HIPK2, CTNND2, NCOA1, NRXN2, CDK5R1	10/42	HIPK2	1/42	miR-139, miR-155, miR-181a, miR-181c, miR-181d, miR-193a, miR-21, miR-22, miR-222, miR-9, miR-95	11/21
40	BUB1, ASPM, PLK1, MCM2, CDK2, BUB1B, BRCA2, WHSC1, CENPF, TOP2A, EZH2, TACC3, SKP2, SOX4, FEN1, CDT1, DEK, DNMT1, BRCA1, HDAC2, SFPQ, MYB, FANCC	23/84	CDK2, BRCA2, BRCA1	3/84	miR-29a, miR-93	2/3
41	LOX, CSTA, PLAUR, FOSL2, MAPK13, TIMP1, DAB2, PLA2G2A, MAP3K8, S100A8, CTSL1, MAFB, IL4R, BIRC3, SLC16A3, TGFBI, IL6, CTSB, CEBPB, TNFRSF1B, TNFAIP3, IER3, ALOX5, JUNB	24/68		0/68	miR-146b, miR-155, miR-181a, miR-181b, miR-181c, miR-181d, miR-193a, miR-19b, miR-21, miR-22, miR-221, miR-222, miR-34a, miR-9, miR-95	15/27
42	LGALS3, CD44, IGFBP2, ANG, ANXA1, CAPG, PLAUR, TIMP1, S100A13, MSN	10/36		0/36	miR-155, miR-222	2/2
43	FBXO5, ASPM, AURKA, MCM2, ECT2, BUB1B, UBE2C, STIL, CENPF, CDKN3, TOP2A, EZH2, CDC20, MAD2L1, CCNB1, PTTG1, PCNA, CKS1B, CKS2	19/54		0/54	miR-106b, miR-15b, miR-18a, miR-19b, miR-25, miR-29a, miR-93	7/9
44	PRCC, CSNK2A1, JAG1, ZFP64, ELAVL1, AATF, TH1L, MAD1L1, NONO, TSC2	10/43	TSC2	1/43	miR-20a, miR-93	2/4
45	HIP1, DNMT1, EWSR1, XPC, TOP1, GNA11, NIPBL, YES1, CSNK1A1, NBR1, TOP2B, RBBP6, TPR, AATF, ANP32B, STAG1, ERCC5, NCOA6, AKT1, AKAP13, TRIM27	21/95	AKT1	1/95	miR-22, miR-29a	2/2
46	USP39, BCOR, PRPF4B, GSPT1, ANP32A, C11orf30, SUZ12, FUS, TSC2	9/60	TSC2	1/60	miR-19a, miR-19b, miR-22, miR-29a	4/5
47	CSNK2A1, EIF4G1, ATF7IP, ABL1, TOP1, GNA11, NFIC, HNRNPA2B1, AATF, BRD4, SMARCA4, NCOA6, AKAP13, KEAP1	14/59		0/59	miR-22, miR-29a	2/2
48	ANG, ANXA1, S100A10, RRAS, CAPG, CSTA, SERPINB1, YAP1, VAMP8, LYN, CASP1, S100A13, CCR1, RHOG	14/68		0/68	miR-146b, miR-155, miR-21, miR-22, miR-222, miR-34a	6/9
49	MPP1, SPI1, CCR1, CSF3R, HCK, SYK, CSF1R	7/41		0/41	miR-21, miR-22, miR-34a	3/6
50	DBN1, SOX11, MAP2, TOP2B, DL-GAP1, ASCL1, TRIM37, OLIG2, TCL1A, NKX2-2	10/49		0/49	miR-155, miR-181a, miR-181b, miR-181d, miR-193a, miR-20a, miR-20b, miR-21, miR-22, miR-222, miR-9, miR-93	12/17

51	CAPG, CSTA, SERPINB1, VAMP8, LYN, CORO1A, CASP1, RPS6KA1, RAC2, MAFB, CD53, BCL2A1, CCR1, ERC2, CEBPA, SERPINA1, HCK, ALOX5, CD69, LAPTM5, SYK, CSF1R, C3AR1	23/88	RPS6KA1, PIK3CG	2/88	miR-146b, miR-155, miR-181a, miR-181d, miR-193a, miR-19b, miR-21, miR-22, miR-222, miR-34a, miR-9	11/18
52	TFAP2A, SOX11, YAP1, SFPQ, HIST1H1C, OPCML, FGF12, SOX10, PLCB1, CDK5R1, RAP2A	11/57		0/57	miR-10b, miR-137, miR-139, miR-155, miR-181a, miR-221, miR-222, miR-34a, miR-7	9/21
53	THRA, NDRG2, MAP2, ASCL1, CTNND2, DNAJC6, NTRK2, OLIG2, NRXN2, NKX2-2	10/39		0/39	miR-9	1/2
54	GATA6, TGFBR2, TEK, MME, CITED2, CSF3R, TNFSF10	7/35		0/35	miR-155, miR-181a, miR-181d, miR-21, miR-22, miR-222, miR-9	7/11
