# Supplementary material for: A Computational Approach to Identifying Gene-microRNA Modules in Cancer
Source: PLoS Comput Biol. 2015 Jan 22;11(1):e1004042. doi: 10.1371/journal.pcbi.1004042 (PMC4303261; doi:10.1371/journal.pcbi.1004042)
Supplement: S7 Table — (PDF) [file pcbi.1004042.s014.pdf]

Table S7. Exprementally validated gene-miRNA interactions in ovarian cancers.

Module ID	Gene	miRNA	Validation Method	Reference
2	MMP9	miR-21	qRT-PCR	19435867
2	KLRB1	miR-335	Microarray	18185580
2	CXCL9	miR-335	Microarray	18185580
2	EPB41L3	miR-223	Luciferase reporter assay, Western blot	21628394
2	CD96	miR-335	Microarray	18185580
2	CD27	miR-335	Microarray	18185580
2	MEF2C	miR-223	Luciferase reporter assay	18278031
2	MEF2C	miR-223	Immunofluorescence, qRT-PCR, Western blot	23094093
2	MEF2C	miR-21	Immunofluorescence, In situ hybridization, Luciferase reporter assay	21170291
2	LSP1	miR-335	Microarray	18185580
2	ITGB2	miR-146a	Microarray	18057241
2	ITGB2	miR-335	Microarray	18185580
2	PLAUR	miR-155	Proteomics	18668040
2	LCK	miR-335	Microarray	18185580
2	SPP1	miR-146a	qRT-PCR	20110513
2	SPP1	miR-335	Microarray	18185580
3	CCNE2	miR-25	CLASH	23622248
3	GINS1	miR-18a	CLASH	23622248
3	GINS1	miR-7	Microarray	19073608
3	GPR19	miR-7	Microarray	17612493
3	E2F1	miR-93	Luciferase reporter assay, Western blot	19486339
3	E2F1	miR-93	Luciferase reporter assay, Microarray, Western blot	18328430
3	E2F1	miR-93	Sequencing	20371350
3	E2F1	miR-106b	Luciferase reporter assay, qRT-PCR	18676839
3	E2F1	miR-106b	Luciferase reporter assay, Western blot	19486339
3	E2F1	miR-106b	Luciferase reporter assay, Microarray, Western blot	18328430
3	E2F1	miR-106b	Luciferase reporter assay	21283765
3	E2F1	miR-106b	Reporter assay	18212054
3	E2F1	miR-130b	Sequencing	20371350
3	KIF22	miR-25	CLASH	23622248
3	ARL6IP1	miR-130b	Sequencing	20371350
3	MXD3	miR-18a	CLASH	23622248
3	EZH2	miR-93	CLASH	23622248
3	EZH2	miR-25	Luciferase reporter assay, qRT-PCR, Western blot	22399519
3	MCM7	miR-93	CLASH	23622248
5	CREBZF	miR-221	Reporter assay, Microarray	20018759
5	PEX19	miR-221	CLASH	23622248
6	CHEK1	miR-15b	CLASH	23622248
6	MKI67	miR-29b	CLASH	23622248
6	MKI67	miR-17	CLASH	23622248
6	CDC7	miR-101	Sequencing	20371350
6	CCNE2	miR-25	CLASH	23622248
6	CCNE2	miR-26a	Luciferase reporter assay, Western blot	19524505
6	LMNB1	miR-101	Sequencing	20371350
6	CDC25A	miR-18a	CLASH	23622248
6	MSH6	miR-106b	CLASH	23622248
6	MSH6	miR-26a	CLASH	23622248
6	PCNA	miR-18a	CLASH	23622248
6	KIF23	miR-25	CLASH	23622248
6	KIF23	miR-106b	Microarray	17242205
7	FLT4	miR-331	CLASH	23622248
9	PPIE	miR-324	CLASH	23622248
10	NCAPD2	let-7b	CLASH	23622248
11	UBE2I	let-7b	Proteomics	18668040
12	NUP153	miR-93	CLASH	23622248
13	CCNE2	miR-25	CLASH	23622248

13	ACOT7	miR-18a	CLASH	23622248
13	RIF1	miR-93	CLASH	23622248
13	CDC42	miR-93	CLASH	23622248
13	CDC42	miR-29a	Luciferase reporter assay, Western blot	19079265
13	MCM7	miR-93	CLASH	23622248
13	DDX49	miR-25	CLASH	23622248
13	BIRC5	miR-93	CLASH	23622248
13	PCNA	miR-18a	CLASH	23622248
13	CBX3	miR-93	CLASH	23622248
13	PSMB4	miR-25	CLASH	23622248
14	CAMTA1	miR-218	Sequencing	20371350
14	DBN1	miR-149	CLASH	23622248
14	CEP170	miR-125b	CLASH	23622248
14	BCL3	miR-125b	Luciferase reporter assay	20658525
14	HK2	miR-125b	Luciferase reporter assay, qRT-PCR	22593586
15	NDRG1	let-7b	CLASH	23622248
15	TUBB	let-7b	CLASH	23622248
15	TUBB	miR-20a	CLASH	23622248
15	SP100	let-7b	Proteomics	18668040
17	DYRK2	miR-93	Sequencing	20371350
17	DYRK2	miR-93	CLASH	23622248
17	MARS	let-7b	Proteomics	18668040
17	NUP107	miR-93	Sequencing	20371350
17	RRAGC	miR-29a	CLASH	23622248
17	NCAPD2	let-7b	CLASH	23622248
17	NCAPD2	miR-93	CLASH	23622248
18	CHEK1	miR-15b	CLASH	23622248
18	CCNE2	miR-25	CLASH	23622248
18	RNGTT	miR-25	CLASH	23622248
18	MCM3	miR-93	CLASH	23622248
18	NASP	miR-29a	Luciferase reporter assay, Western blot	22080513
18	NASP	miR-29a	Luciferase reporter assay	22194605
18	NASP	miR-93	CLASH	23622248
18	KIF23	miR-25	CLASH	23622248
18	MCM7	miR-93	CLASH	23622248
19	VPS39	let-7b	pSILAC, Proteomics, Other	18668040
20	DEPDC1	miR-17	CLASH	23622248
20	ASF1B	miR-18a	CLASH	23622248
20	MAD2L1	miR-93	CLASH	23622248
20	BIRC5	miR-93	CLASH	23622248
20	BIRC5	miR-101	Sequencing	20371350
20	TOP2A	miR-7	CLASH	23622248
20	CDC20	miR-18a	CLASH	23622248
20	CDC20	miR-93	CLASH	23622248
21	CCR1	miR-21	Luciferase reporter assay	21131358
23	FBXO28	miR-17	CLASH	23622248
23	SNRPE	let-7b	CLASH	23622248
23	ARID4B	miR-17	CLASH	23622248
25	CHEK1	miR-15b	CLASH	23622248
25	CCNE2	miR-25	CLASH	23622248
25	PCNA	miR-18a	CLASH	23622248
25	MCM7	miR-93	CLASH	23622248
25	KPNA2	miR-93	CLASH	23622248
25	KIF23	miR-25	CLASH	23622248
26	DEPDC1	miR-17	CLASH	23622248
26	PLK1	let-7b	CLASH	23622248
26	BIRC5	miR-93	CLASH	23622248
26	BIRC5	miR-101	Sequencing	20371350
26	BIRC5	let-7b	CLASH	23622248

26	CCNA2	let-7b	Immunoblot, Immunofluorescence, Luciferase reporter assay, qRT-PCR	18379589
26	MAD2L1	miR-93	CLASH	23622248
26	FEN1	let-7b	CLASH	23622248
26	PCNA	miR-18a	CLASH	23622248
26	CCNB1	let-7b	CLASH	23622248
26	KPNA2	miR-93	CLASH	23622248
26	KPNA2	miR-17	CLASH	23622248
26	MCM4	let-7b	CLASH	23622248
26	CHEK1	miR-15b	CLASH	23622248
26	HMMR	miR-93	CLASH	23622248
26	RAN	miR-29a	flow, qRT-PCR, GFP reporter assay	19818597
26	CDC7	miR-101	Sequencing	20371350
27	TWIST1	miR-214	Luciferase reporter assay, qRT-PCR, Western blot	22540680
27	MMP14	miR-145	Reporter assay, Microarray	21351259
29	PPIE	miR-324	CLASH	23622248
29	MYCBP	miR-93	CLASH	23622248
30	WDR46	miR-324	CLASH	23622248
30	EEF1E1	let-7b	Proteomics	18668040
30	TBCC	miR-93	CLASH	23622248
30	RXRB	let-7b	CLASH	23622248
30	PFDN6	miR-106b	CLASH	23622248
31	TRIM22	miR-146a	Microarray	18057241
31	TRIM22	miR-335	Microarray	18185580
31	STAT1	miR-146a	Microarray	20110513
31	OAS2	miR-335	Microarray	18185580
31	SERPINF1	miR-335	Microarray	18185580
31	STAT3	miR-155	Proteomics	18668040
31	STAT3	miR-20a	CLASH	23622248
31	STAT3	miR-21	Western blot, Other	20048743
31	STAT3	miR-21	Microarray	18591254
31	STAT3	miR-125b	Microarray	17891175
31	STAT3	miR-20b	qRT-PCR, ELISA, ChIP, Western blot	20232316
31	OAS1	miR-335	Microarray	18185580
31	AIM1	miR-21	Microarray	18591254
31	ISG15	miR-146a	Microarray	18057241
31	CTSD	miR-335	Microarray	18185580
31	OASL	miR-146a	Microarray	18057241
31	SPP1	miR-146a	qRT-PCR	20110513
31	SPP1	miR-335	Microarray	18185580
31	PTAFR	miR-335	Microarray	18185580
31	EPB41L3	miR-223	Luciferase reporter assay, Western blot	21628394
31	TNFSF10	miR-222	Western blot	18246122
31	IRF2	miR-335	Microarray	18185580
31	ACVR2B	miR-335	Microarray	18185580
31	ACVR2B	miR-140	Sequencing	20371350
31	LAPTM5	miR-335	Microarray	18185580
31	C1QA	miR-335	Microarray	18185580
31	ITGB2	miR-146a	Microarray	18057241
31	ITGB2	miR-335	Microarray	18185580
31	LYN	miR-222	CLASH	23622248
31	CXCL9	miR-335	Microarray	18185580
32	TOB1	miR-218	Luciferase reporter assay	23060446
33	NCAPG	miR-16	Proteomics	18668040
33	MKI67	miR-29b	CLASH	23622248
33	MKI67	miR-17	CLASH	23622248
33	MKI67	miR-16	Proteomics	18668040
33	KIFC1	let-7b	CLASH	23622248
33	DEPDC1	miR-17	CLASH	23622248
33	PLK1	miR-16	pSILAC, Proteomics, Other	18668040

33	PLK1	let-7b	CLASH	23622248
33	CEP55	miR-16	Sequencing	20371350
33	KIF14	miR-16	Proteomics	18668040
33	AURKA	let-7b	Microarray	17699775
33	AURKA	let-7b	Proteomics	18668040
33	RACGAP1	miR-16	Proteomics	18668040
33	KIF23	miR-16	Proteomics	18668040
33	KIF23	miR-25	CLASH	23622248
33	KIF23	miR-106b	Microarray	17242205
33	OIP5	miR-16	Sequencing	20371350
33	OIP5	miR-29c	Sequencing	20371350
33	UBE2C	miR-17	CLASH	23622248
33	UBE2C	miR-16	Proteomics	18668040
33	CENPF	miR-93	CLASH	23622248
33	CENPF	miR-16	Proteomics	18668040
33	NUSAP1	let-7b	CLASH	23622248
33	TOP2A	miR-7	CLASH	23622248
33	CCNA2	let-7b	Immunoblot, Immunofluorescence, Luciferase reporter assay, qRT-PCR	18379589
33	CDC20	miR-18a	CLASH	23622248
33	CDC20	miR-93	CLASH	23622248
33	CDC20	miR-16	Proteomics	18668040
33	AURKB	miR-16	Microarray, qRT-PCR	19738602
33	AURKB	miR-16	Proteomics	18668040
33	AURKB	let-7b	pSILAC, Proteomics, Other	18668040
33	TACC3	miR-18a	CLASH	23622248
33	RRM2	miR-26a	CLASH	23622248
33	RRM2	miR-425	CLASH	23622248
33	RRM2	let-7b	Microarray	17699775
33	RRM2	let-7b	Proteomics	18668040
33	CKS2	let-7b	CLASH	23622248
33	CCNB2	let-7b	CLASH	23622248
33	CDCA8	miR-16	Proteomics	18668040
33	CDCA8	let-7b	Proteomics	18668040
33	BIRC5	miR-93	CLASH	23622248
33	BIRC5	miR-101	Sequencing	20371350
33	BIRC5	miR-425	Sequencing	20371350
33	BIRC5	let-7b	CLASH	23622248
33	KPNA2	miR-93	CLASH	23622248
33	KPNA2	miR-17	CLASH	23622248
33	KPNA2	miR-16	Proteomics	18668040
