# Supplementary material for: A Computational Approach to Identifying Gene-microRNA Modules in Cancer
Source: PLoS Comput Biol. 2015 Jan 22;11(1):e1004042. doi: 10.1371/journal.pcbi.1004042 (PMC4303261; doi:10.1371/journal.pcbi.1004042)
Supplement: S8 Table — (PDF) [file pcbi.1004042.s015.pdf]

Table S8. Genes and miRNAs are co-regulated by the same TF in ovarian cancer modules.

Module ID	TF	miRNA	Pubmed ID	Genes	p-value
1	EGR1	miR-23b	20811575	BOP1, EXOSC4, FBXL6, GRINA, HSF1, LRRC14, OPLAH, PTK2, PYCRL, SCRIB, SHARPIN, ZC3H3, ZNF16, ZNF250	1.10E-05
1	MYC	miR-23b<br>miR-30b<br>miR-30d<br>miR-378	19219026<br>18066065<br>18066065<br>21242960	C8ORF55, CYC1, GRINA, ZC3H3, ZNF250, ZNF696	1.49E-05
2	MITF	miR-146a	21603362	ACP5, CD44, CD74, CXCL13, DOCK2, DPYD, EPB41L3, FGR, GZMH, HCK, ITGB2, LCP2, MEF2C, SIGLEC1, SNX10, SPP1, VAV1	1.69E-02
2	MYC	miR-29b<br>miR-29c	20564213<br>20564213	CLEC5A, CXCL11, CXCL9, DPYD, FGR, ITGB2, LCK, LSP1, MMP9, TYROBP, VAV1	1.63E-02
3	E2F1	miR-15b<br>miR-18a<br>miR-25<br>miR-7	21454377<br>23396440<br>22431589<br>22492316	CDC6, EZH2, GINS1, GINS2, IQCK, MCM2, MCM7, MXD3, POLA2	2.46E-06
5	RUNX1	miR-221	23344057	ATP5D, C1ORF61, CREBZF, DFFB, MRPL40, PPP1R14B, PTK2, PYCRL, RANBP1, SCRIB	1.20E-02
6	E2F1	miR-15b<br>miR-20b<br>miR-25<br>miR-7	21454377<br>23396440<br>22431589<br>22492316	CBX5, CDC25A, CDC7, CHEK1, DONSON, LMNB1, MCM2, MCM6, MSH6, PCNA, POLE2, TIMELESS	9.30E-10
6	MYCN	miR-17	17943719	CDC25A, CDC7, CHEK1, FEN1, KIF4A, KNTC1, TIMELESS	1.08E-07
7	EGR1	miR-23b	20811575	BOP1, CPSF1, FBXL6, FLT4, HSF1, PLEC1, PRKCE, PTK2, PYCRL, RECQL4, SCRIB, SHARPIN, ZNF250	6.76E-05
8	EGR1	miR-152<br>miR-199a<br>miR-199b<br>miR-214	20811575<br>21189327<br>20811575<br>21189327	AQP1, BGN, CALB2, CEND1, COL1A1, COMP, HNT, IRX5, ITGA5, ITGB1, LGALS1, MMP11, PDGFRB, PLAUR, POU2F3, SERPINE1, TGFB1I1, THBS1, THBS2	2.51E-05
13	E2F1	miR-18a<br>miR-25<br>miR-7	23396443<br>22431589<br>22492316	ARNTL2, AURKB, BUB3, CDC6, CHEK1, EXO1, FANCG, GINS2, GMNN, MCM6, MCM7, NUP155, PCNA, PEX10, POLE2, RPP30, SMARCB1, TIMELESS, TMPO	2.20E-11
18	E2F1	miR-15b<br>miR-18a<br>miR-25	21454377<br>23396444<br>22431589	AURKB, CASP8AP2, CDC7, CHEK1, DEK, DONSON, EXO1, FANCG, MCM2, MCM3, MCM6, MCM7, NASP, TIMELESS, TOPBP1	0.00E+00
19	MITF	let-7b	21603362	CTSB, ETV6, KIF13B, LMNA, PRKCD, RAD54L2, RHBDF2, TFEB	4.98E-02
19	RUNX1	miR-222	23344057	C1ORF116, CAPN1, CD82, ETV6, KIF13B, LMNA, MAPKAPK2, RAD54L2, SMARCA2	9.77E-03
20	E2F1	miR-15b<br>miR-18a<br>miR-7	21454377<br>23396440<br>22492316	AURKB, CENPA, E2F8, EXO1, EXOSC9, GINS2, HMGB2	6.86E-04
20	MYCN	miR-17	17943719	AURKB, CDCA3, CDCA8, FOXM1, KIF4A, MAD2L1, RAD51AP1	5.93E-07
22	EGR1	miR-152	20811575	AEBP1, COL1A1, COL5A1, COL5A3, COL6A1, ITGA5, LOXL2, MMP11, MMP2, THBS2	2.13E-02
22	EGR1	miR-214	21189327	AEBP1, COL1A1, COL5A1, COL5A3, COL6A1, ITGA5, LOXL2, MMP11, MMP2, THBS2	2.13E-02
25	E2F1	miR-15b<br>miR-18a<br>miR-25<br>miR-7	21454377<br>23396446<br>22431589<br>22492316	CDC7, CHEK1, EXO1, FBXO5, KIAA0101, KPNA2, MCM2, MCM6, MCM7, PCNA, POLE2, TIMELESS, TIPIN	5.00E-12
26	E2F1	miR-15b<br>miR-18a<br>miR-25<br>miR-7	21454377<br>23396447<br>22431589<br>22492316	CDC7, CENPA, CHEK1, EXOSC9, FBXO5, GINS2, GMNN, H2AFZ, KPNA2, PCNA, RPA3, SLC25A11, TIMELESS	2.29E-09

27	EGR1	miR-152<br>miR-199a<br>miR-199b<br>miR-214	20811575<br>21189327<br>20811575<br>21189327	AEBP1, BGN, C10ORF56, C7ORF10, COL1A1, COL5A1, COMP, FSTL1, HNT, LAMB1, LOXL2, MMP11, MMP2, PDGFRB, TAGLN, TGFB1I1, THBS1, THBS2, TMEM158, TWIST1, XYLT1	6.88E-04
27	NANOG	miR-22	18079172	MMP2, SPARC, SRPX2, THBS1, THBS2, TNFAIP6, TWIST1	2.23E-02
28	MYC	miR-23a<br>miR-27a	19219026<br>23649631	AKAP8, AKAP8L, HMGA1, ILF3, SLC35E1, SMARCA4, TMEM161A	2.79E-05
31	IRF8	miR-155	23166356	KLF12, OAS1, OAS3, PSMB10, PSMB9, SLC15A3, SP100	2.00E-07
31	MITF	miR-146a	21603362	BAZ1A, CAPG, CTSB, CTSD, CTSL1, DBI, DPYD, EPB41L3, EYA2, HERC5, IRF9, ITGB2, KLF12, LAPTM5, LCP2, OAS2, PTAFR, RAB25, RAB27A, S100A13, SERPINF1, SIGLEC1, SPP1, STAT1, STAT3, STOM, TACC1	2.91E-06
31	MYC	miR-29b<br>miR-29c	20564213<br>18066065	CD82, CXCL11, CXCL9, DPYD, ISG15, ITGB2, LAPTM5, MAPK8IP1, OAS2, RAB25, RFXAP, SMARCA2, STAT1, TACC1, TNFSF10,	6.13E-04
31	RUNX1	miR-222<br>miR-221	23344057<br>23344057	C1ORF116, CAPN1, CD82, PLEKHF2, PLLP, RAB11FIP1, REEP5, S100A4	3.02E-02
33	E2F1	miR-15b<br>miR-16<br>miR-18a<br>miR-20b<br>miR-25<br>miR-7	21454377<br>21454377<br>23396448<br>23396440<br>22431589<br>22492316	AURKB, CENPA, DLG7, EXO1, FBXO5, KPNA2, OIP5, PBK, RRM2	1.56E-04
33	MYCN	miR-17	17943719	AURKB, CDCA8, DLG7, FOXM1, KIF11, KIF4A, RAD51AP1, TROAP, TTK, UBE2C	1.10E-09
