# Supplementary material for: A Computational Approach to Identifying Gene-microRNA Modules in Cancer
Source: PLoS Comput Biol. 2015 Jan 22;11(1):e1004042. doi: 10.1371/journal.pcbi.1004042 (PMC4303261; doi:10.1371/journal.pcbi.1004042)
Supplement: S9 Table — (PDF) [file pcbi.1004042.s016.pdf]

Table S9. MiRNA regulates TFs and the TFs regulate genes in the ovarian cancer modules.

Module ID	miRNA	PubMed ID	TF	Genes	p-value
2	miR-96<br>miR-125b	17597072<br>21368288	MITF	ACP5, CD44, CD74, CXCL13, DOCK2, DPYD, EPB41L3, FGR, GZMH, HCK, ITGB2, LCP2, MEF2C, SIGLEC1, SNX10, SPP1, VAV1	1.69E-02
2	miR-17<br>miR-20a	18700987<br>18700987	RUNX1	FGR, ICOS, LCK, LCP2, PTPN22, RASSF4, SLA, TFEC	4.32E-04
3	miR-25	20036482	MITF	COQ7, E2F1, GINS1, IQCK, MCM2, PLK1, SEC61A2, SYT17, TFDP2, TRMT11, TUBG1	2.67E-02
3	miR-106b<br>miR-18a	19559694<br>23322197	STAT3	ARL6IP1, EZH2, IQCK, LRRC20, MCM2, POLA2, TUBG1	4.72E-02
6	miR-101<br>miR-29a<br>miR-29b<br>miR-29c	21654684<br>21654684<br>21654684<br>21654684	MYCN	CDC25A, CDC7, CHEK1, FEN1, KIF4A, KNTC1, TIMELESS	1.08E-07
13	miR-25	20811575	EGR1	ACOT7, AURKAIP1, AURKB, BIRC5, CBX3, CDC45L, CHEK1, DBF4, FANCE, GAD1, GINS2, HEATR3, LSM5, MCM6, MCM7, NADK, NCAPH, ORC5L, RANBP1, RIF1, SNTB1, SUB1, TFAM, TMPO, WDR8	3.98E-03
13	miR-29a	21654684	MYCN	AURKB, CDC6, CDCA8, CHEK1, KIF4A, TIMELESS	2.88E-04
13	miR-18a	19114653	RUNX1	BIRC5, BUB3, CBX3, CDC42, CDC6, CDCA8, DBF4, DDX49, EIF4EBP1, GAD1, GMNN, MCM7, PCNA, PEX10, RAD1, RANBP1, RIF1, SMARCB1, SUB1, TMPO, UCHL5	6.64E-03
15	miR-20a	19559694	STAT3	DNAH7, GPATCH2, LAMA2, RHOBTB1, SP100, TAF1A, TSN	4.72E-02
17	let-7b	23738143	E2F1	C11ORF30, CAND1, KIAA0286, MSH2, SHMT2, TIMELESS, YEATS4	5.11E-05
18	miR-29a	21654684	MYCN	AURKB, CDC7, CHEK1, FEN1, FOXM1, KNTC1, TIMELESS	5.46E-08
18	miR-18a	23322197	STAT3	CASP8AP2, DSN1, EXO1, MCM2, RACGAP1, RNGTT, WHSC1	2.58E-02
19	miR-17	20811575	EGR1	ALS2CL, CAPN1, CD82, CTSB, ETV6, IFNAR2, KIF13B, LCN2, LMNA, MAPKAPK2, PRKCD, RHBDF2, TFEB, ZDHHC18	4.07E-05
19	miR-17<br>miR-20a	18700987<br>18700987	RUNX1	C1ORF116, CAPN1, CD82, ETV6, KIF13B, LMNA, MAPKAPK2, RAD54L2, SMARCA2	9.77E-03
20	miR-101<br>miR-29a<br>miR-29b	21654684<br>21654684<br>21654684	MYCN	AURKB, CDCA3, CDCA8, FOXM1, KIF4A, MAD2L1, RAD51AP1	5.93E-07
23	miR-17	18700987	RUNX1	CAMSAP1L1, H3F3A, NSL1, NUP133, PARP1, RAB4A, RBBP5, TSNAX	4.97E-03
23	miR-17	19559694	STAT3	CAMSAP1L1, FBXO28, NUP133, RAB4A, TAF1A, TSNAX	7.27E-03
26	let-7b	22698995	BACH1	BIRC5, CCNA2, CENPA, CHEK1, H2AFZ, MCM10, MCM4, PCNA, RAP1GDS1	9.97E-06
26	let-7b	23738143	E2F1	CDC7, CENPA, CHEK1, EXOSC9, FBXO5, GINS2, GMNN, H2AFZ, KPNA2, PCNA, RPA3, SLC25A11, TIMELESS	2.29E-09
26	miR-101<br>miR-29a<br>miR-29b	21654684<br>21654684<br>21654684	MYCN	CDC7, CHEK1, FEN1, MAD2L1, TIMELESS, XRCC4	1.11E-05
26	miR-17<br>miR-18a	18700987<br>19114653	RUNX1	BIRC5, EXOSC9, FAM64A, GMNN, PCNA, RAN, RANBP1, RFC4, RIT1, RPA3, STK17B, UCHL5, XRCC4	1.34E-02
30	miR-25	20811575	EGR1	CDC5L, CSNK2B, DAXX, EHMT2, GTF2H4, PBX2, RDBP, RGL2, RXRB, TBCC, ZBTB22	3.58E-03
30	miR-20b<br>miR-25	19056895<br>20036482	MITF	CDC5L, DAXX, EHMT2, LSM2, PAK1IP1, RGL2, SNRPC, TBCC	4.98E-02
31	miR-185	23417242	AR	CAPG, EYA2, OAS1, RAB25, RAB27A, SAMSN1, SIGLEC1, SMARCA2, SQRDL, STAT1	6.90E-03

31	miR-125b	21368288	MITF	BAZ1A, CAPG, CTSB, CTSD, CTSL1, DBI, DPYD, EPB41L3, EYA2, HERC5, IRF9, ITGB2, KLF12, LAPTM5, LCP2, OAS2, PTAFR, RAB25, RAB27A, S100A13, SERPINF1, SIGLEC1, SPP1, STAT1, STAT3, STOM, TACC1	2.91E-06
31	miR-17<br>miR-20a	18700987<br>18700987	RUNX1	CAPG, CD82, CTSD, CTSL1, DBI, HERC5, IRF1, IRF2, ITGB2, KLF12, LAPTM5, LYN, RFXAP, SLA, SMARCA2, SP100, STAT1, STAT3, STOM, TACC1, TNFSF10, USP18, WASF1	6.58E-05
33	let-7b	22698995	BACH1	BIRC5, BUB1, CCNA2, CENPA, CENPF, KIF11, MCM10, OIP5, RRM2, TK1	1.06E-04
33	miR-146b<br>let-7b	23762142<br>23762142	E2F1	AURKB, CENPA, DLG7, EXO1, FBXO5, KPNA2, OIP5, PBK, RRM2	1.56E-04
33	miR-101<br>miR-29a<br>miR-29b<br>miR-29c	21654684<br>21654684<br>21654684<br>21654684	MYCN	AURKB, CDCA8, DLG7, FOXM1, KIF11, KIF4A, RAD51AP1, TROAP, TTK, UBE2C	1.10E-09
