# Supplementary material for: A Computational Approach to Identifying Gene-microRNA Modules in Cancer
Source: PLoS Comput Biol. 2015 Jan 22;11(1):e1004042. doi: 10.1371/journal.pcbi.1004042 (PMC4303261; doi:10.1371/journal.pcbi.1004042)
Supplement: S10 Table — The significant numbers of genes in each module are regulated by miRNAs, and the significances are shown in ‘p-value’. ‘m’, ‘k’, and ‘x’ represent the number of genes regulated by the miRNA collected from microCosm, the number of genes in the module, and the number of genes regulated by the miRNA in the module, respectively. (PDF) [file pcbi.1004042.s017.pdf]

Table S10. MiRNAs regulate genes in GBM modules.

Module ID	microRNA	m	k	x	p-value	Genes
8	miR-181d	612	73	9	1.13E-02	CD14, CD163, CP, FAS, MAP3K8, MMP7, NCF1, STC1, TLR1
8	miR-181b	612	73	8	3.13E-02	CD14, CD163, FAS, MAP3K8, MMP7, NCF1, SAT1, TLR1
14	miR-181d	612	65	8	1.67E-02	ABCC3, ARSJ, CD163, CHRNA9, NRP2, PLAU, SERPINE1, TGFBI
17	miR-181d	612	59	7	2.92E-02	COL6A3, LAMC3, MYH9, PLAU, SERPINE1, STC1, TGFBI
20	miR-210	469	74	7	2.48E-02	CCKBR, CRHBP, GNG3, HPCA, MYT1L, NEFM, NRGN
25	miR-181d	612	75	9	1.34E-02	CP, F13A1, FAS, FLII, KLF6, PLAU, SERPINE1, SRGN, TGFBI
33	miR-338	1067	83	13	3.01E-02	C13orf18, CAST, DDB2, FAS, IGFBP2, LDHA, PLAUR, SLC25A20, SLC2A10, SLC43A3, TAGLN2, TNFAIP6
34	miR-22	910	27	5	4.89E-02	MDC1, NOL8, PHF2, SPEN, TRRAP
36	miR-223	815	81	10	4.60E-02	BUB3, CDKN1B, CEP72, H3F3A, MBIP, PDCL, PPM1D, PSIP1, RB1CC1, STMN1
41	miR-181d	612	68	9	7.19E-03	CD14, CD163, DSE, F13A1, MAP3K8, MBD4, SRGN, STC1, TGFBI
41	miR-181b	612	68	8	2.14E-02	CD14, CD163, MAP3K8, MAPK13, MBD4, SAT1, SRGN, TGFBI
50	miR-222	838	49	8	1.88E-02	AP3B2, BCHE, DLGAP1, GRIA2, KCNQ2, RAPGEF4, SEZ6L, ZFP2
51	miR-181d	612	88	9	3.43E-02	CASP1, CD14, CD4, DOCK2, PLAC8, RAC2, SRGN, TBXAS1, TLR1
54	miR-181a	1430	35	10	6.06E-03	C1orf78, CITED2, CSF3R, IL7R, LEPR, MME, NPC2, PROCR, TGM2, ZMYM6
