# Supplementary material for: A Computational Approach to Identifying Gene-microRNA Modules in Cancer
Source: PLoS Comput Biol. 2015 Jan 22;11(1):e1004042. doi: 10.1371/journal.pcbi.1004042 (PMC4303261; doi:10.1371/journal.pcbi.1004042)
Supplement: S11 Table — (PDF) [file pcbi.1004042.s018.pdf]

Table S11. Genes and miRNAs are co-regulated by the same TF in GBM modules.

Module ID	TF	miRNA	Pubmed ID	Genes	p-value
2	MYC	miR-29c<br>miR-30a	20564213<br>18066065	BUB3, CDC7, DDX11, FBXO5, KIF23, LMNB1, MCM7, NCAPG	1.89E-03
2	MYCN	miR-152	23482921	AURKB, CCDC15, CDC6, CDC7, CDCA3, CDCA8, CHEK1, FOXM1, KIF11, KIF4A, KNTC1, MAD2L1, MSH5, RAD51AP1, TROAP, TTK, ZWILCH	0.00E+00
2	SOX2	miR-22<br>miR-29a	18079172<br>21693621	ATAD2, BUB1B, BUB3, CDC45L, CDC7, KIF11, KIF15, KIF2C, KNTC1, ORC6L, TOP2A, TUBG1	4.55E-04
2	CREB1	miR-17	21972292	BRCA1, BUB3, CCNA2, CDC45L, CENPE, EXO1, GOT1, GTSE1, HMGB2, MCM6, ORC6L, PRC1, RAD51, SFRS2	1.13E-06
2	E2F1	miR-15b<br>miR-18a	21454377<br>23396440	AP3B2, ATAD2, AURKB, BCOR, BUB3, CDC6, CDC7, CDT1, CENPM, CHEK1, DDX11, E2F8, EXO1, EZH2, FBXO5, GOT1, HMGB2, KPNA2, LMNB1, MCM2, MCM3, MCM6, MCM7, MSH5, MXD3, POLA2, SNRPA1, STIL	0.00E+00
2	MYC	miR-18a<br>miR-92	20516112<br>20516112	BUB3, CDC7, DDX11, FBXO5, KIF23, LMNB1, MCM7, NCAPG	1.89E-03
2	MYCN	miR-106a<br>miR-18a<br>miR-18b<br>miR-19a<br>miR-20a<br>miR-92<br>miR-93	17943719<br>19946337<br>19946337<br>19946337<br>19946337<br>17943719<br>17943719	AURKB, CCDC15, CDC6, CDC7, CDCA3, CDCA8, CHEK1, FOXM1, KIF11, KIF4A, KNTC1, MAD2L1, MSH5, RAD51AP1, TROAP, TTK, ZWILCH	0.00E+00
5	MYCN	miR-93	17943719	AURKB, CDCA3, CHEK1, FOXM1, KIF11, KIF4A, KNTC1, LIG1, RAD51AP1, TIMELESS, TROAP, TTK, ZWILCH	0.00E+00
7	E2F1	miR-15b<br>miR-18a	21454377<br>23396440	AURKB, BCL7C, CENPA, CENPM, DTYMK, GINS2, GMNN, ISG20L1, MXD3, OIP5, PCNA, SNRPD1, STIL	1.66E-09
8	RUNX1	miR-142<br>miR-221<br>miR-222	23509362<br>23344057<br>23344057	CCL2, CD14, CD44, CHI3L2, EMR1, FOSL2, HCK, ICAM1, IL10RA, IL4R, JUNB, LYN, MAP3K8, PLAUR, PLCG2, PTPN6, RNASE2, SLA, SLC11A1, SOD2, SPI1, STAB1, SYK, THBD, TREM1	6.00E-05
9	E2F1	miR-18a	23396441	ERBB3, FHOD3, MBP, NRXN2, PAK7, PEG3, PLCB1, PPP1R16B, RAP2A, TMSL8	3.61E-03
9	MYC	miR-18a	20516112	DGKB, FHOD3, G3BP2, MBP, SH3GL3, ST8SIA3	5.33E-03
10	MYC	miR-29c<br>miR-18a<br>miR-92	20564213<br>20516112<br>20516112	AGPS, CDKN3, GMPS, HIST1H2BH, KIF2C, MELK, NUP155, RAB8A, SERBP1, TAF5L, TPX2	2.80E-04
10	SOX2	miR-29a	21693621	ATAD2, BUB1B, HIST1H2BH, IK, KIF2C, KNTC1, NCBP1	2.38E-02
10	E2F1	miR-15b<br>miR-18a	21454377<br>23396442	ATAD2, CDT1, CHEK1, FANCG, GART, GMPS, HNRPF, LMNB1, LYN, MCM2, MCM3, MSH6, NCAPD3, NUP155, PFN1, RAB8A, RFWD3, SERBP1, SFRS1, STIL, TMPO	0.00E+00
10	MYCN	miR-18a<br>miR-19a<br>miR-92<br>miR-93	19946337<br>19946337<br>17943719<br>17943719	CDCA8, CHEK1, DARS2, FEN1, KNTC1, ZWILCH	2.00E-04
11	RUNX1	miR-221<br>miR-222<br>miR-199a	23344057<br>23344057<br>19114653	ATP5C1, BAX, BEGAIN, CFLAR, DNMBP, EDEM3, EFNB2, F11R, IKBKB, IQSEC1, IRF2, LYL1, MAPK1, PTK2B, RANBP2, RCAN1, SP100, SPRY1, TACC1, TICAM1, TNFRSF10B, TNFRSF12A, TNFRSF1A, TXNDC15, UGP2, WEE1	1.61E-06
16	MYC	miR-29c<br>miR-18a<br>miR-92	20564213<br>20516112<br>20516112	DDX11, KIF23, LMNB1, NCAPG, NUP155, TTF2, UCK2	5.23E-03
16	SOX2	miR-29a	21693621	ATAD2, BUB1B, CDC45L, KIF11, KIF15, KIF2C, KNTC1, OIP5, ORC1L, TTF2	3.22E-03

16	E2F1	miR-15b<br>miR-18a	21454377<br>23396444	ATAD2, AURKB, BRCA2, CDC25A, CDC6, CDT1, CENPA, CHEK1, DDX11, DLG7, EXO1, LMNB1, LMNB2, NUP155, OIP5, POLA2, POLD1, RFWD3, STIL, TMPO	6.10E-11
16	MYCN	miR-106a<br>miR-18a<br>miR-19a<br>miR-20a<br>miR-92<br>miR-93	17943719<br>19946337<br>19946337<br>19946337<br>17943719<br>17943719	AURKB, CDC25A, CDC6, CDCA3, CDCA8, CHEK1, DARS2, DLG7, FEN1, KIF11, KNTC1, SLC6A15, TMEM48, TROAP, TTK	0.00E+00
17	MYC	miR-30c	18066065	BMP1, CD248, COL18A1, COL1A1, COL4A2, COL5A1, COL6A1, FBN1, FN1, IL1R1, ITGB1, MMP9, SLC9A1, TRAM2	6.81E-04
17	RUNX1	miR-199a<br>miR-222	19114653<br>23344057	BMP1, COL18A1, COL4A2, COL5A1, COL6A1, COL6A2, COL6A3, IGFBP4, ITGA4, ITGA5, LAMC3, LOXL1, MICAL2, MMP9, MYH9, SERPINE1, SLC9A1, THBD, THBS1, TRAM2	3.68E-04
21	AHR	miR-21<br>miR-22	23052036<br>23052036	ADAM12, CTNNA1, DNMBP, FOSL2, NEDD9, TGFBI	3.67E-03
21	AR	miR-27a	22505583	CALD1, CHSY1, COL5A2, DNMBP, DUSP1, DUSP5, FAS, FEM1C, GALNT2, IQGAP1, ITPKC, LOXL2, MCL1, PARVA, PODXL, RAB8B, SWAP70, TNFRSF10B, TREM1	1.08E-04
21	RUNX1	miR-142<br>miR-221<br>miR-222<br>miR-27a	23509362<br>23344057<br>23344057<br>19114653	ACTN1, BACH1, BCAT1, C5AR1, CALU, CSNK1D, CTNNA1, CTSD, DNAJB1, DNMBP, DUSP1, DUSP5, FOSL2, FZD5, GALNT2, HSPA5, ICAM1, ITGA5, LAMC1, LGALS1, NEDD9, PLAUR, PTPN12, SERPINE1, STK10, SWAP70, TNFRSF10B, TNFRSF12A, TREM1, WWTR1, ZYX	1.01E-08
22	IRF8	miR-19b	23251709	CTSZ, HERPUD1, HLA-B, HLA-C, NCF4, SLC15A3, SLC2A5, TRIM38	1.20E-05
22	RUNX1	miR-142<br>miR-221<br>miR-222	23509362<br>23344057<br>23344057	ALOX5, ALOX5AP, ARSA, BMP2K, C1ORF38, C1QB, C1RL, C5AR1, CD14, CD300A, CD81, CD86, CFD, CREM, CTSD, CTSL1, DUSP6, ELF1, FCER1G, FGR, FXYD5, FYB, GJA4, GMFG, GYPC, HCK, IL10RA, IL4R, ITGA5, ITGB2, MAN2B1, MAP3K8, MYO1F, NCF2, NCF4, NOD2, PLAUR, PLCG2, RAD52, RIN3, RNASE2, RPS6KA1, SH3GLB1, SLA, SLC11A1, SLC16A3, SLCO2B1, SOCS3, SOD2, ST14, STAT1, STAT6, SYK, TBXAS1, THBD, TIMP1, TNFRSF10B, TRIM38, VAV1, WIPF1, YWHAH	1.60E-11
24	RUNX1	miR-142<br>miR-221<br>miR-222	23509362<br>23344057<br>23344057	AGPS, BACH1, CSF2RB, DDX3X, GM2A, HCK, HEXA, LCP1, NCF2, NFKB1, P2RX4, PICALM, SLCO2B1, TGFBR2, TMOD3, WIPF1	5.24E-05
25	AR	miR-27a	22505583	F13A1, FLII, FTH1, HCLS1, IL13RA1, KLF6, PRRX1, PTX3, SOCS3, TGOLN2, ZFP36L1, ZFP36L2	2.32E-03
25	RUNX1	miR-142<br>miR-221<br>miR-222<br>miR-27a	23509362<br>23344057<br>23344057<br>19114653	ACTN1, BCL6, C1RL, C5AR1, CD44, CLIC4, CTSD, CTSL1, FOSL2, FTH1, GALNT2, ICAM1, ITGA5, JUNB, KLF6, LTF, MAN2B1, PLAUR, RCAN1, SERPINE1, SLA, SLC16A3, SOCS3, SOD2, SQSTM1, STAT3, TGOLN2, THBS1, TREM1, VEGFA, ZFP36L2	7.15E-08
26	SOX2	miR-22	18079172	ABLIM1, ALDH5A1, BMPR2, C1ORF61, CEACAM1, CLASP2, CLDN4, CRMP1, CSNK1E, DAPK1, DBN1, DLGAP1, F11R, FRY, HDAC4, HIPK2, KIAA0888, LPHN1, NEK1, PIK3R1, QKI, RAB3D, RAP2A, RAPGEF4, RTN3, SATB1, SPINT1, TERF2, TNIK	3.21E-08
27	SOX2	miR-29a	21693621	BUB1B, CDC2, KIF11, KIF2C, NUSAP1, OIP5, TOP2A, UBE2C	9.97E-04
27	E2F1	miR-15b	21454377	AURKB, CDC2, CENPA, DLG7, GINS2, KIAA0101, MCM2, MLF1IP, OIP5, PBK	1.37E-05
27	MYCN	miR-93	17943719	AURKB, CDCA3, CDCA8, DLG7, DTL, FOXM1, KIF11, KIF4A, MAD2L1, MLF1IP, RAD51AP1, TROAP, TTK, UBE2C	0.00E+00

28	RUNX1	miR-142	23509362	C5AR1, CAPG, CECR1, CTSA, FXYD5, GM2A, GNAI2, HCK, HEXB, IL10RA, ITGB2, MGAT1, MYO1F, PLAUR, PLD3, RAP2B, RNASE2, SH2B3, SYK, TGFB1, TLN1	2.67E-05
29	SOX2	miR-29a	21693621	BUB1B, CDC45L, CDC7, DCC1, KIF15, KIF2C, OIP5, ORC6L, TCF3	1.44E-06
29	E2F1	miR-15b	21454377	AURKB, CDC7, CENPA, DLG7, EZH2, MCM2, MLF1IP, OIP5, PCNA, POLE2, RNASEH2A	2.90E-09
29	MYCN	miR-93	17943719	AURKB, CDC7, DLG7, MLF1IP, RAD51AP1, TTK	1.07E-06
30	IRF8	miR-19b	23251709	ADORA3, CD37, CD74, CTSZ, HLA-DRA, NCF4, SLC2A5	2.11E-05
30	RUNX1	miR-142<br>miR-222	23509362<br>23344057	ALOX5, ALOX5AP, C1ORF38, C1QB, C3, C5AR1, CAPG, CD300A, CD86, CFD, CTSL1, FCER1G, FTL, FXYD5, FYB, GM2A, GMFG, GPR65, GYPC, HCK, IL10RA, ITGB2, LAPTM5, LCP1, LYN, MFNG, MYO1F, NCF2, NCF4, NINJ1, PLCG2, PTPN6, RNASE2, RNASE6, SLA, SLC11A1, SLCO2B1, SPI1, STAB1, SYK, TBXAS1, UCP2, VAV1	5.18E-07
33	AR	miR-27a	22505583	ABCC3, CCDC109B, CLDN10, DRAM, FAS, IGFBP2, IQ-GAP1, LDHA, MSN, OSBPL3, PDGFA, PGCP, PLA2G5, RGN, SLC27A3, STEAP3, SWAP70, TNFAIP6, TNFRSF1A, VAV3	1.70E-03
33	RUNX1	miR-142<br>miR-221<br>miR-222<br>miR-27a	23509362<br>23344057<br>23344057<br>19114653	ANXA5, CSRP1, CTNNA1, DAG1, EMP3, GSTK1, HEXB, LGALS3, MSN, NUAK2, NUCB1, OSBPL3, PGCP, PLAUR, RCAN1, SLC43A3, SWAP70, TAGLN2, TIMP1, TN-FRSF12A, TNFRSF1A, VAV3, VIM	2.65E-03
35	RUNX1	miR-221<br>miR-222	23344057<br>23344057	CALU, CAPNS1, COL4A2, GNB2, HSPA5, ITGA5, LAMC1, PLAUR, SNAP91, SPRY1, SPRY4, VEGFA, ZYX	1.34E-02
37	SOX2	miR-29a	21693621	CCDC46, IGFBP3, IL1RAP, KHDRBS2, LAMC1, P4HB, PDIA4, PLOD2, SPRY1, SPRY4	1.36E-02
37	E2F1	miR-15b<br>miR-18a<br>miR-18a	21454377<br>23396445<br>23396446	ATAD2, AURKB, CDC6, CENPA, CHEK1, E2F8, EZH2, MXD3, OIP5, STIL, TMPO	6.68E-07
37	MYCN	miR-106a<br>miR-18a<br>miR-19a<br>miR-20a<br>miR-93	17943719<br>19946337<br>19946337<br>19946337<br>17943719	AURKB, CDC6, CDCA3, CDCA8, CHEK1, KIF11, KIF4A, MAD2L1, RAD51AP1, TTK	1.82E-10
40	SOX2	miR-29a	21693621	BUB1B, BUB3, CBX5, CDC7, FANCC, FZD2, HDAC2, ILF3, KIF11, KIF15, KIF2C, KNTC1, MUS81, SFPQ, TOP2A, TUBG1	1.54E-07
40	MYCN	miR-93	17943719	CDC7, CDCA8, CHEK1, DTL, FEN1, KIF11, KIF4A, KNTC1, MSH5, NUP93, POLD3, TIMELESS, TTK	1.90E-11
41	AHR	miR-21	23052036	DAB2, FOSL2, IL1R2, IL6, MFSD1, TGFBI	3.17E-03
41	RUNX1	miR-142<br>miR-221<br>miR-222	23509362<br>23344057<br>23344057	ALOX5, C1ORF38, C5AR1, CD14, CDCP1, CTSL1, FOSL2, IL10RA, IL4R, JUNB, MAP3K8, NOD2, PLAUR, RIN3, SLA, SLC11A1, SLC16A3, SOD2, STAB1, THBD, TIMP1, TNFAIP3, TREM1	1.44E-04
43	SOX2	miR-29a	21693621	BUB1B, HN1, KIF2C, OIP5, TOP2A, UBE2C	1.84E-02
43	E2F1	miR-15b<br>miR-18a	21454377<br>23396447	AURKB, CENPA, DTYMK, EZH2, FBXO5, GINS1, GINS2, H2AFZ, KPNA2, MCM2, MLF1IP, OIP5, PBK, PCNA, RNASEH2A, STIL	3.20E-11
43	MYC	miR-18a	20516112	CDKN3, FBXO5, H2AFZ, KIF2C, MELK, MLF1IP	2.78E-02
43	MYCN	miR-18a<br>miR-93	19946337<br>17943719	AURKB, DTL, KIF4A, MAD2L1, MLF1IP, RAD51AP1, TTK, UBE2C, ZWILCH	1.18E-08
45	SOX2	miR-22	18079172	AATF, ANAPC1, ATG4B, CLASP1, CPSF6, CTBP2, DCP1A, EIF5B, ERCC5, FBXW11, NXF1, PDLIM5, POLR3E, SFRS2IP, SPAG9, STAG1, TNPO3, U2AF1, ZC-CHC14, ZNF532, ZNF638	7.00E-04

46	SOX2	miR-22<br>miR-29a	18079172<br>21693621	SMARCC1, ATAD2B, ZNF426, NASP, GOLGA1, SPAST, PRPF4B, PHF16, ZFP37, DIS3, FAM48A, RBM12, CASP8AP2, RBM4, PDCL, DHX35, RBM4B, RFWD3, ZER1, ZBED4, CEP110, MSL2L1, WDR68, HMG20A, ILF3, TLK2, PTK2, SRPK1, ORC2L, STX5, SMG1, NUP155, ANP32A, SUZ12, NCL, EXOSC2, C11orf30, USP39, SETDB1, TTF1, DEDD, GSPT1, CPSF6, WBP11, REV1, BCOR, PCBP2, DDX18, RAD1, SAFB, CSTF1, UBTF, RIF1, STRN3, MDC1, NFYC, TSC2, FUS, NFRKB, RRN3	7.00E-04
47	SOX2	miR-29a	21693621	AATF, ABL1, ATF7IP, CHD4, CPSF6, DCP1A, GOLGA1, KPNA6, POLR3E, SRRM2, TNPO3	4.00E-02
48	RUNX1	miR-142<br>miR-222	23509362<br>23344057	ANXA1, CASP8, CSTA, EFEMP1, FTL, RHOG, RNASE6, S100A10, S100A11	2.32E-04
50	SOX2	miR-22	18079172	APBA2, ASCL1, FCHSD2, GDAP1L1, MAPT, MYST2, TRIM37	1.24E-02
51	RUNX1	miR-142<br>miR-222	23509362<br>23344057	ALOX5, C1QB, CAPG, CD14, CD300A, CD69, CD86, CEBPA, CSF2RB, DLGAP2, FCER1G, FYB, GMFG, HCK, IL10RA, ITGB2, LAPTM5, LYN, MYO1F, NCF2, NCF4, PLAC8, RAC2, RNASE2, RNASE3, RNASE6, RPS6KA1, SLA, SYK, TBXAS1	9.98E-06
54	RUNX1	miR-142<br>miR-199a<br>miR-222	23509362<br>19114653<br>23344057	CITED2, ERG, HEXB, IMPA2, LEPR, RBPMS, SLIT3, TGFBR2, TGM2, TNFSF10, TSPAN4, ZMYM6	3.63E-03
