# Supplementary material for: A Computational Approach to Identifying Gene-microRNA Modules in Cancer
Source: PLoS Comput Biol. 2015 Jan 22;11(1):e1004042. doi: 10.1371/journal.pcbi.1004042 (PMC4303261; doi:10.1371/journal.pcbi.1004042)
Supplement: S12 Table — (PDF) [file pcbi.1004042.s019.pdf]

Table S12. MiRNA regulates TFs and the TFs regulate genes in GBM modules.

Module ID	miRNA	PubMed ID	TF	Genes	p-value
1	miR-17	18700987	RUNX1	ACTR1A, ATP5C1, BUB3, CORO1C, DCLRE1C, IDI1, KRAS, PITRM1, PPP1CC, RSU1, TMPO	8.55E-03
1	miR-17<br>miR-19a<br>miR-19b	23056458<br>23056458<br>23056458	SPI1	ACTR1A, ARHGAP12, CORO1C, DCLRE1C, DDX21, KRAS, PITRM1, PPP1CC, TFAM, TMPO	8.77E-04
2	miR-223<br>miR-34a	20029046<br>21367750	E2F1	AP3B2, ATAD2, AURKB, BCOR, BUB3, CDC, , CDC7, CDT1, CENPM, CHEK1, DDX11, E2F8, EXO1, EZH2, FBXO5, GOT1, HMGB2, KPNA2, LMNB1, MCM2, MCM3, MCM, , MCM7, MSH5, MXD3, POLA2, SNRPA1, STIL	0.00E+00
2	miR-29a<br>miR-34a	21654684<br>21654684	MYCN	AURKB, CCDC15, CDC6, CDC7, CDCA3, CDCA8, CHEK1, FOXM1, KIF11, KIF4A, KNTC1, MAD2L1, MSH5, RAD51AP1, TROAP, TTK, ZWILCH	0.00E+00
2	miR-34a	22020437	SOX2	ATAD2, BUB1B, BUB3, CDC45L, CDC7, KIF11, KIF15, KIF2C, KNTC1, ORC6L, TOP2A, TUBG1	4.55E-04
2	miR-17	18700987	RUNX1	ATAD2, BCOR, BIRC5, BUB1B, BUB3, CCDC15, CDC6, CDCA8, CDT1, CELSR3, CENPF, DDX11, EZH2, FANCI, FOXM1, KIF11, MCM7, RAD51AP1, SNRPA1, STIL, TOP2A, TUBG1, WDHD1	2.46E-02
2	miR-17<br>miR-19a<br>miR-19b<br>miR-20a	23056458<br>23056458<br>23056458<br>23056458	SPI1	BUB1B, CDT1, CENPF, CEP55, CHEK1, E2F8, FANCI, GOT1, KNTC1, KPNA2, MAD2L1, MCM3, MCM7, MKI67, NCAPH, PLK1, POLQ, TACC3, ZNF248, ZWILCH	1.15E-03
5	miR-29a	21654684	MYCN	AURKB, CDCA3, CHEK1, FOXM1, KIF11, KIF4A, KNTC1, LIG1, RAD51AP1, TIMELESS, TROAP, TTK, ZWILCH	0.00E+00
7	miR-17	18700987	RUNX1	BCL7C, BIRC5, BUB1B, CECR5, DBF4, FAM64A, GMNN, H2AFX, KCNQ2, LSM4, MOBKL3, OIP5, ORC4L, PCNA, PPP1CC, RAD51AP1, SFRS3, STIL	3.09E-05
8	miR-17<br>miR-9	18700987<br>19114653	RUNX1	CCL2, CD14, CD44, CHI3L2, EMR1, FOSL2, HCK, ICAM1, IL10RA, IL4R, JUNB, LYN, MAP3K8, PLAUR, PLCG2, PTPN6, RNASE2, SLA, SLC11A1, SOD2, SPI1, STAB1, SYK, THBD, TREM1	6.00E-05
8	miR-21	22931209	SOX2	ADFP, CCL20, CD14, CD44, CTSB, FAS, G0S2, HP, IL1R2, LYN, MAP3K8, MMP7, PBEF1, PLAUR, SER-PINA1, SLC39A14, TNFRSF1B	3.91E-05
9	miR-223<br>miR-34a	20029046<br>21367750	E2F1	ERBB3, FHOD3, MBP, NRXN2, PAK7, PEG3, PLCB1, PPP1R16B, RAP2A, TMSL8	3.61E-03
9	miR-34a	22020437	SOX2	ALG6, BASP1, CA10, DGKB, DNM3, DUSP26, ELMO1, ERBB3, FGF12, FHOD3, G3BP2, GNAI1, HMP19, MBP, NCAM1, OPCML, PAK3, PAK7, PEG3, PGRMC1, PLCB1, SH3GL3, SOX11, STMN2, TMEM16C, TMSL8, VAX2	6.41E-10
9	miR-155	21460854	TCF4	BASP1, C1ORF106, CA10, DGKB, DUSP26, DYNC1I1, ELMO1, FGF12, FHOD3, GNAI1, HSPB3, KIF21B, MAG, OLIG2, PLCB1, PPP1R16B, RBPJ, SATB1, SH3BP4, SNAP91, SOX11, STMN2, TSPYL4	2.37E-06
9	miR-128a	22541023	RUNX1	OLIG2, PEG3, PPP1R16B, SCN3A, SNAP91, SOX4, STMN2	5.66E-03
10	miR-29a	21654684	MYCN	CDCA8, CHEK1, DARS2, FEN1, KNTC1, ZWILCH	2.00E-04
10	miR-18a	23249750	SMAD3	AGPS, ATAD2, CPSF6, HEATR1, NFKB1, PFN1, SMC2, SUPT16H, TAF5L, ZWILCH	1.06E-02
10	miR-17<br>miR-19a<br>miR-19b<br>miR-20a	23056458<br>23056458<br>23056458<br>23056458	SPI1	AGPS, BUB1B, CDT1, CEP55, CHEK1, CSTF2, CUL4B, EIF4A1, FANCG, FEN1, KNTC1, LYN, MAPK14, MCM3, MKI67, NFKB1, NUP155, PLK1, POLQ, PRPF4, RAB8A, TMPO, UPF2, ZWILCH	5.63E-08
11	miR-155	21460854	TCF4	ACOX2, AR, ARSJ, CBLC, FEM1C, FGF14, FGFR4, IQSEC1, IRF2, KCNJ2, LIMS1, MCC, MREG, PDGFD, PDLIM4, RPS6KA3, SNX1, SP100, SPRY1, TACC1, TIAM1, ZBTB38, ZZEF1	4.90E-07

12	miR-128a	22541023	RUNX1	CD34, CHFR, COL18A1, COL4A2, COL5A1, COL6A3, DOCK6, GJA4, IGFBP4, ITGA5, LAMC1, LAMC3, MYH9, NID1, NOTCH3, PDLIM1, TBXA2R	4.20E-04
13	miR-17<br>miR-19a<br>miR-19b	23056458<br>23056458<br>23056458	SPI1	CD320, CDKN2AIP, CDYL, DHX9, EIF6, EXOSC9, FANCI, GNAI3, LSM2, LSM4, MED14, NCAPH, NUP153, RAD1, RFC5, RFXANK, RP2, SP3, SPCS3, USP39	4.29E-04
14	miR-21	22931209	SOX2	ABCC3, AIM1, ARSJ, CA12, CAV1, CCL20, CSNK1D, CTSB, DPP4, FNDC3B, GBE1, GRN, HSPA5, IQGAP1, ITGA5, NRP2, PDIA3, PLAU, PLAUR, RNASE4, SER-PINE1, TGFB1I1, THBS1, WWTR1	4.80E-11
14	miR-155	21460854	TCF4	ANG, ANXA2, ARSJ, CCL20, CD163, GBE1, IL1RAP, IL4R, MMP19, NRP2, PTPN12, SRPR, WWTR1	3.49E-02
16	miR-29a	21654684	MYCN	AURKB, CDC25A, CDC6, CDCA3, CDCA8, CHEK1, DARS2, DLG7, FEN1, KIF11, KNTC1, SLC6A15, TMEM48, TROAP, TTK	0.00E+00
16	miR-18a	23249750	SMAD3	ATAD2, C12ORF48, CCNB2, CDC45L, CDC6, FUBP1, GPR126, OIP5, SLC6A15, SMC2	4.53E-02
16	miR-17<br>miR-19a<br>miR-19b<br>miR-20a	23056458<br>23056458<br>23056458<br>23056458	SPI1	BRCA2, BUB1B, CDT1, CEP55, CHEK1, CHEK2, CSTF2, FANCI, FEN1, FUBP1, KIAA0922, KNTC1, LMNB2, MKI67, NCAPH, NUP155, P2RX5, PLK1, POLD1, POLQ, TMEM48, TMPO	6.12E-05
17	miR-128a<br>miR-30c<br>miR-9	22541023<br>19114653<br>19114653	RUNX1	BMP1, COL18A1, COL4A2, COL5A1, COL6A1, COL6A2, COL6A3, IGFBP4, ITGA4, ITGA5, LAMC3, LOXL1, MI-CAL2, MMP9, MYH9, SERPINE1, SLC9A1, THBD, THBS1, TRAM2	3.68E-04
17	miR-21	22931209	SOX2	AIM1, BGN, CALD1, COL4A2, COL6A1, FN1, ITGA5, LOXL2, MICAL2, MYO1B, PLAU, PRSS23, SERPINE1, SPON2, TAGLN, THBS1, TPM2, TRAM2	3.47E-07
18	miR-155	21460854	TCF4	ADCY2, ARHGEF4, CTBP2, CTNND2, EPHB1, KIF21B, LRRC1, MPP2, SATB1	1.35E-02
20	miR-128a	22541023	RUNX1	A2BP1, AAK1, ARPP-21, CABP1, CNKSR2, DLGAP2, EPB49, GAD2, PPP1R16B, SLC17A7, SNAP25, SNAP91	2.28E-06
21	miR-128a<br>miR-9	22541023<br>19114653	RUNX1	ACTN1, BACH1, BCAT1, C5AR1, CALU, CSNK1D, CTNNA1, CTSD, DNAJB1, DNMBP, DUSP1, DUSP5, FOSL2, FZD5, GALNT2, HSPA5, ICAM1, ITGA5, LAMC1, LGALS1, NEDD9, PLAUR, PTPN12, SERPINE1, STK10, SWAP70, TNFRSF10B, TNFRSF12A, TREM1, WWTR1, ZYX	1.01E-08
21	miR-21	22931209	SOX2	CA12, CALD1, CSNK1D, CTSD, DUSP5, FAS, GBE1, GRN, HSPA5, IQGAP1, ITGA5, LGALS1, LOXL2, NEDD9, NMI, PLAU, PLAUR, PODXL, SDCBP, SERPINE1, TNFRSF10B, TNFRSF12A, WWTR1	1.73E-09
21	miR-155	21460854	TCF4	BCAT1, CALU, CXCR4, DUSP1, DUSP5, FEM1C, GBE1, IL1RAP, LIMS1, NEDD9, PODXL, PTPN12, SRPR, STK10, SWAP70, WWTR1	5.86E-03
22	miR-17<br>miR-9	18700987<br>19114653	RUNX1	ALOX5, ALOX5AP, ARSA, BMP2K, C1ORF38, C1QB, C1RL, C5AR1, CD14, CD300A, CD81, CD86, CFD, CREM, CTSD, CTSL1, DUSP6, ELF1, FCER1G, FGR, FXYD5, FYB, GJA4, GMFG, GYPC, HCK, IL10RA, IL4R, ITGA5, ITGB2, MAN2B1, MAP3K8, MYO1F, NCF2, NCF4, NOD2, PLAUR, PLCG2, RAD52, RIN3, RNASE2, RPS6KA1, SH3GLB1, SLA, SLC11A1, SLC16A3, SLCO2B1, SOCS3, SOD2, ST14, STAT1, STAT6, SYK, TBXAS1, THBD, TIMP1, TNFRSF10B, TRIM38, VAV1, WIPF1, YWHAH	1.60E-11
22	miR-34a	21654684	MYCN	FXYD5, NCF2, RIN3, SIGLEC9, SLAMF8, SLCO2B1	1.37E-03
22	miR-21	22931209	SOX2	BMP2K, BRD8, CD53, CLIC5, COL15A1, CPM, CTSZ, DOCK2, F13A1, GYPC, HCLS1, HLA-G, MERTK, MFSD1, MSR1, NAGA, PLCG2, RAB20, RAD52, RIN3, SIPA1, SLA, SLCO2B1, SYK, TBXAS1, TRADD	2.21E-02
22	miR-155	21460854	TCF4	FGR, MERTK, MGAT4A, PRKCD, PSCDBP, SH3GLB1, SLAMF8	1.05E-02

24	miR-9	19114653	RUNX1	AGPS, BACH1, CSF2RB, DDX3X, GM2A, HCK, HEXA, LCP1, NCF2, NFKB1, P2RX4, PICALM, SLCO2B1, TGFBR2, TMOD3, WIPF1	5.24E-05
24	miR-21	22931209	SOX2	ADAM10, CTSS, EML4, GRN, HLA-DQB1, LCP1, LPXN, TMOD3	6.93E-03
25	miR-128a<br>miR-17<br>miR-9	22541023<br>18700987<br>19114653	RUNX1	ACTN1, BCL6, C1RL, C5AR1, CD44, CLIC4, CTSD, CTSL1, FOSL2, FTH1, GALNT2, ICAM1, ITGA5, JUNB, KLF6, LTF, MAN2B1, PLAUR, RCAN1, SERPINE1, SLA, SLC16A3, SOCS3, SOD2, SQSTM1, STAT3, TGOLN2, THBS1, TREM1, VEGFA, ZFP36L2	7.15E-08
25	miR-21	22931209	SOX2	BCL6, CALD1, CD44, CLIC4, CTSB, CTSD, F13A1, FAS, HP, IQGAP1, ITGA5, PBEF1, PLAU, PLAUR, SERPINE1, SLC7A7, SQSTM1, STAT3, TGFB2, TGOLN2, THBS1, TN-FRSF1B, ZFP36L2	7.81E-09
26	miR-155<br>miR-21	21460854<br>22931209	TCF4	ABLIM1, APC, BMPR2, CHD9, COL13A1, CTNND2, DAPK1, DLGAP1, EDNRB, EIF4G3, FCHSD2, GRIA2, GRIA3, HDAC4, HERC1, HIPK2, INPP5A, KIF1B, KIF3A, KIF3C, LRP1B, MAP2, NEK1, NLGN1, NOTCH1, NRXN1, NTRK3, OLIG2, PPP1R12B, PPP1R9A, RAB-GAP1, RAPGEF2, RAPGEF4, SATB1, SCAPER, SCG3, SHC2, SPTBN1, TAOK3, TNIK, TSPYL4	6.70E-11
26	miR-128a<br>miR-9	22541023<br>19114653	RUNX1	ALDOC, APBA2, ARHGEF10L, BCAN, BMPR2, C1ORF61, CHD9, CLASP2, CRMP1, CTNND2, DAPK1, DHTKD1, DLGAP1, EHD3, F11R, FAIM2, FCHSD2, FRY, GOLM1, HDAC4, HERC1, HIPK2, ICAM1, INPP5A, KIF1B, MADD, MCF2L, MXI1, MYST2, NLGN1, NOTCH1, PIK3R1, PPP1R9A, PTPN22, RAB3D, RABGAP1, SPOP, SPTBN1, TAOK3, TCF12, TNIK	2.75E-07
27	miR-29a	21654684	MYCN	AURKB, CDCA3, CDCA8, DLG7, DTL, FOXM1, KIF11, KIF4A, MAD2L1, MLF1IP, RAD51AP1, TROAP, TTK, UBE2C	0.00E+00
27	miR-17	18700987	RUNX1	BIRC5, BUB1B, CDCA8, CENPF, FAM64A, FANCI, FOXM1, KIF11, MLF1IP, OIP5, PTTG1, RAD51AP1, TOP2A, UBE2S	2.78E-02
28	miR-21	22931209	SOX2	CTSZ, CXCR4, F13A1, FBP1, GM2A, MGAT1, MSR1, NAGA, SH2B3, SIGLEC7, SIPA1, SYK, TRADD	3.14E-03
29	miR-29a	21654684	MYCN	AURKB, CDC7, DLG7, MLF1IP, RAD51AP1, TTK	1.07E-06
29	miR-17	18700987	RUNX1	BIRC5, BUB1B, EZH2, FANCI, MLF1IP, NEIL3, OIP5, PCNA, RAD51AP1, SFRS3, TCF3	3.77E-03
30	miR-9	19114653	RUNX1	ALOX5, ALOX5AP, C1ORF38, C1QB, C3, C5AR1, CAPG, CD300A, CD86, CFD, CTSL1, FCER1G, FTL, FXYD5, FYB, GM2A, GMFG, GPR65, GYPC, HCK, IL10RA, ITGB2, LAPTM5, LCP1, LYN, MFNG, MYO1F, NCF2, NCF4, NINJ1, PLCG2, PTPN6, RNASE2, RNASE6, SLA, SLC11A1, SLCO2B1, SPI1, STAB1, SYK, TBXAS1, UCP2, VAV1	5.18E-07
30	miR-21	22931209	SOX2	ALOX5, ARHGDIB, CAPG, CPVL, CTSB, CTSS, CTSZ, DAB2, F13A1, FLJ20273, FUCA1, GRN, HLA-DQB1, LAPTM5, LCP1, LYN, LYZ, MFNG, PYCARD, SERPINA1, SERPINF1, SLC7A7, TNFRSF1B, UCP2	7.10E-05
30	miR-155	21460854	TCF4	ADORA3, C1QB, C2, CD163, CD86, CPVL, CSF1R, FYB, GIMAP4, GYPC, HCLS1, HLA-DMB, HLA-DRA, LCP1, LYZ, PLCG2, PTAFR, PTPN6, SYK, TBXAS1, TLR5, VAV1	4.59E-02
31	miR-21	22931209	SOX2	ASL, CAPG, CASP4, CPVL, LYN, PROCR, RRAS, SER-PINA1	6.93E-03
32	miR-29a	22506032	SMAD4	EIF4G3, GPATCH8, HNRNPA2B1, LUC7L2, NIPBL, TOP1, TRRAP, YLPM1, ZCCHC14, ZCCHC6	3.93E-02
32	miR-19a	23056458	SPI1	DNMT1, EIF3A, EIF4G3, KPNB1, MED1, MSH3, NFIC, NIPBL, NUP153, OXSR1, RANBP2, RFC1, SART3, SMARCA4, SON, SPEN, TNPO3, TRRAP, ZCCHC6	3.66E-05

33	miR-128a<br>miR-17<br>miR-9	22541023<br>18700987<br>19114653	RUNX1	ANXA5, CSRP1, CTNNA1, DAG1, EMP3, GSTK1, HEXB, LGALS3, MSN, NUAK2, NUCB1, OSBPL3, PGCP, PLAUR, RCAN1, SLC43A3, SWAP70, TAGLN2, TIMP1, TNFRSF12A, TNFRSF1A, VAV3, VIM	2.65E-03
33	miR-204	23516376	SOX11	ANXA2, C13ORF18, CLIC1, CTNNA1, GNG12, PLAUR, SLC43A3, SORT1	7.62E-03
33	miR-21	22931209	SOX2	ABCC3, ANXA5, BCAP31, CA12, CBR1, EMP3, FAS, G0S2, GNG12, IGFBP2, IQGAP1, PARP12, PDGFA, PDLIM4, PLAU, PLAUR, PTRF, PYGL, RBP1, SLC43A3, TAGLN2, TMBIM1, TNFRSF12A, TRIP6, UPP1, VIM	3.79E-10
34	miR-19a<br>miR-19b	23056458<br>23056458	SPI1	DNMT1, EP400, MDC1, NIPBL, RFC1, SART3, SPEN, TRRAP	3.33E-03
35	miR-21	22931209	SOX2	CCDC46, IGFBP3, IL1RAP, KHDRBS2, LAMC1, P4HB, PDIA4, PLOD2, SPRY1, SPRY4	1.36E-02
35	miR-155	21460854	TCF4	CALU, COL4A2, FEM1C, GNB2, GPI, IL1RAP, NDN, PDIA4, SNAP91, SPRY1, SPRY4	1.04E-02
36	miR-17<br>miR-9	18700987<br>19114653	RUNX1	BUB3, C14ORF104, C5ORF30, CDKN1B, COQ7, CP110, EED, ELF2, GMNN, H3F3A, ING1, NOC4L, PSIP1, RAB4A, RALGPS1, RPS6KA5, SH3GLB2, SIRT1, SSNA1, STMN1, YWHAQ	1.04E-02
36	miR-17<br>miR-19a<br>miR-19b<br>miR-20a	23056458<br>23056458<br>23056458<br>23056458	SPI1	AGTPBP1, AIP, C1D, C5ORF30, EED, ING1, MATR3, PRR3, PSIP1, RP2, RPS3, SPAST, TFAM, VPS72, ZBTB5	1.12E-02
37	miR-29a	21654684	MYCN	AURKB, CDC6, CDCA3, CDCA8, CHEK1, KIF11, KIF4A, MAD2L1, RAD51AP1, TTK	1.82E-10
37	miR-17	18700987	RUNX1	ATAD2, BIRC5, BUB1B, CDC6, CDCA8, CENPF, EZH2, FANCI, KIF11, NEIL3, OIP5, RAD51AP1, RIF1, STIL, TMPO, TRIP13, WDHD1	5.57E-04
37	miR-17<br>miR-19a<br>miR-19b<br>miR-20a	23056458<br>23056458<br>23056458<br>23056458	SPI1	BUB1B, CENPF, CHEK1, E2F8, FANCI, MAD2L1, NCAPH, RFC5, TMPO	4.05E-02
38	miR-9	19114653	RUNX1	ANK2, APBA2, BCAN, CLASP2, CRMP1, CTNND2, DDAH1, NCAN, NDRG4, NES, S100B, TNIK, TTYH1, WASF3	2.38E-02
39	miR-155<br>miR-21	21460854<br>21956205	TCF4	ADCY2, CTNND2, DPP6, GRIA2, HIPK2, KIF3A, NEK1, NRXN1, NTRK3, PID1, SCAPER, SCG3, SCN2A, SORBS1, TAOK3	4.34E-05
40	miR-29a	21654684	MYCN	CDC7, CDCA8, CHEK1, DTL, FEN1, KIF11, KIF4A, KNTC1, MSH5, NUP93, POLD3, TIMELESS, TTK	1.90E-11
40	miR-29a	22506032	SMAD4	BRCA1, BUB3, CDC7, CENPF, DTL, EXO1, EZH2, FANCC, KIF5B, LOC81691, MYB, NUP93	2.32E-02
41	miR-128a<br>miR-17<br>miR-9	22541023<br>18700987<br>19114653	RUNX1	ALOX5, C1ORF38, C5AR1, CD14, CDCP1, CTSL1, FOSL2, IL10RA, IL4R, JUNB, MAP3K8, NOD2, PLAUR, RIN3, SLA, SLC11A1, SLC16A3, SOD2, STAB1, THBD, TIMP1, TNFAIP3, TREM1	1.44E-04
41	miR-21	22931209	SOX2	ADFP, AIM1, ALOX5, CCL20, CD14, CTSB, DAB2, F13A1, HP, IL1R2, MAP3K8, PLAUR, RIN3, SLC39A14, TNFRSF1B	2.08E-04
43	miR-29a	21654684	MYCN	AURKB, DTL, KIF4A, MAD2L1, MLF1IP, RAD51AP1, TTK, UBE2C, ZWILCH	1.18E-08
43	miR-17	18700987	RUNX1	BIRC5, BUB1B, C9ORF46, CENPF, CKS2, DBF4, EZH2, FANCI, MLF1IP, OIP5, PCNA, PTTG1, RAD51AP1, RFC4, STIL, TOP2A	6.28E-03
43	miR-17<br>miR-19b	23056458<br>23056458	SPI1	BUB1B, CCNB1, CENPF, CKS1B, CKS2, DTL, FANCI, KPNA2, LSM2, MAD2L1, RFC4, ZWILCH	5.16E-03
46	miR-17<br>miR-19a<br>miR-19b	23056458<br>23056458<br>23056458	SPI1	CASP8AP2, DDX18, DHX35, GOLGA1, MDC1, NFYC, NUP155, PCBP2, PRPF4B, RAD1, SETDB1, SPAST, STX5, USP39	1.62E-03

48	miR-21	22931209	SOX2	ANXA1, BMP2K, DPYD, EFEMP1, FAS, GM2A, HLA-E, HNMT, LAMP2, NAGA, NPC2, PAK7, PGCP, S100A10, SERPINB1	3.89E-03
48	miR-155	21460854	TCF4	ANG, ANXA4, CASP8, CD86, DPYD, ECM2, EFEMP1, FYB, HNMT, HOMER3, PROCR, SP100, TLR5	4.82E-02
50	miR-223	20029046	E2F1	APBA2, ASCL1, FCHSD2, GDAP1L1, MAPT, MYST2, TRIM37	1.24E-02
50	miR-155<br>miR-21	21460854<br>21956205	TCF4	ANKRD46, ARHGEF7, BMP7, DGKB, DLGAP1, DPP6, FCHSD2, GRIA2, IL1R1, MAP2, OLIG2, RAPGEF4, REV3L, SCG3, SEZ6L, SOX11, TRIM37, TSPAN12, ZFP2	7.06E-07
50	miR-9	19114653	RUNX1	ANKRD46, APBA2, BCAN, CLASP2, CRMP1, DLGAP1, DPF1, FCHSD2, GTF2I, IPO9, KCNQ2, MYST2, NCAN, PAFAH1B3, PLA2G6, RBM4B, REV3L, TOP2B, TRIM24	4.78E-05
50	miR-17<br>miR-20a	23056458<br>23056458	SPI1	APBA2, DLGAP1, DPP6, IPO9, RBM4B, SCG3	1.55E-02
51	miR-9	19114653	RUNX1	ALOX5, C1QB, CAPG, CD14, CD300A, CD69, CD86, CEBPA, CSF2RB, DLGAP2, FCER1G, FYB, GMFG, HCK, IL10RA, ITGB2, LAPTM5, LYN, MYO1F, NCF2, NCF4, PLAC8, RAC2, RNASE2, RNASE3, RNASE6, RPS6KA1, SLA, SYK, TBXAS1	9.98E-06
51	miR-21	22931209	SOX2	ALOX5, CAPG, CD14, CTSS, LAPTM5, LPXN, LYN, LYZ, PLAC8, PYCARD, SERPINA1, SLC7A7	3.85E-02
52	miR-34a	22020437	SOX2	AMPH, B4GALNT1, CA10, CLASP2, DCX, DNM3, DUSP26, ELAVL4, FGF12, HMP19, MAGI1, MBP, MYT1, NCAM1, NOL4, OPCML, PAK3, PAK7, PGRMC1, PLCB1, SOX11, SPTBN2, STMN2, TMEM16C	6.75E-10
52	miR-155	21460854	TCF4	AMPH, CA10, DUSP26, DYNC1I1, FGF12, MAGI1, NM-NAT2, NOL4, PLCB1, SFPQ, SNAP91, SOX11, STMN2, TF	4.91E-03
54	miR-9	19114653	RUNX1	CITED2, ERG, HEXB, IMPA2, LEPR, RBPMS, SLIT3, TGFBR2, TGM2, TNFSF10, TSPAN4, ZMYM6	3.63E-03
54	miR-21	22931209	SOX2	CASP4, CST7, FBP1, GATA6, LEPR, MALL, MME, PPIC, PROCR, SOSTDC1, TGM2, TNFSF10	5.66E-06
54	miR-155	21460854	TCF4	ERG, IL7R, LEPR, PROCR, RBPMS, SLC25A24, SLIT3, TGFBR2	3.79E-02
