# Supplementary material for: A Computational Approach to Identifying Gene-microRNA Modules in Cancer
Source: PLoS Comput Biol. 2015 Jan 22;11(1):e1004042. doi: 10.1371/journal.pcbi.1004042 (PMC4303261; doi:10.1371/journal.pcbi.1004042)
Supplement: S13 Table — (PDF) [file pcbi.1004042.s020.pdf]

Table S13. Exprementally validated gene-miRNA interactions in GBM.

Module ID	Gene	miRNA	Validation Method	Reference
1	ZMYND11	miR-222	CLASH	23622248
1	ARHGAP12	miR-19b	Sequencing	20371350
1	ARHGAP12	miR-130b	Sequencing	20371350
1	DDX21	miR-222	CLASH	23622248
2	NCAPG	miR-34a	Proteomics	21566225
2	NCAPG	miR-16	Proteomics	18668040
2	STIL	miR-20a	CLASH	23622248
2	MKI67	miR-29b	CLASH	23622248
2	MKI67	miR-34a	Proteomics	21566225
2	MKI67	miR-16	Proteomics	18668040
2	CENPF	miR-93	CLASH	23622248
2	CENPF	miR-16	Proteomics	18668040
2	KIF2C	miR-20a	CLASH	23622248
2	KIF2C	miR-34a	Proteomics	21566225
2	KIF2C	miR-16	Proteomics	18668040
2	ASF1B	miR-130b	CLASH	23622248
2	ASF1B	miR-18a	CLASH	23622248
2	KIF11	miR-34a	Proteomics	21566225
2	RACGAP1	miR-16	Proteomics	18668040
2	MCM10	miR-34a	CLASH	23622248
2	KIF23	miR-106b	Microarray	17242205
2	KIF23	miR-25	CLASH	23622248
2	KIF23	miR-16	Proteomics	18668040
2	CENPM	miR-106b	CLASH	23622248
2	KIF4A	miR-34a	Proteomics	21566225
2	KIF4A	miR-222	CLASH	23622248
2	MXD3	miR-18a	CLASH	23622248
2	CDT1	miR-20a	CLASH	23622248
2	CDCA8	miR-16	Proteomics	18668040
2	KIF14	miR-16	Proteomics	18668040
2	E2F8	miR-19b	Sequencing	20371350
2	TRAIP	miR-34a	Proteomics	21566225
2	BUB1B	miR-22	CLASH	23622248
2	KPNA2	miR-93	CLASH	23622248
2	KPNA2	miR-106a	CLASH	23622248
2	KPNA2	miR-16	Proteomics	18668040
2	PLK1	miR-16	pSILAC, Proteomics, Other	18668040
2	TACC3	miR-18a	CLASH	23622248
2	CHEK1	miR-15b	CLASH	23622248
2	AURKB	miR-16	Microarray, qRT-PCR	19738602
2	AURKB	miR-16	Proteomics	18668040
2	EZH2	miR-93	CLASH	23622248
2	EZH2	miR-25	Luciferase reporter assay, qRT-PCR, Western blot	22399519
2	BRCA1	miR-16	Luciferase reporter assay	19144710
2	MCM2	miR-34a	Proteomics	21566225
2	MCM4	miR-34a	Proteomics	21566225
2	ATAD2	miR-106b	Microarray	17242205
2	CDC25C	miR-34a	Microarray	19461653
2	MCM3	miR-93	CLASH	23622248
2	MCM3	miR-34a	Proteomics	21566225
2	MCM3	miR-222	CLASH	23622248
2	CEP55	miR-16	Sequencing	20371350
2	MCM7	miR-93	CLASH	23622248
2	MCM7	miR-34a	Proteomics	21566225
2	MCM7	miR-222	CLASH	23622248
2	CDC20	miR-93	CLASH	23622248

2	CDC20	miR-18a	CLASH	23622248
2	CDC20	miR-34a	Proteomics	21566225
2	CDC20	miR-34a	CLASH	23622248
2	CDC20	miR-16	Proteomics	18668040
2	SNRPA1	miR-34a	CLASH	23622248
2	SNRPA1	miR-16	Proteomics	18668040
2	MAD2L1	miR-93	CLASH	23622248
2	BIRC5	miR-93	CLASH	23622248
2	MCM6	miR-34a	Proteomics	21566225
4	STAT1	miR-34a	Proteomics	21566225
5	NASP	miR-93	CLASH	23622248
5	NASP	miR-29a	Luciferase reporter assay, Western blot	22080513
5	NASP	miR-29a	Luciferase reporter assay	22194605
5	MCM7	miR-93	CLASH	23622248
5	TRIM28	miR-93	CLASH	23622248
5	CENPF	miR-93	CLASH	23622248
5	TMPO	miR-106b	CLASH	23622248
5	MCM3	miR-93	CLASH	23622248
5	EZH2	miR-93	CLASH	23622248
5	KIF23	miR-106b	Microarray	17242205
7	CENPM	miR-106b	CLASH	23622248
7	MXD3	miR-18a	CLASH	23622248
7	CD320	miR-18a	CLASH	23622248
7	BIRC5	miR-93	CLASH	23622248
7	MAD2L1	miR-93	CLASH	23622248
7	CCNB1	miR-20a	CLASH	23622248
7	PCNA	miR-18a	CLASH	23622248
7	PPP1CC	miR-15b	CLASH	23622248
7	STIL	miR-20a	CLASH	23622248
8	PTX3	miR-21	Luciferase reporter assay	21131358
8	SOD2	miR-222	Flow, Luciferase reporter assay, Microarray, qRT-PCR, Western blot	19487542
8	CCL20	miR-21	ELISA, Immunofluorescence, Luciferase reporter assay	22001440
8	BIRC3	miR-34a	Microarray, Northern blot	17540599
8	SLC39A14	miR-9	Microarray	17612493
8	SLC39A14	miR-155	Proteomics	18668040
8	CPD	miR-155	Proteomics	18668040
8	CPD	miR-19b	Sequencing	20371350
8	ICAM1	miR-21	Luciferase reporter assay, Microarray, qRT-PCR	21131358
8	ICAM1	miR-222	Luciferase reporter assay	19949084
8	ICAM1	miR-155	Luciferase reporter assay	19949084
8	ICAM1	miR-155	qRT-PCR	21310411
8	ICAM1	miR-221	Luciferase reporter assay, Western blot	20110463
8	ICAM1	miR-221	Luciferase reporter assay	19949084
8	IL1B	miR-21	Luciferase reporter assay, Microarray, qRT-PCR	21131358
8	PLAUR	miR-155	Proteomics	18668040
8	CEBPB	miR-155	Luciferase reporter assay	18367535
8	CEBPB	miR-155	Microarray, qRT-PCR, Western blot	19193853
8	CEBPB	miR-155	Luciferase reporter assay, qRT-PCR, Western blot	20427544
8	CEBPB	miR-155	pSILAC, Proteomics	18668040
8	CEBPB	miR-155	Luciferase reporter assay	19596814
8	CEBPB	miR-155	ELISA, Immunoblot, Immunocytochemistry, Immunofluorescence, Luciferase reporter assay, qRT-PCR	19887047
8	CEBPB	miR-34a	Luciferase reporter assay, Reporter assay	20598588
8	JUNB	miR-155	Proteomics	18668040
8	SPI1	miR-9	Microarray	17612493
8	SPI1	miR-155	Luciferase reporter assay	19386588
8	SPI1	miR-155	Reporter assay	20680360
8	SPI1	miR-155	Proteomics	18668040
8	SPI1	miR-34a	Luciferase reporter assay, Reporter assay	20598588

8	CCR1	miR-21	Luciferase reporter assay	21131358
8	CCL2	miR-155	Proteomics	18668040
8	FAS	miR-21	Microarray	20048743
8	LYN	miR-222	CLASH	23622248
8	CD44	miR-34a	Immunohistochemistry, qRT-PCR, Western blot, Reporter assay, Other	21240262
8	IL13RA1	miR-155	Luciferase reporter assay	21097505
9	INA	miR-155	Proteomics	18668040
9	SOX4	miR-138	Luciferase reporter assay	23389731
9	SOX4	miR-204	Luciferase reporter assay, qRT-PCR, Western blot	23204229
9	SOX4	miR-340	Sequencing	20371350
9	RAP2A	miR-34a	CLASH	23622248
9	SH3BP4	miR-155	pSILAC, Proteomics, Other	18668040
9	SH3BP4	miR-7	Microarray	19073608
9	SH3BP4	miR-93	CLASH	23622248
9	ERBB3	miR-22	Luciferase reporter assay, qRT-PCR, Western blot	22484852
9	RBPJ	miR-155	Proteomics	18668040
9	RBPJ	miR-18a	CLASH	23622248
9	AMOTL2	miR-27a	Sequencing	20371350
9	G3BP2	miR-34a	Proteomics	21566225
9	MAP3K4	miR-93	CLASH	23622248
10	MKI67	miR-29b	CLASH	23622248
10	GART	miR-18a	CLASH	23622248
10	NONO	miR-93	CLASH	23622248
10	CHEK1	miR-15b	CLASH	23622248
10	HEATR1	miR-106a	CLASH	23622248
10	SERBP1	miR-15b	CLASH	23622248
10	SERBP1	miR-93	CLASH	23622248
10	SERBP1	miR-18a	CLASH	23622248
10	CDT1	miR-20a	CLASH	23622248
10	TMPO	miR-106b	CLASH	23622248
10	BUB1B	miR-22	CLASH	23622248
10	NUDT21	miR-20a	CLASH	23622248
10	MSH6	miR-106b	CLASH	23622248
10	MCM3	miR-93	CLASH	23622248
10	SRPK1	miR-93	Sequencing	20371350
10	EIF4A1	miR-15b	Luciferase reporter assay	16609010
10	EIF4A1	miR-93	CLASH	23622248
10	STIL	miR-20a	CLASH	23622248
10	ATAD2	miR-106b	Microarray	17242205
10	GMPS	miR-18a	CLASH	23622248
10	KIF2C	miR-20a	CLASH	23622248
10	SUPT16H	miR-18a	CLASH	23622248
10	SUPT16H	miR-25	CLASH	23622248
11	WEE1	miR-221	Sequencing	20371350
11	WEE1	miR-155	Reporter assay, Other	20584899
11	PDGFD	miR-21	Microarray	18591254
11	LIMS1	miR-221	Sequencing	20371350
11	EDEM3	miR-155	pSILAC, Proteomics, Other	18668040
11	PXN	miR-221	CLASH	23622248
11	FGF2	miR-155	Proteomics	18668040
11	FAS	miR-21	Microarray	20048743
11	JUN	miR-155	Luciferase reporter assay, qRT-PCR, Western blot	22508041
11	DDB2	miR-155	Proteomics	18668040
11	ERBB2	miR-21	qRT-PCR, Western blot	19419954
11	TICAM1	miR-221	Luciferase reporter assay, Northern blot, qRT-PCR, Western blot	21236259
11	MYD88	miR-155	Western blot	21030878
11	RPS6KA3	miR-21	Microarray	18591254
11	FLNA	miR-222	CLASH	23622248
11	FLNA	miR-221	CLASH	23622248

11	FLNA	miR-155	Proteomics	18668040
11	TIAM1	miR-221	CLASH	23622248
11	TIAM1	miR-21	Luciferase reporter assay, Microarray, Northern blot, qRT-PCR	20826792
11	TIAM1	miR-21	Luciferase reporter assay	23442323
11	PTX3	miR-21	Luciferase reporter assay	21131358
11	ZBTB38	miR-21	Microarray	18591254
12	COL1A1	miR-143	Immunoblot, Luciferase reporter assay, qRT-PCR	19913496
13	KLHDC3	miR-93	CLASH	23622248
13	KLHDC3	miR-20a	CLASH	23622248
13	UQCRC1	miR-93	CLASH	23622248
13	UQCRC1	miR-20a	CLASH	23622248
13	DHX9	miR-93	CLASH	23622248
13	STIL	miR-20a	CLASH	23622248
13	GPS1	miR-93	CLASH	23622248
13	NUP153	miR-93	CLASH	23622248
13	NUP153	miR-106b	CLASH	23622248
13	NUDT21	miR-20a	CLASH	23622248
13	SMAD4	miR-130b	Sequencing	20371350
13	SMAD4	miR-93	Sequencing	20371350
13	SMAD4	miR-19b	Sequencing	20371350
13	SMAD4	miR-19a	Luciferase reporter assay, Microarray	20940405
13	SMAD4	miR-20a	Microarray	20940405
13	KIF23	miR-106b	Microarray	17242205
14	CAV1	miR-34b	Microarray	19461653
14	VEGFA	miR-21	qRT-PCR	19435867
14	VEGFA	miR-21	Western blot, qRT-PCR	21544242
14	VEGFA	miR-9	qRT-PCR, Other	20173740
14	VEGFA	miR-34a	ELISA, Luciferase reporter assay	18320040
14	VEGFA	miR-34b	ELISA, Luciferase reporter assay	18320040
14	NRP1	miR-181a	Microarray	17612493
14	NRP1	miR-181a	Sequencing	20371350
14	THBS1	miR-155	Proteomics	18668040
14	FAM46A	miR-21	Microarray	18591254
14	FAM46A	miR-9	Microarray	17612493
14	FAM46A	miR-34a	CLASH	23622248
14	CAST	miR-222	CLASH	23622248
14	AIM1	miR-21	Microarray	18591254
14	FNDC3B	miR-155	Proteomics	18668040
14	CCL20	miR-21	ELISA, Immunofluorescence, Luciferase reporter assay	22001440
14	GLIPR1	miR-155	Proteomics	18668040
14	PLAUR	miR-155	Proteomics	18668040
14	FLNA	miR-222	CLASH	23622248
14	FLNA	miR-155	Proteomics	18668040
14	FLNA	miR-221	CLASH	23622248
14	FLNA	miR-23a	CLASH	23622248
14	TGFBI	miR-21	Luciferase reporter assay, Western blot	19136465
14	TGFBI	miR-21	Microarray	18591254
14	TGFBI	miR-9	In situ hybridization, Luciferase reporter assay	21720722
14	ANXA2	miR-155	Proteomics	18668040
14	P4HB	miR-210	2DGE, immunoprecipitaion, Mass spectrometry, Microarray, qRT-PCR, Western blot	19826008
14	IL13RA1	miR-155	Luciferase reporter assay	21097505
14	GRN	miR-9	Reporter assay	20479936
15	CENPF	miR-93	CLASH	23622248
15	REV3L	miR-93	Sequencing	20371350
15	UBE2C	miR-20a	CLASH	23622248
15	CENPM	miR-106b	CLASH	23622248
15	CDT1	miR-20a	CLASH	23622248
15	BUB1B	miR-22	CLASH	23622248

15	TTC3	miR-130b	CLASH	23622248
15	HN1	miR-106b	Microarray	17242205
15	TRIB2	miR-106b	CLASH	23622248
15	GPR162	miR-93	CLASH	23622248
15	CCND1	miR-93	Sequencing	20371350
15	CCND1	miR-19a	flow, Luciferase reporter assay, Microarray, qRT-PCR, Western blot	20133739
15	CCND1	miR-106b	Luciferase reporter assay	21283765
15	CCND1	miR-20a	Luciferase reporter assay, qRT-PCR, Western blot	18695042
15	CCND1	miR-20a	Luciferase reporter assay	21283765
15	POLR2A	miR-93	CLASH	23622248
15	E2F3	miR-106b	Luciferase reporter assay	21283765
15	E2F3	miR-20a	Luciferase reporter assay	21283765
15	KIF4A	miR-222	CLASH	23622248
15	MAP3K4	miR-93	CLASH	23622248
15	E2F1	miR-130b	Sequencing	20371350
15	E2F1	miR-93	Luciferase reporter assay, Western blot	19486339
15	E2F1	miR-93	Luciferase reporter assay, Microarray, Western blot	18328430
15	E2F1	miR-93	Sequencing	20371350
15	E2F1	miR-106a	Immunohistochemistry, Microarray, Western blot	20643754
15	E2F1	miR-106a	qRT-PCR	18521848
15	E2F1	miR-106a	Luciferase reporter assay, Western blot	21656380
15	E2F1	miR-106b	Luciferase reporter assay, qRT-PCR	18676839
15	E2F1	miR-106b	Luciferase reporter assay, Western blot	19486339
15	E2F1	miR-106b	Luciferase reporter assay, Microarray, Western blot	18328430
15	E2F1	miR-106b	Luciferase reporter assay	21283765
15	E2F1	miR-106b	Reporter assay	18212054
15	E2F1	miR-20a	Western blot	19110058
15	E2F1	miR-20a	Western blot	18025036
15	E2F1	miR-20a	Western blot	18836483
15	E2F1	miR-20a	Luciferase reporter assay, Northern blot, Western blot, Reporter assay	15944709
15	E2F1	miR-20a	Luciferase reporter assay	21283765
15	MAD2L1	miR-93	CLASH	23622248
15	TCERG1	miR-222	CLASH	23622248
15	KIF2C	miR-20a	CLASH	23622248
15	CDC20	miR-93	CLASH	23622248
15	MYO9B	miR-93	CLASH	23622248
15	ASF1B	miR-130b	CLASH	23622248
15	FUBP1	miR-22	CLASH	23622248
15	FUBP1	miR-222	CLASH	23622248
15	TMPO	miR-106b	CLASH	23622248
15	RPL21	miR-106b	CLASH	23622248
15	RPL21	miR-20a	CLASH	23622248
15	HERPUD1	miR-93	CLASH	23622248
15	TRPC3	miR-130b	Sequencing	20371350
15	TRPC3	miR-19b	Sequencing	20371350
15	PCNT	miR-222	CLASH	23622248
16	MKI67	miR-29b	CLASH	23622248
16	HMMR	miR-93	CLASH	23622248
16	HMMR	miR-106b	CLASH	23622248
16	KIF2C	miR-20a	CLASH	23622248
16	ASF1B	miR-130b	CLASH	23622248
16	ASF1B	miR-18a	CLASH	23622248
16	CHEK1	miR-15b	CLASH	23622248
16	KIF23	miR-106b	Microarray	17242205
16	KIF23	miR-25	CLASH	23622248
16	CENPN	miR-19b	Sequencing	20371350
16	CDT1	miR-20a	CLASH	23622248
16	TMPO	miR-106b	CLASH	23622248
16	ATAD2	miR-106b	Microarray	17242205

16	STIL	miR-20a	CLASH	23622248
16	OIP5	miR-29c	Sequencing	20371350
16	CDC20	miR-93	CLASH	23622248
16	CDC20	miR-18a	CLASH	23622248
16	CDC25A	miR-18a	CLASH	23622248
16	LMNB2	miR-18a	Sequencing	20371350
16	LMNB2	miR-18a	CLASH	23622248
17	MMP9	miR-21	qRT-PCR	19435867
17	THBS1	miR-155	Proteomics	18668040
17	AIM1	miR-21	Microarray	18591254
17	NID2	miR-181d	CLASH	23622248
17	COL1A1	miR-143	Immunoblot, Luciferase reporter assay, qRT-PCR	19913496
17	COL4A2	miR-155	Proteomics	18668040
17	PXDN	miR-9	Microarray	17612493
17	SPON2	miR-9	Microarray	17612493
17	COL5A2	miR-21	Microarray	18591254
17	COL5A2	miR-222	CLASH	23622248
17	TGFBI	miR-21	Luciferase reporter assay, Western blot	19136465
17	TGFBI	miR-21	Microarray	18591254
17	TGFBI	miR-9	In situ hybridization, Luciferase reporter assay	21720722
17	TAGLN	miR-9	Microarray	17612493
17	ITGB1	miR-9	Luciferase reporter assay	23530058
17	MYH9	miR-9	Microarray	17612493
17	CALD1	miR-21	Microarray	18591254
17	ITGA4	miR-30c	qRT-PCR	23418453
17	SERPINE1	miR-145	Immunoblot, Luciferase reporter assay, qRT-PCR, Western blot	22108519
17	SERPINE1	miR-30c	Luciferase reporter assay	21175428
17	SERPINE1	miR-143	Immunoblot, Luciferase reporter assay, qRT-PCR, Western blot	22108519
18	MPP2	miR-155	Proteomics	18668040
20	SNCA	miR-7	qRT-PCR, Luciferase reporter assay, Western blot	19628698
20	SNCA	miR-7	Immunocytochemistry, Luciferase reporter assay, qRT-PCR, Western blot	20106983
21	PTX3	miR-21	Luciferase reporter assay	21131358
21	ADAM9	miR-23a	CLASH	23622248
21	ZYX	miR-221	CLASH	23622248
21	BCAT1	miR-21	Microarray	18591254
21	BCAT1	miR-155	Microarray	19193853
21	BACH1	miR-155	Luciferase reporter assay, Microarray, qRT-PCR	19193853
21	BACH1	miR-155	qRT-PCR, Luciferase reporter assay	18299402
21	BACH1	miR-155	Luciferase reporter assay	18367535
21	BACH1	miR-155	Immunohistochemistry, In situ hybridization, Luciferase reporter assay, Microarray, qRT-PCR, Western blot	21541331
21	BACH1	miR-155	Reporter assay, Other	20584899
21	BACH1	miR-155	Reporter assay	17881434
21	LIMS1	miR-221	Sequencing	20371350
21	RAB8B	miR-101	Sequencing	20371350
21	PODXL	miR-155	pSILAC, Proteomics, Other	18668040
21	SERPINE1	miR-99a	Luciferase reporter assay	21175428
21	CHSY1	miR-9	Sequencing	20371350
21	ICAM1	miR-21	Luciferase reporter assay, Microarray, qRT-PCR	21131358
21	ICAM1	miR-222	Luciferase reporter assay	19949084
21	ICAM1	miR-155	Luciferase reporter assay	19949084
21	ICAM1	miR-155	qRT-PCR	21310411
21	ICAM1	miR-221	Luciferase reporter assay, Western blot	20110463
21	ICAM1	miR-221	Luciferase reporter assay	19949084
21	FOSL2	miR-99a	CLASH	23622248
21	ITPKC	miR-23a	CLASH	23622248
21	PLAUR	miR-155	Proteomics	18668040
21	CALD1	miR-21	Microarray	18591254
21	CALD1	miR-34a	Proteomics	21566225

21	TGFBI	miR-21	Luciferase reporter assay, Western blot	19136465
21	TGFBI	miR-21	Microarray	18591254
21	TGFBI	miR-9	In situ hybridization, Luciferase reporter assay	21720722
21	CAST	miR-222	CLASH	23622248
21	MCL1	miR-181a	Luciferase reporter assay	22610076
21	MCL1	miR-101	Luciferase reporter assay, qRT-PCR, Western blot, Reporter assay	19155302
21	MCL1	miR-101	Luciferase reporter assay, Western blot	20829195
21	MCL1	miR-101	qRT-PCR	20712078
21	MCL1	miR-181b	Luciferase reporter assay	22610076
21	FLNA	miR-222	CLASH	23622248
21	FLNA	miR-155	Proteomics	18668040
21	FLNA	miR-221	CLASH	23622248
21	FLNA	miR-23a	CLASH	23622248
21	CDKN1A	miR-146b	Luciferase reporter assay	20101223
21	IL13RA1	miR-155	Luciferase reporter assay	21097505
21	SDCBP	miR-155	pSILAC, Proteomics	18668040
21	SDCBP	miR-155	Reporter assay, Other	20584899
21	FAS	miR-21	Microarray	20048743
21	FAS	miR-504	qRT-PCR	20542001
21	CALU	miR-155	Proteomics	18668040
21	CTNNA1	miR-155	Proteomics	18668040
21	DUSP5	miR-181a	Luciferase reporter assay, Western blot	17382377
21	DUSP5	miR-27a	CLASH	23622248
21	DUSP1	miR-101	Luciferase reporter assay, qRT-PCR, Western blot	21068409
21	COL5A2	miR-21	Microarray	18591254
21	COL5A2	miR-222	CLASH	23622248
21	GRN	miR-9	Reporter assay	20479936
21	DNAJB1	miR-155	pSILAC, Proteomics, Other	18668040
22	SOD2	miR-222	Flow, Luciferase reporter assay, Microarray, qRT-PCR, Western blot	19487542
22	DSE	miR-21	Microarray	18591254
22	TRIM38	miR-21	Microarray	18591254
22	GRN	miR-9	Reporter assay	20479936
22	LHFPL2	miR-221	Sequencing	20371350
22	LHFPL2	miR-93	Sequencing	20371350
22	SYNGR2	miR-9	Sequencing	20371350
22	TLR2	miR-19b	Luciferase reporter assay, qRT-PCR, Western blot	22105995
22	TLR2	miR-106b	Microarray	17242205
22	TLR2	miR-19a	Luciferase reporter assay, qRT-PCR, Western blot	22105995
22	MGAT4A	miR-21	Microarray	18591254
22	SLC2A5	miR-9	Microarray	17612493
22	HERPUD1	miR-93	CLASH	23622248
22	MYD88	miR-155	Western blot	21030878
22	PLAUR	miR-155	Proteomics	18668040
22	STAT1	miR-34a	Proteomics	21566225
22	CEBPB	miR-34a	Luciferase reporter assay, Reporter assay	20598588
22	CEBPB	miR-155	Luciferase reporter assay	18367535
22	CEBPB	miR-155	Microarray, qRT-PCR, Western blot	19193853
22	CEBPB	miR-155	Luciferase reporter assay, qRT-PCR, Western blot	20427544
22	CEBPB	miR-155	pSILAC, Proteomics	18668040
22	CEBPB	miR-155	Luciferase reporter assay	19596814
22	CEBPB	miR-155	ELISA, Immunoblot, Immunocytochemistry, Immunofluorescence, Luciferase reporter assay, qRT-PCR	19887047
22	AP1B1	miR-222	CLASH	23622248
22	DUSP6	miR-181a	Luciferase reporter assay, Western blot	17382377
22	INCENP	miR-181a	Microarray	17612493
22	AAMP	miR-130b	CLASH	23622248
22	CD81	miR-155	Proteomics	18668040
22	CSF1	miR-130b	ELISA, Luciferase reporter assay, qRT-PCR	22005523
22	CCR1	miR-21	Luciferase reporter assay	21131358

22	PSAP	miR-19b	Sequencing	20371350
22	PSAP	miR-130b	Sequencing	20371350
22	PSAP	miR-19a	Luciferase reporter assay	23451058
22	SH3GLB1	miR-93	Sequencing	20371350
22	IL13RA1	miR-155	Luciferase reporter assay	21097505
24	BACH1	miR-155	Luciferase reporter assay, Microarray, qRT-PCR	19193853
24	BACH1	miR-155	qRT-PCR, Luciferase reporter assay	18299402
24	BACH1	miR-155	Luciferase reporter assay	18367535
24	BACH1	miR-155	Immunohistochemistry, In situ hybridization, Luciferase reporter assay, Microarray, qRT-PCR, Western blot	21541331
24	BACH1	miR-155	Reporter assay, Other	20584899
24	BACH1	miR-155	Reporter assay	17881434
24	SNAP23	miR-34a	Proteomics	21566225
24	TGFBR2	miR-21	qRT-PCR, Western blot	19906824
24	TGFBR2	miR-21	qRT-PCR, Luciferase reporter assay, Western blot, Reporter assay	19816956
24	TGFBR2	miR-21	Luciferase reporter assay, qRT-PCR, Western blot	18829576
24	TGFBR2	miR-21	Luciferase reporter assay, Western blot	22072622
24	TGFBR2	miR-21	Microarray	18591254
24	DDX3X	miR-21	Microarray	18591254
24	DDX3X	miR-181a	Sequencing	20371350
24	DDX3X	miR-181a	CLASH	23622248
24	ADAM10	miR-155	Proteomics	21030878
24	FLI1	miR-155	Western blot	18950466
24	FLI1	miR-155	Proteomics	20584899
24	TMOD3	miR-34a	Proteomics	21566225
24	TMOD3	miR-155	Proteomics	18668040
24	NFKB1	miR-21	ChIP-seq	21081469
24	NFKB1	miR-146b	Luciferase reporter assay	18504431
24	NFKB1	miR-155	Proteomics	21030878
24	NFKB1	miR-9	qRT-PCR, Luciferase reporter assay, Western blot	19702828
24	NFKB1	miR-9	GFP reporter assay, qRT-PCR, Western blot	20102618
24	NFKB1	miR-9	GFP reporter assay, Western blot, EGFP reporter assay, qRT-PCR	22131135
24	NFKB1	miR-9	Reporter assay, Western blot	19289835
24	PICALM	miR-155	pSILAC, Proteomics	18668040
24	PICALM	miR-155	Reporter assay, Other	20584899
24	LPXN	miR-9	Microarray	17612493
24	GRN	miR-9	Reporter assay	20479936
24	IL13RA1	miR-155	Luciferase reporter assay	21097505
25	PTX3	miR-21	Luciferase reporter assay	21131358
25	VEGFA	miR-21	qRT-PCR	19435867
25	VEGFA	miR-21	Western blot, qRT-PCR	21544242
25	VEGFA	miR-34a	ELISA, Luciferase reporter assay	18320040
25	VEGFA	miR-9	qRT-PCR, Other	20173740
25	VEGFA	miR-34b	ELISA, Luciferase reporter assay	18320040
25	VEGFA	miR-93	ELISA, Luciferase reporter assay	18320040
25	VEGFA	miR-20b	ELISA, Luciferase reporter assay	18320040
25	VEGFA	miR-106a	ELISA, Luciferase reporter assay	18320040
25	SOD2	miR-222	Flow, Luciferase reporter assay, Microarray, qRT-PCR, Western blot	19487542
25	CLIC4	miR-155	Proteomics	20584899
25	TGFB2	miR-21	Microarray	18591254
25	KLF6	miR-181a	Luciferase reporter assay, qRT-PCR, Western blot	22581522
25	CD44	miR-34a	Immunohistochemistry, qRT-PCR, Western blot, Reporter assay, Other	21240262
25	ZFP36L2	miR-181a	CLASH	23622248
25	EXOC5	miR-21	Microarray	18591254
25	STAT3	miR-21	Western blot, Other	20048743
25	STAT3	miR-21	Microarray	18591254
25	STAT3	miR-155	Proteomics	18668040
25	STAT3	miR-93	Sequencing	20371350
25	STAT3	miR-20b	qRT-PCR, ELISA, ChIP, Western blot	20232316

25	FAS	miR-21	Microarray	20048743
25	FAS	miR-106a	Luciferase reporter assay	22431000
25	CPD	miR-155	Proteomics	18668040
25	CPD	miR-19b	Sequencing	20371350
25	BCL6	miR-21	Luciferase reporter assay	23416424
25	BCL6	miR-155	Luciferase reporter assay	23041630
25	BCL6	miR-9	Luciferase reporter assay	19956200
25	ACTN1	miR-93	CLASH	23622248
25	CALD1	miR-21	Microarray	18591254
25	CALD1	miR-34a	Proteomics	21566225
25	IL13RA1	miR-155	Luciferase reporter assay	21097505
25	TLR2	miR-19b	Luciferase reporter assay, qRT-PCR, Western blot	22105995
25	TLR2	miR-19a	Luciferase reporter assay, qRT-PCR, Western blot	22105995
25	CAST	miR-222	CLASH	23622248
25	TGFBI	miR-21	Luciferase reporter assay, Western blot	19136465
25	TGFBI	miR-21	Microarray	18591254
25	TGFBI	miR-9	In situ hybridization, Luciferase reporter assay	21720722
25	BIRC3	miR-34a	Microarray, Northern blot	17540599
25	FLNA	miR-155	Proteomics	18668040
25	FLNA	miR-222	CLASH	23622248
25	FLNA	miR-221	CLASH	23622248
25	FLNA	miR-23a	CLASH	23622248
25	THBS1	miR-155	Proteomics	18668040
25	JUNB	miR-155	Proteomics	18668040
25	ICAM1	miR-21	Luciferase reporter assay, Microarray, qRT-PCR	21131358
25	ICAM1	miR-155	Luciferase reporter assay	19949084
25	ICAM1	miR-155	qRT-PCR	21310411
25	ICAM1	miR-222	Luciferase reporter assay	19949084
25	ICAM1	miR-221	Luciferase reporter assay, Western blot	20110463
25	ICAM1	miR-221	Luciferase reporter assay	19949084
25	PLAUR	miR-155	Proteomics	18668040
26	RTN3	miR-155	Proteomics	18668040
26	ELAVL4	miR-21	qRT-PCR	20584986
26	KIF1B	miR-9	Microarray	17612493
26	RABGAP1	miR-9	Microarray	17612493
26	RABGAP1	miR-21	Microarray	18591254
26	PHF16	miR-21	Microarray	18591254
26	ZNF248	miR-155	Reporter assay, Other	20584899
26	ALDH5A1	miR-155	Proteomics	18668040
26	NTRK3	miR-9	Luciferase reporter assay, Western blot	17483472
26	NTRK3	miR-9	Luciferase reporter assay	19188439
26	NTRK3	miR-9	Luciferase reporter assay	18973228
26	ZMYND11	miR-222	CLASH	23622248
26	PPP1R9A	miR-181a	CLASH	23622248
26	SPTBN1	miR-9	Microarray	17612493
26	RAPGEF2	miR-155	Reporter assay, Other	20584899
26	RAPGEF4	miR-98	Microarray	19088304
26	RNF2	miR-155	Proteomics	18668040
26	NOTCH1	miR-181a	Luciferase reporter assay, Western blot	22916024
26	APC	miR-21	Microarray	18591254
26	APC	miR-155	Luciferase reporter assay, Western blot	19136465
26	APC	miR-155	Proteomics	20584899
26	RBM8A	miR-340	Sequencing	20371350
26	PIK3R1	miR-21	Microarray	18591254
26	TCF12	miR-155	Reporter assay, Other	20584899
26	QKI	miR-222	CLASH	23622248
26	ICAM1	miR-21	Luciferase reporter assay, Microarray, qRT-PCR	21131358
26	ICAM1	miR-222	Luciferase reporter assay	19949084
26	ICAM1	miR-155	Luciferase reporter assay	19949084

26	ICAM1	miR-155	qRT-PCR	21310411
26	ICAM1	miR-98	Microarray	19088304
26	NEK1	miR-21	Microarray	18591254
26	PTPN22	miR-181a	Luciferase reporter assay, Western blot	17382377
26	NKX2-2	miR-9	Microarray	17612493
26	CHD9	miR-155	Reporter assay, Other	20584899
26	HIPK2	miR-181a	Immunoblot, Luciferase reporter assay, qRT-PCR	21274007
26	HIPK2	miR-222	CLASH	23622248
26	HDAC4	miR-22	Luciferase reporter assay, qRT-PCR, Western blot	20842113
26	HDAC4	miR-22	Luciferase reporter assay	23349832
26	BMPR2	miR-21	Luciferase reporter assay, qRT-PCR, Western blot	19578724
26	BMPR2	miR-21	Luciferase reporter assay, qRT-PCR	18829576
26	BMPR2	miR-21	Microarray	18591254
26	BMPR2	miR-181c	Luciferase reporter assay	23352489
26	DBN1	miR-222	CLASH	23622248
26	DBN1	miR-155	Proteomics	18668040
27	BIRC5	miR-93	CLASH	23622248
27	CENPF	miR-93	CLASH	23622248
27	CDC20	miR-93	CLASH	23622248
27	MAD2L1	miR-93	CLASH	23622248
27	KIF23	miR-106b	Microarray	17242205
28	TGFB1	miR-21	Microarray	18591254
28	CAPG	miR-34a	Proteomics	21566225
29	BIRC5	miR-93	CLASH	23622248
29	EZH2	miR-93	CLASH	23622248
30	IL13RA1	miR-155	Luciferase reporter assay	21097505
30	CAPG	miR-34a	Proteomics	21566225
30	GRN	miR-9	Reporter assay	20479936
30	SERPINB1	miR-34a	Proteomics	21566225
30	LYN	miR-222	CLASH	23622248
30	SYNGR2	miR-9	Sequencing	20371350
30	TLR2	miR-19b	Luciferase reporter assay, qRT-PCR, Western blot	22105995
30	MAFB	miR-155	Microarray	19193853
30	MGAT4A	miR-21	Microarray	18591254
30	SLC2A5	miR-9	Microarray	17612493
30	CCR1	miR-21	Luciferase reporter assay	21131358
30	SPI1	miR-34a	Luciferase reporter assay, Reporter assay	20598588
30	SPI1	miR-9	Microarray	17612493
30	SPI1	miR-155	Luciferase reporter assay	19386588
30	SPI1	miR-155	Reporter assay	20680360
30	SPI1	miR-155	Proteomics	18668040
30	CORO1A	miR-222	Western blot	21226887
31	CAST	miR-222	CLASH	23622248
31	S100A11	miR-155	Proteomics	18668040
31	LYN	miR-222	CLASH	23622248
33	PYGL	miR-155	Proteomics	18668040
33	PYGL	miR-93	CLASH	23622248
33	PTX3	miR-21	Luciferase reporter assay	21131358
33	PTRF	miR-342	CLASH	23622248
33	TAGLN2	miR-93	CLASH	23622248
33	DDB2	miR-155	Proteomics	18668040
33	RRBP1	miR-34a	Proteomics	21566225
33	FAS	miR-21	Microarray	20048743
33	FAS	miR-504	qRT-PCR	20542001
33	FAS	miR-106a	Luciferase reporter assay	22431000
33	OSBPL3	miR-21	Microarray	18591254
33	LDHA	miR-34a	Reporter assay, Proteomics	21566225
33	LDHA	miR-23a	CLASH	23622248
33	CLIC1	miR-221	CLASH	23622248

33	ANXA2	miR-155	Proteomics	18668040
33	CAST	miR-222	CLASH	23622248
33	CBR1	miR-93	CLASH	23622248
33	SORT1	miR-222	CLASH	23622248
33	SORT1	miR-34a	Proteomics	21566225
33	CTNNA1	miR-155	Proteomics	18668040
33	PLAUR	miR-155	Proteomics	18668040
33	PLAUR	miR-204	Microarray	21282569
33	VIM	miR-9	Western blot	20173740
33	DAG1	miR-222	CLASH	23622248
33	DAG1	miR-155	Proteomics	18668040
34	NASP	miR-29a	Luciferase reporter assay, Western blot	22080513
34	NASP	miR-29a	Luciferase reporter assay	22194605
34	HNRNPA1	miR-22	CLASH	23622248
34	HNRNPA1	miR-19b	Sequencing	20371350
34	GPATCH8	miR-19b	Sequencing	20371350
35	PYGL	miR-155	Proteomics	18668040
35	PLOD2	miR-221	Sequencing	20371350
35	VEGFA	miR-504	ELISA, Luciferase reporter assay	18320040
35	VEGFA	miR-21	qRT-PCR	19435867
35	VEGFA	miR-21	Western blot, qRT-PCR	21544242
35	VEGFA	miR-34a	ELISA, Luciferase reporter assay	18320040
35	VEGFA	miR-101	Sequencing	20371350
35	VEGFA	miR-34b	ELISA, Luciferase reporter assay	18320040
35	P4HB	miR-210	2DGE, immunoprecipitaion, Mass spectrometry, Microarray, qRT-PCR, Western blot	19826008
35	CALU	miR-155	Proteomics	18668040
35	PLOD3	miR-21	Luciferase reporter assay, Quantitative proteomic approach	19253296
35	GPI	miR-34a	Proteomics	21566225
35	CANX	miR-222	CLASH	23622248
35	CANX	miR-342	CLASH	23622248
35	GNB2	miR-23a	CLASH	23622248
35	NSUN5	miR-155	Proteomics	18668040
35	NSUN5	miR-222	CLASH	23622248
35	NSUN5	miR-34a	Proteomics	21566225
35	ZYX	miR-221	CLASH	23622248
35	IL1RAP	miR-204	Microarray	21282569
35	COL4A2	miR-155	Proteomics	18668040
35	PRKCSH	miR-342	CLASH	23622248
35	PLAUR	miR-155	Proteomics	18668040
35	PLAUR	miR-204	Microarray	21282569
36	RTN2	miR-20a	CLASH	23622248
36	YWHAQ	miR-93	CLASH	23622248
36	PAIP1	miR-20a	CLASH	23622248
36	STMN1	miR-9	Reporter assay, Western blot, qRT-PCR	20362537
36	STMN1	miR-223	Luciferase reporter assay, qRT-PCR, Western blot	18555017
36	STMN1	miR-223	Flow, Immunohistochemistry, Luciferase reporter assay, qRT-PCR, Western blot	22470493
36	SIRT1	miR-9	In situ hybridization, Luciferase reporter assay	21720722
36	SIRT1	miR-9	Western blot, qRT-PCR	20362537
36	RPS6KA5	miR-19a	CLASH	23622248
36	RPS6KA5	miR-93	CLASH	23622248
36	POLD3	miR-130b	CLASH	23622248
36	CDKN1B	miR-222	Luciferase reporter assay, Western blot, Western blot, Other, Northern blot	17569667
36	CDKN1B	miR-222	qRT-PCR, Luciferase reporter assay, Western blot	19153141
36	CDKN1B	miR-222	Western blot, Northern blot	19107213
36	CDKN1B	miR-222	Luciferase reporter assay, Western blot	19859555
36	CDKN1B	miR-222	Luciferase reporter assay, qRT-PCR, Western blot	18417445

36	CDKN1B	miR-222	Luciferase reporter assay	19150885
36	CDKN1B	miR-222	Luciferase reporter assay	18983236
36	CDKN1B	miR-222	Luciferase reporter assay	18246122
36	CDKN1B	miR-222	immunohistochemistry, Microarray, qRT-PCR	20012062
36	CDKN1B	miR-222	Northern blot, qRT-PCR, Western blot	20018759
36	CDKN1B	miR-222	Immunohistochemistry, In situ hybridization, Luciferase reporter assay, Northern blot, Western blot, Reporter assay, Western blot	19424584
36	CDKN1B	miR-222	Western blot, Reporter assay	17721077
36	CDKN1B	miR-222	Luciferase reporter assay	17627278
36	CDKN1B	miR-222	qRT-PCR, Western blot	18708351
36	CDKN1B	miR-222	qRT-PCR	23447020
36	CDKN1B	miR-222	Reporter assay	17914108
36	CDKN1B	miR-222	Reporter assay	18413744
36	SRPK1	miR-93	Sequencing	20371350
37	ASF1B	miR-130b	CLASH	23622248
37	ASF1B	miR-18a	CLASH	23622248
37	CHEK1	miR-15b	CLASH	23622248
37	SMCHD1	miR-18a	CLASH	23622248
37	KIF23	miR-106b	Microarray	17242205
37	KIF23	miR-25	CLASH	23622248
37	TMPO	miR-106b	CLASH	23622248
37	E2F8	miR-19b	Sequencing	20371350
37	RIF1	miR-93	CLASH	23622248
37	ATAD2	miR-106b	Microarray	17242205
37	KIF2C	miR-20a	CLASH	23622248
37	STIL	miR-20a	CLASH	23622248
37	BIRC5	miR-93	CLASH	23622248
37	MXD3	miR-18a	CLASH	23622248
37	CENPF	miR-93	CLASH	23622248
37	EZH2	miR-93	CLASH	23622248
37	EZH2	miR-25	Luciferase reporter assay, qRT-PCR, Western blot	22399519
37	HMMR	miR-93	CLASH	23622248
37	HMMR	miR-106b	CLASH	23622248
37	OIP5	miR-29c	Sequencing	20371350
37	MAD2L1	miR-93	CLASH	23622248
38	DDAH1	miR-21	Microarray	18591254
38	NOL4	miR-181a	Microarray	17612493
38	NKX2-2	miR-9	Microarray	17612493
38	APC	miR-21	Microarray	18591254
39	ALDH5A1	miR-155	Proteomics	18668040
39	NTRK3	miR-9	Luciferase reporter assay, Western blot	17483472
39	NTRK3	miR-9	Luciferase reporter assay	19188439
39	NTRK3	miR-9	Luciferase reporter assay	18973228
39	HIPK2	miR-181a	Immunoblot, Luciferase reporter assay, qRT-PCR	21274007
39	HIPK2	miR-222	CLASH	23622248
39	NEK1	miR-21	Microarray	18591254
40	EZH2	miR-93	CLASH	23622248
40	NASP	miR-93	CLASH	23622248
40	NASP	miR-29a	Luciferase reporter assay, Western blot	22080513
40	NASP	miR-29a	Luciferase reporter assay	22194605
40	MCM7	miR-93	CLASH	23622248
40	CENPF	miR-93	CLASH	23622248
40	TLK2	miR-93	CLASH	23622248
40	POLD3	miR-130b	CLASH	23622248
40	POLD3	miR-29a	CLASH	23622248
40	MCM3	miR-93	CLASH	23622248
40	WAC	miR-93	CLASH	23622248
40	TRIM28	miR-93	CLASH	23622248
41	SOD2	miR-222	Flow, Luciferase reporter assay, Microarray, qRT-PCR, Western blot	19487542

41	DSE	miR-21	Microarray	18591254
41	AIM1	miR-21	Microarray	18591254
41	CCL20	miR-21	ELISA, Immunofluorescence, Luciferase reporter assay	22001440
41	BIRC3	miR-34a	Microarray, Northern blot	17540599
41	SLC39A14	miR-9	Microarray	17612493
41	SLC39A14	miR-155	Proteomics	18668040
41	MAFB	miR-155	Microarray	19193853
41	GLIPR1	miR-155	Proteomics	18668040
41	SLC2A5	miR-9	Microarray	17612493
41	PLAUR	miR-155	Proteomics	18668040
41	CEBPB	miR-155	Luciferase reporter assay	18367535
41	CEBPB	miR-155	Microarray, qRT-PCR, Western blot	19193853
41	CEBPB	miR-155	Luciferase reporter assay, qRT-PCR, Western blot	20427544
41	CEBPB	miR-155	pSILAC, Proteomics	18668040
41	CEBPB	miR-155	Luciferase reporter assay	19596814
41	CEBPB	miR-155	ELISA, Immunoblot, Immunocytochemistry, Immunofluorescence, Luciferase reporter assay, qRT-PCR	19887047
41	CEBPB	miR-34a	Luciferase reporter assay, Reporter assay	20598588
41	TLR2	miR-19b	Luciferase reporter assay, qRT-PCR, Western blot	22105995
41	S100A11	miR-155	Proteomics	18668040
41	IL6	miR-155	Proteomics	20584899
41	IL6	miR-155	Proteomics	18668040
41	JUNB	miR-155	Proteomics	18668040
41	TGFBI	miR-21	Luciferase reporter assay, Western blot	19136465
41	TGFBI	miR-21	Microarray	18591254
41	TGFBI	miR-9	In situ hybridization, Luciferase reporter assay	21720722
41	TNFAIP3	miR-21	Luciferase reporter assay	21131358
42	PYGL	miR-155	Proteomics	18668040
42	ANXA2	miR-155	Proteomics	18668040
42	S100A11	miR-155	Proteomics	18668040
42	PLAUR	miR-155	Proteomics	18668040
43	BIRC5	miR-93	CLASH	23622248
43	CENPF	miR-93	CLASH	23622248
43	KIF23	miR-106b	Microarray	17242205
43	KIF23	miR-25	CLASH	23622248
43	TYMS	miR-18a	CLASH	23622248
43	PCNA	miR-18a	CLASH	23622248
43	RNASEH2A	miR-25	CLASH	23622248
43	GINS1	miR-18a	CLASH	23622248
43	MAD2L1	miR-93	CLASH	23622248
43	HN1	miR-106b	Microarray	17242205
43	CDC20	miR-93	CLASH	23622248
43	CDC20	miR-18a	CLASH	23622248
43	KPNA2	miR-93	CLASH	23622248
43	EZH2	miR-93	CLASH	23622248
43	EZH2	miR-25	Luciferase reporter assay, qRT-PCR, Western blot	22399519
44	ELAVL1	miR-93	CLASH	23622248
44	DHFR	miR-93	CLASH	23622248
44	ZNF76	miR-93	CLASH	23622248
44	MAD1L1	miR-20a	CLASH	23622248
44	ADRM1	miR-93	CLASH	23622248
44	DHX30	miR-93	CLASH	23622248
44	NONO	miR-93	CLASH	23622248
46	NASP	miR-29a	Luciferase reporter assay, Western blot	22080513
46	NASP	miR-29a	Luciferase reporter assay	22194605
46	ZER1	miR-19b	Sequencing	20371350
46	SUZ12	miR-19a	Luciferase reporter assay	23451058
47	CSNK2A1	miR-22	Sequencing	20371350
47	ABL1	miR-29a	Luciferase reporter assay	23428668

48	CAPG	miR-34a	Proteomics	21566225
48	FAH	miR-34a	Proteomics	21566225
48	CYBRD1	miR-21	Microarray	18591254
48	TRIM38	miR-21	Microarray	18591254
48	ANXA4	miR-34a	Proteomics	21566225
48	SERPINB1	miR-34a	Proteomics	21566225
48	LRP10	miR-222	CLASH	23622248
48	FAS	miR-21	Microarray	20048743
48	RRAS	miR-34a	Sequencing	20371350
48	LYN	miR-222	CLASH	23622248
48	LAMP2	miR-21	Microarray	18591254
48	CCR1	miR-21	Luciferase reporter assay	21131358
48	S100A11	miR-155	Proteomics	18668040
49	SPI1	miR-34a	Luciferase reporter assay, Reporter assay	20598588
49	CCR1	miR-21	Luciferase reporter assay	21131358
50	ANKRD46	miR-21	Immunoblot, Immunohistochemistry, Luciferase reporter assay, Microarray, qRT-PCR, Western blot	21219636
50	BMP7	miR-22	qRT-PCR, Luciferase reporter assay, Western blot	19011694
50	GTF2I	miR-21	Microarray	18591254
50	ARHGEF7	miR-20a	CLASH	23622248
50	GRIA2	miR-181b	Luciferase assay/RT-PCR, Reporter assay, Other	18184693
50	NOVA1	miR-155	Reporter assay, Other	20584899
50	DBN1	miR-222	CLASH	23622248
50	DBN1	miR-155	Proteomics	18668040
50	TCL1A	miR-181b	Luciferase reporter assay, Microarray, qRT-PCR, Western blot	17178851
50	TRIM24	miR-155	Proteomics	20584899
50	REV3L	miR-21	Microarray	18591254
50	REV3L	miR-93	Sequencing	20371350
50	NKX2-2	miR-9	Microarray	17612493
50	APBB2	miR-9	Microarray	17612493
50	APBB2	miR-93	CLASH	23622248
51	SERPINB1	miR-34a	Proteomics	21566225
51	TLR2	miR-19b	Luciferase reporter assay, qRT-PCR, Western blot	22105995
51	CCR1	miR-21	Luciferase reporter assay	21131358
51	CEBPA	miR-193a	CLASH	23622248
51	CORO1A	miR-222	Western blot	21226887
51	MAFB	miR-155	Microarray	19193853
51	LPXN	miR-9	Microarray	17612493
51	LYN	miR-222	CLASH	23622248
51	CAPG	miR-34a	Proteomics	21566225
52	PFN2	miR-7	Microarray, Microarray, Other	19073608
52	UCHL1	miR-181a	CLASH	23622248
52	HOXC6	miR-27a	Sequencing	20371350
52	SPTBN2	miR-222	CLASH	23622248
52	HIST1H1C	miR-34a	Proteomics	21566225
52	INA	miR-155	Proteomics	18668040
52	MYT1	miR-27a	Western blot, Luciferase reporter assay	18006846
52	MAGI1	miR-34a	Sequencing	20371350
52	RAP2A	miR-34a	CLASH	23622248
52	TFAP2A	miR-221	Sequencing	20371350
52	NOL4	miR-181a	Microarray	17612493
52	CLIC1	miR-221	CLASH	23622248
53	KIF1B	miR-9	Microarray	17612493
53	NKX2-2	miR-9	Microarray	17612493
54	TNFSF10	miR-222	Western blot	18246122
54	GATA6	miR-181a	Luciferase reporter assay	19585654
54	GATA6	miR-181a	Microarray	17612493
54	TGM2	miR-155	Proteomics	18668040
54	TGFBR2	miR-21	qRT-PCR, Western blot	19906824

54	TGFBR2	miR-21	qRT-PCR, Luciferase reporter assay, Western blot, Reporter assay	19816956
54	TGFBR2	miR-21	Luciferase reporter assay, qRT-PCR, Western blot	18829576
54	TGFBR2	miR-21	Luciferase reporter assay, Western blot	22072622
54	TGFBR2	miR-21	Microarray	18591254
33	KPNA2	miR-16	Proteomics	18668040
