# Supplementary material for: A Computational Approach to Identifying Gene-microRNA Modules in Cancer
Source: PLoS Comput Biol. 2015 Jan 22;11(1):e1004042. doi: 10.1371/journal.pcbi.1004042 (PMC4303261; doi:10.1371/journal.pcbi.1004042)
Supplement: S14 Table — The significant numbers of genes in each module are enriched in gene ontology biological process, and the significance is shown in ‘p-value’. ‘m’, ‘k’, and ‘x’ represent the number of genes in the corresponding GO term, the number of genes in the module, and the number of genes belonging to the GO term in the module, respectively. (PDF) [file pcbi.1004042.s021.pdf]

Table S14. Ovarian cancer modules with enriched GO terms.

Module ID	m	k	x	Gene Ontology	p-value	Genes	p-value
2	69	60	5	Hemopoiesis	2.28E-05	MMP9, BLNK, CD4, LCK, LYN	2.09E-03
2	74	60	5	Immune System Development	3.21E-05	MMP9, BLNK, CD4, LCK, LYN	2.27E-03
2	233	60	8	Defense Response	1.87E-05	CXCL11, CXCL9, BLNK, CXCL10, CLEC5A, LSP1, CCR5, TYROBP	1.93E-03
2	128	60	8	Behavior	2.11E-07	CXCL13, CXCL11, CXCL9, CXCL10, DOCK2, CCR5, ITGB2, PLAUR	4.34E-05
2	80	60	8	Locomotory Behavior	5.14E-09	CXCL13, CXCL11, CXCL9, CXCL10, DOCK2, CCR5, ITGB2, PLAUR	1.41E-06
2	34	60	3	Leukocyte Differentiation	6.30E-04	MMP9, CD4, LCK	2.78E-02
2	113	60	5	Inflammatory Response	2.41E-04	CXCL11, CXCL9, BLNK, CXCL10, CCR5	1.33E-02
2	261	60	10	Response To Chemical Stimulus	5.59E-07	CXCL13, CXCL11, CXCL9, CXCL10, DOCK2, CCR5, ITGB2, PLAUR, LCK, LYN	9.23E-05
2	207	60	11	Immune Response	4.87E-09	CXCL13, IL18, BLNK, CD96, POU2AF1, AIM2, PSMB10, LCP2, CCR5, ARHGDIB, CD74	1.41E-06
2	71	60	5	Hemopoietic Or Lymphoid Organ Development	2.62E-05	MMP9, BLNK, CD4, LCK, LYN	2.16E-03
2	290	60	17	Immune System Process	8.52E-14	CXCL13, MMP9, IL18, BLNK, CD96, POU2AF1, AIM2, PSMB10, LCP2, DOCK2, CCR5, ITGB2, ARHGDIB, CD4, CD74, LCK, LYN	7.03E-11
2	39	60	3	T Cell Activation	9.46E-04	IL18, CD4, LCK	3.49E-02
2	269	60	9	Response To External Stimulus	6.61E-06	CXCL13, CXCL11, CXCL9, BLNK, CXCL10, DOCK2, CCR5, ITGB2, PLAUR	7.79E-04
2	226	60	6	Protein Amino Acid Phosphorylation	8.79E-04	HCK, ITGB2, BTK, LCK, LYN, FGR	3.45E-02
2	168	60	5	Response To Wounding	1.46E-03	CXCL11, CXCL9, BLNK, CXCL10, CCR5	4.65E-02
2	29	60	3	Humoral Immune Response	3.92E-04	BLNK, POU2AF1, PSMB10	1.90E-02
2	253	60	6	Phosphorylation	1.57E-03	HCK, ITGB2, BTK, LCK, LYN, FGR	4.80E-02
2	52	60	5	Cellular Defense Response	5.63E-06	CXCL9, CLEC5A, LSP1, CCR5, TYROBP	7.74E-04
3	170	35	8	Cell Cycle Process	2.28E-08	PLK1, E2F1, KIF22, CDC6, TUBG1, PRC1, BUB1, CDK2	9.39E-06
3	134	35	7	Mitotic Cell Cycle	9.36E-08	PLK1, E2F1, KIF22, CDC6, PRC1, BUB1, CDK2	2.57E-05
3	153	35	6	Cell Cycle Phase	4.57E-06	PLK1, E2F1, KIF22, CDC6, BUB1, CDK2	9.42E-04
3	43	35	3	Cell Cycle Checkpoint Go 0000075	2.56E-04	CCNE2, CDC6, BUB1	2.64E-02
3	13	35	3	G1 Phase Of Mitotic Cell Cycle	6.31E-06	E2F1, CDC6, CDK2	1.04E-03
3	93	35	4	Dna Replication	1.45E-04	MCM2, CDC6, CDK2, MCM7	1.71E-02
3	15	35	3	G1 Phase	9.99E-06	E2F1, CDC6, CDK2	1.37E-03
3	276	35	10	Cell Cycle Go 0007049	3.76E-09	CCNE2, PLK1, MCM2, E2F1, KIF22, CDC6, TUBG1, PRC1, BUB1, CDK2	3.11E-06

3	57	35	3	Interphase Of Mitotic Cell Cycle	5.91E-04	E2F1, CDC6, CDK2	4.88E-02
6	234	34	7	Dna Metabolic Process	3.29E-06	CHEK1, POLE2, CDK2, PRIM1, FEN1, MCM2, MSH6	3.88E-04
6	134	34	4	Regulation Of Kinase Activity	5.25E-04	CHEK1, CCNE2, CDC25A, CDKN3	2.69E-02
6	133	34	4	Regulation Of Protein Kinase Activity	5.10E-04	CHEK1, CCNE2, CDC25A, CDKN3	2.69E-02
6	99	34	4	M Phase	1.65E-04	CHEK1, KIF15, KNTC1, BUB1	1.13E-02
6	136	34	4	Regulation Of Transferase Activity	5.55E-04	CHEK1, CCNE2, CDC25A, CDKN3	2.69E-02
6	63	34	4	Interphase	2.80E-05	CDC7, TIMELESS, CDK2, CDKN3	2.10E-03
6	170	34	10	Cell Cycle Process	2.36E-11	CHEK1, CDC7, TIMELESS, CDK2, KIF15, KNTC1, KIF23, BUB1, RACGAP1, CDKN3	9.74E-09
6	134	34	7	Mitotic Cell Cycle	7.56E-08	CDC7, CDK2, KIF15, KNTC1, KIF23, BUB1, CDKN3	1.04E-05
6	153	34	8	Cell Cycle Phase	7.76E-09	CHEK1, CDC7, TIMELESS, CDK2, KIF15, KNTC1, BUB1, CDKN3	1.60E-06
6	43	34	4	Cell Cycle Checkpoint Go 0000075	6.05E-06	CHEK1, CCNE2, KNTC1, BUB1	4.99E-04
6	93	34	5	Dna Replication	5.75E-06	POLE2, CDK2, PRIM1, MCM2, MSH6	4.99E-04
6	39	34	4	Regulation Of Cyclin Dependent Protein Kinase Activity	4.06E-06	CHEK1, CCNE2, CDC25A, CDKN3	4.19E-04
6	70	34	3	Mitosis	9.89E-04	KIF15, KNTC1, BUB1	3.89E-02
6	276	34	13	Cell Cycle Go 0007049	2.13E-13	CHEK1, CDC7, CCNE2, TIMELESS, CDK2, KIF15, KNTC1, CDC25A, MCM2, KIF23, BUB1, RACGAP1, CDKN3	1.75E-10
6	51	34	3	Dna Dependent Dna Replication	3.90E-04	CDK2, PRIM1, MSH6	2.48E-02
6	57	34	3	Interphase Of Mitotic Cell Cycle	5.42E-04	CDC7, CDK2, CDKN3	2.69E-02
6	150	34	4	Response To Dna Damage Stimulus	8.02E-04	CHEK1, POLE2, FEN1, MSH6	3.44E-02
6	157	34	9	Regulation Of Cell Cycle	3.43E-10	CHEK1, CDC7, CCNE2, TIMELESS, CDK2, KNTC1, CDC25A, BUB1, CDKN3	9.44E-08
6	72	34	3	M Phase Of Mitotic Cell Cycle	1.07E-03	KIF15, KNTC1, BUB1	4.03E-02
6	266	34	5	Regulation Of Cell Proliferation	8.34E-04	CHEK1, CDC7, TIMELESS, CDK2, CDKN3	3.44E-02
13	234	78	16	Dna Metabolic Process	1.47E-12	CDC6, EXO1, RAD1, POLE2, MCM7, SMARCB1, POLL, ORC2L, FANCG, DBF4, CHEK1, CEBPG, ORC5L, TFAM, ORC1L, GMNN	1.21E-09
13	118	78	6	Dna Repair	1.13E-04	EXO1, RAD1, POLE2, POLL, FANCG, CEBPG	5.47E-03
13	177	78	8	Response To Endogenous Stimulus	1.82E-05	EXO1, RAD1, POLE2, MCM7, POLL, FANCG, CHEK1, CEBPG	1.25E-03

13	99	78	7	M Phase	3.36E-06	BUB1, NCAPH, RAD1, PLK1, BIRC5, CHEK1, SMC4	2.77E-04
13	20	78	3	Dna Integrity Checkpoint	2.75E-04	CDC6, RAD1, CHEK1	1.26E-02
13	63	78	4	Interphase	7.26E-04	CDC6, BIRC5, DBF4, TIMELESS	3.13E-02
13	170	78	10	Cell Cycle Process	1.33E-07	BUB1, NCAPH, CDC6, RAD1, PLK1, BIRC5, DBF4, CHEK1, TIMELESS, SMC4	1.56E-05
13	134	78	7	Mitotic Cell Cycle	2.47E-05	BUB1, NCAPH, CDC6, PLK1, BIRC5, DBF4, SMC4	1.57E-03
13	153	78	10	Cell Cycle Phase	4.90E-08	BUB1, NCAPH, CDC6, RAD1, PLK1, BIRC5, DBF4, CHEK1, TIMELESS, SMC4	6.74E-06
13	43	78	7	Cell Cycle Checkpoint Go 0000075	9.72E-09	BUB1, CCNE2, CDC6, RAD1, FANCG, BIRC5, CHEK1	2.67E-06
13	15	78	4	Dna Replication Initiation	2.11E-06	CDC6, ORC2L, ORC5L, ORC1L	1.93E-04
13	93	78	10	Dna Replication	3.76E-10	CDC6, EXO1, POLE2, MCM7, ORC2L, DBF4, ORC5L, TFAM, ORC1L, GMNN	1.55E-07
13	70	78	5	Mitosis	8.73E-05	BUB1, NCAPH, PLK1, BIRC5, SMC4	4.80E-03
13	276	78	13	Cell Cycle Go 0007049	2.12E-08	BUB1, CCNE2, NCAPH, CDC6, RAD1, FANCG, PLK1, BIRC5, DBF4, CHEK1, TIMELESS, SMC4, GMNN	4.37E-06
13	51	78	7	Dna Dependent Dna Replication	3.35E-08	CDC6, EXO1, ORC2L, ORC5L, TFAM, ORC1L, GMNN	5.53E-06
13	150	78	8	Response To Dna Damage Stimulus	5.39E-06	EXO1, RAD1, POLE2, MCM7, POLL, FANCG, CHEK1, CEBPG	4.05E-04
13	157	78	9	Regulation Of Cell Cycle	7.32E-07	BUB1, CCNE2, CDC6, RAD1, FANCG, BIRC5, CHEK1, TIMELESS, GMNN	7.55E-05
13	72	78	5	M Phase Of Mitotic Cell Cycle	9.99E-05	BUB1, NCAPH, PLK1, BIRC5, SMC4	5.15E-03
18	234	31	9	Dna Metabolic Process	4.71E-09	CHEK1, MCM3, DMC1, NASP, MCM2, FEN1, EXO1, MCM7, FANCG	1.94E-06
18	177	31	5	Response To Endogenous Stimulus	8.08E-05	CHEK1, FEN1, EXO1, MCM7, FANCG	6.67E-03
18	99	31	4	M Phase	1.14E-04	CHEK1, KNTC1, DMC1, SMC4	8.55E-03
18	170	31	8	Cell Cycle Process	8.00E-09	CHEK1, KNTC1, DMC1, TIMELESS, SMC4, KIF23, RACGAP1, CDC7	2.20E-06
18	134	31	4	Mitotic Cell Cycle	3.65E-04	KNTC1, SMC4, KIF23, CDC7	2.15E-02
18	153	31	6	Cell Cycle Phase	2.16E-06	CHEK1, KNTC1, DMC1, TIMELESS, SMC4, CDC7	3.45E-04
18	43	31	4	Cell Cycle Checkpoint Go 0000075	4.13E-06	CHEK1, CCNE2, KNTC1, FANCG	4.26E-04
18	77	31	3	Response To Abiotic Stimulus	9.93E-04	TIMELESS, FEN1, TOPBP1	4.55E-02
18	93	31	5	Dna Replication	3.57E-06	MCM3, NASP, MCM2, EXO1, MCM7	4.21E-04
18	44	31	3	Dna Recombination	1.90E-04	CHEK1, DMC1, EXO1	1.21E-02

18	276	31	12	Cell Cycle Go 0007049	1.56E-12	CHEK1, CCNE2, KNTC1, DMC1, NASP, MCM2, TIMELESS, SMC4, KIF23, RACGAP1, FANCG, CDC7	1.29E-09
18	150	31	5	Response To Dna Damage Stimulus	3.67E-05	CHEK1, FEN1, EXO1, MCM7, FANCG	3.36E-03
18	157	31	6	Regulation Of Cell Cycle	2.51E-06	CHEK1, CCNE2, KNTC1, TIMELESS, FANCG, CDC7	3.45E-04
20	118	44	4	Dna Repair	8.79E-04	POLQ, EXO1, RAD51, HMGB2	4.27E-02
20	19	44	4	Mitotic Cell Cycle Checkpoint	5.77E-07	BUB1, CCNA2, MAD2L1, BUB1B	4.76E-05
20	177	44	5	Response To Endogenous Stimulus	4.43E-04	POLQ, EXO1, CCNA2, RAD51, HMGB2	2.43E-02
20	99	44	12	M Phase	1.11E-16	BUB1, PLK1, CENPE, CCNA2, RAD51, MAD2L1, NEK2, BIRC5, NCAPH, BUB1B, KIF15, AURKA	9.16E-14
20	33	44	6	Regulation Of Mitosis	1.72E-09	BUB1, CCNA2, MAD2L1, NEK2, BIRC5, BUB1B	1.77E-07
20	170	44	14	Cell Cycle Process	5.08E-14	BUB1, PLK1, CENPE, CCNA2, RAD51, MAD2L1, NEK2, BIRC5, NCAPH, BUB1B, KIF23, KIF15, RACGAP1, AURKA	8.01E-12
20	134	44	12	Mitotic Cell Cycle	4.67E-14	BUB1, PLK1, CENPE, CCNA2, MAD2L1, NEK2, BIRC5, NCAPH, BUB1B, KIF23, KIF15, AURKA	8.01E-12
20	153	44	12	Cell Cycle Phase	1.90E-13	BUB1, PLK1, CENPE, CCNA2, RAD51, MAD2L1, NEK2, BIRC5, NCAPH, BUB1B, KIF15, AURKA	2.23E-11
20	43	44	5	Cell Cycle Checkpoint Go 0000075	4.47E-07	BUB1, CCNA2, MAD2L1, BIRC5, BUB1B	4.10E-05
20	28	44	3	Chromosome Segregation	1.40E-04	CENPE, NCAPH, TOP2A	9.62E-03
20	70	44	11	Mitosis	2.09E-14	BUB1, PLK1, CENPE, CCNA2, MAD2L1, NEK2, BIRC5, NCAPH, BUB1B, KIF15, AURKA	8.01E-12
20	276	44	15	Cell Cycle Go 0007049	5.83E-14	BUB1, PLK1, CENPE, CCNA2, RAD51, MAD2L1, NEK2, BIRC5, NCAPH, BUB1B, KIF23, KIF15, CDC20, RACGAP1, AURKA	8.01E-12
20	51	44	3	Dna Dependent Dna Replication	8.39E-04	EXO1, RAD51, HMGB2	4.27E-02
20	150	44	5	Response To Dna Damage Stimulus	2.06E-04	POLQ, EXO1, CCNA2, RAD51, HMGB2	1.31E-02
20	157	44	6	Regulation Of Cell Cycle	2.09E-05	BUB1, CCNA2, MAD2L1, NEK2, BIRC5, BUB1B	1.57E-03
20	72	44	11	M Phase Of Mitotic Cell Cycle	3.64E-14	BUB1, PLK1, CENPE, CCNA2, MAD2L1, NEK2, BIRC5, NCAPH, BUB1B, KIF15, AURKA	8.01E-12
21	233	30	7	Defense Response	1.29E-06	CXCL11, CXCL9, CCR5, PTPRCAP, ITK, MNDA, CCR1	2.94E-04
21	128	30	5	Behavior	1.44E-05	CXCL13, CXCL11, CXCL9, CCR5, DOCK2	1.72E-03

21	80	30	5	Locomotory Behavior	1.43E-06	CXCL13, CXCL11, CXCL9, CCR5, DOCK2	2.94E-04
21	113	30	4	Inflammatory Response	1.67E-04	CXCL11, CXCL9, CCR5, CCR1	1.05E-02
21	88	30	4	Cellular Cation Homeostasis	6.31E-05	CXCL13, CCR5, LCK, CCR1	5.21E-03
21	261	30	6	Response To Chemical Stimulus	3.75E-05	CXCL13, CXCL11, CXCL9, CCR5, LCK, DOCK2	3.87E-03
21	207	30	8	Immune Response	2.82E-08	CXCL13, CD96, AIM2, CCR5, IL2RG, GZMA, LCP2, CCR1	1.16E-05
21	172	30	4	Homeostatic Process	8.24E-04	CXCL13, CCR5, LCK, CCR1	3.09E-02
21	115	30	4	Cellular Homeostasis	1.79E-04	CXCL13, CCR5, LCK, CCR1	1.05E-02
21	290	30	10	Immune System Process	1.10E-09	CXCL13, CD96, AIM2, CCR5, IL2RG, GZMA, LCP2, LCK, CCR1, DOCK2	9.10E-07
21	269	30	6	Response To External Stimulus	4.44E-05	CXCL13, CXCL11, CXCL9, CCR5, CCR1, DOCK2	4.07E-03
21	130	30	4	Chemical Homeostasis	2.86E-04	CXCL13, CCR5, LCK, CCR1	1.42E-02
21	107	30	4	Ion Homeostasis	1.35E-04	CXCL13, CCR5, LCK, CCR1	9.29E-03
21	55	30	3	Apoptotic Program	3.35E-04	GZMA, GZMB, LCK	1.46E-02
21	91	30	4	Cation Homeostasis	7.20E-05	CXCL13, CCR5, LCK, CCR1	5.40E-03
21	168	30	4	Response To Wounding	7.55E-04	CXCL11, CXCL9, CCR5, CCR1	3.09E-02
21	52	30	4	Cellular Defense Response	7.80E-06	CXCL9, CCR5, ITK, MNDA	1.29E-03
22	163	27	5	Proteolysis	2.71E-05	MMP11, MMP1, CTSK, PLAU, MMP2	2.24E-02
25	234	29	8	Dna Metabolic Process	5.44E-08	CHEK1, POLE2, MCM2, TIPIN, FEN1, MCM7, KPNA2, EXO1	8.98E-06
25	177	29	5	Response To Endogenous Stimulus	5.78E-05	CHEK1, POLE2, FEN1, MCM7, EXO1	3.41E-03
25	99	29	5	M Phase	3.44E-06	CHEK1, KIF15, SMC4, KPNA2, NDC80	3.24E-04
25	74	29	4	Microtubule Based Process	2.78E-05	KIF4A, KPNA2, PRC1, KIF23	1.91E-03
25	63	29	4	Interphase	1.46E-05	CDC7, TIPIN, KPNA2, TIMELESS	1.21E-03
25	170	29	12	Cell Cycle Process	2.00E-15	CHEK1, FBXO5, CDC7, TIPIN, KIF15, SMC4, KPNA2, TIMELESS, PRC1, RACGAP1, KIF23, NDC80	8.24E-13
25	134	29	8	Mitotic Cell Cycle	6.72E-10	FBXO5, CDC7, KIF15, SMC4, KPNA2, PRC1, KIF23, NDC80	1.85E-07
25	153	29	8	Cell Cycle Phase	1.93E-09	CHEK1, CDC7, TIPIN, KIF15, SMC4, KPNA2, TIMELESS, NDC80	3.99E-07
25	13	29	3	S Phase	3.53E-06	CDC7, TIPIN, TIMELESS	3.24E-04
25	43	29	3	Cell Cycle Checkpoint Go 0000075	1.45E-04	CHEK1, CCNE2, TIPIN	6.76E-03
25	93	29	5	Dna Replication	2.53E-06	POLE2, MCM2, TIPIN, MCM7, EXO1	2.98E-04
25	44	29	3	Dna Recombination	1.56E-04	CHEK1, KPNA2, EXO1	6.76E-03
25	70	29	3	Mitosis	6.17E-04	KIF15, SMC4, NDC80	2.12E-02
25	276	29	14	Cell Cycle Go 0007049	4.44E-16	CHEK1, FBXO5, CDC7, CCNE2, MCM2, TIPIN, KIF15, SMC4, KPNA2, TIMELESS, PRC1, RACGAP1, KIF23, NDC80	3.66E-13
25	181	29	5	Cytoskeleton Organization And Biogenesis	6.43E-05	KIF4A, KPNA2, PRC1, RACGAP1, KIF23	3.54E-03

25	150	29	5	Response To Dna Damage Stimulus	2.62E-05	CHEK1, POLE2, FEN1, MCM7, EXO1	1.91E-03
25	157	29	6	Regulation Of Cell Cycle	1.65E-06	CHEK1, FBXO5, CDC7, CCNE2, TIPIN, TIMELESS	2.27E-04
25	72	29	3	M Phase Of Mitotic Cell Cycle	6.70E-04	KIF15, SMC4, NDC80	2.13E-02
26	234	44	8	Dna Metabolic Process	1.75E-06	FEN1, KPNA2, CHEK1, RPA3, XRCC4, GMNN, RAN, RFC4	2.06E-04
26	99	44	7	M Phase	6.12E-08	PLK1, BIRC5, CCNA2, MAD2L1, KPNA2, CHEK1, RAN	1.01E-05
26	33	44	3	Regulation Of Mitosis	2.30E-04	BIRC5, CCNA2, MAD2L1	1.58E-02
26	63	44	4	Interphase	7.89E-05	BIRC5, TIMELESS, KPNA2, CDC7	5.92E-03
26	170	44	10	Cell Cycle Process	3.96E-10	FBXO5, PLK1, BIRC5, CCNA2, MAD2L1, TIMELESS, KPNA2, CHEK1, RAN, CDC7	3.26E-07
26	134	44	8	Mitotic Cell Cycle	2.41E-08	FBXO5, PLK1, BIRC5, CCNA2, MAD2L1, KPNA2, RAN, CDC7	4.97E-06
26	153	44	9	Cell Cycle Phase	3.31E-09	PLK1, BIRC5, CCNA2, MAD2L1, TIMELESS, KPNA2, CHEK1, RAN, CDC7	9.09E-07
26	43	44	4	Cell Cycle Checkpoint Go 0000075	1.72E-05	BIRC5, CCNA2, MAD2L1, CHEK1	1.42E-03
26	70	44	5	Mitosis	5.23E-06	PLK1, BIRC5, CCNA2, MAD2L1, RAN	5.39E-04
26	276	44	11	Cell Cycle Go 0007049	2.95E-09	FBXO5, PLK1, BIRC5, CCNA2, MAD2L1, TIMELESS, KPNA2, CHEK1, GMNN, RAN, CDC7	9.09E-07
26	157	44	8	Regulation Of Cell Cycle	8.31E-08	FBXO5, BIRC5, CCNA2, MAD2L1, TIMELESS, CHEK1, GMNN, CDC7	1.14E-05
26	72	44	5	M Phase Of Mitotic Cell Cycle	6.01E-06	PLK1, BIRC5, CCNA2, MAD2L1, RAN	5.51E-04
27	90	55	8	Skeletal Development	6.48E-09	COL11A1, INHBA, TWIST1, COMP, FBN1, SPARC, AEBP1, COL1A2	1.78E-06
31	26	65	3	Jak Stat Cascade	3.57E-04	STAT1, STAT3, LYN	4.21E-02
31	128	65	6	Behavior	6.34E-05	CXCL10, CXCL11, PTAFR, ITGB2, CCL5, CXCL9	1.74E-02
31	80	65	6	Locomotory Behavior	4.30E-06	CXCL10, CXCL11, PTAFR, ITGB2, CCL5, CXCL9	3.12E-03
31	113	65	5	Inflammatory Response	3.51E-04	CXCL10, CXCL11, PTAFR, CCL5, CXCL9	4.21E-02
31	22	65	3	Peptidyl Tyrosine Phosphorylation	2.15E-04	STAT1, ITGB2, LYN	3.54E-02
31	47	65	4	Peptidyl Amino Acid Modification	1.16E-04	STAT1, TPST2, ITGB2, LYN	2.39E-02
31	24	65	4	Peptidyl Tyrosine Modification	7.57E-06	STAT1, TPST2, ITGB2, LYN	3.12E-03
33	19	57	4	Mitotic Cell Cycle Checkpoint	1.66E-06	BUB1, TTK, BUB1B, CCNA2	6.25E-05
33	13	57	4	Cytokinesis	3.12E-07	RACGAP1, PRC1, NUSAP1, BIRC5	1.36E-05

33	99	57	18	M Phase	3.18E-14	BUB1, TTK, ESPL1, PLK1, NDC80, AURKA, KIF11, SMC4, TPX2, BUB1B, KIF15, UBE2C, NUSAP1, CCNA2, RAD54L, NCAPH, BIRC5, KPNA2	5.24E-12
33	74	57	7	Microtubule Based Process	5.12E-08	TTK, KIF11, KIF23, PRC1, NUSAP1, KIF4A, KPNA2	2.64E-06
33	33	57	7	Regulation Of Mitosis	1.41E-10	BUB1, TTK, BUB1B, UBE2C, NUSAP1, CCNA2, BIRC5	1.30E-08
33	31	57	5	Microtubule Cytoskeleton Organization And Biogenesis	3.07E-07	TTK, KIF11, KIF23, PRC1, NUSAP1	1.36E-05
33	170	57	23	Cell Cycle Process	5.44E-14	BUB1, FBXO5, TTK, ESPL1, PLK1, NDC80, AURKA, RACGAP1, KIF11, KIF23, SMC4, PRC1, TPX2, BUB1B, KIF15, UBE2C, CENPF, NUSAP1, CCNA2, RAD54L, NCAPH, BIRC5, KPNA2	6.41E-12
33	15	57	5	Mitotic Sister Chromatid Segregation	5.75E-09	ESPL1, NDC80, SMC4, NUSAP1, NCAPH	4.75E-07
33	134	57	21	Mitotic Cell Cycle	5.23E-14	BUB1, FBXO5, TTK, ESPL1, PLK1, NDC80, AURKA, KIF11, KIF23, SMC4, PRC1, TPX2, BUB1B, KIF15, UBE2C, CENPF, NUSAP1, CCNA2, NCAPH, BIRC5, KPNA2	6.41E-12
33	153	57	19	Cell Cycle Phase	1.15E-14	BUB1, TTK, ESPL1, PLK1, NDC80, AURKA, KIF11, SMC4, TPX2, BUB1B, KIF15, UBE2C, CENPF, NUSAP1, CCNA2, RAD54L, NCAPH, BIRC5, KPNA2	3.18E-12
33	43	57	5	Cell Cycle Checkpoint Go 0000075	1.67E-06	BUB1, TTK, BUB1B, CCNA2, BIRC5	6.25E-05
33	28	57	7	Chromosome Segregation	3.99E-11	ESPL1, NDC80, SMC4, CENPF, NUSAP1, TOP2A, NCAPH	4.12E-09
33	15	57	3	Establishment Of Organelle Localization	4.39E-05	CENPF, NUSAP1, BIRC5	1.39E-03
33	15	57	4	Cell Division	5.92E-07	RACGAP1, PRC1, NUSAP1, BIRC5	2.44E-05
33	44	57	3	Dna Recombination	1.16E-03	EXO1, RAD54L, KPNA2	3.30E-02
33	8	57	4	Mitotic Spindle Organization And Biogenesis	3.11E-08	TTK, KIF11, KIF23, PRC1	1.98E-06
33	70	57	16	Mitosis	0.00E+00	BUB1, TTK, ESPL1, PLK1, NDC80, AURKA, KIF11, SMC4, TPX2, BUB1B, KIF15, UBE2C, NUSAP1, CCNA2, NCAPH, BIRC5	0.00E+00
33	276	57	25	Cell Cycle Go 0007049	2.81E-14	BUB1, FBXO5, TTK, ESPL1, PLK1, NDC80, AURKA, RACGAP1, KIF11, KIF23, SMC4, PRC1, TPX2, BUB1B, KIF15, UBE2C, CENPF, NUSAP1, CCNA2, CDC20, RAD54L, CKS2, NCAPH, BIRC5, KPNA2	5.24E-12

33	16	57	5	Sister Chromatid Segregation	8.34E-09	ESPL1, NDC80, SMC4, NUSAP1, NCAPH	6.25E-07
33	19	57	3	Organelle Localization	9.23E-05	CENPF, NUSAP1, BIRC5	2.82E-03
33	181	57	8	Cytoskeleton Organization And Biogenesis	1.97E-06	TTK, RACGAP1, KIF11, KIF23, PRC1, NUSAP1, KIF4A, KPNA2	7.05E-05
33	105	57	6	Chromosome Organization And Biogenesis	9.63E-06	ESPL1, NDC80, SMC4, NUSAP1, TOP2A, NCAPH	3.18E-04
33	27	57	4	Dna Packaging	7.30E-06	SMC4, NUSAP1, TOP2A, NCAPH	2.51E-04
33	9	57	4	Spindle Organization And Biogenesis	5.58E-08	TTK, KIF11, KIF23, PRC1	2.71E-06
33	157	57	9	Regulation Of Cell Cycle	4.56E-08	BUB1, FBXO5, TTK, BUB1B, UBE2C, NUSAP1, CCNA2, CKS2, BIRC5	2.51E-06
33	72	57	16	M Phase Of Mitotic Cell Cycle	0.00E+00	BUB1, TTK, ESPL1, PLK1, NDC80, AURKA, KIF11, SMC4, TPX2, BUB1B, KIF15, UBE2C, NUSAP1, CCNA2, NCAPH, BIRC5	0.00E+00
33	8	57	4	Chromosome Condensation	3.11E-08	SMC4, NUSAP1, TOP2A, NCAPH	1.98E-06
