# Supplementary material for: A Computational Approach to Identifying Gene-microRNA Modules in Cancer
Source: PLoS Comput Biol. 2015 Jan 22;11(1):e1004042. doi: 10.1371/journal.pcbi.1004042 (PMC4303261; doi:10.1371/journal.pcbi.1004042)
Supplement: S15 Table — The significant numbers of genes in each module are enriched in KEGG pathway, and the significance is shown in ‘p-value’. ‘m’, ‘k’, and ‘x’ represent the number of genes in the corresponding KEGG pathway, the number of genes in the module, and the number of genes belonging to the pathway in the module, respectively. (PDF) [file pcbi.1004042.s022.pdf]

Table S15. Ovarian cancer modules with enriched pathways in KEGG.

Module ID	m	k	x	Gene Ontology	p-value	Genes	p-value
2	218	60	8	Cytokine Cytokine Receptor Interaction	1.16E-05	CXCL13, LTB, CXCL11, IL18, CXCL9, CD27, CXCL10, CCR5	7.16E-04
2	156	60	10	Chemokine Signaling Pathway	4.31E-09	CXCL13, CXCL11, CXCL9, VAV1, CXCL10, HCK, DOCK2, CCR5, LYN, FGR	8.02E-07
2	116	60	4	Cell Adhesion Molecules Cams	2.63E-03	ICOS, SIGLEC1, ITGB2, CD4	4.45E-02
2	90	60	4	Toll Like Receptor Signaling Pathway	1.03E-03	CXCL11, CXCL9, CXCL10, SPP1	1.92E-02
2	42	60	4	Cytosolic Dna Sensing Pathway	5.41E-05	IL18, CXCL10, AIM2, CASP1	2.52E-03
2	108	60	5	Natural Killer Cell Mediated Cytotoxicity	1.95E-04	VAV1, LCP2, ITGB2, TYROBP, LCK	6.05E-03
2	103	60	5	T Cell Receptor Signaling Pathway	1.56E-04	ICOS, VAV1, LCP2, CD4, LCK	5.81E-03
2	68	60	4	B Cell Receptor Signaling Pathway	3.57E-04	BLNK, VAV1, BTK, LYN	9.50E-03
2	72	60	4	Fc Epsilon Ri Signaling Pathway	4.45E-04	VAV1, LCP2, BTK, LYN	1.03E-02
2	84	60	4	Fc Gamma R Mediated Phagocytosis	7.98E-04	VAV1, HCK, DOCK2, LYN	1.65E-02
2	33	60	5	Primary Immunodeficiency	5.53E-07	BLNK, ICOS, BTK, CD4, LCK	5.14E-05
3	32	35	4	Dna Replication	2.03E-06	MCM2, MCM4, POLA2, MCM7	1.89E-04
3	111	35	11	Cell Cycle	0.00E+00	CCNE2, PLK1, MCM2, E2F1, CDC6, MCM4, BUB1, TFDP2, CDK2, MCM7, CCNB2	0.00E+00
3	92	35	5	Oocyte Meiosis	6.32E-06	CCNE2, PLK1, BUB1, CDK2, CCNB2	3.92E-04
3	60	35	3	P53 Signaling Pathway	6.87E-04	CCNE2, CDK2, CCNB2	2.56E-02
3	73	35	4	Progesterone Mediated Oocyte Maturation	5.65E-05	PLK1, BUB1, CDK2, CCNB2	2.62E-03
6	32	34	8	Dna Replication	3.66E-14	POLE2, MCM4, MCM6, RFC5, PRIM1, FEN1, MCM2, PCNA	6.81E-12
6	31	34	4	Base Excision Repair	1.58E-06	POLE2, FEN1, PCNA, PARP2	9.79E-05
6	41	34	3	Nucleotide Excision Repair	2.04E-04	POLE2, RFC5, PCNA	7.58E-03
6	23	34	3	Mismatch Repair	3.50E-05	RFC5, MSH6, PCNA	1.63E-03
6	111	34	10	Cell Cycle	3.20E-13	CHEK1, CDC7, CCNE2, MCM4, CDK2, MCM6, CDC25A, MCM2, PCNA, BUB1	2.97E-11
6	60	34	3	P53 Signaling Pathway	6.30E-04	CHEK1, CCNE2, CDK2	1.95E-02
6	73	34	3	Progesterone Mediated Oocyte Maturation	1.12E-03	CDK2, CDC25A, BUB1	2.97E-02
8	78	39	4	Tgf Beta Signaling Pathway	1.13E-04	INHBA, COMP, THBS2, THBS1	6.99E-03
8	183	39	11	Focal Adhesion	8.60E-12	MYLK, COMP, ITGB1, THBS2, THBS1, COL3A1, COL1A1, FN1, PDGFRB, COL1A2, ITGA5	8.00E-10
8	78	39	9	Ecm Receptor Interaction	2.33E-12	COMP, ITGB1, THBS2, THBS1, COL3A1, COL1A1, FN1, COL1A2, ITGA5	4.33E-10

8	65	39	3	Complement And Coagulation Cascades	1.19E-03	SERPINE1, PLAU, PLAUR	4.44E-02
8	182	39	5	Regulation Of Actin Cytoskeleton	2.83E-04	MYLK, ITGB1, FN1, PDGFRB, ITGA5	1.32E-02
12	43	33	3	Rna Degradation	2.15E-04	EXOSC5, LSM2, LSM3	1.06E-02
12	32	33	4	Dna Replication	1.59E-06	PRIM1, RFC5, FEN1, MCM2	2.96E-04
12	98	33	4	Spliceosome	1.41E-04	SNRPE, LSM2, CDC5L, LSM3	1.06E-02
12	111	33	4	Cell Cycle	2.28E-04	E2F3, MCM2, STAG2, TFDP2	1.06E-02
13	32	78	4	Dna Replication	5.10E-05	POLE2, MCM7, PCNA, MCM6	4.75E-03
13	111	78	15	Cell Cycle	3.29E-14	BUB1, CCNE2, CDC6, TFDP2, HDAC1, MCM7, ORC2L, PLK1, DBF4, PCNA, CHEK1, MCM6, ORC5L, ORC1L, BUB3	6.11E-12
18	32	31	7	Dna Replication	1.15E-12	MCM4, MCM6, RFC5, MCM3, MCM2, FEN1, MCM7	2.14E-10
18	111	31	8	Cell Cycle	2.67E-10	CHEK1, CCNE2, MCM4, MCM6, MCM3, MCM2, MCM7, CDC7	2.48E-08
20	111	44	8	Cell Cycle	5.43E-09	BUB1, PLK1, CCNA2, MAD2L1, CCNB1, BUB1B, CCNB2, CDC20	1.01E-06
20	92	44	7	Oocyte Meiosis	3.67E-08	BUB1, PLK1, MAD2L1, CCNB1, CCNB2, CDC20, AURKA	3.41E-06
20	73	44	6	Progesterone Mediated Oocyte Maturation	2.37E-07	BUB1, PLK1, CCNA2, MAD2L1, CCNB1, CCNB2	1.47E-05
21	218	30	8	Cytokine Cytokine Receptor Interaction	4.22E-08	CXCL13, CXCL11, CXCL9, CD27, CCR5, IL2RG, IL2RB, CCR1	3.92E-06
21	156	30	8	Chemokine Signaling Pathway	3.05E-09	CXCL13, CXCL11, CXCL9, CCR5, ITK, CCR1, DOCK2, HCK	5.66E-07
21	103	30	4	T Cell Receptor Signaling Pathway	1.17E-04	ICOS, LCP2, ITK, LCK	5.42E-03
21	33	30	4	Primary Immunodeficiency	1.22E-06	ICOS, IL2RG, LCK, BTK	7.55E-05
22	183	27	8	Focal Adhesion	4.23E-09	COL5A3, COL1A1, COL6A1, COL5A1, THBS2, FN1, ITGA5, COL3A1	3.94E-07
22	78	27	8	Ecm Receptor Interaction	4.31E-12	COL5A3, COL1A1, COL6A1, COL5A1, THBS2, FN1, ITGA5, COL3A1	8.02E-10
25	32	29	7	Dna Replication	6.94E-13	POLE2, MCM2, MCM6, PCNA, FEN1, MCM7, MCM4	1.29E-10
25	31	29	3	Base Excision Repair	5.39E-05	POLE2, PCNA, FEN1	3.34E-03
25	111	29	9	Cell Cycle	2.97E-12	CHEK1, CDC7, CCNE2, MCM2, MCM6, PCNA, MCM7, CCNB2, MCM4	2.76E-10
25	92	29	3	Oocyte Meiosis	1.37E-03	FBXO5, CCNE2, CCNB2	4.26E-02
25	60	29	3	P53 Signaling Pathway	3.92E-04	CHEK1, CCNE2, CCNB2	1.82E-02
26	32	44	6	Dna Replication	1.41E-09	FEN1, PCNA, MCM4, RPA3, RFC5, RFC4	2.62E-07
26	41	44	4	Nucleotide Excision Repair	1.42E-05	PCNA, RPA3, RFC5, RFC4	6.62E-04
26	23	44	4	Mismatch Repair	1.30E-06	PCNA, RPA3, RFC5, RFC4	8.09E-05

26	111	44	8	Cell Cycle	5.43E-09	PLK1, CCNA2, MAD2L1, PCNA, CCNB1, MCM4, CHEK1, CDC7	5.05E-07
26	92	44	4	Oocyte Meiosis	3.43E-04	FBXO5, PLK1, MAD2L1, CCNB1	1.06E-02
26	73	44	4	Progesterone Mediated Oocyte Maturation	1.40E-04	PLK1, CCNA2, MAD2L1, CCNB1	5.22E-03
27	78	55	5	Tgf Beta Signaling Pathway	2.71E-05	INHBA, DCN, COMP, THBS2, THBS1	1.68E-03
27	183	55	13	Focal Adhesion	1.21E-12	COL11A1, COL5A2, COL1A1, LAMB1, COMP, COL5A1, COL4A1, THBS2, COL3A1, PDGFRB, THBS1, FN1, COL1A2	1.12E-10
27	78	55	12	Ecm Receptor Interaction	6.13E-14	COL11A1, COL5A2, COL1A1, LAMB1, COMP, COL5A1, COL4A1, THBS2, COL3A1, THBS1, FN1, COL1A2	1.14E-11
31	42	65	3	Proteasome	1.48E-03	PSMB9, PSMB10, PSME2	3.94E-02
31	218	65	6	Cytokine Cytokine Receptor Interaction	1.11E-03	CXCL10, CXCL11, TNFSF10, ACVR2B, CCL5, CXCL9	3.46E-02
31	156	65	7	Chemokine Signaling Pathway	1.98E-05	STAT1, STAT3, CXCL10, CXCL11, LYN, CCL5, CXCL9	1.85E-03
31	108	65	5	Lysosome	2.85E-04	CTSO, CTSD, CTSB, CTSL1, LAPTM5	1.32E-02
31	67	65	4	Antigen Processing And Presentation	4.59E-04	CTSB, CTSL1, RFXAP, PSME2	1.71E-02
31	90	65	6	Toll Like Receptor Signaling Pathway	8.54E-06	STAT1, CXCL10, CXCL11, SPP1, CCL5, CXCL9	1.59E-03
31	42	65	4	Cytosolic Dna Sensing Pathway	7.42E-05	PYCARD, CXCL10, CASP1, CCL5	4.60E-03
33	111	57	8	Cell Cycle	4.59E-08	BUB1, TTK, ESPL1, PLK1, BUB1B, CCNA2, CDC20, CCNB2	8.53E-06
33	92	57	7	Oocyte Meiosis	2.34E-07	BUB1, FBXO5, ESPL1, PLK1, AURKA, CDC20, CCNB2	2.17E-05
33	73	57	4	Progesterone Mediated Oocyte Maturation	3.85E-04	BUB1, PLK1, CCNA2, CCNB2	2.39E-02
