# Supplementary material for: A Computational Approach to Identifying Gene-microRNA Modules in Cancer
Source: PLoS Comput Biol. 2015 Jan 22;11(1):e1004042. doi: 10.1371/journal.pcbi.1004042 (PMC4303261; doi:10.1371/journal.pcbi.1004042)
Supplement: S16 Table — The significant numbers of genes in each module are enriched in BioCarta pathways, and the significance is shown in ‘p-value’. ‘m’, ‘k’, and ‘x’ represent the number of genes in the corresponding BioCarta pathway, the number of genes in the module, and the number of genes belonging to the BioCarta pathway in the module, respectively. (PDF) [file pcbi.1004042.s023.pdf]

Table S16. Ovarian cancer modules with enriched pathways in BioCarta.

Module ID	m	k	x	Gene Ontology	p-value	Genes	p-value
2	31	60	4	Bcr Pathway	1.58E-05	BLNK, VAV1, BTK, LYN	3.44E-03
3	18	35	5	Mcm Pathway	1.28E-09	MCM2, CDC6, MCM4, CDK2, MCM7	2.79E-07
5	55	30	3	Hivnef Pathway	3.35E-04	DFFB, PTK2, CASP7	3.64E-02
6	18	34	4	Mcm Pathway	1.58E-07	MCM4, CDK2, MCM6, MCM2	3.42E-05
13	18	78	6	Mcm Pathway	1.06E-09	CDC6, MCM7, ORC2L, MCM6, ORC5L, ORC1L	2.30E-07
13	20	78	4	Atrbrca Pathway	7.29E-06	RAD1, FANCE, FANCG, CHEK1	7.91E-04
18	18	31	5	Mcm Pathway	6.74E-10	MCM4, MCM6, MCM3, MCM2, MCM7	1.46E-07
21	21	30	3	Il2 Pathway	1.80E-05	IL2RG, IL2RB, LCK	1.95E-03
21	20	30	3	Ctla4 Pathway	1.55E-05	ICOS, ITK, LCK	1.95E-03
25	18	29	4	Mcm Pathway	8.11E-08	MCM2, MCM6, MCM7, MCM4	1.76E-05
26	23	44	3	G2 Pathway	7.66E-05	PLK1, CCNB1, CHEK1	8.31E-03
26	9	44	3	Ranms Pathway	3.77E-06	KPNA2, RAN, RANBP1	8.17E-04
33	9	57	4	Ranms Pathway	5.58E-08	AURKA, TPX2, KIF15, KPNA2	1.21E-05
