# Supplementary material for: A Computational Approach to Identifying Gene-microRNA Modules in Cancer
Source: PLoS Comput Biol. 2015 Jan 22;11(1):e1004042. doi: 10.1371/journal.pcbi.1004042 (PMC4303261; doi:10.1371/journal.pcbi.1004042)
Supplement: S17 Table — (PDF) [file pcbi.1004042.s024.pdf]

Table S17. Literature evidences for ovarian cancer-related genes from ovarian cancer modules 22 and 8, and GBM related genes from GBM module 22.

Genes	PubMed ID
ITGA5	21119598
ITGB1	7542622
MMP11	18208802
MMP19	22902510
CALB2	15802873
VCAN	20204274
LOXL4	19015874
ADAM12	21617380
THBS2	18955754
FAP	22614695
COL6A1	21463610
COL1A1	22249249
CTSK	21463610
TIMP3	18344519
THBS1	22136063
RECK	21432940
MYLK	22759382
HNT	16115914

Genes	PubMed ID
ITGA5	21119598
MMP11	18208802
ECM1	19422301
GREM1	19123201
MMP19	22902510
VCAN	20204274
LOXL4	19015874
ADAM12	21617380
THBS2	18955754
FAP	22614695
COL6A1	21463610
COL1A1	22249249
CTSK	21463610

Genes	PubMed ID
FGFR	22837387
PTK2B	15967096
FAS	9600216
JUN	17321721
PXN	18813807
ITGA2	10744032
BAX	11912183
FGF2	8381111
AHSG	18281421
BCL2L1	16187019
CFLAR	12635660
MCL1	9060818
ITGA3	18712382
FGFR4	21555372
CD63	20150644
PDGFD	20406896
FGF14	17018610
EFNB2	19728339

SP100	21274506
