# Supplementary material for: A Computational Approach to Identifying Gene-microRNA Modules in Cancer
Source: PLoS Comput Biol. 2015 Jan 22;11(1):e1004042. doi: 10.1371/journal.pcbi.1004042 (PMC4303261; doi:10.1371/journal.pcbi.1004042)
Supplement: S18 Table — The significant numbers of genes in each module are enriched in GO biological process, and the significance is shown in ‘p-value’. ‘m’, ‘k’, and ‘x’ represent the number of genes in the corresponding GO term, the number of genes in the module, and the number of genes belonging to the GO term in the module, respectively. (PDF) [file pcbi.1004042.s025.pdf]

Table S18. GBM modules with enriched GO terms.

Module ID	m	k	x	Gene Ontology	p-value	Genes	p-value
2	234	98	15	Dna Metabolic Process	5.93E-10	TOP2A, RAD51, CDT1, POLQ, EXO1, MSH5, RAD54L, KPNA2, CHEK1, BRCA1, MCM2, HMGB2, CDC6, MCM3, MCM7	3.77E-08
2	118	98	7	Dna Repair	4.81E-05	RAD51, POLQ, EXO1, MSH5, RAD54L, BRCA1, HMGB2	1.17E-03
2	19	98	7	Mitotic Cell Cycle Checkpoint	8.83E-11	TTK, BUB1, KNTC1, BUB1B, ZWINT, CCNA2, MAD2L1	6.07E-09
2	13	98	3	Cytokinesis	1.41E-04	RACGAP1, PRC1, BIRC5	3.32E-03
2	20	98	3	Meiosis I	5.39E-04	RAD51, MSH5, CHEK1	1.03E-02
2	177	98	10	Response To Endogenous Stimulus	1.67E-06	RAD51, POLQ, EXO1, MSH5, RAD54L, CHEK1, CCNA2, BRCA1, HMGB2, MCM7	4.93E-05
2	99	98	26	M Phase	0.00E+00	NDC80, CENPE, KIF2C, TTK, ESPL1, KIF15, KIF11, NCAPH, AURKA, BUB1, RAD51, KNTC1, MSH5, DDX11, RAD54L, BUB1B, KPNA2, PLK1, ZWINT, CHEK1, TPX2, CCNA2, NEK2, CDC25C, MAD2L1, BIRC5	0.00E+00
2	74	98	8	Microtubule Based Process	1.40E-07	KIF2C, TTK, KIF11, KIF23, KIF4A, KPNA2, PRC1, TUBG1	5.48E-06
2	20	98	4	Dna Integrity Checkpoint	1.81E-05	CDT1, CHEK1, CCNA2, CDC6	4.52E-04
2	33	98	10	Regulation Of Mitosis	1.65E-14	TTK, BUB1, KNTC1, BUB1B, ZWINT, CCNA2, NEK2, CDC25C, MAD2L1, BIRC5	1.52E-12
2	31	98	6	Microtubule Cytoskeleton Organization And Biogenesis	1.55E-07	KIF2C, TTK, KIF11, KIF23, PRC1, TUBG1	5.83E-06
2	63	98	7	Interphase	7.26E-07	CENPF, CDC7, DDX11, KPNA2, CDC6, CDC25C, BIRC5	2.22E-05
2	170	98	34	Cell Cycle Process	0.00E+00	NDC80, CENPE, CENPF, KIF2C, TTK, ESPL1, KIF15, KIF11, NCAPH, AURKA, RACGAP1, BUB1, FBXO5, KIF23, RAD51, KNTC1, CDC7, MSH5, DDX11, RAD54L, BUB1B, KPNA2, PLK1, ZWINT, CHEK1, TPX2, CCNA2, PRC1, NEK2, CDC6, CDC25C, TUBG1, MAD2L1, BIRC5	0.00E+00
2	15	98	6	Mitotic Sister Chromatid Segregation	1.17E-09	NDC80, CENPE, ESPL1, NCAPH, DDX11, ZWINT	6.92E-08
2	39	98	4	Regulation Of Dna Metabolic Process	2.73E-04	RAD51, CDT1, KPNA2, CDC6	5.77E-03
2	32	98	5	Meiotic Cell Cycle	5.45E-06	RAD51, MSH5, RAD54L, CHEK1, TUBG1	1.50E-04

2	134	98	28	Mitotic Cell Cycle	0.00E+00	NDC80, CENPE, CENPF, KIF2C, TTK, ESPL1, KIF15, KIF11, NCAPH, AURKA, BUB1, FBXO5, KIF23, KNTC1, CDC7, DDX11, BUB1B, KPNA2, PLK1, ZWINT, TPX2, CCNA2, PRC1, NEK2, CDC6, CDC25C, MAD2L1, BIRC5	0.00E+00
2	134	98	28	Mitotic Cell Cycle	0.00E+00	NDC80, CENPE, CENPF, KIF2C, TTK, ESPL1, KIF15, KIF11, NCAPH, AURKA, BUB1, FBXO5, KIF23, KNTC1, CDC7, DDX11, BUB1B, KPNA2, PLK1, ZWINT, TPX2, CCNA2, PRC1, NEK2, CDC6, CDC25C, MAD2L1, BIRC5	0.00E+00
2	153	98	29	Cell Cycle Phase	0.00E+00	NDC80, CENPE, CENPF, KIF2C, TTK, ESPL1, KIF15, KIF11, NCAPH, AURKA, BUB1, RAD51, KNTC1, CDC7, MSH5, DDX11, RAD54L, BUB1B, KPNA2, PLK1, ZWINT, CHEK1, TPX2, CCNA2, NEK2, CDC6, CDC25C, MAD2L1, BIRC5	0.00E+00
2	43	98	11	Cell Cycle Checkpoint Go 0000075	5.66E-15	TTK, BUB1, KNTC1, CDT1, BUB1B, ZWINT, CHEK1, CCNA2, CDC6, MAD2L1, BIRC5	5.84E-13
2	15	98	3	Dna Replication Initiation	2.22E-04	CDT1, CDC6, MCM3	4.81E-03
2	28	98	9	Chromosome Segregation	6.18E-13	NDC80, CENPE, CENPF, TOP2A, ESPL1, NCAPH, DDX11, ZWINT, BRCA1	5.10E-11
2	15	98	3	Establishment Of Organelle Localization	2.22E-04	CENPE, CENPF, BIRC5	4.81E-03
2	15	98	3	Cell Division	2.22E-04	RACGAP1, PRC1, BIRC5	4.81E-03
2	19	98	4	Regulation Of Mitotic Cell Cycle	1.46E-05	FBXO5, CDC6, CDC25C, BIRC5	3.87E-04
2	93	98	9	Dna Replication	5.90E-08	RAD51, CDT1, EXO1, MSH5, MCM2, HMGB2, CDC6, MCM3, MCM7	2.44E-06
2	31	98	3	Dna Damage Responsesignal Transduction	1.99E-03	CHEK1, CCNA2, BRCA1	3.65E-02
2	44	98	7	Dna Recombination	5.70E-08	RAD51, EXO1, MSH5, RAD54L, KPNA2, CHEK1, BRCA1	2.44E-06
2	8	98	4	Mitotic Spindle Organization And Biogenesis	2.82E-07	TTK, KIF11, KIF23, PRC1	9.68E-06
2	17	98	3	Meiotic Recombination	3.27E-04	RAD51, MSH5, CHEK1	6.75E-03
2	70	98	21	Mitosis	0.00E+00	NDC80, CENPE, KIF2C, TTK, ESPL1, KIF15, KIF11, NCAPH, AURKA, BUB1, KNTC1, DDX11, BUB1B, PLK1, ZWINT, TPX2, CCNA2, NEK2, CDC25C, MAD2L1, BIRC5	0.00E+00

2	276	98	37	Cell Cycle Go 0007049	0.00E+00	NDC80, CENPE, CENPF, KIF2C, TTK, ESPL1, KIF15, KIF11, NCAPH, AURKA, RACGAP1, BUB1, FBXO5, KIF23, RAD51, KNTC1, CDT1, CDC7, MSH5, DDX11, RAD54L, BUB1B, KPNA2, PLK1, ZWINT, CHEK1, TPX2, CCNA2, MCM2, PRC1, NEK2, CDC6, CDC25C, TUBG1, CDC20, MAD2L1, BIRC5	0.00E+00
2	16	98	6	Sister Chromatid Segregation	1.87E-09	NDC80, CENPE, ESPL1, NCAPH, DDX11, ZWINT	1.03E-07
2	19	98	3	Organelle Localization	4.61E-04	CENPE, CENPF, BIRC5	9.05E-03
2	181	98	9	Cytoskeleton Organization And Biogenesis	1.60E-05	KIF2C, TTK, KIF11, RACGAP1, KIF23, KIF4A, KPNA2, PRC1, TUBG1	4.13E-04
2	105	98	8	Chromosome Organization And Biogenesis	2.10E-06	NDC80, CENPE, TOP2A, ESPL1, NCAPH, DDX11, ZWINT, EZH2	5.98E-05
2	51	98	7	Dna Dependent Dna Replication	1.64E-07	RAD51, CDT1, EXO1, MSH5, HMGB2, CDC6, MCM3	5.90E-06
2	57	98	7	Interphase Of Mitotic Cell Cycle	3.61E-07	CENPF, CDC7, DDX11, KPNA2, CDC6, CDC25C, BIRC5	1.16E-05
2	9	98	5	Spindle Organization And Biogenesis	3.95E-09	TTK, KIF11, KIF23, PRC1, TUBG1	2.04E-07
2	150	98	10	Response To Dna Damage Stimulus	3.65E-07	RAD51, POLQ, EXO1, MSH5, RAD54L, CHEK1, CCNA2, BRCA1, HMGB2, MCM7	1.16E-05
2	157	98	15	Regulation Of Cell Cycle	1.96E-12	TTK, BUB1, FBXO5, KNTC1, CDT1, CDC7, BUB1B, ZWINT, CHEK1, CCNA2, NEK2, CDC6, CDC25C, MAD2L1, BIRC5	1.47E-10
2	72	98	21	M Phase Of Mitotic Cell Cycle	0.00E+00	NDC80, CENPE, KIF2C, TTK, ESPL1, KIF15, KIF11, NCAPH, AURKA, BUB1, KNTC1, DDX11, BUB1B, PLK1, ZWINT, TPX2, CCNA2, NEK2, CDC25C, MAD2L1, BIRC5	0.00E+00
3	43	101	5	Cell Cycle Checkpoint Go 0000075	2.81E-05	KNTC1, RAD9A, CHFR, ATM, ATR	1.44E-02
3	74	101	6	Rna Splicing	3.50E-05	PRPF3, SRRM1, PPIG, PRPF4B, TRA2A, SFRS2IP	1.44E-02
3	150	101	7	Response To Dna Damage Stimulus	2.62E-04	LIG1, MSH5, RAD9A, ATM, XRCC2, ATR, BCL6	4.32E-02
4	233	41	9	Defense Response	6.66E-08	CXCL11, NMI, TLR3, CXCL10, APOL3, RSAD2, MX2, HCP5, MX1	5.49E-05
4	63	41	4	Response To Other Organism	5.96E-05	IRF7, IFI44, TLR3, RSAD2	2.46E-02
5	234	56	12	Dna Metabolic Process	5.74E-10	LIG1, CHEK1, NASP, MCM7, FANCC, RAD51, EXO1, XRCC2, CDK2, MCM2, MCM3, TOP2A	5.92E-08
5	118	56	5	Dna Repair	2.13E-04	LIG1, FANCC, RAD51, EXO1, XRCC2	6.05E-03
5	19	56	3	Mitotic Cell Cycle Checkpoint	8.76E-05	KNTC1, TTK, BUB1B	2.72E-03

5	177	56	7	Response To Endogenous Stimulus	1.66E-05	LIG1, CHEK1, MCM7, FANCC, RAD51, EXO1, XRCC2	6.54E-04
5	99	56	14	M Phase	4.66E-15	CENPE, CHEK1, RAD51, XRCC2, KIF2C, NCAPH, TPX2, KIF15, KIF11, NDC80, KNTC1, TTK, PLK1, BUB1B	9.62E-13
5	74	56	6	Microtubule Based Process	1.12E-06	KIF2C, KIF11, PRC1, KIF4A, TTK, KIF23	5.75E-05
5	33	56	3	Regulation Of Mitosis	4.71E-04	KNTC1, TTK, BUB1B	1.21E-02
5	31	56	5	Microtubule Cytoskeleton Organization And Biogenesis	2.81E-07	KIF2C, KIF11, PRC1, TTK, KIF23	1.65E-05
5	170	56	20	Cell Cycle Process	4.97E-14	CENPE, CHEK1, TIMELESS, RAD51, XRCC2, KIF2C, NCAPH, TPX2, CENPF, KIF15, CDK2, KIF11, NDC80, PRC1, KNTC1, TTK, PLK1, FBXO5, BUB1B, KIF23	5.86E-12
5	15	56	3	Mitotic Sister Chromatid Segregation	4.17E-05	CENPE, NCAPH, NDC80	1.56E-03
5	32	56	3	Meiotic Cell Cycle	4.29E-04	CHEK1, RAD51, XRCC2	1.14E-02
5	134	56	16	Mitotic Cell Cycle	0.00E+00	CENPE, KIF2C, NCAPH, TPX2, CENPF, KIF15, CDK2, KIF11, NDC80, PRC1, KNTC1, TTK, PLK1, FBXO5, BUB1B, KIF23	0.00E+00
5	153	56	17	Cell Cycle Phase	1.07E-14	CENPE, CHEK1, TIMELESS, RAD51, XRCC2, KIF2C, NCAPH, TPX2, CENPF, KIF15, CDK2, KIF11, NDC80, KNTC1, TTK, PLK1, BUB1B	1.47E-12
5	43	56	4	Cell Cycle Checkpoint Go 0000075	4.52E-05	CHEK1, KNTC1, TTK, BUB1B	1.62E-03
5	28	56	5	Chromosome Segregation	1.64E-07	CENPE, NCAPH, CENPF, NDC80, TOP2A	1.13E-05
5	93	56	7	Dna Replication	2.22E-07	NASP, MCM7, RAD51, EXO1, CDK2, MCM2, MCM3	1.41E-05
5	44	56	4	Dna Recombination	4.96E-05	LIG1, CHEK1, RAD51, EXO1	1.69E-03
5	8	56	4	Mitotic Spindle Organization And Biogenesis	2.90E-08	KIF11, PRC1, TTK, KIF23	2.39E-06
5	70	56	11	Mitosis	0.00E+00	CENPE, KIF2C, NCAPH, TPX2, KIF15, KIF11, NDC80, KNTC1, TTK, PLK1, BUB1B	0.00E+00
5	276	56	22	Cell Cycle Go 0007049	0.00E+00	CENPE, CHEK1, NASP, TIMELESS, RAD51, XRCC2, KIF2C, NCAPH, TPX2, CENPF, KIF15, CDK2, MCM2, KIF11, NDC80, PRC1, KNTC1, TTK, PLK1, FBXO5, BUB1B, KIF23	0.00E+00
5	16	56	3	Sister Chromatid Segregation	5.11E-05	CENPE, NCAPH, NDC80	1.69E-03
5	181	56	6	Cytoskeleton Organization And Biogenesis	1.85E-04	KIF2C, KIF11, PRC1, KIF4A, TTK, KIF23	5.44E-03
5	105	56	7	Chromosome Organization And Biogenesis	5.11E-07	CENPE, NASP, CHAF1A, NCAPH, NDC80, TOP2A, EZH2	2.81E-05
5	51	56	4	Dna Dependent Dna Replication	8.91E-05	RAD51, EXO1, CDK2, MCM3	2.72E-03

5	27	56	3	Dna Packaging	2.57E-04	CHAF1A, NCAPH, TOP2A	7.08E-03
5	9	56	4	Spindle Organization And Biogenesis	5.19E-08	KIF11, PRC1, TTK, KIF23	3.90E-06
5	150	56	7	Response To Dna Damage Stimulus	5.63E-06	LIG1, CHEK1, MCM7, FANCC, RAD51, EXO1, XRCC2	2.58E-04
5	157	56	7	Regulation Of Cell Cycle	7.60E-06	CHEK1, TIMELESS, CDK2, KNTC1, TTK, FBXO5, BUB1B	3.14E-04
5	72	56	11	M Phase Of Mitotic Cell Cycle	8.10E-15	CENPE, KIF2C, NCAPH, TPX2, KIF15, KIF11, NDC80, KNTC1, TTK, PLK1, BUB1B	1.34E-12
6	146	23	4	Synaptic Transmission	1.53E-04	GRM3, MBP, NPY, PLP1	4.22E-02
6	16	23	3	Regulation Of Action Potential	3.35E-06	MAL, MBP, PLP1	2.77E-03
6	159	23	5	Transmission Of Nerve Impulse	1.05E-05	GRM3, MAL, MBP, NPY, PLP1	4.32E-03
7	99	43	7	M Phase	5.18E-08	BIRC5, MAD2L1, AURKA, BUB1B, RAD54B, NCAPH, NDC80	1.16E-05
7	33	43	3	Regulation Of Mitosis	2.15E-04	BIRC5, MAD2L1, BUB1B	2.21E-02
7	170	43	9	Cell Cycle Process	6.73E-09	BIRC5, MAD2L1, DBF4, AURKA, BUB1B, RACGAP1, RAD54B, NCAPH, NDC80	2.78E-06
7	134	43	7	Mitotic Cell Cycle	4.17E-07	BIRC5, MAD2L1, DBF4, AURKA, BUB1B, NCAPH, NDC80	4.91E-05
7	153	43	8	Cell Cycle Phase	5.62E-08	BIRC5, MAD2L1, DBF4, AURKA, BUB1B, RAD54B, NCAPH, NDC80	1.16E-05
7	43	43	3	Cell Cycle Checkpoint Go 0000075	4.74E-04	BIRC5, MAD2L1, BUB1B	4.34E-02
7	70	43	6	Mitosis	1.60E-07	BIRC5, MAD2L1, AURKA, BUB1B, NCAPH, NDC80	2.60E-05
7	276	43	11	Cell Cycle Go 0007049	2.26E-09	DTYMK, BIRC5, MAD2L1, DBF4, AURKA, GMNN, BUB1B, RACGAP1, RAD54B, NCAPH, NDC80	1.86E-06
7	72	43	6	M Phase Of Mitotic Cell Cycle	1.89E-07	BIRC5, MAD2L1, AURKA, BUB1B, NCAPH, NDC80	2.60E-05
8	233	73	12	Defense Response	1.35E-08	PTX3, IL32, CCL20, STAB1, TCIRG1, HP, CCR5, NCF1, WAS, CEBPB, CCR1, PTPRC	2.78E-06
8	128	73	7	Behavior	1.18E-05	CXCL5, CCL20, RNASE2, CCR5, PLAUR, SYK, CCL2	1.22E-03
8	80	73	7	Locomotory Behavior	5.01E-07	CXCL5, CCL20, RNASE2, CCR5, PLAUR, SYK, CCL2	6.89E-05
8	113	73	5	Inflammatory Response	6.02E-04	PTX3, CCL20, CCR5, CEBPB, CCR1	2.61E-02
8	88	73	6	Cellular Cation Homeostasis	1.47E-05	STC1, CP, CCR5, CCR1, CCL2, PTPRC	1.35E-03
8	261	73	11	Response To Chemical Stimulus	4.36E-07	SOD2, CXCL5, STC1, CCL20, RNASE2, HSPA6, CCR5, PLAUR, SYK, CCL2, LYN	6.89E-05
8	207	73	12	Immune Response	3.55E-09	TREM1, IL32, IL1R2, CCL20, CCR5, IL4R, FCGR2B, WAS, CEBPB, CCR1, CCL2, PTPRC	1.46E-06
8	172	73	8	Homeostatic Process	8.98E-06	STC1, CP, CCR5, SPI1, CCR1, CCL2, LYN, PTPRC	1.06E-03

8	115	73	6	Cellular Homeostasis	6.73E-05	STC1, CP, CCR5, CCR1, CCL2, PTPRC	4.02E-03
8	290	73	15	Immune System Process	1.54E-10	TREM1, IL32, IL1R2, CCL20, CCR5, IL4R, FCGR2B, WAS, CEBPB, SPI1, SYK, CCR1, CCL2, LYN, PTPRC	1.27E-07
8	269	73	13	Response To External Stimulus	6.74E-09	PTX3, CXCL5, STC1, CCL20, RNASE2, THBD, CCR5, PLAUR, WAS, CEBPB, SYK, CCR1, CCL2	1.85E-06
8	130	73	6	Chemical Homeostasis	1.33E-04	STC1, CP, CCR5, CCR1, CCL2, PTPRC	7.30E-03
8	107	73	6	Ion Homeostasis	4.49E-05	STC1, CP, CCR5, CCR1, CCL2, PTPRC	3.17E-03
8	91	73	6	Cation Homeostasis	1.79E-05	STC1, CP, CCR5, CCR1, CCL2, PTPRC	1.47E-03
8	168	73	7	Response To Wounding	6.82E-05	PTX3, CCL20, THBD, CCR5, WAS, CEBPB, CCR1	4.02E-03
10	234	73	10	Dna Metabolic Process	1.35E-06	CHEK1, ORC2L, CDK2, CDT1, POLQ, MSH6, FEN1, MCM3, FANCG, MCM2	8.55E-05
10	19	73	4	Mitotic Cell Cycle Checkpoint	4.50E-06	KNTC1, BUB1B, ZWINT, CCNA2	2.32E-04
10	177	73	7	Response To Endogenous Stimulus	9.48E-05	CHEK1, POLQ, MSH6, FEN1, FANCG, CCNA2, LYN	3.40E-03
10	99	73	10	M Phase	3.59E-10	CHEK1, PLK1, KNTC1, BUB1B, NDC80, TPX2, ZWINT, KIF2C, CCNA2, AURKA	4.30E-08
10	20	73	3	Dna Integrity Checkpoint	2.26E-04	CHEK1, CDT1, CCNA2	7.78E-03
10	33	73	4	Regulation Of Mitosis	4.45E-05	KNTC1, BUB1B, ZWINT, CCNA2	1.93E-03
10	170	73	13	Cell Cycle Process	2.30E-11	CHEK1, CDK2, PLK1, KNTC1, BUB1B, SKP2, NDC80, TPX2, ZWINT, KIF2C, CCNA2, AURKA, CDKN3	4.75E-09
10	134	73	12	Mitotic Cell Cycle	2.25E-11	CDK2, PLK1, KNTC1, BUB1B, SKP2, NDC80, TPX2, ZWINT, KIF2C, CCNA2, AURKA, CDKN3	4.75E-09
10	153	73	13	Cell Cycle Phase	5.93E-12	CHEK1, CDK2, PLK1, KNTC1, BUB1B, SKP2, NDC80, TPX2, ZWINT, KIF2C, CCNA2, AURKA, CDKN3	2.52E-09
10	43	73	7	Cell Cycle Checkpoint Go 0000075	6.07E-09	CHEK1, KNTC1, CDT1, BUB1B, ZWINT, FANCG, CCNA2	5.01E-07
10	15	73	3	Dna Replication Initiation	9.23E-05	ORC2L, CDT1, MCM3	3.40E-03
10	28	73	3	Chromosome Segregation	6.28E-04	NDC80, SRPK1, ZWINT	1.92E-02
10	138	73	8	Rna Processing	1.74E-06	NONO, NCBP1, SFRS1, NUDT21, CSTF2, SRPK1, SFRS2, CPSF6	1.03E-04
10	61	73	8	Mrna Processing Go 0006397	2.80E-09	NONO, NCBP1, SFRS1, NUDT21, CSTF2, SRPK1, SFRS2, CPSF6	2.57E-07
10	93	73	6	Dna Replication	2.02E-05	ORC2L, CDK2, CDT1, MSH6, MCM3, MCM2	9.28E-04

10	70	73	9	Mitosis	3.22E-10	PLK1, KNTC1, BUB1B, NDC80, TPX2, ZWINT, KIF2C, CCNA2, AURKA	4.30E-08
10	276	73	16	Cell Cycle Go 0007049	6.10E-12	CHEK1, CDK2, PLK1, KNTC1, CDT1, BUB1B, SKP2, NDC80, TPX2, ZWINT, FANCG, KIF2C, CCNA2, AURKA, CDKN3, MCM2	2.52E-09
10	71	73	9	Mrna Metabolic Process	3.67E-10	NONO, NCBP1, SFRS1, NUDT21, CSTF2, UPF2, SRPK1, SFRS2, CPSF6	4.30E-08
10	74	73	5	Rna Splicing	8.29E-05	NONO, NCBP1, SFRS1, SRPK1, SFRS2	3.26E-03
10	51	73	5	Dna Dependent Dna Replication	1.35E-05	ORC2L, CDK2, CDT1, MSH6, MCM3	6.54E-04
10	150	73	6	Response To Dna Damage Stimulus	2.89E-04	CHEK1, POLQ, MSH6, FEN1, FANCG, CCNA2	9.55E-03
10	157	73	9	Regulation Of Cell Cycle	4.12E-07	CHEK1, CDK2, KNTC1, CDT1, BUB1B, ZWINT, FANCG, CCNA2, CDKN3	2.83E-05
10	72	73	9	M Phase Of Mitotic Cell Cycle	4.17E-10	PLK1, KNTC1, BUB1B, NDC80, TPX2, ZWINT, KIF2C, CCNA2, AURKA	4.30E-08
11	95	89	6	Positive Regulation Of Signal Transduction	7.05E-05	TNFRSF10B, CFLAR, TNFRSF1A, TICAM1, MYD88, FLNA	6.47E-03
11	128	89	6	Enzyme Linked Receptor Protein Signaling Pathway	3.62E-04	SHC1, PXN, PTK2B, FGFR4, ERBB2, CBLC	2.30E-02
11	87	89	6	I Kappab Kinase Nf Kappab Cascade	4.30E-05	TNFRSF10B, CFLAR, TNFRSF1A, TICAM1, MYD88, FLNA	5.06E-03
11	170	89	9	Regulation Of Signal Transduction	4.35E-06	RGS4, SHC1, TNFRSF10B, CFLAR, TNFRSF1A, TICAM1, MYD88, CBLC, FLNA	1.87E-03
11	66	89	6	Positive Regulation Of I Kappab Kinase Nf Kappab Cascade	8.75E-06	TNFRSF10B, CFLAR, TNFRSF1A, TICAM1, MYD88, FLNA	1.87E-03
11	236	89	10	Protein Kinase Cascade	9.06E-06	RGS4, SHC1, TNFRSF10B, NMI, CFLAR, FGF2, TNFRSF1A, TICAM1, MYD88, FLNA	1.87E-03
11	57	89	4	Regulation Of Map Kinase Activity	8.17E-04	RGS4, SHC1, FGF2, ERBB2	4.81E-02
11	269	89	9	Response To External Stimulus	1.62E-04	ITGA2, NMI, F11R, AHSG, FGF2, TNFRSF1A, MAPK1, PTX3, CDKN1A	1.21E-02
11	77	89	6	Transmembrane Receptor Protein Tyrosine Kinase Signaling Pathway	2.14E-05	SHC1, PXN, PTK2B, FGFR4, ERBB2, CBLC	2.94E-03
11	71	89	6	Regulation Of I Kappab Kinase Nf Kappab Cascade	1.34E-05	TNFRSF10B, CFLAR, TNFRSF1A, TICAM1, MYD88, FLNA	2.21E-03
12	81	47	6	Cell Migration	6.62E-07	LAMC1, ENPEP, LAMB1, ITGB1, MYH9, CD34	1.82E-04
13	20	91	3	Meiosis I	4.34E-04	LIG3, RAD1, RAD51	4.47E-02
13	99	91	9	M Phase	5.33E-08	LIG3, PIN1, RAD1, NDC80, NEK2, RAD51, KIF11, BUB1, NCAPH	2.20E-05

13	170	91	12	Cell Cycle Process	5.10E-09	LIG3, PIN1, FBXO5, RAD1, RACGAP1, NDC80, NEK2, RAD51, KIF23, KIF11, BUB1, NCAPH	4.21E-06
13	134	91	8	Mitotic Cell Cycle	7.46E-06	PIN1, FBXO5, NDC80, NEK2, KIF23, KIF11, BUB1, NCAPH	1.23E-03
13	153	91	9	Cell Cycle Phase	2.20E-06	LIG3, PIN1, RAD1, NDC80, NEK2, RAD51, KIF11, BUB1, NCAPH	4.54E-04
13	70	91	6	Mitosis	1.40E-05	PIN1, NDC80, NEK2, KIF11, BUB1, NCAPH	1.93E-03
13	276	91	13	Cell Cycle Go 0007049	1.41E-07	LIG3, PIN1, FBXO5, RAD1, RACGAP1, GPS1, NDC80, NEK2, RAD51, KIF23, KIF11, BUB1, NCAPH	3.88E-05
13	72	91	6	M Phase Of Mitotic Cell Cycle	1.65E-05	PIN1, NDC80, NEK2, KIF11, BUB1, NCAPH	1.95E-03
14	124	65	6	Positive Regulation Of Cell Proliferation	5.31E-05	VEGFA, NRP1, LAMC1, TIMP1, LAMB1, ANG	3.02E-02
14	81	65	5	Cell Migration	7.32E-05	NRP1, LAMC1, NRP2, LAMB1, ANG	3.02E-02
16	234	93	15	Dna Metabolic Process	2.77E-10	CDC6, CHEK1, RAD51, CDT1, POLQ, EXO1, RAD54L, BRCA2, LIG3, BRCA1, ORC1L, POLG2, POLD1, CDK2, FEN1	2.28E-08
16	118	93	9	Dna Repair	2.96E-07	RAD51, POLQ, EXO1, RAD54L, BRCA2, LIG3, BRCA1, POLD1, FEN1	1.22E-05
16	19	93	5	Mitotic Cell Cycle Checkpoint	2.63E-07	CCNA2, BUB1, BUB1B, TTK, KNTC1	1.14E-05
16	20	93	3	Meiosis I	4.62E-04	CHEK1, RAD51, LIG3	8.87E-03
16	18	93	3	Regulation Of Dna Replication	3.35E-04	CDC6, CDT1, CDK2	6.73E-03
16	177	93	12	Response To Endogenous Stimulus	1.04E-08	CCNA2, CHEK1, CHEK2, RAD51, POLQ, EXO1, RAD54L, BRCA2, LIG3, BRCA1, POLD1, FEN1	6.11E-07
16	99	93	22	M Phase	0.00E+00	CCNA2, KIF2C, ESPL1, NEK2, CHEK1, NCAPH, AURKA, BUB1, PLK1, RAD51, BUB1B, DDX11, RAD54L, LIG3, CDC25C, NDC80, KIF11, KIF15, TTK, KNTC1, TPX2, CENPE	0.00E+00
16	74	93	4	Microtubule Based Process	2.54E-03	KIF2C, KIF23, KIF11, TTK	4.28E-02
16	20	93	5	Dna Integrity Checkpoint	3.49E-07	CCNA2, CDC6, CHEK1, CHEK2, CDT1	1.37E-05
16	33	93	7	Regulation Of Mitosis	4.73E-09	CCNA2, NEK2, BUB1, BUB1B, CDC25C, TTK, KNTC1	3.25E-07
16	31	93	4	Microtubule Cytoskeleton Organization And Biogenesis	8.94E-05	KIF2C, KIF23, KIF11, TTK	2.38E-03
16	63	93	6	Interphase	8.60E-06	CDC6, DDX11, SKP2, CDC25C, POLD1, CDK2	2.84E-04

16	170	93	28	Cell Cycle Process	1.71E-14	CCNA2, KIF2C, ESPL1, NEK2, CDC6, CHEK1, NCAPH, AURKA, BUB1, KIF23, PLK1, RAD51, BUB1B, DDX11, RAD54L, BRCA2, LIG3, SKP2, CDC25C, POLD1, NDC80, KIF11, KIF15, TTK, KNTC1, CDK2, TPX2, CENPE	3.57E-12
16	15	93	5	Mitotic Sister Chromatid Segregation	6.96E-08	ESPL1, NCAPH, DDX11, NDC80, CENPE	3.59E-06
16	39	93	4	Regulation Of Dna Metabolic Process	2.23E-04	CDC6, RAD51, CDT1, CDK2	4.84E-03
16	32	93	4	Meiotic Cell Cycle	1.02E-04	CHEK1, RAD51, RAD54L, LIG3	2.62E-03
16	134	93	23	Mitotic Cell Cycle	4.22E-14	CCNA2, KIF2C, ESPL1, NEK2, CDC6, NCAPH, AURKA, BUB1, KIF23, PLK1, BUB1B, DDX11, SKP2, CDC25C, POLD1, NDC80, KIF11, KIF15, TTK, KNTC1, CDK2, TPX2, CENPE	6.96E-12
16	153	93	26	Cell Cycle Phase	1.73E-14	CCNA2, KIF2C, ESPL1, NEK2, CDC6, CHEK1, NCAPH, AURKA, BUB1, PLK1, RAD51, BUB1B, DDX11, RAD54L, LIG3, SKP2, CDC25C, POLD1, NDC80, KIF11, KIF15, TTK, KNTC1, CDK2, TPX2, CENPE	3.57E-12
16	43	93	9	Cell Cycle Checkpoint Go 0000075	3.00E-11	CCNA2, CDC6, CHEK1, BUB1, CHEK2, CDT1, BUB1B, TTK, KNTC1	3.10E-09
16	15	93	3	Dna Replication Initiation	1.90E-04	CDC6, CDT1, ORC1L	4.35E-03
16	28	93	6	Chromosome Segregation	5.92E-08	ESPL1, NCAPH, DDX11, BRCA1, NDC80, CENPE	3.26E-06
16	13	93	3	G1 Phase Of Mitotic Cell Cycle	1.21E-04	CDC6, CDC25C, CDK2	3.01E-03
16	19	93	3	Regulation Of Mitotic Cell Cycle	3.95E-04	CDC6, CDC25C, CDK2	7.76E-03
16	93	93	8	Dna Replication	5.55E-07	CDC6, RAD51, CDT1, EXO1, ORC1L, POLG2, POLD1, CDK2	2.08E-05
16	31	93	4	Dna Damage Responsesignal Transduction	8.94E-05	CCNA2, CHEK1, CHEK2, BRCA1	2.38E-03
16	17	93	3	Dna Damage Checkpoint	2.80E-04	CCNA2, CHEK1, CHEK2	5.78E-03
16	39	93	4	Regulation Of Cyclin Dependent Protein Kinase Activity	2.23E-04	CDC6, CHEK1, CDC25C, CDC25A	4.84E-03
16	21	93	3	Double Strand Break Repair	5.36E-04	RAD51, BRCA1, FEN1	1.01E-02
16	44	93	6	Dna Recombination	1.00E-06	CHEK1, RAD51, EXO1, RAD54L, LIG3, BRCA1	3.60E-05
16	15	93	3	G1 Phase	1.90E-04	CDC6, CDC25C, CDK2	4.35E-03
16	8	93	3	Mitotic Spindle Organization And Biogenesis	2.43E-05	KIF23, KIF11, TTK	7.71E-04
16	17	93	3	Meiotic Recombination	2.80E-04	CHEK1, RAD51, LIG3	5.78E-03

16	70	93	18	Mitosis	1.13E-14	CCNA2, KIF2C, ESPL1, NEK2, NCAPH, AURKA, BUB1, PLK1, BUB1B, DDX11, CDC25C, NDC80, KIF11, KIF15, TTK, KNTC1, TPX2, CENPE	3.57E-12
16	276	93	32	Cell Cycle Go 0007049	9.88E-14	CCNA2, KIF2C, ESPL1, NEK2, CDC6, CHEK1, NCAPH, AURKA, BUB1, KIF23, CHEK2, PLK1, RAD51, CDT1, BUB1B, DDX11, RAD54L, BRCA2, LIG3, SKP2, CDC25C, POLD1, NDC80, KIF11, KIF15, TTK, KNTC1, CDK2, TPX2, CDC20, CDC25A, CENPE	1.16E-11
16	16	93	5	Sister Chromatid Segregation	1.01E-07	ESPL1, NCAPH, DDX11, NDC80, CENPE	4.89E-06
16	105	93	5	Chromosome Organization And Biogenesis	1.30E-03	ESPL1, NCAPH, DDX11, NDC80, CENPE	2.32E-02
16	51	93	7	Dna Dependent Dna Replication	1.14E-07	CDC6, RAD51, CDT1, EXO1, ORC1L, POLG2, CDK2	5.25E-06
16	57	93	6	Interphase Of Mitotic Cell Cycle	4.77E-06	CDC6, DDX11, SKP2, CDC25C, POLD1, CDK2	1.64E-04
16	9	93	3	Spindle Organization And Biogenesis	3.62E-05	KIF23, KIF11, TTK	1.11E-03
16	150	93	12	Response To Dna Damage Stimulus	1.57E-09	CCNA2, CHEK1, CHEK2, RAD51, POLQ, EXO1, RAD54L, BRCA2, LIG3, BRCA1, POLD1, FEN1	1.18E-07
16	157	93	13	Regulation Of Cell Cycle	2.02E-10	CCNA2, NEK2, CDC6, CHEK1, BUB1, CHEK2, CDT1, BUB1B, CDC25C, TTK, KNTC1, CDK2, CDC25A	1.85E-08
16	72	93	18	M Phase Of Mitotic Cell Cycle	6.15E-14	CCNA2, KIF2C, ESPL1, NEK2, NCAPH, AURKA, BUB1, PLK1, BUB1B, DDX11, CDC25C, NDC80, KIF11, KIF15, TTK, KNTC1, TPX2, CENPE	8.46E-12
19	146	48	6	Synaptic Transmission	2.31E-05	SYN1, GAD2, NPY, SYN2, SYT1, RAB3A	6.36E-03
19	23	48	4	Generation Of A Signal Involved In Cell Cell Signaling	1.86E-06	SYT1, RAB3A, SNAP25, STX1A	1.54E-03
19	159	48	6	Transmission Of Nerve Impulse	3.74E-05	SYN1, GAD2, NPY, SYN2, SYT1, RAB3A	7.72E-03
20	146	74	11	Synaptic Transmission	1.24E-09	SYT1, SYN1, CPNE6, DOC2A, SYN2, HTR2A, RAB3A, NPY, BSN, HTR1E, GAD2	1.02E-06
20	23	74	3	Generation Of A Signal Involved In Cell Cell Signaling	3.61E-04	SNAP25, SYT1, RAB3A	4.97E-02
20	159	74	11	Transmission Of Nerve Impulse	3.07E-09	SYT1, SYN1, CPNE6, DOC2A, SYN2, HTR2A, RAB3A, NPY, BSN, HTR1E, GAD2	1.27E-06
21	236	70	8	Protein Kinase Cascade	6.40E-05	ADAM9, FLNA, LGALS1, CXCR4, C5AR1, NMI, SHC1, TNFRSF10B	2.64E-02

21	269	70	9	Response To External Stimulus	2.39E-05	PLAU, PTX3, IL1RAP, SERPINE1, PLAUR, CXCR4, CDKN1A, C5AR1, NMI	1.97E-02
25	26	75	3	Negative Regulation Of Multicellular Organismal Process	5.44E-04	TGFB2, SERPINE1, SRGN	4.48E-02
25	26	75	3	Jak Stat Cascade	5.44E-04	STAT3, SOCS3, HCLS1	4.48E-02
25	207	75	7	Immune Response	2.96E-04	TREM1, LTF, TGFB2, C5AR1, LCP2, FCGR2B, FTH1	4.48E-02
25	290	75	8	Immune System Process	4.28E-04	TREM1, LTF, TGFB2, C5AR1, LCP2, FCGR2B, FTH1, HCLS1	4.48E-02
26	18	119	4	Axon Guidance	2.49E-05	CDK5R1, SLIT1, NRXN1, SIAH1	5.14E-03
26	41	119	5	Neurite Development	4.88E-05	MAPT, CDK5R1, SLIT1, NRXN1, SIAH1	5.76E-03
26	74	119	6	Microtubule Based Process	8.79E-05	MAPT, KIF1B, CLASP2, NLGN1, APC, ARHGEF10L	8.69E-03
26	33	119	5	Axonogenesis	1.65E-05	MAPT, CDK5R1, SLIT1, NRXN1, SIAH1	5.14E-03
26	38	119	5	Cellular Morphogenesis During Differentiation	3.35E-05	MAPT, CDK5R1, SLIT1, NRXN1, SIAH1	5.53E-03
26	65	119	6	Generation Of Neurons	4.21E-05	MAPT, CDK5R1, NLGN1, SLIT1, NRXN1, SIAH1	5.76E-03
26	103	119	6	Regulation Of Cellular Component Organization And Biogenesis	5.38E-04	MAPT, CLASP2, APC, ARHGEF10L, TERF2, EIF4G3	3.17E-02
26	35	119	5	Regulation Of Organelle Organization And Biogenesis	2.22E-05	MAPT, CLASP2, APC, ARHGEF10L, TERF2	5.14E-03
26	70	119	5	Cell Cell Adhesion	6.28E-04	CYFIP2, CDK5R1, NLGN1, CLDN4, COL13A1	3.24E-02
26	75	119	6	Neurogenesis	9.48E-05	MAPT, CDK5R1, NLGN1, SLIT1, NRXN1, SIAH1	8.69E-03
26	26	119	4	Regulation Of Cytoskeleton Organization And Biogenesis	1.15E-04	MAPT, CLASP2, APC, ARHGEF10L	8.75E-03
26	58	119	6	Neuron Differentiation	2.18E-05	MAPT, CDK5R1, NLGN1, SLIT1, NRXN1, SIAH1	5.14E-03
26	49	119	5	Neuron Development	1.17E-04	MAPT, CDK5R1, SLIT1, NRXN1, SIAH1	8.75E-03
26	11	119	3	Microtubule Polymerization Or Depolymerization	1.47E-04	MAPT, CLASP2, APC	1.01E-02
27	19	53	4	Mitotic Cell Cycle Checkpoint	1.23E-06	CCNA2, TTK, MAD2L1, BUB1B	4.63E-05
27	13	53	4	Cytokinesis	2.32E-07	BIRC5, NUSAP1, PRC1, RACGAP1	1.37E-05
27	99	53	15	M Phase	0.00E+00	BIRC5, NDC80, NUSAP1, CENPE, CCNA2, KIF2C, TTK, KIF11, TPX2, AURKA, UBE2C, RAD51, MAD2L1, BUB1B, CDC25C	0.00E+00
27	74	53	7	Microtubule Based Process	3.05E-08	NUSAP1, PRC1, KIF2C, TTK, KIF11, KIF4A, KIF23	2.29E-06
27	33	53	8	Regulation Of Mitosis	9.43E-13	BIRC5, NUSAP1, CCNA2, TTK, UBE2C, MAD2L1, BUB1B, CDC25C	9.73E-11
27	31	53	6	Microtubule Cytoskeleton Organization And Biogenesis	3.67E-09	NUSAP1, PRC1, KIF2C, TTK, KIF11, KIF23	3.37E-07

27	170	53	19	Cell Cycle Process	0.00E+00	BIRC5, NDC80, NUSAP1, CENPE, CENPF, CCNA2, PRC1, KIF2C, TTK, KIF11, TPX2, AURKA, RACGAP1, UBE2C, RAD51, MAD2L1, BUB1B, CDC25C, KIF23	0.00E+00
27	15	53	3	Mitotic Sister Chromatid Segregation	3.53E-05	NDC80, NUSAP1, CENPE	1.27E-03
27	134	53	17	Mitotic Cell Cycle	0.00E+00	BIRC5, NDC80, NUSAP1, CENPE, CENPF, CCNA2, PRC1, KIF2C, TTK, KIF11, TPX2, AURKA, UBE2C, MAD2L1, BUB1B, CDC25C, KIF23	0.00E+00
27	153	53	16	Cell Cycle Phase	0.00E+00	BIRC5, NDC80, NUSAP1, CENPE, CENPF, CCNA2, KIF2C, TTK, KIF11, TPX2, AURKA, UBE2C, RAD51, MAD2L1, BUB1B, CDC25C	0.00E+00
27	43	53	5	Cell Cycle Checkpoint Go 0000075	1.15E-06	BIRC5, CCNA2, TTK, MAD2L1, BUB1B	4.63E-05
27	28	53	5	Chromosome Segregation	1.24E-07	NDC80, NUSAP1, CENPE, CENPF, TOP2A	7.86E-06
27	15	53	4	Establishment Of Organelle Localization	4.40E-07	BIRC5, NUSAP1, CENPE, CENPF	2.14E-05
27	15	53	4	Cell Division	4.40E-07	BIRC5, NUSAP1, PRC1, RACGAP1	2.14E-05
27	8	53	4	Mitotic Spindle Organization And Biogenesis	2.31E-08	PRC1, TTK, KIF11, KIF23	1.91E-06
27	70	53	14	Mitosis	0.00E+00	BIRC5, NDC80, NUSAP1, CENPE, CCNA2, KIF2C, TTK, KIF11, TPX2, AURKA, UBE2C, MAD2L1, BUB1B, CDC25C	0.00E+00
27	276	53	21	Cell Cycle Go 0007049	0.00E+00	BIRC5, NDC80, NUSAP1, CENPE, CENPF, CCNA2, PRC1, KIF2C, TTK, KIF11, TPX2, AURKA, RACGAP1, UBE2C, CDC20, MCM2, RAD51, MAD2L1, BUB1B, CDC25C, KIF23	0.00E+00
27	16	53	3	Sister Chromatid Segregation	4.33E-05	NDC80, NUSAP1, CENPE	1.49E-03
27	19	53	4	Organelle Localization	1.23E-06	BIRC5, NUSAP1, CENPE, CENPF	4.63E-05
27	181	53	8	Cytoskeleton Organization And Biogenesis	1.11E-06	NUSAP1, PRC1, KIF2C, TTK, KIF11, RACGAP1, KIF4A, KIF23	4.63E-05
27	105	53	4	Chromosome Organization And Biogenesis	1.15E-03	NDC80, NUSAP1, CENPE, TOP2A	3.65E-02
27	9	53	4	Spindle Organization And Biogenesis	4.15E-08	PRC1, TTK, KIF11, KIF23	2.85E-06
27	157	53	8	Regulation Of Cell Cycle	3.76E-07	BIRC5, NUSAP1, CCNA2, TTK, UBE2C, MAD2L1, BUB1B, CDC25C	2.07E-05
27	72	53	14	M Phase Of Mitotic Cell Cycle	0.00E+00	BIRC5, NDC80, NUSAP1, CENPE, CCNA2, KIF2C, TTK, KIF11, TPX2, AURKA, UBE2C, MAD2L1, BUB1B, CDC25C	0.00E+00
28	128	55	6	Behavior	2.43E-05	ITGB2, PLAUR, RNASE2, CXCR4, SYK, C5AR1	6.67E-03

28	80	55	6	Locomotory Behavior	1.59E-06	ITGB2, PLAUR, RNASE2, CXCR4, SYK, C5AR1	6.56E-04
28	261	55	7	Response To Chemical Stimulus	1.73E-04	ITGB2, GNAI2, PLAUR, RNASE2, CXCR4, SYK, C5AR1	2.86E-02
28	290	55	8	Immune System Process	4.68E-05	ITGB2, TGFB1, FCGR2B, CXCR4, CTSS, CD4, SYK, C5AR1	9.65E-03
28	269	55	10	Response To External Stimulus	3.16E-07	ITGB2, TGFB1, GNAI2, F13A1, PLAUR, RNASE2, CXCR4, CYBB, SYK, C5AR1	2.60E-04
29	99	30	6	M Phase	1.34E-07	KIF15, BUB1B, KIF2C, NEK2, BIRC5, TTK	1.38E-05
29	33	30	4	Regulation Of Mitosis	1.22E-06	BUB1B, NEK2, BIRC5, TTK	1.00E-04
29	63	30	4	Interphase	1.68E-05	CDC7, CDKN2C, BIRC5, CDKN3	1.16E-03
29	170	30	9	Cell Cycle Process	2.00E-10	CDC7, CDKN2C, KIF15, BUB1B, KIF2C, NEK2, BIRC5, CDKN3, TTK	5.49E-08
29	134	30	9	Mitotic Cell Cycle	2.35E-11	CDC7, CDKN2C, KIF15, BUB1B, KIF2C, NEK2, BIRC5, CDKN3, TTK	1.93E-08
29	153	30	9	Cell Cycle Phase	7.76E-11	CDC7, CDKN2C, KIF15, BUB1B, KIF2C, NEK2, BIRC5, CDKN3, TTK	3.20E-08
29	43	30	3	Cell Cycle Checkpoint Go 0000075	1.61E-04	BUB1B, BIRC5, TTK	9.49E-03
29	70	30	6	Mitosis	1.65E-08	KIF15, BUB1B, KIF2C, NEK2, BIRC5, TTK	2.69E-06
29	276	30	10	Cell Cycle Go 0007049	6.82E-10	CDC7, CDKN2C, KIF15, BUB1B, KIF2C, NEK2, BIRC5, CDKN3, TTK, MCM2	1.41E-07
29	57	30	4	Interphase Of Mitotic Cell Cycle	1.13E-05	CDC7, CDKN2C, BIRC5, CDKN3	8.46E-04
29	157	30	7	Regulation Of Cell Cycle	8.86E-08	CDC7, CDKN2C, BUB1B, NEK2, BIRC5, CDKN3, TTK	1.04E-05
29	72	30	6	M Phase Of Mitotic Cell Cycle	1.96E-08	KIF15, BUB1B, KIF2C, NEK2, BIRC5, TTK	2.69E-06
29	23	30	3	G1 S Transition Of Mitotic Cell Cycle	2.39E-05	CDC7, CDKN2C, CDKN3	1.52E-03
32	46	71	4	Nuclear Import	1.50E-04	TPR, KPNB1, KPNA6, RANBP2	2.18E-02
32	249	71	7	Macromolecular Complex Assembly	6.40E-04	SMARCA5, EIF3B, MED1, EIF3A, EIF4G3, SFRS2IP, NCOA6	4.06E-02
32	34	71	3	Translational Initiation	1.03E-03	EIF3B, EIF3A, EIF4G3	4.73E-02
32	77	71	5	Nuclear Transport	8.78E-05	TPR, KHDRBS1, KPNB1, KPNA6, RANBP2	2.18E-02
32	56	71	4	Protein Import	3.23E-04	TPR, KPNB1, KPNA6, RANBP2	2.67E-02
32	32	71	3	Transcription Initiation	8.62E-04	SMARCA5, MED1, NCOA6	4.58E-02
32	263	71	8	Regulation Of Transcription From Rna Polymerase Ii Promoter	1.51E-04	SMARCA5, DNMT1, CHD4, MED1, NCOA6, CHD1, MAML1, DEK	2.18E-02
32	69	71	4	Ribonucleoprotein Complex Biogenesis And Assembly	7.18E-04	EIF3B, EIF3A, EIF4G3, SFRS2IP	4.23E-02
32	44	71	4	Protein Import Into Nucleus	1.26E-04	TPR, KPNB1, KPNA6, RANBP2	2.18E-02

32	56	71	4	Protein Rna Complex Assembly	3.23E-04	EIF3B, EIF3A, EIF4G3, SFRS2IP	2.67E-02
32	266	71	7	Cellular Component Assembly	9.44E-04	SMARCA5, EIF3B, MED1, EIF3A, EIF4G3, SFRS2IP, NCOA6	4.58E-02
32	201	71	6	Macromolecule Localization	1.17E-03	HNRNPA2B1, TPR, KHDRBS1, KPNB1, KPNA6, RANBP2	4.96E-02
32	77	71	5	Nucleocytoplasmic Transport	8.78E-05	TPR, KHDRBS1, KPNB1, KPNA6, RANBP2	2.18E-02
34	234	27	7	Dna Metabolic Process	6.10E-07	NASP, NOL8, SMC1A, DNMT1, ATF7IP, RFC1, TLK2	5.03E-04
34	105	27	4	Chromosome Organization And Biogenesis	8.21E-05	NASP, SMC1A, RFC1, TLK2	2.26E-02
37	19	48	5	Mitotic Cell Cycle Checkpoint	9.06E-09	BUB1, TTK, CCNA2, BUB1B, MAD2L1	6.79E-07
37	99	48	15	M Phase	1.97E-14	CHEK1, NCAPH, RAD54L, KIF15, BUB1, TTK, KIF2C, CCNA2, BIRC5, NEK2, KIF11, BUB1B, CENPE, MAD2L1, TPX2	2.70E-12
37	74	48	5	Microtubule Based Process	1.07E-05	KIF23, KIF4A, TTK, KIF2C, KIF11	5.17E-04
37	20	48	3	Dna Integrity Checkpoint	6.46E-05	CDC6, CHEK1, CCNA2	2.54E-03
37	33	48	7	Regulation Of Mitosis	4.00E-11	BUB1, TTK, CCNA2, BIRC5, NEK2, BUB1B, MAD2L1	3.67E-09
37	31	48	4	Microtubule Cytoskeleton Organization And Biogenesis	6.46E-06	KIF23, TTK, KIF2C, KIF11	3.33E-04
37	63	48	4	Interphase	1.11E-04	CDC6, CDK2, BIRC5, CENPF	4.00E-03
37	170	48	19	Cell Cycle Process	2.63E-14	CDC6, CHEK1, NCAPH, CDK2, KIF23, RAD54L, KIF15, BUB1, TTK, KIF2C, CCNA2, BIRC5, NEK2, KIF11, CENPF, BUB1B, CENPE, MAD2L1, TPX2	3.10E-12
37	134	48	17	Mitotic Cell Cycle	0.00E+00	CDC6, NCAPH, CDK2, KIF23, KIF15, BUB1, TTK, KIF2C, CCNA2, BIRC5, NEK2, KIF11, CENPF, BUB1B, CENPE, MAD2L1, TPX2	0.00E+00
37	153	48	18	Cell Cycle Phase	0.00E+00	CDC6, CHEK1, NCAPH, CDK2, RAD54L, KIF15, BUB1, TTK, KIF2C, CCNA2, BIRC5, NEK2, KIF11, CENPF, BUB1B, CENPE, MAD2L1, TPX2	0.00E+00
37	43	48	8	Cell Cycle Checkpoint Go 0000075	4.52E-12	CDC6, CHEK1, BUB1, TTK, CCNA2, BIRC5, BUB1B, MAD2L1	4.66E-10
37	28	48	4	Chromosome Segregation	4.24E-06	NCAPH, BRCA1, CENPF, CENPE	2.50E-04
37	15	48	3	Establishment Of Organelle Localization	2.62E-05	BIRC5, CENPF, CENPE	1.20E-03
37	19	48	3	Regulation Of Mitotic Cell Cycle	5.51E-05	CDC6, CDK2, BIRC5	2.27E-03
37	31	48	3	Dna Damage Responsesignal Transduction	2.47E-04	CHEK1, BRCA1, CCNA2	8.15E-03

37	44	48	3	Dna Recombination	7.02E-04	CHEK1, RAD54L, BRCA1	2.23E-02
37	8	48	3	Mitotic Spindle Organization And Biogenesis	3.28E-06	KIF23, TTK, KIF11	2.08E-04
37	70	48	13	Mitosis	0.00E+00	NCAPH, KIF15, BUB1, TTK, KIF2C, CCNA2, BIRC5, NEK2, KIF11, BUB1B, CENPE, MAD2L1, TPX2	0.00E+00
37	276	48	19	Cell Cycle Go 0007049	7.77E-15	CDC6, CHEK1, NCAPH, CDK2, KIF23, RAD54L, KIF15, BUB1, TTK, KIF2C, CCNA2, BIRC5, NEK2, KIF11, CENPF, BUB1B, CENPE, MAD2L1, TPX2	1.28E-12
37	19	48	3	Organelle Localization	5.51E-05	BIRC5, CENPF, CENPE	2.27E-03
37	181	48	5	Cytoskeleton Organization And Biogenesis	7.36E-04	KIF23, KIF4A, TTK, KIF2C, KIF11	2.25E-02
37	57	48	4	Interphase Of Mitotic Cell Cycle	7.52E-05	CDC6, CDK2, BIRC5, CENPF	2.82E-03
37	9	48	3	Spindle Organization And Biogenesis	4.91E-06	KIF23, TTK, KIF11	2.70E-04
37	157	48	10	Regulation Of Cell Cycle	4.55E-10	CDC6, CHEK1, CDK2, BUB1, TTK, CCNA2, BIRC5, NEK2, BUB1B, MAD2L1	3.75E-08
37	72	48	13	M Phase Of Mitotic Cell Cycle	0.00E+00	NCAPH, KIF15, BUB1, TTK, KIF2C, CCNA2, BIRC5, NEK2, KIF11, BUB1B, CENPE, MAD2L1, TPX2	0.00E+00
38	11	52	3	Microtubule Polymerization Or Depolymerization	1.22E-05	MAPT, CLASP2, APC	1.01E-02
39	41	42	3	Neurite Development	3.84E-04	MAPT, NRXN1, CDK5R1	3.96E-02
39	33	42	3	Axonogenesis	2.00E-04	MAPT, NRXN1, CDK5R1	3.30E-02
39	18	42	3	Regulation Of Mapkkk Cascade	3.10E-05	MAPK8IP3, TAOK3, HIPK2	6.40E-03
39	38	42	3	Cellular Morphogenesis During Differentiation	3.06E-04	MAPT, NRXN1, CDK5R1	3.60E-02
39	43	42	4	Jnk Cascade	1.43E-05	MAPK8IP3, TAOK3, MAPK10, HIPK2	4.31E-03
39	12	42	3	Regulation Of Jnk Cascade	8.49E-06	MAPK8IP3, TAOK3, HIPK2	4.31E-03
39	89	42	4	Mapkkk Cascade Go 0000165	2.52E-04	MAPK8IP3, TAOK3, MAPK10, HIPK2	3.46E-02
39	44	42	4	Stress Activated Protein Kinase Signaling Pathway	1.57E-05	MAPK8IP3, TAOK3, MAPK10, HIPK2	4.31E-03
40	234	84	19	Dna Metabolic Process	1.29E-13	TOP2A, NASP, FANCC, CDT1, EXO1, MSH5, RAD54L, POLD1, CHEK1, BRCA2, BRCA1, MCM2, MCM7, CDK2, TLK2, FEN1, MCM3, RFC4, DNMT1	1.52E-11
40	118	84	8	Dna Repair	1.57E-06	FANCC, EXO1, MSH5, RAD54L, POLD1, BRCA2, BRCA1, FEN1	5.18E-05
40	19	84	4	Mitotic Cell Cycle Checkpoint	7.88E-06	BUB1, KNTC1, BUB1B, TTK	2.32E-04
40	182	84	6	Negative Regulation Of Nucleobasenucleosidenucleotide And Nucleic Acid Metabolic Process	1.68E-03	TIMELESS, CDT1, BRCA1, CENPF, ILF3, DNMT1	3.30E-02
40	177	84	10	Response To Endogenous Stimulus	3.94E-07	FANCC, EXO1, MSH5, RAD54L, POLD1, CHEK1, BRCA2, BRCA1, MCM7, FEN1	1.55E-05

40	99	84	15	M Phase	7.88E-14	KIF15, NCAPH, BUB1, KNTC1, MSH5, RAD54L, KIF11, CHEK1, KIF2C, TPX2, BUB1B, SMC3, NDC80, TTK, PLK1	1.52E-11
40	74	84	8	Microtubule Based Process	4.14E-08	KIF11, KIF2C, KIF4A, SMC3, KIF5B, TTK, TUBG1, PRC1	2.14E-06
40	33	84	4	Regulation Of Mitosis	7.73E-05	BUB1, KNTC1, BUB1B, TTK	1.93E-03
40	31	84	6	Microtubule Cytoskeleton Organization And Biogenesis	6.16E-08	KIF11, KIF2C, SMC3, TTK, TUBG1, PRC1	2.99E-06
40	63	84	6	Interphase	4.76E-06	TIMELESS, SKP2, POLD1, CDK2, CENPF, CDC7	1.45E-04
40	170	84	24	Cell Cycle Process	1.23E-13	KIF15, NCAPH, BUB1, TIMELESS, KNTC1, MSH5, RAD54L, SKP2, POLD1, KIF11, CHEK1, BRCA2, KIF2C, TPX2, CDK2, CENPF, BUB1B, SMC3, CDC7, NDC80, TTK, TUBG1, PRC1, PLK1	1.52E-11
40	39	84	3	Regulation Of Dna Metabolic Process	2.50E-03	CDT1, CDK2, TLK2	4.63E-02
40	32	84	5	Meiotic Cell Cycle	2.54E-06	MSH5, RAD54L, CHEK1, SMC3, TUBG1	8.07E-05
40	134	84	18	Mitotic Cell Cycle	6.54E-14	KIF15, NCAPH, BUB1, KNTC1, SKP2, POLD1, KIF11, KIF2C, TPX2, CDK2, CENPF, BUB1B, SMC3, CDC7, NDC80, TTK, PRC1, PLK1	1.52E-11
40	153	84	21	Cell Cycle Phase	9.08E-14	KIF15, NCAPH, BUB1, TIMELESS, KNTC1, MSH5, RAD54L, SKP2, POLD1, KIF11, CHEK1, KIF2C, TPX2, CDK2, CENPF, BUB1B, SMC3, CDC7, NDC80, TTK, PLK1	1.52E-11
40	13	84	3	S Phase	8.90E-05	TIMELESS, POLD1, CDC7	2.16E-03
40	43	84	7	Cell Cycle Checkpoint Go 0000075	1.64E-08	CCNE2, BUB1, KNTC1, CDT1, CHEK1, BUB1B, TTK	9.48E-07
40	28	84	5	Chromosome Segregation	1.27E-06	TOP2A, NCAPH, BRCA1, CENPF, NDC80	4.55E-05
40	93	84	10	Dna Replication	7.95E-10	NASP, CDT1, EXO1, MSH5, POLD1, MCM2, MCM7, CDK2, MCM3, RFC4	6.56E-08
40	44	84	5	Dna Recombination	1.28E-05	EXO1, MSH5, RAD54L, CHEK1, BRCA1	3.65E-04
40	8	84	4	Mitotic Spindle Organization And Biogenesis	1.51E-07	KIF11, SMC3, TTK, PRC1	6.23E-06
40	70	84	12	Mitosis	1.04E-13	KIF15, NCAPH, BUB1, KNTC1, KIF11, KIF2C, TPX2, BUB1B, SMC3, NDC80, TTK, PLK1	1.52E-11

40	276	84	28	Cell Cycle Go 0007049	1.06E-13	CCNE2, KIF15, NCAPH, BUB1, NASP, TIMELESS, KNTC1, CDT1, MSH5, RAD54L, SKP2, POLD1, KIF11, CHEK1, BRCA2, MCM2, KIF2C, TPX2, CDK2, CENPF, BUB1B, SMC3, CDC7, NDC80, TTK, TUBG1, PRC1, PLK1	1.52E-11
40	181	84	8	Cytoskeleton Organization And Biogenesis	3.67E-05	KIF11, KIF2C, KIF4A, SMC3, KIF5B, TTK, TUBG1, PRC1	1.01E-03
40	105	84	8	Chromosome Organization And Biogenesis	6.43E-07	EZH2, TOP2A, NCAPH, NASP, HDAC2, CHAF1A, TLK2, NDC80	2.41E-05
40	74	84	4	Rna Splicing	1.75E-03	BAT1, SFRS1, SFPQ, PRPF3	3.36E-02
40	63	84	5	Establishment And Or Maintenance Of Chromatin Architecture	7.49E-05	EZH2, NASP, HDAC2, CHAF1A, TLK2	1.93E-03
40	51	84	6	Dna Dependent Dna Replication	1.35E-06	CDT1, EXO1, MSH5, CDK2, MCM3, RFC4	4.63E-05
40	57	84	5	Interphase Of Mitotic Cell Cycle	4.61E-05	SKP2, POLD1, CDK2, CENPF, CDC7	1.23E-03
40	27	84	3	Dna Packaging	8.49E-04	TOP2A, NCAPH, CHAF1A	1.89E-02
40	9	84	5	Spindle Organization And Biogenesis	1.81E-09	KIF11, SMC3, TTK, TUBG1, PRC1	1.33E-07
40	150	84	10	Response To Dna Damage Stimulus	8.35E-08	FANCC, EXO1, MSH5, RAD54L, POLD1, CHEK1, BRCA2, BRCA1, MCM7, FEN1	3.83E-06
40	157	84	10	Regulation Of Cell Cycle	1.29E-07	CCNE2, BUB1, TIMELESS, KNTC1, CDT1, CHEK1, CDK2, BUB1B, CDC7, TTK	5.58E-06
40	72	84	12	M Phase Of Mitotic Cell Cycle	1.66E-13	KIF15, NCAPH, BUB1, KNTC1, KIF11, KIF2C, TPX2, BUB1B, SMC3, NDC80, TTK, PLK1	1.71E-11
40	266	84	7	Regulation Of Cell Proliferation	2.52E-03	TIMELESS, CHEK1, BRCA2, BRCA1, CDK2, CDC7, TTK	4.63E-02
41	233	68	8	Defense Response	4.74E-05	CCL20, C5AR1, C2, HP, S100A8, STAB1, CEBPB, NOD2	9.77E-03
41	80	68	5	Locomotory Behavior	8.57E-05	CXCL5, CCL20, C5AR1, PLAUR, FPR1	1.41E-02
41	261	68	8	Response To Chemical Stimulus	1.05E-04	SOD2, CXCL5, STC1, CCL20, C5AR1, PLAUR, FPR1, NOD2	1.44E-02
41	21	68	3	Response To Bacterium	2.13E-04	SLC11A1, STAB1, NOD2	2.51E-02
41	207	68	9	Immune Response	2.26E-06	TREM1, IL1R2, CCL20, C5AR1, C2, CTSC, CEBPB, IL4R, IL6	9.31E-04
41	290	68	10	Immune System Process	4.76E-06	TREM1, IL1R2, CCL20, C5AR1, C2, CTSC, MAFB, CEBPB, IL4R, IL6	1.31E-03
41	269	68	12	Response To External Stimulus	2.89E-08	CXCL5, STC1, CCL20, C5AR1, C2, F13A1, PLAUR, S100A8, THBD, FPR1, CEBPB, NOD2	2.39E-05
41	168	68	6	Response To Wounding	3.61E-04	CCL20, C2, F13A1, S100A8, THBD, CEBPB	3.72E-02

41	174	68	6	Negative Regulation Of Developmental Process	4.35E-04	BIRC3, MAFB, STAB1, IER3, IL6, SRGN	3.99E-02
41	29	68	3	Humoral Immune Response	5.67E-04	TREM1, C2, IL6	4.67E-02
43	234	54	7	Dna Metabolic Process	7.78E-05	MCM2, RNASEH2A, DBF4, RFC4, TOP2A, KPNA2, RAD51	3.60E-03
43	19	54	3	Mitotic Cell Cycle Checkpoint	7.85E-05	MAD2L1, TTK, BUB1B	3.60E-03
43	99	54	10	M Phase	1.57E-11	BIRC5, MAD2L1, TTK, UBE2C, NDC80, BUB1B, KPNA2, AURKA, KIF2C, RAD51	2.59E-09
43	74	54	5	Microtubule Based Process	1.91E-05	KIF23, TTK, KPNA2, KIF4A, KIF2C	1.31E-03
43	33	54	5	Regulation Of Mitosis	3.24E-07	BIRC5, MAD2L1, TTK, UBE2C, BUB1B	2.97E-05
43	31	54	3	Microtubule Cytoskeleton Organization And Biogenesis	3.51E-04	KIF23, TTK, KIF2C	1.38E-02
43	63	54	5	Interphase	8.66E-06	BIRC5, CENPF, DBF4, CDKN3, KPNA2	6.49E-04
43	170	54	16	Cell Cycle Process	0.00E+00	BIRC5, CENPF, FBXO5, KIF23, MAD2L1, DBF4, TTK, UBE2C, NDC80, BUB1B, CDKN3, RACGAP1, KPNA2, AURKA, KIF2C, RAD51	0.00E+00
43	134	54	14	Mitotic Cell Cycle	0.00E+00	BIRC5, CENPF, FBXO5, KIF23, MAD2L1, DBF4, TTK, UBE2C, NDC80, BUB1B, CDKN3, KPNA2, AURKA, KIF2C	0.00E+00
43	153	54	13	Cell Cycle Phase	7.52E-14	BIRC5, CENPF, MAD2L1, DBF4, TTK, UBE2C, NDC80, BUB1B, CDKN3, KPNA2, AURKA, KIF2C, RAD51	1.55E-11
43	43	54	4	Cell Cycle Checkpoint Go 0000075	3.91E-05	BIRC5, MAD2L1, TTK, BUB1B	2.31E-03
43	28	54	3	Chromosome Segregation	2.58E-04	CENPF, TOP2A, NDC80	1.06E-02
43	93	54	5	Dna Replication	5.78E-05	MCM2, RNASEH2A, DBF4, RFC4, RAD51	3.18E-03
43	39	54	3	Regulation Of Cyclin Dependent Protein Kinase Activity	6.95E-04	CKS2, CKS1B, CDKN3	2.39E-02
43	70	54	8	Mitosis	7.27E-10	BIRC5, MAD2L1, TTK, UBE2C, NDC80, BUB1B, AURKA, KIF2C	9.99E-08
43	276	54	21	Cell Cycle Go 0007049	0.00E+00	BIRC5, CENPF, FBXO5, KIF23, MCM2, MAD2L1, DTYMK, DBF4, CKS2, CKS1B, TTK, UBE2C, NDC80, CDC20, BUB1B, CDKN3, RACGAP1, KPNA2, AURKA, KIF2C, RAD51	0.00E+00
43	181	54	6	Cytoskeleton Organization And Biogenesis	1.51E-04	KIF23, TTK, RACGAP1, KPNA2, KIF4A, KIF2C	6.54E-03
43	57	54	5	Interphase Of Mitotic Cell Cycle	5.26E-06	BIRC5, CENPF, DBF4, CDKN3, KPNA2	4.34E-04
43	157	54	9	Regulation Of Cell Cycle	2.79E-08	BIRC5, FBXO5, MAD2L1, CKS2, CKS1B, TTK, UBE2C, BUB1B, CDKN3	2.87E-06

43	72	54	8	M Phase Of Mitotic Cell Cycle	9.15E-10	BIRC5, MAD2L1, TTK, UBE2C, NDC80, BUB1B, AURKA, KIF2C	1.08E-07
44	138	43	5	Rna Processing	1.25E-04	PRPF31, CPSF6, CSTF2, DHX38, NONO	3.43E-02
44	61	43	5	Mrna Processing Go 0006397	2.34E-06	PRPF31, CPSF6, CSTF2, DHX38, NONO	1.93E-03
44	71	43	5	Mrna Metabolic Process	5.00E-06	PRPF31, CPSF6, CSTF2, DHX38, NONO	2.06E-03
45	104	95	6	Positive Regulation Of Transcriptiondna Dependent	1.68E-04	TCF4, BPTF, NCOA6, MAML1, RBM14, MED1	1.53E-02
45	59	95	4	Positive Regulation Of Transcription From Rna Polymerase Ii Promoter	1.19E-03	NCOA6, MAML1, RBM14, MED1	4.26E-02
45	249	95	8	Macromolecular Complex Assembly	7.71E-04	SMARCA5, GTF2I, ZW10, NCOA6, SFRS2IP, SFRS1, MED1, GTF2F1	3.03E-02
45	133	95	6	Positive Regulation Of Nucleobasenucleosidenucleotide And Nucleic Acid Metabolic Process	6.31E-04	TCF4, BPTF, NCOA6, MAML1, RBM14, MED1	2.74E-02
45	77	95	5	Nuclear Transport	3.47E-04	AKT1, KHDRBS1, KPNB1, XPO6, TPR	1.91E-02
45	106	95	6	Positive Regulation Of Rna Metabolic Process	1.86E-04	TCF4, BPTF, NCOA6, MAML1, RBM14, MED1	1.53E-02
45	93	95	5	Protein Targeting	8.26E-04	AKT1, KPNB1, XPO6, ATG4B, TPR	3.10E-02
45	32	95	5	Transcription Initiation	4.68E-06	SMARCA5, GTF2I, NCOA6, MED1, GTF2F1	9.64E-04
45	43	95	5	Protein Dna Complex Assembly	2.09E-05	SMARCA5, GTF2I, NCOA6, MED1, GTF2F1	2.87E-03
45	263	95	9	Regulation Of Transcription From Rna Polymerase Ii Promoter	2.25E-04	SMARCA5, TRIM27, BPTF, NCOA6, MAML1, RBM14, ZNF148, MED1, DNMT1	1.55E-02
45	138	95	8	Rna Processing	1.28E-05	CPSF6, KHDRBS1, SFRS2IP, ZNF638, SFRS1, U2AF1, SSB, PABPC4	2.10E-03
45	266	95	9	Cellular Component Assembly	2.45E-04	SMARCA5, HIP1, GTF2I, ZW10, NCOA6, SFRS2IP, SFRS1, MED1, GTF2F1	1.55E-02
45	61	95	4	Mrna Processing Go 0006397	1.35E-03	CPSF6, KHDRBS1, SFRS2IP, SFRS1	4.48E-02
45	27	95	4	Transcription Initiation From Rna Polymerase Ii Promoter	5.55E-05	GTF2I, NCOA6, MED1, GTF2F1	6.54E-03
45	71	95	5	Mrna Metabolic Process	2.37E-04	CPSF6, KHDRBS1, SFRS2IP, SFRS1, SSB	1.55E-02
45	105	95	5	Chromosome Organization And Biogenesis	1.43E-03	SMARCA5, ZW10, BPTF, RFC1, RBM14	4.52E-02
45	124	95	6	Positive Regulation Of Transcription	4.35E-04	TCF4, BPTF, NCOA6, MAML1, RBM14, MED1	2.24E-02
45	77	95	5	Nucleocytoplasmic Transport	3.47E-04	AKT1, KHDRBS1, KPNB1, XPO6, TPR	1.91E-02
46	138	60	7	Rna Processing	5.19E-06	PRPF4B, DIS3, SRPK1, EXOSC2, USP39, CPSF6, CSTF1	8.56E-04
46	61	60	4	Mrna Processing Go 0006397	2.35E-04	SRPK1, USP39, CPSF6, CSTF1	3.14E-02
46	71	60	6	Mrna Metabolic Process	1.32E-06	SRPK1, SMG1, USP39, GSPT1, CPSF6, CSTF1	3.63E-04

46	63	60	4	Establishment And Or Maintenance Of Chromatin Architecture	2.66E-04	SMARCC1, NASP, TLK2, SAFB	3.14E-02
47	249	59	9	Macromolecular Complex Assembly	3.05E-06	CHAF1A, GTF2F1, HCFC1, EIF3B, NCOA6, MED1, ATF7IP, EPRS, GTF2I	1.26E-03
47	77	59	4	Nuclear Transport	5.39E-04	KHDRBS1, XPO6, KPNB1, KPNA6	2.96E-02
47	32	59	4	Transcription Initiation	1.69E-05	GTF2F1, NCOA6, MED1, GTF2I	2.32E-03
47	43	59	6	Protein Dna Complex Assembly	5.65E-08	CHAF1A, GTF2F1, NCOA6, MED1, ATF7IP, GTF2I	4.66E-05
47	266	59	9	Cellular Component Assembly	5.23E-06	CHAF1A, GTF2F1, HCFC1, EIF3B, NCOA6, MED1, ATF7IP, EPRS, GTF2I	1.44E-03
47	27	59	4	Transcription Initiation From Rna Polymerase Ii Promoter	8.38E-06	GTF2F1, NCOA6, MED1, GTF2I	1.73E-03
47	77	59	4	Nucleocytoplasmic Transport	5.39E-04	KHDRBS1, XPO6, KPNB1, KPNA6	2.96E-02
49	40	41	3	Response To Virus	3.32E-04	TLR7, PTPRC, LILRB1	2.49E-02
49	128	41	4	Multi Organism Process	9.10E-04	CD4, TLR7, PTPRC, LILRB1	3.09E-02
49	124	41	4	Positive Regulation Of Cell Proliferation	8.08E-04	CD86, IL18, PTPRC, HCLS1	3.09E-02
49	62	41	3	Cell Activation	1.21E-03	CD4, IL18, PTPRC	3.11E-02
49	69	41	4	Hemopoiesis	8.52E-05	CD4, PTPRC, HCLS1, SPI1	1.54E-02
49	74	41	4	Immune System Development	1.12E-04	CD4, PTPRC, HCLS1, SPI1	1.54E-02
49	35	41	3	Cytokine Metabolic Process	2.22E-04	IL18, TLR7, TLR1	2.04E-02
49	233	41	7	Defense Response	1.19E-05	TLR7, CYBB, MNDA, PTPRC, RNASE6, CSF3R, CCR1	3.27E-03
49	150	41	4	Regulation Of Protein Metabolic Process	1.64E-03	TLR7, ITGB2, TLR1, HCLS1	3.65E-02
49	60	41	3	Cytokine Production	1.10E-03	IL18, TLR7, TLR1	3.11E-02
49	62	41	3	Positive Regulation Of Cellular Protein Metabolic Process	1.21E-03	TLR7, TLR1, HCLS1	3.11E-02
49	57	41	3	Leukocyte Activation	9.44E-04	CD4, IL18, PTPRC	3.09E-02
49	207	41	9	Immune Response	2.40E-08	FYB, NCF4, CD86, IL18, TLR7, CTSS, LCP2, PTPRC, CCR1	9.89E-06
49	71	41	4	Hemopoietic Or Lymphoid Organ Development	9.53E-05	CD4, PTPRC, HCLS1, SPI1	1.54E-02
49	63	41	3	Response To Other Organism	1.26E-03	TLR7, PTPRC, LILRB1	3.11E-02
49	172	41	4	Homeostatic Process	2.70E-03	PTPRC, HCLS1, SPI1, CCR1	4.80E-02
49	290	41	14	Immune System Process	3.06E-13	FYB, NCF4, CD4, SYK, CD86, IL18, TLR7, CTSS, ITGB2, LCP2, PTPRC, HCLS1, SPI1, CCR1	2.53E-10
49	39	41	3	T Cell Activation	3.07E-04	CD4, IL18, PTPRC	2.49E-02
49	34	41	3	Cytokine Biosynthetic Process	2.04E-04	IL18, TLR7, TLR1	2.04E-02
49	64	41	3	Positive Regulation Of Protein Metabolic Process	1.32E-03	TLR7, TLR1, HCLS1	3.11E-02
49	52	41	3	Lymphocyte Activation	7.21E-04	CD4, IL18, PTPRC	3.09E-02
49	139	41	4	Regulation Of Cellular Protein Metabolic Process	1.24E-03	TLR7, ITGB2, TLR1, HCLS1	3.11E-02
51	40	88	4	Response To Virus	1.99E-04	PTPRC, TLR7, LILRB1, APOBEC3G	1.28E-02
51	95	88	5	Positive Regulation Of Signal Transduction	6.42E-04	PTPRC, LGALS9, CASP1, LYN, HMOX1	3.12E-02

51	128	88	6	Multi Organism Process	3.41E-04	PTPRC, CD4, TLR7, LILRB1, HLA-DPB1, APOBEC3G	1.88E-02
51	69	88	5	Hemopoiesis	1.45E-04	PTPRC, SCIN, CD4, MAFB, LYN	1.28E-02
51	74	88	5	Immune System Development	2.01E-04	PTPRC, SCIN, CD4, MAFB, LYN	1.28E-02
51	233	88	13	Defense Response	1.27E-08	PTPRC, LY96, ADORA3, MNDA, CCR1, AIF1, C3AR1, NCF2, TLR7, RNASE6, CYBB, LYZ, APOBEC3G	3.49E-06
51	60	88	5	Cytokine Production	7.40E-05	IL18, PYCARD, SRGN, TLR1, TLR7	8.72E-03
51	11	88	3	Leukocyte Chemotaxis	5.96E-05	SYK, ITGB2, DOCK2	8.20E-03
51	10	88	3	Defense Response To Virus	4.36E-05	PTPRC, TLR7, APOBEC3G	7.20E-03
51	113	88	6	Inflammatory Response	1.73E-04	ADORA3, CCR1, AIF1, C3AR1, CYBB, LYZ	1.28E-02
51	207	88	13	Immune Response	3.04E-09	FYB, CD86, IL18, PTPRC, TREM2, IGSF6, LCP2, CCR1, FCGR1A, CTSS, TLR7, NCF4, APOBEC3G	1.25E-06
51	71	88	5	Hemopoietic Or Lymphoid Organ Development	1.66E-04	PTPRC, SCIN, CD4, MAFB, LYN	1.28E-02
51	63	88	4	Response To Other Organism	1.14E-03	PTPRC, TLR7, LILRB1, APOBEC3G	4.86E-02
51	290	88	20	Immune System Process	0.00E+00	FYB, SYK, CD86, IL18, PTPRC, ITGB2, DOCK2, SCIN, TREM2, IGSF6, LCP2, CCR1, CD4, FCGR1A, CTSS, TLR7, NCF4, MAFB, LYN, APOBEC3G	0.00E+00
51	269	88	10	Response To External Stimulus	2.55E-05	SYK, ITGB2, DOCK2, RNASE2, ADORA3, CCR1, AIF1, C3AR1, CYBB, LYZ	5.26E-03
51	19	88	3	Rna Catabolic Process	3.36E-04	RNASE2, RNASE3, RNASE6	1.88E-02
51	13	88	3	Leukocyte Migration	1.02E-04	SYK, ITGB2, DOCK2	1.05E-02
