# Supplementary material for: A Computational Approach to Identifying Gene-microRNA Modules in Cancer
Source: PLoS Comput Biol. 2015 Jan 22;11(1):e1004042. doi: 10.1371/journal.pcbi.1004042 (PMC4303261; doi:10.1371/journal.pcbi.1004042)
Supplement: S19 Table — The significant numbers of genes in each module are enriched in KEGG pathways, and the significance is shown in ‘p-value’. ‘m’, ‘k’, and ‘x’ represent the number of genes in the corresponding pathway, the number of genes in the module, and the number of genes belonging to the pathway in the module, respectively. (PDF) [file pcbi.1004042.s026.pdf]

Table S19. GBM modules with enriched pathways in KEGG.

Module ID	m	k	x	Gene Ontology	p-value	Genes	p-value
2	32	98	6	Dna Replication	1.90E-07	MCM2, MCM4, POLA2, MCM3, MCM7, MCM6	1.77E-05
2	111	98	19	Cell Cycle	0.00E+00	TTK, ESPL1, BUB1, CDC7, ORC6L, BUB1B, PLK1, CHEK1, CCNA2, MCM2, MCM4, CDC6, CDC25C, MCM3, BUB3, MCM7, CDC20, MAD2L1, MCM6	0.00E+00
2	92	98	8	Oocyte Meiosis	7.65E-07	ESPL1, AURKA, BUB1, FBXO5, PLK1, CDC25C, CDC20, MAD2L1	4.74E-05
2	73	98	5	Progesterone Mediated Oocyte Maturation	3.12E-04	BUB1, PLK1, CCNA2, CDC25C, MAD2L1	1.45E-02
4	90	41	5	Toll Like Receptor Signaling Pathway	1.26E-05	CXCL11, IRF7, STAT1, TLR3, CXCL10	2.35E-03
5	32	56	5	Dna Replication	3.32E-07	LIG1, MCM7, MCM2, MCM4, MCM3	3.08E-05
5	111	56	9	Cell Cycle	1.83E-09	CHEK1, MCM7, CDK2, MCM2, MCM4, MCM3, TTK, PLK1, BUB1B	3.40E-07
7	98	43	4	Spliceosome	3.99E-04	LSM4, SFRS3, SNRPD1, SFRS2	2.47E-02
7	111	43	6	Cell Cycle	2.47E-06	MAD2L1, DBF4, CCNB1, PCNA, ORC4L, BUB1B	4.60E-04
7	92	43	4	Oocyte Meiosis	3.13E-04	MAD2L1, CCNB1, AURKA, PPP1CC	2.47E-02
8	236	73	7	Mapk Signaling Pathway	5.51E-04	PLA2G2A, IL1R2, HSPA6, IL1B, CD14, MAP3K8, FAS	1.14E-02
8	218	73	12	Cytokine Cytokine Receptor Interaction	6.37E-09	CXCL5, IL1R2, CCL20, IL10RA, TNFRSF1B, CCR5, IL1B, IL4R, CCR1, CCL2, FAS, IL13RA1	5.93E-07
8	156	73	9	Chemokine Signaling Pathway	3.90E-07	CXCL5, CCL20, HCK, CCR5, NCF1, WAS, CCR1, CCL2, LYN	2.42E-05
8	90	73	4	Toll Like Receptor Signaling Pathway	2.14E-03	IL1B, CD14, TLR1, MAP3K8	3.99E-02
8	78	73	6	Hematopoietic Cell Lineage	7.33E-06	IL1R2, IL1B, CD14, IL4R, ITGAM, CD44	2.73E-04
8	108	73	5	Natural Killer Cell Mediated Cytotoxicity	4.89E-04	ICAM1, PTPN6, SYK, PLCG2, FAS	1.14E-02
8	68	73	6	B Cell Receptor Signaling Pathway	3.28E-06	PTPN6, FCGR2B, SYK, BTK, PLCG2, LYN	1.52E-04
8	72	73	5	Fc Epsilon Ri Signaling Pathway	7.27E-05	PLA2G2A, SYK, BTK, PLCG2, LYN	1.93E-03
8	84	73	9	Fc Gamma R Mediated Phagocytosis	1.70E-09	HCK, NCF1, FCGR2B, WAS, SYK, PLCG2, LYN, PTPRC, FCGR2A	3.17E-07
8	63	73	5	Leishmania Infection	3.81E-05	IL1B, NCF1, PTPN6, ITGAM, FCGR2A	1.18E-03
10	32	73	4	Dna Replication	3.93E-05	FEN1, MCM3, MCM4, MCM2	3.66E-03
10	111	73	12	Cell Cycle	2.38E-12	CHEK1, ORC2L, CDK2, PLK1, BUB1B, SKP2, TFDP2, MCM3, HDAC1, MCM4, CCNA2, MCM2	4.43E-10

11	236	89	13	Mapk Signaling Pathway	1.70E-08	PLA2G5, BDNF, FGF14, FGF2, FAS, IKBKB, JUN, FGFR4, TNFRSF1A, RPS6KA3, RELB, MAPK1, FLNA	3.16E-06
11	80	89	6	Erbb Signaling Pathway	2.66E-05	SHC1, JUN, ERBB2, CBLC, MAPK1, CDKN1A	6.20E-04
11	156	89	6	Chemokine Signaling Pathway	1.03E-03	SHC1, PXN, PTK2B, IKBKB, MAPK1, TIAM1	1.47E-02
11	60	89	5	P53 Signaling Pathway	7.81E-05	BAX, TNFRSF10B, FAS, DDB2, CDKN1A	1.45E-03
11	79	89	8	Apoptosis	1.10E-07	BAX, TNFRSF10B, CFLAR, FAS, IKBKB, BCL2L1, TNFRSF1A, MYD88	6.82E-06
11	90	89	5	Toll Like Receptor Signaling Pathway	5.28E-04	IKBKB, JUN, TICAM1, MYD88, MAPK1	8.19E-03
11	108	89	5	Natural Killer Cell Mediated Cytotoxicity	1.21E-03	SHC1, PTK2B, TNFRSF10B, FAS, MAPK1	1.60E-02
11	116	89	7	Neurotrophin Signaling Pathway	2.30E-05	BAX, SHC1, BDNF, IKBKB, JUN, RPS6KA3, MAPK1	6.12E-04
11	182	89	10	Regulation Of Actin Cytoskeleton	8.80E-07	PDGFD, ITGA2, ITGA3, PXN, FGF14, FGF2, IQGAP1, FGFR4, MAPK1, TIAM1	3.44E-05
11	90	89	4	Gnrh Signaling Pathway	4.39E-03	PLA2G5, PTK2B, JUN, MAPK1	4.54E-02
11	70	89	4	Pancreatic Cancer	1.76E-03	IKBKB, BCL2L1, ERBB2, MAPK1	2.19E-02
11	85	89	6	Prostate Cancer	3.77E-05	PDGFD, IKBKB, ERBB2, AR, MAPK1, CDKN1A	7.78E-04
11	66	89	5	Melanoma	1.23E-04	PDGFD, FGF14, FGF2, MAPK1, CDKN1A	2.09E-03
11	41	89	3	Bladder Cancer	3.40E-03	ERBB2, MAPK1, CDKN1A	3.72E-02
11	70	89	6	Chronic Myeloid Leukemia	1.23E-05	SHC1, IKBKB, BCL2L1, CBLC, MAPK1, CDKN1A	3.82E-04
11	81	89	4	Small Cell Lung Cancer	3.01E-03	ITGA2, ITGA3, IKBKB, BCL2L1	3.50E-02
12	183	47	15	Focal Adhesion	0.00E+00	COL5A1, COL4A1, LAMC1, COL1A1, COL4A2, COL5A2, LAMB1, LAMC3, PDGFRB, ITGA5, COL6A3, ITGB1, COL1A2, COL3A1, FN1	0.00E+00
12	78	47	14	Ecm Receptor Interaction	2.83E-14	COL5A1, COL4A1, LAMC1, COL1A1, COL4A2, COL5A2, LAMB1, LAMC3, ITGA5, COL6A3, ITGB1, COL1A2, COL3A1, FN1	2.63E-12
12	182	47	5	Regulation Of Actin Cytoskeleton	6.85E-04	PDGFRB, ITGA5, ITGB1, MYH9, FN1	2.55E-02
12	81	47	7	Small Cell Lung Cancer	2.42E-08	COL4A1, LAMC1, COL4A2, LAMB1, LAMC3, ITGB1, FN1	1.50E-06
13	43	91	4	Rna Degradation	3.01E-04	LSM2, EXOSC5, EXOSC9, LSM4	1.86E-02
13	32	91	4	Dna Replication	9.33E-05	RFC2, RNASEH2A, RFC5, PCNA	1.74E-02
13	98	91	5	Spliceosome	8.62E-04	USP39, LSM2, SFRS3, SNRPD1, LSM4	3.21E-02
13	41	91	4	Nucleotide Excision Repair	2.50E-04	RFC2, ERCC8, RFC5, PCNA	1.86E-02
13	23	91	3	Mismatch Repair	6.63E-04	RFC2, RFC5, PCNA	3.08E-02

14	183	65	8	Focal Adhesion	5.88E-06	CAV1, VEGFA, THBS1, ITGA5, FLNA, SHC1, LAMC1, LAMB1	1.09E-03
14	78	65	4	Ecm Receptor Interaction	8.17E-04	THBS1, ITGA5, LAMC1, LAMB1	3.80E-02
14	65	65	5	Complement And Coagulation Cascades	2.52E-05	PLAU, C5AR1, SERPINE1, PLAUR, THBD	1.81E-03
14	67	65	5	Antigen Processing And Presentation	2.92E-05	HSPA6, HSPA5, CTSB, CTSL1, PDIA3	1.81E-03
16	32	93	4	Dna Replication	1.02E-04	MCM4, POLD1, POLA2, FEN1	2.70E-03
16	31	93	4	Base Excision Repair	8.94E-05	NEIL3, LIG3, POLD1, FEN1	2.70E-03
16	27	93	4	Homologous Recombination	5.11E-05	RAD51, RAD54L, BRCA2, POLD1	2.37E-03
16	111	93	17	Cell Cycle	3.38E-14	CCNA2, ESPL1, CDC6, CHEK1, BUB1, CHEK2, PLK1, MCM4, BUB1B, SKP2, ORC1L, CDC25C, CCNB2, TTK, CDK2, CDC20, CDC25A	6.28E-12
16	92	93	8	Oocyte Meiosis	5.11E-07	ESPL1, AURKA, BUB1, PLK1, CDC25C, CCNB2, CDK2, CDC20	4.75E-05
16	60	93	5	P53 Signaling Pathway	9.63E-05	GTSE1, CHEK1, CHEK2, CCNB2, CDK2	2.70E-03
16	73	93	7	Progesterone Mediated Oocyte Maturation	1.40E-06	CCNA2, BUB1, PLK1, CDC25C, CCNB2, CDK2, CDC25A	8.70E-05
17	183	59	18	Focal Adhesion	8.88E-16	COL5A1, COL6A2, THBS1, COL6A1, COL1A1, COL4A2, COL5A2, LAMB1, FN1, LAMC3, MYL9, PDGFRB, ITGA5, COL6A3, ITGB1, COL1A2, COL3A1, ITGA4	1.65E-13
17	78	59	16	Ecm Receptor Interaction	1.72E-14	COL5A1, COL6A2, THBS1, COL6A1, COL1A1, COL4A2, COL5A2, LAMB1, FN1, LAMC3, ITGA5, COL6A3, ITGB1, COL1A2, COL3A1, ITGA4	1.60E-12
17	100	59	4	Leukocyte Transendothelial Migration	1.44E-03	MMP9, MYL9, ITGB1, ITGA4	3.34E-02
17	182	59	8	Regulation Of Actin Cytoskeleton	2.68E-06	FN1, MYL9, PDGFRB, ITGA5, ITGB1, MYH9, ITGA4, SLC9A1	1.66E-04
17	81	59	5	Small Cell Lung Cancer	4.58E-05	COL4A2, LAMB1, FN1, LAMC3, ITGB1	1.42E-03
17	76	59	5	Hypertrophic Cardiomyopathy Hcm	3.36E-05	TPM2, ITGA5, ITGB1, ITGA4, TPM4	1.42E-03
17	81	59	5	Dilated Cardiomyopathy	4.58E-05	TPM2, ITGA5, ITGB1, ITGA4, TPM4	1.42E-03
21	60	70	4	P53 Signaling Pathway	3.99E-04	SERPINE1, CDKN1A, FAS, TNFRSF10B	3.36E-02
21	183	70	9	Focal Adhesion	1.04E-06	ZYX, ITGA5, LAMC1, FLNA, SHC1, ACTN1, PARVA, LAMB1, COL5A2	1.93E-04
21	65	70	4	Complement And Coagulation Cascades	5.43E-04	PLAU, SERPINE1, PLAUR, C5AR1	3.36E-02
24	218	38	5	Cytokine Cytokine Receptor Interaction	5.73E-04	TGFBR1, TGFBR2, OSMR, CSF2RB, IL13RA1	2.59E-02

24	156	38	4	Chemokine Signaling Pathway	1.42E-03	NFKB1, PRKCD, JAK2, HCK	2.93E-02
24	108	38	4	Lysosome	3.57E-04	GM2A, CTSS, NAGA, HEXA	2.59E-02
24	79	38	3	Apoptosis	1.94E-03	RIPK1, NFKB1, CSF2RB	3.61E-02
24	90	38	3	Toll Like Receptor Signaling Pathway	2.82E-03	RIPK1, NFKB1, TLR2	4.37E-02
24	59	38	3	Rig I Like Receptor Signaling Pathway	8.34E-04	RIPK1, DDX3X, NFKB1	2.59E-02
24	128	38	4	Jak Stat Signaling Pathway	6.80E-04	OSMR, CSF2RB, JAK2, IL13RA1	2.59E-02
24	84	38	3	Fc Gamma R Mediated Phagocytosis	2.32E-03	PRKCD, PTPRC, HCK	3.92E-02
24	63	38	5	Leishmania Infection	1.47E-06	NFKB1, HLA-DQB1, JAK2, NCF2, TLR2	2.73E-04
24	70	38	3	Pancreatic Cancer	1.37E-03	TGFBR1, TGFBR2, NFKB1	2.93E-02
24	70	38	3	Chronic Myeloid Leukemia	1.37E-03	TGFBR1, TGFBR2, NFKB1	2.93E-02
25	183	75	7	Focal Adhesion	1.39E-04	VEGFA, IBSP, ACTN1, BIRC3, FLNA, THBS1, ITGA5	1.29E-02
25	65	75	5	Complement And Coagulation Cascades	5.06E-05	PLAU, SERPINE1, F13A1, C5AR1, PLAUR	9.40E-03
26	116	119	7	Cell Adhesion Molecules Cams	1.48E-04	NCAM1, NLGN1, ICAM1, CLDN4, NRXN2, NRXN1, F11R	2.75E-02
27	111	53	10	Cell Cycle	4.10E-11	CCNA2, TTK, CCNB2, CDC20, MCM2, CCNB1, MAD2L1, BUB1B, PTTG1, CDC25C	7.63E-09
27	92	53	7	Oocyte Meiosis	1.40E-07	CCNB2, AURKA, CDC20, CCNB1, MAD2L1, PTTG1, CDC25C	1.30E-05
27	73	53	5	Progesterone Mediated Oocyte Maturation	1.63E-05	CCNA2, CCNB2, CCNB1, MAD2L1, CDC25C	1.01E-03
28	108	55	8	Lysosome	2.77E-08	HEXB, CTSA, TCIRG1, CTSB, CTSZ, NAGA, CTSS, GM2A	5.14E-06
28	65	55	4	Complement And Coagulation Cascades	2.14E-04	SERPINA1, F13A1, PLAUR, C5AR1	8.97E-03
28	67	55	4	Antigen Processing And Presentation	2.41E-04	CTSB, IFI30, CTSS, CD4	8.97E-03
28	84	55	4	Fc Gamma R Mediated Phagocytosis	5.73E-04	ARPC1B, FCGR2B, HCK, SYK	1.78E-02
28	100	55	7	Leukocyte Transendothelial Migration	3.23E-07	ITGB2, SIPA1, CYBA, GNAI2, ITGAM, CXCR4, CYBB	3.00E-05
28	63	55	4	Leishmania Infection	1.90E-04	ITGB2, TGFB1, CYBA, ITGAM	8.97E-03
29	32	30	4	Dna Replication	1.07E-06	POLE2, RNASEH2A, PCNA, MCM2	9.97E-05
29	31	30	3	Base Excision Repair	5.98E-05	POLE2, NEIL3, PCNA	3.71E-03
29	111	30	8	Cell Cycle	2.00E-10	CDC7, CDKN2C, ORC6L, BUB1B, PCNA, TTK, MCM2, CCNB2	3.71E-08
37	111	48	8	Cell Cycle	1.12E-08	CDC6, CHEK1, CDK2, BUB1, TTK, CCNA2, BUB1B, MAD2L1	2.09E-06
37	73	48	4	Progesterone Mediated Oocyte Maturation	1.98E-04	CDK2, BUB1, CCNA2, MAD2L1	1.84E-02
40	32	84	9	Dna Replication	6.81E-13	POLD1, MCM2, MCM7, MCM4, RFC5, FEN1, POLD3, MCM3, RFC4	6.33E-11

40	31	84	3	Base Excision Repair	1.28E-03	POLD1, FEN1, POLD3	3.40E-02
40	41	84	4	Nucleotide Excision Repair	1.83E-04	POLD1, RFC5, POLD3, RFC4	6.82E-03
40	23	84	5	Mismatch Repair	4.46E-07	EXO1, POLD1, RFC5, POLD3, RFC4	2.77E-05
40	27	84	5	Homologous Recombination	1.05E-06	RAD54L, POLD1, BRCA2, MUS81, POLD3	4.87E-05
40	111	84	16	Cell Cycle	8.89E-14	CCNE2, BUB1, HDAC2, SKP2, CHEK1, MCM2, MCM7, MCM4, CDK2, BUB1B, SMC3, CDC7, TTK, MCM3, BUB3, PLK1	1.65E-11
40	92	84	5	Oocyte Meiosis	4.48E-04	CCNE2, BUB1, CDK2, SMC3, PLK1	1.39E-02
41	218	68	7	Cytokine Cytokine Receptor Interaction	2.20E-04	CXCL5, IL1R2, CCL20, IL10RA, TNFRSF1B, IL4R, IL6	1.02E-02
41	65	68	5	Complement And Coagulation Cascades	3.14E-05	C5AR1, C2, F13A1, PLAUR, THBD	2.92E-03
41	90	68	5	Toll Like Receptor Signaling Pathway	1.50E-04	MAP3K8, CD14, TLR2, MAPK13, IL6	9.30E-03
41	52	68	5	Nod Like Receptor Signaling Pathway	1.05E-05	BIRC3, MAPK13, NOD2, IL6, TNFAIP3	1.95E-03
41	78	68	4	Hematopoietic Cell Lineage	9.69E-04	IL1R2, CD14, IL4R, IL6	3.60E-02
43	32	54	4	Dna Replication	1.18E-05	MCM2, PCNA, RNASEH2A, RFC4	7.34E-04
43	111	54	10	Cell Cycle	5.00E-11	CCNB2, MCM2, PCNA, CCNB1, MAD2L1, DBF4, PTTG1, TTK, CDC20, BUB1B	9.30E-09
43	92	54	7	Oocyte Meiosis	1.59E-07	CCNB2, FBXO5, CCNB1, MAD2L1, PTTG1, CDC20, AURKA	1.48E-05
48	90	68	5	Toll Like Receptor Signaling Pathway	1.50E-04	CASP8, LY96, TLR1, TLR5, CD86	4.65E-03
48	39	68	4	Type I Diabetes Mellitus	6.59E-05	HLA-B, HLA-E, FAS, CD86	3.06E-03
48	42	68	4	Autoimmune Thyroid Disease	8.86E-05	HLA-B, HLA-E, FAS, CD86	3.29E-03
48	33	68	4	Allograft Rejection	3.36E-05	HLA-B, HLA-E, FAS, CD86	2.09E-03
48	32	68	4	Graft Versus Host Disease	2.97E-05	HLA-B, HLA-E, FAS, CD86	2.09E-03
48	65	68	5	Viral Myocarditis	3.14E-05	HLA-B, ITGB2, HLA-E, CASP8, CD86	2.09E-03
49	218	41	5	Cytokine Cytokine Receptor Interaction	8.19E-04	IL18, IL10RA, CSF3R, CCR1, CSF1R	2.54E-02
49	116	41	6	Cell Adhesion Molecules Cams	2.40E-06	CD4, CD86, ITGB2, PTPRC, HLA-DMA, ITGAM	2.23E-04
49	67	41	3	Antigen Processing And Presentation	1.51E-03	CD4, CTSS, HLA-DMA	3.51E-02
49	78	41	4	Hematopoietic Cell Lineage	1.38E-04	CD4, CSF3R, ITGAM, CSF1R	6.39E-03
49	100	41	4	Leukocyte Transendothelial Migration	3.58E-04	NCF4, CYBB, ITGB2, ITGAM	1.33E-02
49	63	41	5	Leishmania Infection	2.16E-06	NCF4, ITGB2, TLR2, HLA-DMA, ITGAM	2.23E-04
49	65	41	3	Viral Myocarditis	1.38E-03	CD86, ITGB2, HLA-DMA	3.51E-02
51	156	88	6	Chemokine Signaling Pathway	9.70E-04	DOCK2, HCK, CCR1, LYN, PIK3CG, RAC2	1.06E-02
51	116	88	7	Cell Adhesion Molecules Cams	2.14E-05	CD86, PTPRC, ITGB2, HLA-DQA1, CD4, HLA-DMB, HLA-DPB1	4.42E-04
51	65	88	4	Complement And Coagulation Cascades	1.28E-03	C1QA, C1QB, SERPINA1, C3AR1	1.33E-02

51	67	88	5	Antigen Processing And Presentation	1.26E-04	HLA-DQA1, CD4, CTSS, HLA-DMB, HLA-DPB1	1.93E-03
51	90	88	7	Toll Like Receptor Signaling Pathway	4.00E-06	CD86, LY96, TLR2, TLR1, CD14, TLR7, PIK3CG	1.49E-04
51	42	88	3	Cytosolic Dna Sensing Pathway	3.52E-03	IL18, PYCARD, CASP1	2.98E-02
51	78	88	4	Hematopoietic Cell Lineage	2.52E-03	CD4, FCGR1A, CD14, CSF1R	2.23E-02
51	108	88	6	Natural Killer Cell Mediated Cytotoxicity	1.35E-04	SYK, ITGB2, FCER1G, LCP2, PIK3CG, RAC2	1.93E-03
51	68	88	4	B Cell Receptor Signaling Pathway	1.52E-03	SYK, LYN, PIK3CG, RAC2	1.49E-02
51	72	88	6	Fc Epsilon Ri Signaling Pathway	1.36E-05	SYK, FCER1G, LCP2, LYN, PIK3CG, RAC2	3.16E-04
51	84	88	10	Fc Gamma R Mediated Phagocytosis	4.57E-10	SYK, PTPRC, DOCK2, SCIN, HCK, FCGR1A, FCGR2A, LYN, PIK3CG, RAC2	4.25E-08
51	100	88	7	Leukocyte Transendothelial Migration	8.07E-06	ITGB2, CYBA, NCF2, CYBB, NCF4, PIK3CG, RAC2	2.14E-04
51	39	88	4	Intestinal Immune Network For Iga Production	1.80E-04	CD86, HLA-DQA1, HLA-DMB, HLA-DPB1	2.23E-03
51	182	88	6	Regulation Of Actin Cytoskeleton	2.13E-03	NCKAP1L, ITGB2, SCIN, CD14, PIK3CG, RAC2	1.98E-02
51	39	88	4	Type I Diabetes Mellitus	1.80E-04	CD86, HLA-DQA1, HLA-DMB, HLA-DPB1	2.23E-03
51	63	88	10	Leishmania Infection	2.39E-11	ITGB2, HLA-DQA1, CYBA, TLR2, FCGR1A, FCGR2A, NCF2, NCF4, HLA-DMB, HLA-DPB1	4.44E-09
51	25	88	5	Asthma	8.80E-07	RNASE3, HLA-DQA1, FCER1G, HLA-DMB, HLA-DPB1	4.09E-05
51	42	88	4	Autoimmune Thyroid Disease	2.41E-04	CD86, HLA-DQA1, HLA-DMB, HLA-DPB1	2.80E-03
51	97	88	8	Systemic Lupus Erythematosus	5.01E-07	CD86, C1QA, HLA-DQA1, C1QB, FCGR1A, FCGR2A, HLA-DMB, HLA-DPB1	3.11E-05
51	33	88	4	Allograft Rejection	9.27E-05	CD86, HLA-DQA1, HLA-DMB, HLA-DPB1	1.57E-03
51	32	88	4	Graft Versus Host Disease	8.19E-05	CD86, HLA-DQA1, HLA-DMB, HLA-DPB1	1.52E-03
51	65	88	6	Viral Myocarditis	7.49E-06	CD86, ITGB2, HLA-DQA1, HLA-DMB, HLA-DPB1, RAC2	2.14E-04
52	182	57	6	Regulation Of Actin Cytoskeleton	2.10E-04	PFN2, PAK7, FGF12, PAK3, CYFIP2, WASF1	3.91E-02
54	218	35	5	Cytokine Cytokine Receptor Interaction	3.87E-04	TNFSF10, LEPR, CSF3R, IL7R, TGFBR2	4.59E-02
54	128	35	4	Jak Stat Signaling Pathway	4.94E-04	LEPR, CSF3R, IL7R, STAT4	4.59E-02
