# Supplementary material for: A Computational Approach to Identifying Gene-microRNA Modules in Cancer
Source: PLoS Comput Biol. 2015 Jan 22;11(1):e1004042. doi: 10.1371/journal.pcbi.1004042 (PMC4303261; doi:10.1371/journal.pcbi.1004042)
Supplement: S20 Table — The significant numbers of genes in each module are enriched in BioCarta pathways, and the significance is shown in ‘p-value’. ‘m’, ‘k’, and ‘x’ represent the number of genes in the corresponding pathway, the number of genes in the module, and the number of genes belonging to the pathway in the module, respectively. (PDF) [file pcbi.1004042.s027.pdf]

Table S20. GBM modules with enriched pathways in BioCarta.

Module ID	m	k	x	Gene Ontology	p-value	Genes	p-value
2	20	98	3	Atm Pathway	5.39E-04	RAD51, CHEK1, BRCA1	2.34E-02
2	18	98	8	Mcm Pathway	5.43E-13	CDT1, ORC6L, MCM2, MCM4, CDC6, MCM3, MCM7, MCM6	1.18E-10
2	23	98	4	G2 Pathway	3.24E-05	PLK1, CHEK1, BRCA1, CDC25C	2.35E-03
2	20	98	3	Atrbrca Pathway	5.39E-04	RAD51, CHEK1, BRCA1	2.34E-02
2	9	98	4	Ranms Pathway	5.04E-07	KIF15, AURKA, KPNA2, TPX2	5.47E-05
3	14	101	3	Akapcentrosome Pathway	1.95E-04	AKAP9, PRKAR2A, PCNT	4.23E-02
5	18	56	5	Mcm Pathway	1.48E-08	MCM7, CDK2, MCM2, MCM4, MCM3	3.22E-06
5	20	56	3	Atrbrca Pathway	1.03E-04	CHEK1, FANCC, RAD51	1.11E-02
8	11	73	3	Blymphocyte Pathway	3.41E-05	ICAM1, FCGR2B, PTPRC	3.70E-03
8	31	73	3	Bcr Pathway	8.51E-04	SYK, BTK, LYN	4.29E-02
8	35	73	3	Fcer1 Pathway	1.22E-03	SYK, BTK, LYN	4.29E-02
8	37	73	3	Il2Rb Pathway	1.43E-03	PTPN6, SYK, FAS	4.29E-02
8	10	73	3	Monocyte Pathway	2.49E-05	ICAM1, ITGAM, CD44	3.70E-03
8	27	73	3	Nkt Pathway	5.63E-04	CCR5, IL4R, CCR1	4.07E-02
10	18	73	6	Mcm Pathway	7.05E-10	ORC2L, CDK2, CDT1, MCM3, MCM4, MCM2	1.53E-07
10	27	73	3	G1 Pathway	5.63E-04	CDK2, SKP2, HDAC1	4.07E-02
10	12	73	3	Rb Pathway	4.52E-05	CHEK1, CDK2, MAPK14	4.91E-03
11	33	89	3	Agr Pathway	1.81E-03	PXN, JUN, MAPK1	1.87E-02
11	30	89	4	At1R Pathway	6.60E-05	SHC1, PTK2B, JUN, MAPK1	3.58E-03
11	21	89	3	Chemical Pathway	4.71E-04	BAX, PXN, BCL2L1	9.06E-03
11	39	89	3	Biopeptides Pathway	2.94E-03	SHC1, PTK2B, MAPK1	2.78E-02
11	22	89	3	Ceramide Pathway	5.43E-04	BAX, TNFRSF1A, MAPK1	9.06E-03
11	22	89	3	Cxcr4 Pathway	5.43E-04	PXN, PTK2B, MAPK1	9.06E-03
11	23	89	3	Ecm Pathway	6.21E-04	SHC1, PXN, MAPK1	9.63E-03
11	30	89	3	Fas Pathway	1.37E-03	CFLAR, FAS, JUN	1.65E-02
11	35	89	3	Fcer1 Pathway	2.15E-03	SHC1, JUN, MAPK1	2.12E-02
11	55	89	3	Hivnef Pathway	7.76E-03	CFLAR, FAS, TNFRSF1A	4.81E-02
11	37	89	6	Il2Rb Pathway	2.66E-07	SHC1, NMI, CFLAR, FAS, BCL2L1, MAPK1	5.77E-05
11	26	89	3	Raccycd Pathway	8.97E-04	IKBKB, MAPK1, CDKN1A	1.15E-02
11	34	89	4	Integrin Pathway	1.09E-04	SHC1, PXN, JUN, MAPK1	3.95E-03
11	45	89	5	Keratinocyte Pathway	1.91E-05	FAS, IKBKB, JUN, TN-FRSF1A, MAPK1	2.07E-03
11	25	89	4	Pyk2 Pathway	3.13E-05	SHC1, PTK2B, JUN, MAPK1	2.27E-03
11	86	89	5	Mapk Pathway	4.28E-04	SHC1, IKBKB, JUN, RPS6KA3, MAPK1	9.06E-03
11	22	89	3	Nfkb Pathway	5.43E-04	IKBKB, TNFRSF1A, MYD88	9.06E-03
11	25	89	3	Bad Pathway	7.98E-04	BAX, BCL2L1, MAPK1	1.08E-02
11	16	89	3	Pml Pathway	2.04E-04	FAS, TNFRSF1A, SP100	5.53E-03
11	31	89	3	Il1R Pathway	1.51E-03	IKBKB, JUN, MYD88	1.72E-02
11	34	89	4	Met Pathway	1.09E-04	PXN, PTK2B, JUN, MAPK1	3.95E-03
11	16	89	3	Spry Pathway	2.04E-04	SHC1, SPRY1, MAPK1	5.53E-03
11	25	89	3	Stress Pathway	7.98E-04	IKBKB, JUN, TNFRSF1A	1.08E-02
11	32	89	3	Toll Pathway	1.66E-03	IKBKB, JUN, MYD88	1.80E-02
16	20	93	4	Atm Pathway	1.47E-05	CHEK1, CHEK2, RAD51, BRCA1	7.97E-04
16	18	93	5	Mcm Pathway	1.95E-07	CDC6, CDT1, MCM4, ORC1L, CDK2	2.12E-05
16	27	93	3	G1 Pathway	1.14E-03	SKP2, CDK2, CDC25A	3.54E-02
16	23	93	6	G2 Pathway	1.64E-08	CHEK1, CHEK2, PLK1, BRCA1, CDC25C, CDC25A	3.55E-06

16	12	93	3	Rb Pathway	9.33E-05	CHEK1, CDC25C, CDK2	3.37E-03
16	20	93	5	Atrbrca Pathway	3.49E-07	CHEK1, CHEK2, RAD51, BRCA2, BRCA1	2.52E-05
16	9	93	3	Ranms Pathway	3.62E-05	AURKA, KIF15, TPX2	1.57E-03
17	14	59	3	Plateletapp Pathway	3.91E-05	PLAU, COL4A2, SERPINE1	8.49E-03
20	10	74	3	Gaba Pathway	2.59E-05	GABRA1, DNM1, GABRA5	5.62E-03
24	55	38	3	Hivnef Pathway	6.79E-04	RIPK1, NFKB1, PRKCD	3.21E-02
24	45	38	3	Keratinocyte Pathway	3.75E-04	RIPK1, NFKB1, PRKCD	3.21E-02
24	86	38	3	Mapk Pathway	2.48E-03	RIPK1, TGFBR1, NFKB1	3.84E-02
24	24	38	4	Nthi Pathway	8.56E-07	TGFBR1, TGFBR2, NFKB1, TLR2	1.86E-04
25	12	75	3	Fibrinolysis Pathway	4.90E-05	PLAU, SERPINE1, F13A1	1.06E-02
32	22	71	4	Mtor Pathway	7.49E-06	PDPK1, EIF3A, RPS6KB1, EIF4G3	1.63E-03
32	22	71	3	Eif4 Pathway	2.79E-04	PDPK1, RPS6KB1, EIF4G3	3.03E-02
40	18	84	6	Mcm Pathway	1.67E-09	CDT1, MCM2, MCM7, MCM4, CDK2, MCM3	3.62E-07
40	23	84	3	G2 Pathway	5.24E-04	CHEK1, BRCA1, PLK1	3.79E-02
40	20	84	4	Atrbrca Pathway	9.80E-06	FANCC, CHEK1, BRCA2, BRCA1	1.06E-03
41	17	68	3	Il10 Pathway	1.11E-04	IL10RA, HMOX1, IL6	2.40E-02
49	13	41	3	Thelper Pathway	1.02E-05	CD4, ITGB2, PTPRC	2.22E-03
51	35	88	4	Fcer1 Pathway	1.17E-04	SYK, FCER1G, LYN, PIK3CG	8.48E-03
51	25	88	3	Bad Pathway	7.72E-04	RPS6KA1, CSF2RB, PIK3CG	4.19E-02
51	13	88	3	Thelper Pathway	1.02E-04	PTPRC, ITGB2, CD4	8.48E-03
51	32	88	4	Toll Pathway	8.19E-05	LY96, TLR2, CD14, TLR7	8.48E-03
