# Supplementary material for: A Computational Approach to Identifying Gene-microRNA Modules in Cancer
Source: PLoS Comput Biol. 2015 Jan 22;11(1):e1004042. doi: 10.1371/journal.pcbi.1004042 (PMC4303261; doi:10.1371/journal.pcbi.1004042)
Supplement: S21 Table — (PDF) [file pcbi.1004042.s028.pdf]

Table S21. Comparisons of enriched pathways from the NMF approach and our approach.

Common pathways
Defense Response, Inflammatory Response, Immune Response, Response To Wounding, Regulation Of Cell Proliferation, Locomotory Behavior, Behavior, Response To Chemical Stimulus, Response To External Stimulus, Cellular Defense Response, Mitotic Cell Cycle Checkpoint, M Phase, Microtubule Based Process, Regulation Of Mitosis, Microtubule Cytoskeleton Organization And Biogenesis, Cell Cycle Process, Mitotic Cell Cycle, Cell Cycle Phase, Chromosome Segregation, Mitotic Spindle Organization And Biogenesis, Mitosis, Cytoskeleton Organization And Biogenesis, Dna Packaging, Spindle Organization And Biogenesis, M Phase Of Mitotic Cell Cycle, Cytokine Cytokine Receptor Interaction, Chemokine Signaling Pathway, Natural Killer Cell Mediated Cytotoxicity, T Cell Receptor Signaling Pathway, B Cell Receptor Signaling Pathway, Fc Epsilon Ri Signaling Pathway, Primary Immunodeficiency, Cell Adhesion Molecules Cams, Antigen Processing And Presentation, Toll Like Receptor Signaling Pathway, Cytosolic Dna Sensing Pathway, Complement And Coagulation Cascades, Tgf Beta Signaling Pathway, Ecm Receptor Interaction

Pathways enriched only in our modules
Bcr Pathway, Mcm Pathway, Hivnef Pathway, Atrbrca Pathway, Il2 Pathway, Ctla4 Pathway, G2 Pathway, Ranms Pathway, Hemopoiesis, Immune System Development, Leukocyte Differentiation, Hemopoietic Or Lymphoid Organ Development, Immune System Process, T Cell Activation, Protein Amino Acid Phosphorylation, Humoral Immune Response, Phosphorylation, Cell Cycle Checkpoint Go 0000075, G1 Phase Of Mitotic Cell Cycle, Dna Replication, G1 Phase, Cell Cycle Go 0007049, Interphase Of Mitotic Cell Cycle, Dna Metabolic Process, Regulation Of Kinase Activity, Regulation Of Protein Kinase Activity, Regulation Of Transferase Activity, Interphase, Regulation Of Cyclin Dependent Protein Kinase Activity, Dna Dependent Dna Replication, Response To Dna Damage Stimulus, Regulation Of Cell Cycle, Dna Repair, Response To Endogenous Stimulus, Dna Integrity Checkpoint, Dna Replication Initiation, Response To Abiotic Stimulus, Dna Recombination, Cellular Cation Homeostasis, Homeostatic Process, Cellular Homeostasis, Chemical Homeostasis, Ion Homeostasis, Apoptotic Program, Cation Homeostasis, Proteolysis, S Phase, Skeletal Development, Jak Stat Cascade, Peptidyl Tyrosine Phosphorylation, Peptidyl Amino Acid Modification, Peptidyl Tyrosine Modification, Cytokinesis, Mitotic Sister Chromatid Segregation, Establishment Of Organelle Localization, Cell Division, Sister Chromatid Segregation, Organelle Localization, Chromosome Organization And Biogenesis, Chromosome Condensation, Fc Gamma R Mediated Phagocytosis, Dna Replication, Cell Cycle, Oocyte Meiosis, P53 Signaling Pathway, Progesterone Mediated Oocyte Maturation, Base Excision Repair, Nucleotide Excision Repair, Mismatch Repair, Focal Adhesion, Regulation Of Actin Cytoskeleton, Rna Degradation, Spliceosome, Proteasome, Lysosome

Pathways enriched only in modules identified from the NMF approach
Fibrinolysis Pathway, Caspase Pathway, Rankl Pathway, Il6 Pathway, Arenrf2 Pathway, Cardiacegf Pathway, Anatomical Structure Formation, Angiogenesis, Vasculature Development, Negative Regulation Of Developmental Process, Acute Inflammatory Response, Negative Regulation Of Cell Proliferation, Response To Xenobiotic Stimulus, Xenobiotic Metabolic Process, Response To Virus, Multi Organism Process, Response To Biotic Stimulus, Response To Other Organism, Translation, Synaptogenesis, Extracellular Structure Organization And Biogenesis, Synaptic Transmission, Synapse Organization And Biogenesis, Transmission Of Nerve Impulse, Phagocytosis, Response To Bacterium, Defense Response To Bacterium, Regulation Of Cellular Ph, Monovalent Inorganic Cation Homeostasis, Regulation Of Ph, Cellular Monovalent Inorganic Cation Homeostasis, Epidermis Development, Ectoderm Development, Nod Like Receptor Signaling Pathway, Hematopoietic Cell Lineage, Leukocyte Transendothelial Migration, Pentose And Glucuronate Interconversions, Ascorbate And Aldarate Metabolism, Endocytosis, Rig I Like Receptor Signaling Pathway, Type I Diabetes Mellitus, Autoimmune Thyroid Disease, Allograft Rejection, Graft Versus Host Disease, Viral Myocarditis, Ppar Signaling Pathway, Ribosome, Intestinal Immune Network For Iga Production, Leishmania Infection, Asthma, Systemic Lupus Erythematosus, O Glycan Biosynthesis, Basal Cell Carcinoma, Hedgehog Signaling Pathway, Neuroactive Ligand Receptor Interaction, Mapk Signaling Pathway,
