# Supplementary material for: A Computational Approach to Identifying Gene-microRNA Modules in Cancer
Source: PLoS Comput Biol. 2015 Jan 22;11(1):e1004042. doi: 10.1371/journal.pcbi.1004042 (PMC4303261; doi:10.1371/journal.pcbi.1004042)
Supplement: S22 Table — (PDF) [file pcbi.1004042.s029.pdf]

Table S22. Comparisons of enriched pathways from the COSMIC algorithm and our approach.

Common pathways
Microtubule Based Process, Microtubule Cytoskeleton Organization And Biogenesis, Cell Cycle Process, Mitotic Cell Cycle, Cell Cycle Phase, Cell Cycle Go 0007049, Defense Response, Inflammatory Response, Immune Response, Immune System Process, Response To External Stimulus, Response To Wounding, Cytokine Cytokine Receptor Interaction, Skeletal Development, Focal Adhesion, Ecm Receptor Interaction, Regulation Of Actin Cytoskeleton, Locomotory Behavior, Natural Killer Cell Mediated Cytotoxicity, T Cell Receptor Signaling Pathway, Antigen Processing And Presentation, Chemokine Signaling Pathway, Dna Replication, Nucleotide Excision Repair, Mismatch Repair, G2 Pathway

Pathways enriched only in our modules
Bcr Pathway, Mcm Pathway, Hivnef Pathway, Atrbrca Pathway, Il2 Pathway, Ctla4 Pathway, Ranms Pathway, Hemopoiesis, Immune System Development, Behavior, Leukocyte Differentiation, Response To Chemical Stimulus, Hemopoietic Or Lymphoid Organ Development, T Cell Activation, Protein Amino Acid Phosphorylation, Humoral Immune Response, Phosphorylation, Cellular Defense Response, Cell Cycle Checkpoint Go 0000075, G1 Phase Of Mitotic Cell Cycle, Dna Replication, G1 Phase, Interphase Of Mitotic Cell Cycle, Dna Metabolic Process, Regulation Of Kinase Activity, Regulation Of Protein Kinase Activity, M Phase, Regulation Of Transferase Activity, Interphase, Regulation Of Cyclin Dependent Protein Kinase Activity, Mitosis, Dna Dependent Dna Replication, Response To Dna Damage Stimulus, Regulation Of Cell Cycle, M Phase Of Mitotic Cell Cycle, Regulation Of Cell Proliferation, Dna Repair, Response To Endogenous Stimulus, Dna Integrity Checkpoint, Dna Replication Initiation, Response To Abiotic Stimulus, Dna Recombination, Mitotic Cell Cycle Checkpoint, Regulation Of Mitosis, Chromosome Segregation, Cellular Cation Homeostasis, Homeostatic Process, Cellular Homeostasis, Chemical Homeostasis, Ion Homeostasis, Apoptotic Program, Cation Homeostasis, Proteolysis, S Phase, Cytoskeleton Organization And Biogenesis, Jak Stat Cascade, Peptidyl Tyrosine Phosphorylation, Peptidyl Amino Acid Modification, Peptidyl Tyrosine Modification, Cytokinesis, Mitotic Sister Chromatid Segregation, Establishment Of Organelle Localization, Cell Division, Mitotic Spindle Organization And Biogenesis, Sister Chromatid Segregation, Organelle Localization, Chromosome Organization And Biogenesis, Dna Packaging, Spindle Organization And Biogenesis, Chromosome Condensation, Cell Adhesion Molecules Cams, Toll Like Receptor Signaling Pathway, Cytosolic Dna Sensing Pathway, B Cell Receptor Signaling Pathway, Fc Epsilon Ri Signaling Pathway, Fc Gamma R Mediated Phagocytosis, Primary Immunodeficiency, Cell Cycle, Oocyte Meiosis, P53 Signaling Pathway, Progesterone Mediated Oocyte Maturation, Base Excision Repair, Tgf Beta Signaling Pathway, Complement And Coagulation Cascades, Rna Degradation, Spliceosome, Proteasome, Lysosome

Pathways enriched only in modules identified from the COSMIC algorithm
Mrna Metabolic Process, Response To Virus, Multi Organism Process, Regulation Of T Cell Activation, Leukocyte Activation, Response To Biotic Stimulus, Response To Other Organism, Regulation Of Cell Cell Adhesion, Lymphocyte Activation, Regulation Of Multicellular Organismal Process, Calcium Signaling Pathway, Dilated Cardiomyopathy, Mrna Processing Go 0006397, Mcalpain Pathway, Rna Processing, Tob1 Pathway, Muscle Cell Differentiation, Enzyme Linked Receptor Protein Signaling Pathway, Transforming Growth Factor Beta Receptor Signaling Pathway, Transmembrane Receptor Protein Serine Threonine Kinase Signaling Pathway, Nod Like Receptor Signaling Pathway, Hedgehog Signaling Pathway, Basal Cell Carcinoma, Csk Pathway, Tcra Pathway, Leukocyte Transendothelial Migration, Glycerolipid Metabolism, Ppar Signaling Pathway, Small Cell Lung Cancer, Rig I Like Receptor Signaling Pathway, Death Pathway, Ami Pathway, Ace2 Pathway, Intrinsic Pathway, Plateletapp Pathway, Vitcb Pathway, Granulocytes Pathway, Monocyte Pathway
