# Supplementary material for: A Computational Approach to Identifying Gene-microRNA Modules in Cancer
Source: PLoS Comput Biol. 2015 Jan 22;11(1):e1004042. doi: 10.1371/journal.pcbi.1004042 (PMC4303261; doi:10.1371/journal.pcbi.1004042)
Supplement: S23 Table — (PDF) [file pcbi.1004042.s030.pdf]

Table S23. Ovarian cancer modules enriched with GO terms using a TANGO tool.

Module ID	x	Description	p-value	q-value	Frequency	Genes
2	13	Leukocyte Activation	6.94E-10	0.001	0.22	PSMB10, MEF2C, LYN, PTPN22, VAV1, CD74, BTK, DOCK2, LCK, CD4, BLNK, LCP2, TYROBP
2	11	Positive Regulation Of Leukocyte Activation	3.75E-09	0.001	0.19	MEF2C, LYN, FGR, IL18, ICOS, LCK, CD4, VAV1, CD27, CD74, BTK
2	10	Immune Response-Regulating Signaling Pathway	3.11E-08	0.002	0.17	MEF2C, LYN, FGR, HCK, LCK, PTPN22, CD4, CTSH, LCP2, BTK
2	11	Response To Bacterium	9.03E-08	0.002	0.19	MEF2C, LYN, FGR, CCR5, CXCL13, HCK, IL18, MMP9, ACP5, CD4, CXCL10
2	10	Regulation Of Cell Adhesion	1.70E-07	0.005	0.17	LPXN, LYN, CD44, CCR5, CXCL13, IL18, CYTH4, VAV1, ARHGDIB, SPP1
2	9	Leukocyte Differentiation	3.14E-07	0.01	0.15	MEF2C, DOCK2, LYN, MMP9, LCK, PTPN22, CD4, CD74, BLNK
2	7	Adaptive Immune Response	5.67E-07	0.014	0.12	MEF2C, CXCL13, IL18, CTSH, CD27, CD74, BTK
2	5	Regulation Of Leukocyte Apoptotic Process	1.36E-06	0.025	0.085	MEF2C, LYN, CCR5, CD74, BTK
2	8	Regulation Of Immune Effector Process	2.59E-06	0.042	0.14	DOCK2, LYN, FGR, HCK, LCK, CD4, CD74, BTK
3	17	Interphase Of Mitotic Cell Cycle	1.26E-18	0.001	0.49	E2F1, GINS1, CDC6, GINS2, POLA2, MCM2, MCM10, MCM4, CDK2, CEP70, CCNE2, CDC45, CCNB2, MCM7, PLK1, TFDP2, TUBG1
3	12	Cell Cycle Checkpoint	3.08E-13	0.001	0.34	E2F1, CCNE2, CDC6, CDC45, MCM7, CCNB2, PLK1, BUB1, MCM2, MCM10, MCM4, CDK2
3	7	Dna Strand Elongation Involved In Dna Replication	2.83E-12	0.001	0.2	GINS1, GINS2, CDC45, MCM7, MCM2, POLA2, MCM4
3	10	Dna Replication	2.75E-11	0.001	0.29	GINS1, GINS2, CDC6, CDC45, MCM7, MCM2, POLA2, MCM10, MCM4, CDK2
3	8	M/G1 Transition Of Mitotic Cell Cycle	3.51E-11	0.001	0.23	CDC6, CDC45, MCM7, MCM2, POLA2, MCM10, MCM4, CDK2
3	5	Dna-Dependent Dna Replication Initiation	2.65E-09	0.001	0.14	CDC45, MCM7, MCM2, POLA2, MCM4
3	8	Mitosis	4.30E-07	0.013	0.23	KIF22, CDC6, CCNB2, PLK1, BUB1, CEP55, ASPM, CDK2
4	5	Cilium Morphogenesis	2.93E-06	0.049	0.1	TMEM231, FOXJ1, DNAAF1, TCTN1, DNAI2
6	12	Dna Replication	1.47E-14	0.001	0.35	CDC7, PRIM1, RFC5, POLE2, PCNA, CHEK1, MCM2, MCM4, CDK2, CDC25A, FEN1, MCM6
6	14	Interphase Of Mitotic Cell Cycle	2.66E-14	0.001	0.41	CDC7, PRIM1, RFC5, CCNE2, POLE2, PCNA, CHEK1, MCM2, CDKN3, MCM4, CDK2, CDC25A, FEN1, MCM6
6	11	G1/S Transition Of Mitotic Cell Cycle	1.71E-13	0.001	0.32	CDC7, PRIM1, CCNE2, POLE2, PCNA, MCM2, CDKN3, MCM4, CDK2, CDC25A, MCM6
6	10	S Phase Of Mitotic Cell Cycle	2.62E-13	0.001	0.29	PRIM1, RFC5, POLE2, PCNA, MCM2, MCM4, CDK2, CDC25A, FEN1, MCM6
6	7	Dna Strand Elongation Involved In Dna Replication	2.28E-12	0.001	0.21	PRIM1, RFC5, PCNA, MCM2, MCM4, FEN1, MCM6
6	11	Cell Cycle Checkpoint	6.43E-12	0.001	0.32	CDC7, RFC5, CCNE2, KNTC1, BUB1, CHEK1, MCM2, MCM4, CDK2, CDC25A, MCM6

6	10	Mitosis	1.04E-09	0.001	0.29	KIF23, TIMELESS, DSN1, KIF15, KNTC1, BUB1, CEP55, RACGAP1, CDK2, CDC25A
6	5	Dna-Dependent Dna Replication Initiation	2.29E-09	0.001	0.15	PRIM1, POLE2, MCM2, MCM4, MCM6
6	5	Telomere Maintenance Via Semi-Conservative Replication	2.99E-09	0.001	0.15	PRIM1, RFC5, POLE2, PCNA, FEN1
6	9	Dna Repair	9.72E-08	0.002	0.26	RFC5, MSH6, POLE2, PCNA, CHEK1, PARP2, CDK2, FEN1, BARD1
8	7	Regulation Of Cell-Substrate Adhesion	1.22E-08	0.001	0.18	CCL21, LGALS1, SERPINE1, COL1A1, THBS1, ITGB1, PLAU
8	9	Blood Vessel Morphogenesis	2.99E-07	0.009	0.23	ITGA5, SERPINE1, MMP19, PDGFRB, THBS1, ITGB1, PLAU, MYLK, FN1
8	4	Peptide Cross-Linking	1.05E-06	0.02	0.1	BGN, COL3A1, THBS1, FN1
8	5	Response To Amino Acid Stimulus	1.88E-06	0.031	0.13	COL3A1, COL1A2, COL1A1, COL16A1, TIMP3
8	7	Platelet Activation	2.47E-06	0.041	0.18	SERPINE1, COL3A1, COL1A2, TUBA4A, COL1A1, THBS1, FN1
8	4	Collagen Metabolic Process	2.68E-06	0.042	0.1	MMP19, COL3A1, COL1A1, MMP11
12	10	Interphase Of Mitotic Cell Cycle	4.68E-09	0.001	0.3	PRIM1, RFC5, E2F3, NBN, PSMA5, GMNN, TFDP2, MCM2, MCM10, FEN1
12	4	Dna Strand Elongation Involved In Dna Replication	1.54E-06	0.028	0.12	PRIM1, RFC5, MCM2, FEN1
13	21	Interphase Of Mitotic Cell Cycle	1.41E-16	0.001	0.27	CDC6, GINS2, DBF4, GMNN, CHEK1, BIRC5, MCM10, MCM6, CCNE2, PSMB4, EIF4EBP1, CDC45, MCM7, POLE2, PLK1, TFDP2, PCNA, ORC5, ORC1, MELK, ORC2
13	18	Cell Cycle Checkpoint	6.03E-16	0.001	0.23	CDC6, DBF4, CHEK1, BIRC5, MCM10, MCM6, RAD1, CCNE2, PSMB4, CDC45, MCM7, PLK1, BUB1, ORC5, FANCG, ORC1, BUB3, ORC2
13	16	Dna Replication	6.02E-15	0.001	0.21	POLL, CDC6, GINS2, DBF4, CHEK1, MCM10, MCM6, RAD1, TFAM, CDC45, MCM7, POLE2, PCNA, ORC5, ORC1, ORC2
13	12	M/G1 Transition Of Mitotic Cell Cycle	4.19E-14	0.001	0.16	PSMB4, CDC6, CDC45, MCM7, POLE2, GMNN, DBF4, ORC5, MCM10, ORC1, ORC2, MCM6
13	14	G1/S Transition Of Mitotic Cell Cycle	4.81E-13	0.001	0.18	CDC6, DBF4, MCM10, MCM6, CCNE2, PSMB4, EIF4EBP1, CDC45, MCM7, POLE2, PCNA, ORC5, ORC1, ORC2
13	10	Dna-Dependent Dna Replication	1.49E-11	0.001	0.13	GINS2, TFAM, CDC45, MCM7, POLE2, PCNA, ORC5, ORC1, ORC2, MCM6
13	7	Dna-Dependent Dna Replication Initiation	2.89E-11	0.001	0.091	CDC45, MCM7, POLE2, ORC5, ORC1, ORC2, MCM6
13	6	Regulation Of Chromosome Segregation	1.15E-09	0.001	0.078	CDC42, CDC6, BUB1, AURKB, ECT2, BUB3
13	13	Mitosis	5.27E-09	0.001	0.17	CDC6, HAUS6, NCAPH, CDCA8, TIMELESS, PLK1, DSN1, BUB1, BIRC5, AURKB, SMC2, BUB3, SMC4
13	8	Regulation Of Nuclear Division	1.98E-08	0.002	0.1	CDC42, CDC6, AURKAIP1, PLK1, BUB1, RANBP1, CHEK1, BUB3
13	12	Regulation Of Mitotic Cell Cycle	1.12E-07	0.002	0.16	CDC42, PSMB4, CDC6, CDC45, EIF4EBP1, AURKAIP1, PLK1, BUB1, RANBP1, BIRC5, CHEK1, BUB3
13	4	Regulation Of Cell Cycle Cytokinesis	1.16E-06	0.021	0.052	CDC42, CDC6, AURKB, ECT2

13	11	Dna Repair	2.68E-06	0.042	0.14	EXO1, RAD1, POLL, POLE2, UCHL5, PCNA, FANCE, CHEK1, FANCG, SMC2, SMC4
18	12	Regulation Of Cell Cycle Arrest	1.72E-13	0.001	0.39	CDC7, RFC5, CCNE2, MCM7, FOXM1, KNTC1, CHEK1, MCM2, FANCG, MCM3, MCM4, MCM6
18	11	Dna Replication	1.80E-13	0.001	0.35	CDC7, RFC5, MCM7, NASP, CHEK1, MCM2, MCM3, MCM4, FEN1, MCM6, SLBP
18	13	Interphase	2.05E-13	0.001	0.42	CDC7, RFC5, CCNE2, MCM7, FOXM1, CHEK1, MCM2, MCM3, MCM4, FEN1, MELK, MCM6, SLBP
18	7	Dna Strand Elongation Involved In Dna Replication	1.12E-12	0.001	0.23	RFC5, MCM7, MCM2, MCM3, MCM4, FEN1, MCM6
18	8	Dna Repair	6.37E-07	0.016	0.26	EXO1, RFC5, FOXM1, CHEK1, TOPBP1, FANCG, FEN1, SMC4
18	4	Regulation Of S Phase	6.66E-07	0.016	0.13	CDC7, TIMELESS, CHEK1, SLBP
18	7	Mitosis	2.52E-06	0.042	0.23	KIF23, TIMELESS, DSN1, KNTC1, AURKB, RACGAP1, SMC4
20	26	Mitosis	3.82E-33	0.001	0.59	KIF23, NEK2, AURKA, CEP55, AURKB, MYBL2, SPC25, NCAPH, CDCA8, CENPA, BUB1, CCNA2, ASPM, CDCA3, ERCC6L, KIF15, CENPE, BIRC5, CDC20, RACGAP1, CCNB1, MAD2L1, CCNB2, SPAG5, PLK1, BUB1B
20	13	Mitotic Prometaphase	2.13E-19	0.001	0.3	CCNB1, SPC25, CDCA8, MAD2L1, PLK1, CENPA, BUB1, BUB1B, CENPE, BIRC5, CDC20, AURKB, ERCC6L
20	17	Microtubule-Based Process	2.22E-17	0.001	0.39	KIF14, KIF23, KIF4A, NEK2, KIF15, CENPE, AURKA, AURKB, RACGAP1, MYBL2, GTSE1, SPC25, PLK4, PLK1, CENPA, SPAG5, BUB1B
20	11	Chromosome Segregation	5.43E-14	0.001	0.25	CCNB1, SPC25, NCAPH, MAD2L1, NEK2, SPAG5, BUB1, CENPE, BIRC5, AURKB, TOP2A
20	9	Spindle Organization	1.28E-12	0.001	0.2	KIF23, SPC25, NEK2, SPAG5, BUB1B, AURKA, AURKB, RACGAP1, MYBL2
20	10	G2/M Transition Of Mitotic Cell Cycle	1.47E-11	0.001	0.23	CCNB1, PLK4, CCNB2, PLK1, NEK2, FOXM1, BIRC5, TOP2A, CCNA2, MELK
20	12	Regulation Of Cell Cycle Arrest	2.03E-11	0.001	0.27	CCNB1, MAD2L1, CCNB2, PLK1, FOXM1, BUB1, BUB1B, BIRC5, CDC20, TOP2A, CCNA2, GTSE1
20	6	Regulation Of Chromosome Segregation	3.56E-11	0.001	0.14	CCNB1, NEK2, SPAG5, BUB1, AURKB, RACGAP1
20	12	Regulation Of Mitotic Cell Cycle	1.32E-10	0.001	0.27	CCNB1, MAD2L1, PLK1, NEK2, BUB1, BUB1B, AURKA, BIRC5, CDC20, TOP2A, CCNA2, GTSE1
20	6	Spindle Checkpoint	7.04E-10	0.001	0.14	CCNB1, MAD2L1, BUB1, BUB1B, BIRC5, CDC20
20	7	Organelle Assembly	7.72E-09	0.001	0.16	KIF23, NEK2, CENPA, CENPE, AURKB, RACGAP1, MYBL2
20	7	Anaphase-Promoting Complex-Dependent Proteasomal Ubiquitin-Dependent Protein Catabolic Process	7.72E-09	0.001	0.16	CCNB1, MAD2L1, PLK1, BUB1B, AURKA, CDC20, AURKB
20	7	Regulation Of Nuclear Division	7.72E-09	0.001	0.16	MAD2L1, PLK1, NEK2, BUB1, BUB1B, AURKA, CDC20

20	8	Positive Regulation Of Cell Cycle Process	4.55E-08	0.002	0.18	KIF23, PLK4, PLK1, AURKA, BIRC5, AURKB, RACGAP1, GTSE1
20	6	Regulation Of Microtubule Cytoskeleton Organization	1.50E-07	0.005	0.14	CCNB1, PLK4, NEK2, SPAG5, AURKA, RACGAP1
20	4	Protein Localization To Chromosome	6.47E-07	0.016	0.091	PLK1, CENPA, BUB1B, AURKB
21	7	Activation Of Immune Response	8.41E-07	0.02	0.23	C1QA, ITK, HCK, LCK, MNDA, LCP2, BTK
22	10	Extracellular Matrix Organization	2.27E-13	0.001	0.37	COL3A1, COL1A1, GREM1, LOXL2, COL5A3, MMP2, COL5A1, MMP1, MMP11, DPT
22	7	Collagen Metabolic Process	2.91E-13	0.001	0.26	MMP19, COL3A1, COL1A1, MMP2, COL5A1, MMP1, MMP11
22	7	Collagen Fibril Organization	3.79E-13	0.001	0.26	COL3A1, COL1A1, GREM1, LOXL2, COL5A3, COL5A1, DPT
22	8	Angiogenesis	2.24E-08	0.002	0.3	ITGA5, MMP19, GREM1, LOXL2, ECM1, MMP2, PLAU, FN1
22	4	Cellular Response To Amino Acid Stimulus	8.64E-07	0.02	0.15	COL3A1, COL6A1, COL1A1, MMP2
22	4	Skin Development	2.83E-06	0.045	0.15	COL3A1, COL1A1, COL5A3, COL5A1
25	15	Interphase Of Mitotic Cell Cycle	4.14E-17	0.001	0.52	CDC7, CHEK1, MCM2, MCM10, MCM4, MCM6, CCNE2, CCNB2, MCM7, POLE2, PCNA, FBXO5, KPNA2, FEN1, MELK
25	11	Dna Replication	7.52E-14	0.001	0.38	CDC7, MCM7, POLE2, PCNA, TIPIN, CHEK1, MCM2, MCM10, MCM4, FEN1, MCM6
25	8	Dna-Dependent Dna Replication	2.91E-12	0.001	0.28	MCM7, POLE2, PCNA, TIPIN, MCM2, MCM4, FEN1, MCM6
25	11	Mitosis	6.26E-12	0.001	0.38	KIF23, CCNB2, TIMELESS, DSN1, KIF15, TIPIN, FBXO5, NDC80, RACGAP1, MYBL2, SMC4
25	10	Cell Cycle Checkpoint	2.90E-11	0.001	0.34	CDC7, CCNE2, MCM7, CCNB2, TIPIN, CHEK1, MCM2, MCM10, MCM4, MCM6
25	7	M/G1 Transition Of Mitotic Cell Cycle	4.20E-10	0.001	0.24	CDC7, MCM7, POLE2, MCM2, MCM10, MCM4, MCM6
25	5	Dna-Dependent Dna Replication Initiation	9.79E-10	0.001	0.17	MCM7, POLE2, MCM2, MCM4, MCM6
25	4	Dna Unwinding Involved In Replication	2.12E-09	0.001	0.14	MCM7, MCM2, MCM4, MCM6
25	5	Mitotic Spindle Organization	5.01E-09	0.001	0.17	KIF23, PRC1, NDC80, RACGAP1, MYBL2
25	9	Microtubule-Based Process	8.30E-09	0.001	0.31	KIF23, KIF4A, PRC1, KIF15, FBXO5, NDC80, RACGAP1, MYBL2, KPNA2
25	4	Regulation Of S Phase	5.07E-07	0.013	0.14	CDC7, TIMELESS, TIPIN, CHEK1
25	4	Spindle Assembly	1.02E-06	0.02	0.14	KIF23, FBXO5, RACGAP1, MYBL2
26	19	Interphase Of Mitotic Cell Cycle	1.88E-19	0.001	0.43	CDC7, GINS2, GMNN, CHEK1, BIRC5, PSMA7, MCM10, MCM4, RPA3, CCNB1, RFC5, PLK4, RFC4, PLK1, PCNA, FBXO5, CCNA2, KPNA2, FEN1
26	13	Cell Cycle Checkpoint	2.78E-13	0.001	0.3	CDC7, CHEK1, BIRC5, PSMA7, MCM10, MCM4, RPA3, RFC5, CCNB1, MAD2L1, RFC4, PLK1, CCNA2
26	13	Mitosis	2.89E-12	0.001	0.3	CENPN, DSN1, RAN, DYNLT1, BIRC5, CCNB1, SPC25, MAD2L1, TIMELESS, CENPA, PLK1, FBXO5, CCNA2
26	7	Dna Strand Elongation Involved In Dna Replication	1.59E-11	0.001	0.16	RFC5, GINS2, RFC4, PCNA, MCM4, FEN1, RPA3

26	10	Dna Replication	3.34E-10	0.001	0.23	CDC7, RFC5, GINS2, RFC4, PCNA, CHEK1, MCM10, MCM4, FEN1, RPA3
26	8	Microtubule Cytoskeleton Organization	2.81E-07	0.009	0.18	SPC25, PLK4, RAN, PLK1, CENPA, FBXO5, RANBP1, DYNLT1
26	9	Regulation Of Mitotic Cell Cycle	4.10E-07	0.013	0.2	CCNB1, MAD2L1, PLK1, FBXO5, RANBP1, BIRC5, CHEK1, PSMA7, CCNA2
26	7	Dna Recombination	7.29E-07	0.017	0.16	RFC5, XRCC4, RFC4, UCHL5, PCNA, FEN1, RPA3
26	6	Regulation Of Ligase Activity	8.29E-07	0.019	0.14	CCNB1, XRCC4, MAD2L1, PLK1, FBXO5, PSMA7
27	9	Collagen Fibril Organization	1.05E-14	0.001	0.16	COL3A1, COL1A2, LOX, COL1A1, GREM1, LOXL2, COL11A1, COL5A2, COL5A1
27	13	Extracellular Matrix Organization	3.68E-14	0.001	0.24	COL3A1, COL1A2, LOX, COL1A1, MMP14, GREM1, LOXL2, ECM2, COL11A1, COL5A2, MMP2, COL5A1, MMP11
27	7	Cellular Response To Amino Acid Stimulus	1.31E-10	0.001	0.13	COL4A1, COL3A1, COL1A2, COL1A1, COL16A1, COL5A2, MMP2
27	12	Angiogenesis	3.07E-10	0.001	0.22	COL4A1, SRPX2, MMP19, PDGFRB, MMP14, THBS1, GREM1, LOXL2, ECM1, MMP2, PLAU, FN1
27	6	Collagen Metabolic Process	4.38E-09	0.001	0.11	MMP19, COL3A1, COL1A1, MMP2, COL5A1, MMP11
27	5	Peptide Cross-Linking	7.38E-08	0.002	0.091	BGN, COL3A1, DCN, THBS1, FN1
27	8	Ossification	5.07E-07	0.013	0.15	CTSK, SPARC, COL1A1, MMP14, COL11A1, ECM1, MMP2, TWIST1
27	5	Skin Development	1.76E-06	0.031	0.091	COL3A1, COL1A2, COL1A1, COL5A2, COL5A1
31	11	Response To Type I Interferon	8.04E-15	0.001	0.17	IRF9, USP18, OASL, SP100, ISG15, OAS3, IRF1, IRF2, OAS1, OAS2, STAT1
31	11	Cellular Response To Interferon-Gamma	1.22E-13	0.001	0.17	IRF9, OASL, SP100, OAS3, IRF1, IRF2, OAS1, OAS2, STAT1, CCL5, GBP1
31	16	Cytokine-Mediated Signaling Pathway	1.73E-12	0.001	0.25	SP100, OAS3, HERC5, OAS1, OAS2, STAT1, CCL5, STAT3, IRF9, USP18, OASL, ISG15, IRF1, PYCARD, IRF2, GBP1
31	13	Response To Virus	6.69E-12	0.001	0.2	OAS3, HERC5, CXCL9, OAS1, OAS2, STAT1, CCL5, TRIM22, CXCL10, IRF9, OASL, ISG15, IRF1
31	6	Negative Regulation Of Reproductive Process	1.64E-07	0.005	0.094	OASL, SP100, SERPINF1, OAS3, OAS1, CCL5
31	9	Immune Effector Process	5.85E-07	0.015	0.14	C1QA, LYN, OAS3, IRF1, CXCL9, OAS1, S100A13, CXCL10, RAB27A
31	4	Adenylyltransferase Activity	1.41E-06	0.027	0.063	OASL, OAS3, OAS1, OAS2
33	34	M Phase Of Mitotic Cell Cycle	2.04E-43	0.001	0.61	KIF23, KIFC1, AURKA, AURKB, CEP55, SPC25, NCAPH, CDCA8, CENPA, OIP5, NCAPG, BUB1, FBXO5, CCNA2, ASPM, KIF11, DLGAP5, KIF15, TPX2, CENPF, NUSAP1, CDC20, ESPL1, NDC80, BIRC5, PBK, RACGAP1, UBE2C, SMC4, CCNB2, PLK1, SPAG5, BUB1B, KIF20A
33	26	Microtubule-Based Process	1.45E-28	0.001	0.46	KIF14, KIF23, KIFC1, KIF4A, KIF11, PRC1, DLGAP5, KIF15, TTK, NUSAP1, NDC80, AURKA, ESPL1, AURKB, UBE2C, TACC3, RACGAP1, SPC25, CENPA, SPAG5, PLK1, CKS2, FBXO5, BUB1B, KPNA2, KIF20A

33	16	Spindle Organization	2.25E-24	0.001	0.29	KIF23, KIF11, PRC1, TTK, NDC80, ESPL1, AURKA, AURKB, TACC3, UBE2C, RACGAP1, SPC25, SPAG5, CKS2, BUB1B, FBXO5
33	15	Chromosome Segregation	3.29E-19	0.001	0.27	KIFC1, DLGAP5, NUSAP1, CENPF, BIRC5, NDC80, ESPL1, AURKB, SMC4, SPC25, NCAPH, SPAG5, NCAPG, BUB1, TOP2A
33	10	Regulation Of Mitotic Metaphase/Anaphase Transition	7.52E-17	0.001	0.18	PLK1, DLGAP5, BUB1, FBXO5, TTK, CENPF, BUB1B, ESPL1, CDC20, UBE2C
33	12	Regulation Of Nuclear Division	5.04E-16	0.001	0.21	PLK1, DLGAP5, BUB1, FBXO5, TTK, CENPF, BUB1B, NUSAP1, ESPL1, AURKA, CDC20, UBE2C
33	10	Sister Chromatid Segregation	3.54E-15	0.001	0.18	KIFC1, NCAPH, SPAG5, NCAPG, DLGAP5, NUSAP1, ESPL1, NDC80, TOP2A, SMC4
33	11	Cytokinesis	7.74E-15	0.001	0.2	KIF23, PRC1, PLK1, NUSAP1, ESPL1, BIRC5, CEP55, AURKB, RACGAP1, ECT2, KIF20A
33	16	Regulation Of Mitotic Cell Cycle	4.53E-14	0.001	0.29	DLGAP5, TPX2, TTK, NUSAP1, CENPF, BIRC5, ESPL1, AURKA, CDC20, UBE2C, PLK1, BUB1, BUB1B, FBXO5, CCNA2, TOP2A
33	13	Regulation Of Cell Cycle Arrest	2.68E-11	0.001	0.23	FOXM1, TTK, CENPF, CDC20, BIRC5, MCM10, UBE2C, CCNB2, PLK1, BUB1, BUB1B, CCNA2, TOP2A
33	7	Spindle Checkpoint	4.26E-11	0.001	0.13	BUB1, TTK, CENPF, BUB1B, BIRC5, CDC20, UBE2C
33	11	Positive Regulation Of Cell Cycle Process	5.12E-11	0.001	0.2	KIF23, PLK1, DLGAP5, NUSAP1, ESPL1, AURKA, BIRC5, AURKB, RACGAP1, UBE2C, ECT2
33	6	Regulation Of Chromosome Segregation	1.61E-10	0.001	0.11	SPAG5, BUB1, ESPL1, AURKB, RACGAP1, ECT2
33	9	Organelle Localization	9.74E-09	0.001	0.16	CENPA, DLGAP5, CENPF, NUSAP1, ESPL1, NDC80, BIRC5, TACC3, ASPM
33	12	Interphase Of Mitotic Cell Cycle	1.01E-08	0.001	0.21	CCNB2, PLK1, FOXM1, RRM2, FBXO5, CENPF, BIRC5, MCM10, TOP2A, KPNA2, CCNA2, MELK
33	8	Meiosis	1.39E-08	0.001	0.14	EXO1, MKI67, PLK1, CKS2, FBXO5, ESPL1, TOP2A, RAD54L
33	7	Anaphase-Promoting Complex-Dependent Proteasomal Ubiquitin-Dependent Protein Catabolic Process	4.39E-08	0.002	0.13	PLK1, FBXO5, BUB1B, AURKA, CDC20, AURKB, UBE2C
33	4	Mitotic Spindle	5.85E-08	0.002	0.071	KIF23, SPAG5, RACGAP1, ECT2
33	7	Inositol Lipid-Mediated Signaling	6.39E-08	0.002	0.13	SPAG5, CKS2, BUB1B, AURKA, NDC80, UBE2C, TOP2A
33	4	Regulation Of Cell Cycle Cytokinesis	3.24E-07	0.01	0.071	KIF23, AURKB, RACGAP1, ECT2
33	7	Dna Packaging	5.14E-07	0.013	0.13	NCAPH, NCAPG, CENPA, OIP5, NUSAP1, TOP2A, SMC4
33	4	Establishment Of Mitotic Spindle Localization	6.14E-07	0.015	0.071	CENPA, NUSAP1, ESPL1, NDC80
33	4	Protein Localization To Chromosome	1.72E-06	0.029	0.071	PLK1, CENPA, BUB1B, AURKB
33	6	Regulation Of Microtubule-Based Process	1.73E-06	0.03	0.11	SPAG5, TPX2, AURKA, RACGAP1, TACC3, ECT2
