# Supplementary material for: A Computational Approach to Identifying Gene-microRNA Modules in Cancer
Source: PLoS Comput Biol. 2015 Jan 22;11(1):e1004042. doi: 10.1371/journal.pcbi.1004042 (PMC4303261; doi:10.1371/journal.pcbi.1004042)
Supplement: S24 Table — (PDF) [file pcbi.1004042.s031.pdf]

**Table S24. GBM modules enriched with GO terms using a TANGO tool.**

| Module ID | x | Description | p-value | q-value | Frequency | Genes |
|---|---|---|---|---|---|---|
| 2 | 40 | M Phase Of Mitotic Cell Cycle | 1.91E-42 | 0.001 | 42 | KIF23, NEK2, KNTC1, AURKA, AURKB, CEP55, MYBL2, KIF2C, SPC25, NCAPH, CDCA8, DDX11, NCAPG, BUB1, FBXO5, ZWILCH, CCNA2, BUB3, ASPM, CDCA3, CDC6, CENPM, KIF11, KIF15, TPX2, CENPF, CDC20, CENPE, ESPL1, NDC80, BIRC5, CDC25C, RACGAP1, SMC2, MAD2L1, SPAG5, PLK1, ZWINT, BUB1B, KIF20A |
| 2 | 30 | Regulation Of Cell Cycle Arrest | 5.51E-29 | 0.001 | 31 | FOXM1, KNTC1, TTK, CHEK1, MCM10, GTSE1, CDT1, CDC45, MCM7, BUB1, ORC6, ZWILCH, CCNA2, TOP2A, BUB3, CDC7, CDC6, CENPF, CDC20, BIRC5, MCM2, CDC25C, MCM3, MCM4, BRCA1, MCM6, MAD2L1, PLK1, ZWINT, BUB1B |
| 2 | 29 | Microtubule-Based Process | 1.88E-25 | 0.001 | 30 | KIF23, KIF4A, PRC1, NEK2, TTK, AURKA, AURKB, MYBL2, GTSE1, KIF2C, SPC25, FBXO5, TUBG1, BUB3, KIF14, KIF11, KIF15, ESPL1, NDC80, CENPE, RACGAP1, TACC3, BRCA1, PLK1, SPAG5, ZWINT, BUB1B, KPNA2, KIF20A |
| 2 | 21 | Chromosome Segregation | 1.17E-24 | 0.001 | 22 | NEK2, MSH5, CENPF, ESPL1, BIRC5, NDC80, CENPE, AURKB, SMC2, BRCA1, KIF2C, SPC25, NCAPH, MAD2L1, DDX11, SPAG5, NCAPG, ZWINT, BUB1, TOP2A, BUB3 |
| 2 | 18 | Spindle Organization | 8.24E-24 | 0.001 | 19 | KIF23, KIF11, PRC1, NEK2, TTK, NDC80, AURKA, ESPL1, AURKB, TACC3, RACGAP1, MYBL2, SPC25, SPAG5, ZWINT, BUB1B, FBXO5, TUBG1 |
| 2 | 18 | Mitotic Prometaphase | 9.43E-23 | 0.001 | 19 | CENPM, KNTC1, CENPF, BIRC5, NDC80, CENPE, CDC20, AURKB, SPC25, KIF2C, CDCA8, MAD2L1, PLK1, ZWINT, BUB1, BUB1B, ZWILCH, BUB3 |
| 2 | 27 | Interphase Of Mitotic Cell Cycle | 1.67E-21 | 0.001 | 28 | NEK2, FOXM1, CHEK1, POLA2, MCM10, GTSE1, CDT1, CDC45, MCM7, DDX11, FBXO5, ORC6, TUBG1, CCNA2, TOP2A, CDC7, CDC6, CENPF, BIRC5, MCM2, CDC25C, MCM3, MCM4, MCM6, PLK1, KPNA2, MELK |
| 2 | 25 | Regulation Of Mitotic Cell Cycle | 2.37E-20 | 0.001 | 26 | CDC6, NEK2, KNTC1, TPX2, CENPF, TTK, CDC20, CHEK1, BIRC5, ESPL1, AURKA, CDC25C, GTSE1, CDT1, CDC45, MAD2L1, PLK1, ZWINT, BUB1, FBXO5, BUB1B, ZWILCH, TOP2A, CCNA2, BUB3 |
| 2 | 13 | Sister Chromatid Segregation | 5.94E-18 | 0.001 | 14 | NEK2, ESPL1, NDC80, CENPE, SMC2, NCAPH, MAD2L1, DDX11, NCAPG, SPAG5, ZWINT, TOP2A, BUB3 |
| 2 | 15 | Regulation Of Nuclear Division | 1.08E-17 | 0.001 | 16 | CDC6, NEK2, CENPF, TTK, ESPL1, AURKA, CDC20, CHEK1, CDC25C, MAD2L1, PLK1, BUB1, BUB1B, FBXO5, BUB3 |
| 2 | 10 | Regulation Of Chromosome Segregation | 3.18E-17 | 0.001 | 10 | CDC6, KIF2C, NEK2, SPAG5, BUB1, ESPL1, AURKB, RACGAP1, ECT2, BUB3 |
| 2 | 19 | Dna Replication | 3.28E-17 | 0.001 | 20 | CDC7, CDC6, CENPF, CHEK1, MCM2, POLA2, CDC25C, MCM10, MCM3, MCM4, BRCA1, RAD51, MCM6, CDT1, CDC45, MCM7, ORC6, POLQ, TOP2A |

| | | | | | | |
|---|---|---|---|---|---|---|
| 2 | 14 | Negative Regulation Of Cell Cycle Process | 1.08E-15 | 0.001 | 15 | CDC6, TTK, CENPF, CHEK1, ESPL1, CDC20, BRCA1, CDT1, CDC45, MAD2L1, BUB1, FBXO5, BUB1B, BUB3 |
| 2 | 10 | Regulation Of Mitotic Metaphase/Anaphase Transition | 1.99E-14 | 0.001 | 10 | MAD2L1, PLK1, BUB1, FBXO5, TTK, CENPF, BUB1B, ESPL1, CDC20, BUB3 |
| 2 | 12 | M/G1 Transition Of Mitotic Cell Cycle | 5.67E-13 | 0.001 | 13 | CDC7, CDC6, CDC45, MCM7, ORC6, MCM2, POLA2, MCM10, MCM3, MCM4, CDT1, MCM6 |
| 2 | 12 | Meiotic Cell Cycle | 2.00E-11 | 0.001 | 13 | EXO1, MKI67, PLK1, NEK2, MSH5, FBXO5, ESPL1, TUBG1, TOP2A, RAD54L, BUB3, RAD51 |
| 2 | 8 | Spindle Checkpoint | 3.78E-11 | 0.001 | 8.3 | MAD2L1, BUB1, TTK, CENPF, BUB1B, BIRC5, CDC20, BUB3 |
| 2 | 10 | Cytokinesis | 9.19E-11 | 0.001 | 10 | KIF23, PRC1, PLK1, ESPL1, BIRC5, CEP55, AURKB, RACGAP1, ECT2, KIF20A |
| 2 | 7 | Dna-Dependent Dna Replication Initiation | 1.31E-10 | 0.001 | 7.3 | CDC45, MCM7, MCM2, POLA2, MCM3, MCM4, MCM6 |
| 2 | 12 | Negative Regulation Of Organelle Organization | 8.37E-10 | 0.001 | 13 | MAD2L1, BUB1, FBXO5, TTK, CENPF, BUB1B, ESPL1, CHEK1, CDC20, BCOR, BUB3, BRCA1 |
| 2 | 5 | Dna Unwinding Involved In Replication | 1.75E-09 | 0.001 | 5.2 | MCM7, MCM2, MCM4, RAD51, MCM6 |
| 2 | 12 | Dna Conformation Change | 3.01E-09 | 0.001 | 13 | NCAPH, HMGB2, MCM7, DDX11, NCAPG, MCM2, ASF1B, TOP2A, SMC2, MCM4, RAD51, MCM6 |
| 2 | 7 | Dna Strand Elongation Involved In Dna Replication | 4.10E-09 | 0.001 | 7.3 | CDC45, MCM7, MCM2, POLA2, MCM3, MCM4, MCM6 |
| 2 | 9 | Organelle Assembly | 5.58E-09 | 0.002 | 9.4 | KIF23, KIF11, NEK2, FBXO5, CENPF, CENPE, AURKB, RACGAP1, MYBL2 |
| 2 | 9 | Regulation Of Microtubule-Based Process | 1.14E-08 | 0.002 | 9.4 | NEK2, SPAG5, TPX2, AURKA, CHEK1, RACGAP1, TACC3, ECT2, BRCA1 |
| 2 | 11 | Positive Regulation Of Cell Cycle Process | 1.72E-08 | 0.002 | 11 | KIF23, CDC6, PLK1, ESPL1, AURKA, BIRC5, AURKB, RACGAP1, ECT2, GTSE1, BRCA1 |
| 2 | 5 | Regulation Of Cell Cycle Cytokinesis | 3.86E-08 | 0.004 | 5.2 | KIF23, CDC6, AURKB, RACGAP1, ECT2 |
| 2 | 6 | Regulation Of G2/M Transition Of Mitotic Cell Cycle | 4.83E-08 | 0.005 | 6.3 | CDC6, CDC45, CENPF, TOP2A, CCNA2, CDT1 |
| 2 | 8 | Anaphase-Promoting Complex-Dependent Proteasomal Ubiquitin-Dependent Protein Catabolic Process | 1.03E-07 | 0.008 | 8.3 | MAD2L1, PLK1, FBXO5, BUB1B, AURKA, CDC20, AURKB, BUB3 |
| 2 | 10 | Dna Recombination | 1.59E-07 | 0.011 | 10 | EXO1, HMGB2, RAD51AP1, MSH5, POLA2, TOP2A, SMC2, RAD54L, BRCA1, RAD51 |
| 2 | 4 | Mitotic Spindle | 4.95E-07 | 0.027 | 4.2 | KIF23, SPAG5, RACGAP1, ECT2 |
| 2 | 13 | Dna Repair | 5.58E-07 | 0.028 | 14 | EXO1, HMGB2, RAD51AP1, FANCI, MSH5, FOXM1, CHEK1, POLQ, TOP2A, SMC2, RAD54L, BRCA1, RAD51 |
| 3 | 8 | Response To Ionizing Radiation | 8.12E-07 | 0.038 | 8.1 | DCLRE1C, PAXIP1, XRCC2, CLK2, RAD9A, TOPBP1, ATR, ATM |
| 4 | 18 | Response To Virus | 1.20E-23 | 0.001 | 46 | IFIH1, OAS3, HERC5, RSAD2, TLR3, IFI44L, OAS1, IFI44, OAS2, STAT1, IFI35, CXCL10, IRF9, IRF7, DDX60, MX1, EIF2AK2, MX2 |

| | | | | | | |
|---|---|---|---|---|---|---|
| 4 | 13 | Response To Type I Interferon | 9.93E-22 | 0.001 | 33 | IFIT3, IRF9, USP18, SP100, IRF7, OAS3, OAS1, OAS2, XAF1, MX1, STAT1, MX2, IFI35 |
| 4 | 9 | Cellular Response To Interferon-Gamma | 1.06E-12 | 0.001 | 23 | IRF9, SP100, IRF7, OAS3, TLR3, OAS1, OAS2, STAT1, GBP1 |
| 5 | 22 | Mitosis | 2.81E-23 | 0.001 | 40 | KIF23, KIF11, KIF15, TPX2, KNTC1, CENPF, NDC80, CENPE, AURKB, MYBL2, CDK2, KIF2C, NCAPH, TIMELESS, PLK1, SPAG5, NCAPG, BUB1B, FBXO5, ZWILCH, ASPM, CDCA3 |
| 5 | 19 | Microtubule-Based Process | 3.32E-18 | 0.001 | 35 | KIF23, KIF14, KIF4A, KIF11, PRC1, KIF15, TTK, NDC80, CENPE, AURKB, TACC3, MYBL2, GTSE1, CDK2, KIF2C, PLK1, SPAG5, BUB1B, FBXO5 |
| 5 | 16 | Regulation Of Cell Cycle Arrest | 2.61E-15 | 0.001 | 29 | FOXM1, KNTC1, TTK, CENPF, CHEK1, MCM2, MCM3, MCM4, GTSE1, CDK2, CDC45, MCM7, PLK1, BUB1B, ZWILCH, TOP2A |
| 5 | 11 | Spindle Organization | 4.34E-15 | 0.001 | 20 | KIF23, KIF11, PRC1, SPAG5, FBXO5, TTK, BUB1B, NDC80, AURKB, MYBL2, TACC3 |
| 5 | 13 | Dna Replication | 3.86E-13 | 0.001 | 24 | NASP, LIG1, CENPF, CHEK1, MCM2, MCM3, MCM4, CDK2, RAD51, CDC45, MCM7, CHAF1A, TOP2A |
| 5 | 14 | Interphase Of Mitotic Cell Cycle | 4.96E-11 | 0.001 | 25 | FOXM1, LIG1, CENPF, CHEK1, MCM2, MCM3, MCM4, GTSE1, CDK2, CDC45, MCM7, PLK1, FBXO5, TOP2A |
| 5 | 13 | Regulation Of Mitotic Cell Cycle | 1.56E-10 | 0.001 | 24 | TPX2, KNTC1, TTK, CENPF, CHEK1, CDK2, GTSE1, CDC45, PLK1, FBXO5, BUB1B, ZWILCH, TOP2A |
| 5 | 13 | Dna Repair | 6.16E-10 | 0.001 | 24 | EXO1, RAD51AP1, XRCC2, FANCI, LIG1, FOXM1, TRIM28, CHEK1, CHAF1A, TOP2A, CDK2, FANCC, RAD51 |
| 5 | 6 | Dna Strand Elongation Involved In Dna Replication | 5.45E-09 | 0.001 | 11 | CDC45, MCM7, LIG1, MCM2, MCM3, MCM4 |
| 5 | 8 | Meiosis | 1.19E-08 | 0.002 | 15 | EXO1, XRCC2, MKI67, PLK1, FBXO5, TOP2A, CDK2, RAD51 |
| 5 | 5 | Dna-Dependent Dna Replication Initiation | 2.78E-08 | 0.003 | 9.1 | CDC45, MCM7, MCM2, MCM3, MCM4 |
| 5 | 4 | Dna Unwinding Involved In Replication | 3.00E-08 | 0.003 | 7.3 | MCM7, MCM2, MCM4, RAD51 |
| 5 | 7 | Organelle Assembly | 3.84E-08 | 0.004 | 13 | KIF23, KIF11, FBXO5, CENPF, CENPE, AURKB, MYBL2 |
| 5 | 5 | Negative Regulation Of Mitosis | 2.05E-07 | 0.014 | 9.1 | FBXO5, TTK, CENPF, BUB1B, CHEK1 |
| 5 | 5 | Regulation Of Mitotic Metaphase/Anaphase Transition | 2.44E-07 | 0.015 | 9.1 | PLK1, FBXO5, TTK, CENPF, BUB1B |
| 5 | 8 | Dna Conformation Change | 4.62E-07 | 0.024 | 15 | NCAPH, MCM7, NCAPG, MCM2, CHAF1A, TOP2A, MCM4, RAD51 |
| 7 | 15 | Mitosis | 3.47E-15 | 0.001 | 35 | CENPM, BIRC5, NDC80, AURKA, AURKB, RACGAP1, PPP1CC, CCNB1, SPC25, NCAPH, MAD2L1, OIP5, CENPA, BUB1B, CDCA3 |
| 7 | 10 | Mitotic Prometaphase | 5.67E-14 | 0.001 | 23 | CCNB1, SPC25, CENPM, MAD2L1, CENPA, BUB1B, NDC80, BIRC5, AURKB, PPP1CC |
| 7 | 7 | Spindle Organization | 2.52E-09 | 0.001 | 16 | CEP72, SPC25, BUB1B, AURKA, NDC80, AURKB, RACGAP1 |

| | | | | | | |
|---|---|---|---|---|---|---|
| 8 | 22 | Response To Bacterium | 4.94E-19 | 0.001 | 30 | ICAM1, CCL2, CEBPB, LYN, NCF1, HCK, TLR1, MMP7, SOD2, SLC11A1, TNFRSF1B, THBD, CCL20, CCR5, STAB1, PLCG2, PLA2G2A, IL1B, SERPINA1, FAS, CD14, SYK |
| 8 | 15 | Cytokine-Mediated Signaling Pathway | 1.73E-10 | 0.001 | 21 | ICAM1, IL1R2, PTPN6, CCL2, PTPN2, HCK, CCR1, TNFRSF1B, CD44, CCR5, IL10RA, IL4R, IL1B, FAS, IL13RA1 |
| 8 | 10 | Cellular Response To Biotic Stimulus | 1.96E-10 | 0.001 | 14 | ICAM1, TNFRSF1B, CCL2, LYN, CCR5, HCK, TLR1, IL1B, CD14, SYK |
| 8 | 12 | Leukocyte Migration | 9.32E-10 | 0.001 | 16 | ICAM1, PTPN6, THBD, CCL2, LYN, CCR5, HCK, CCR1, IL1B, TREM1, ITGAM, SYK |
| 8 | 12 | Immune Effector Process | 1.20E-09 | 0.001 | 16 | ICAM1, PTPRC, PTPN6, SLC11A1, LYN, NCF1, HCK, TLR1, PLCG2, FAS, PTX3, SYK |
| 8 | 6 | B Cell Receptor Signaling Pathway | 7.54E-09 | 0.002 | 8.2 | PTPRC, PTPN6, LYN, PLCG2, SYK, BTK |
| 8 | 13 | Cellular Cation Homeostasis | 8.63E-09 | 0.002 | 18 | TCIRG1, STEAP3, PTPRC, SLC39A14, CCL2, CCR1, HP, SLC11A1, CCR5, PLCG2, IL1B, STC1, CP |
| 8 | 8 | Response To Interferon-Gamma | 4.45E-08 | 0.005 | 11 | ICAM1, PTPN6, SLC11A1, CCL2, CD44, PTPN2, HCK, UBD |
| 8 | 12 | Positive Regulation Of Immune Response | 1.12E-07 | 0.009 | 16 | PTPRC, PTPN6, SLC11A1, LYN, HCK, TLR1, PLCG2, IL1B, WAS, CD14, SYK, BTK |
| 8 | 10 | Regulation Of Immune Effector Process | 1.29E-07 | 0.009 | 14 | ICAM1, PTPRC, PTPN6, CCL2, LYN, HCK, IL4R, IL1B, SYK, BTK |
| 8 | 11 | Leukocyte Activation | 8.33E-07 | 0.039 | 15 | ICAM1, PTPRC, SLC11A1, LYN, TLR1, PLCG2, UBD, FAS, WAS, SYK, BTK |
| 10 | 18 | M Phase Of Mitotic Cell Cycle | 6.16E-15 | 0.001 | 25 | BORA, TPX2, KNTC1, NDC80, AURKA, CEP55, SMC2, NCAPD3, CDK2, KIF2C, CDCA8, PLK1, NCAPG, ZWINT, BUB1B, ZWILCH, CCNA2, KIF20A |
| 10 | 16 | Cell Cycle Checkpoint | 6.24E-14 | 0.001 | 22 | KNTC1, CHEK1, MCM2, MCM3, MCM4, CDK2, CDT1, RFWD3, PLK1, ZWINT, MAPK14, BUB1B, FANCG, ZWILCH, CCNA2, ORC2 |
| 10 | 14 | Interphase Of Mitotic Cell Cycle | 2.25E-09 | 0.001 | 19 | SKP2, CHEK1, MCM2, MCM3, CDKN3, MCM4, CDK2, CDT1, PLK1, TFDP2, CCNA2, ORC2, MELK, FEN1 |
| 10 | 11 | Dna Replication | 3.71E-09 | 0.001 | 15 | BAZ1A, SUPT16H, CHEK1, MCM2, POLQ, MCM3, MCM4, CDK2, FEN1, ORC2, CDT1 |
| 10 | 13 | Regulation Of Mitotic Cell Cycle | 5.31E-09 | 0.001 | 18 | BORA, KNTC1, TPX2, CHEK1, AURKA, CDK2, CDT1, RFWD3, PLK1, ZWINT, BUB1B, ZWILCH, CCNA2 |
| 10 | 7 | Mrna 3'-End Processing | 1.01E-07 | 0.008 | 9.7 | SRSF1, SRSF2, NCBP1, CSTF2, EIF4A1, NUDT21, CPSF6 |
| 12 | 12 | Angiogenesis | 4.24E-11 | 0.001 | 26 | EDNRA, COL4A2, COL4A1, CD34, ITGA5, PDGFRB, ENPEP, MCAM, MYH9, ANGPT2, ITGB1, FN1 |
| 12 | 10 | Extracellular Matrix Organization | 1.12E-10 | 0.001 | 21 | COL4A2, PXDN, COL3A1, COL1A2, OLFML2A, NID1, COL1A1, LAMC1, COL5A2, COL5A1 |
| 12 | 6 | Glomerulus Development | 1.03E-08 | 0.002 | 13 | NOTCH3, CD34, PDGFRB, NID1, ENPEP, ANGPT2 |
| 12 | 8 | Cell-Substrate Adhesion | 1.49E-08 | 0.002 | 17 | CD34, COL3A1, NID1, NID2, LAMC1, LAMB1, ITGB1, FN1 |
| 12 | 5 | Cellular Response To Amino Acid Stimulus | 1.80E-07 | 0.013 | 11 | COL4A1, COL3A1, COL1A2, COL1A1, COL5A2 |

| | | | | | | |
|---|---|---|---|---|---|---|
| 12 | 8 | Leukocyte Migration | 4.76E-07 | 0.026 | 17 | CD34, ITGA5, COL1A2, COL1A1, MYH9, ANGPT2, ITGB1, FN1 |
| 12 | 5 | Skin Development | 7.95E-07 | 0.038 | 11 | COL3A1, COL1A2, COL1A1, COL5A2, COL5A1 |
| 13 | 7 | Spindle Organization | 4.34E-07 | 0.023 | 7.8 | KIF23, SPC25, KIF11, NEK2, FBXO5, NDC80, RACGAP1 |
| 14 | 13 | Angiogenesis | 1.44E-10 | 0.001 | 20 | NRP2, NRP1, SRPX2, ITGA5, ANG, TGFBI, MMP19, VEGFA, SERPINE1, SHC1, THBS1, PLAU, ANXA2 |
| 14 | 7 | Regulation Of Hemostasis | 2.77E-08 | 0.003 | 11 | CAV1, THBD, SERPINE1, THBS1, PLAU, PLAUR, ANXA2 |
| 14 | 9 | Regulation Of Epithelial Cell Proliferation | 4.02E-07 | 0.021 | 14 | NRP2, CAV1, NRP1, ANG, GRN, VEGFA, LAMC1, THBS1, LAMB1 |
| 15 | 29 | M Phase Of Mitotic Cell Cycle | 8.21E-15 | 0.001 | 13 | BORA, CEP55, AURKB, MYBL2, KIAA1009, KIF2C, NCAPH, CDCA8, DDX11, NCAPG, RHOA, ASPM, CDCA3, CDC6, CENPM, KIF11, KIF15, CENPF, CDC20, CENPE, UBE2C, MLF1IP, CDC25A, MAD2L1, PLK1, SPAG5, ZWINT, BUB1B, KIF20A |
| 15 | 23 | Regulation Of Cell Cycle Arrest | 6.77E-11 | 0.001 | 10 | E2F1, CDC6, DTL, SOX11, FOXM1, TTK, CENPF, CDC20, UBE2C, GTSE1, CDC25A, CDT1, PSMA2, CCND1, CDC45, MAD2L1, PLK1, ZWINT, PSMC2, BUB1B, ORC6, H2AFX, TOP2A |
| 15 | 24 | Microtubule-Based Process | 3.24E-10 | 0.001 | 10 | KIF14, KIF4A, KIF11, PRC1, PCNT, KIF15, TTK, BRCA2, CENPE, AURKB, MYBL2, TACC3, UBE2C, GTSE1, KIF2C, UXT, PLK1, SPAG5, ZWINT, RHOA, BUB1B, KIF21B, NEFL, KIF20A |
| 15 | 12 | Spindle Organization | 2.71E-09 | 0.001 | 5.2 | KIF11, PRC1, SPAG5, ZWINT, PCNT, RHOA, TTK, BUB1B, AURKB, UBE2C, MYBL2, TACC3 |
| 15 | 12 | Mitotic Prometaphase | 1.11E-08 | 0.002 | 5.2 | KIF2C, CENPM, CDCA8, MAD2L1, PLK1, ZWINT, CENPF, BUB1B, CENPE, CDC20, AURKB, MLF1IP |
| 15 | 20 | Regulation Of Mitotic Cell Cycle | 1.89E-07 | 0.014 | 8.7 | E2F1, CDC6, BORA, TTK, BRCA2, CENPF, CDC20, UBE2C, GTSE1, CDT1, PSMA2, CCND1, CDC45, MAD2L1, PLK1, ZWINT, PSMC2, BUB1B, TOP2A, LRP5 |
| 15 | 12 | Chromosome Segregation | 4.70E-07 | 0.026 | 5.2 | KIF2C, NCAPH, MAD2L1, DDX11, SPAG5, NCAPG, ZWINT, CENPF, BRCA2, CENPE, AURKB, TOP2A |
| 16 | 40 | Mitosis | 1.62E-43 | 0.001 | 43 | KIF23, NEK2, BORA, KNTC1, AURKA, AURKB, CEP55, CHEK2, MYBL2, KIF2C, SPC25, NCAPH, CDCA8, DDX11, CENPA, OIP5, NCAPG, BUB1, CCNA2, ASPM, ERCC6L, CDCA3, CDC6, CENPN, KIF11, DLGAP5, KIF15, TPX2, CDC20, CENPE, ESPL1, NDC80, CDC25C, SMC2, CDC25A, CDK2, CCNB2, SPAG5, PLK1, BUB1B |
| 16 | 22 | Cell Cycle Checkpoint | 1.80E-19 | 0.001 | 24 | CDC6, KNTC1, TTK, CDC20, CHEK1, CHEK2, CDC25C, MCM10, MCM4, GTSE1, CDC25A, BRCA1, CDK2, CDT1, CDC45, CCNB2, RFWD3, PLK1, BUB1, BUB1B, CCNA2, ORC1 |
| 16 | 24 | Microtubule-Based Process | 1.92E-19 | 0.001 | 26 | KIF23, KIF14, KIF11, NEK2, DLGAP5, KIF15, TTK, BRCA2, NDC80, CENPE, AURKA, ESPL1, AURKB, CHEK2, MYBL2, GTSE1, BRCA1, CDK2, KIF2C, SPC25, PLK1, SPAG5, CENPA, BUB1B |

| | | | | | | |
|---|---|---|---|---|---|---|
| 16 | 20 | Dna Replication | 7.95E-19 | 0.001 | 22 | CDC6, LIG3, BRCA2, CHEK1, POLA2, CDC25C, MCM10, MCM4, CDC25A, BRCA1, CDK2, TK1, RAD51, CDT1, CDC45, POLD1, POLG2, POLQ, ORC1, FEN1 |
| 16 | 23 | Regulation Of Mitotic Cell Cycle | 2.91E-18 | 0.001 | 25 | CDC6, NEK2, DLGAP5, BORA, TPX2, KNTC1, TTK, BRCA2, CDC20, AURKA, ESPL1, CHEK1, CDC25C, GTSE1, CDK2, CDT1, CDC45, RFWD3, PLK1, POLD1, BUB1, BUB1B, CCNA2 |
| 16 | 16 | Chromosome Segregation | 3.60E-17 | 0.001 | 17 | NEK2, DLGAP5, BRCA2, NDC80, CENPE, ESPL1, AURKB, SMC2, BRCA1, KIF2C, SPC25, NCAPH, DDX11, SPAG5, NCAPG, BUB1 |
| 16 | 13 | Spindle Organization | 1.36E-15 | 0.001 | 14 | KIF23, KIF11, NEK2, TTK, ESPL1, NDC80, AURKA, AURKB, CHEK2, MYBL2, SPC25, SPAG5, BUB1B |
| 16 | 21 | Interphase Of Mitotic Cell Cycle | 7.12E-15 | 0.001 | 23 | CDC6, NEK2, SKP2, CHEK1, CHEK2, POLA2, CDC25C, MCM10, MCM4, GTSE1, CDC25A, CDK2, CDT1, CDC45, CCNB2, DDX11, PLK1, POLD1, CCNA2, ORC1, FEN1 |
| 16 | 13 | Regulation Of Nuclear Division | 8.69E-15 | 0.001 | 14 | CDC6, NEK2, DLGAP5, BORA, TTK, ESPL1, CDC20, CHEK1, AURKA, CDC25C, PLK1, BUB1, BUB1B |
| 16 | 10 | Mitotic Sister Chromatid Segregation | 2.85E-13 | 0.001 | 11 | NCAPH, DDX11, NEK2, SPAG5, NCAPG, DLGAP5, ESPL1, CENPE, NDC80, SMC2 |
| 16 | 18 | Dna Repair | 9.02E-12 | 0.001 | 20 | EXO1, NEIL3, LIG3, BRCA2, CHEK1, CHEK2, RAD54L, SMC2, BRCA1, CDK2, RAD51, RFWD3, FANCI, POLD1, PARPBP, POLG2, POLQ, FEN1 |
| 16 | 12 | S Phase Of Mitotic Cell Cycle | 3.09E-11 | 0.001 | 13 | CDC6, CDC45, DDX11, POLD1, SKP2, POLA2, ORC1, MCM4, CDK2, CDC25A, FEN1, CDT1 |
| 16 | 7 | Regulation Of Chromosome Segregation | 4.36E-11 | 0.001 | 7.6 | CDC6, KIF2C, NEK2, SPAG5, BUB1, ESPL1, AURKB |
| 16 | 10 | Negative Regulation Of Cell Cycle Process | 3.74E-10 | 0.001 | 11 | CDC6, CDC45, BUB1, TTK, BUB1B, ESPL1, CHEK1, CDC20, BRCA1, CDT1 |
| 16 | 10 | Meiosis | 3.04E-09 | 0.001 | 11 | EXO1, MKI67, PLK1, NEK2, LIG3, BRCA2, ESPL1, RAD54L, CDK2, RAD51 |
| 16 | 10 | Dna Recombination | 1.18E-07 | 0.009 | 11 | EXO1, POLD1, LIG3, BRCA2, POLA2, SMC2, RAD54L, BRCA1, FEN1, RAD51 |
| 16 | 10 | Positive Regulation Of Cell Cycle Process | 1.43E-07 | 0.009 | 11 | KIF23, CDC6, PLK1, DLGAP5, ESPL1, AURKA, AURKB, GTSE1, CDK2, BRCA1 |
| 16 | 10 | Dna Conformation Change | 2.71E-07 | 0.018 | 11 | CENPN, NCAPH, DDX11, NCAPG, CENPA, OIP5, ASF1B, SMC2, MCM4, RAD51 |
| 16 | 7 | Regulation Of Microtubule Cytoskeleton Organization | 7.48E-07 | 0.037 | 7.6 | NEK2, SPAG5, BORA, TPX2, AURKA, CHEK1, BRCA1 |
| 17 | 15 | Extracellular Matrix Organization | 1.10E-16 | 0.001 | 25 | PXDN, COL4A2, MMP9, OLFML2B, LUM, COL3A1, OLFML2A, COL5A2, COL5A1, TGFBI, COL6A2, COL1A2, LOX, COL1A1, LOXL2 |
| 17 | 8 | Collagen Fibril Organization | 1.83E-12 | 0.001 | 14 | LUM, COL3A1, COL1A2, LOX, COL1A1, LOXL2, COL5A2, COL5A1 |
| 17 | 13 | Angiogenesis | 4.84E-11 | 0.001 | 22 | COL4A2, SRPX2, ITGA5, SERPINE1, TGFBI, MMP19, PDGFRB, THBS1, LOXL2, MYH9, ITGB1, PLAU, FN1 |
| 17 | 6 | Collagen Metabolic Process | 6.75E-09 | 0.002 | 10 | TRAM2, MMP9, MMP19, COL3A1, COL1A1, COL5A1 |

| | | | | | | |
|---|---|---|---|---|---|---|
| 17 | 4 | Collagen Biosynthetic Process | 8.65E-09 | 0.002 | 6.8 | TRAM2, COL3A1, COL1A1, COL5A1 |
| 17 | 4 | Protein Heterotrimerization | 4.00E-08 | 0.005 | 6.8 | COL1A2, COL6A2, COL6A1, COL1A1 |
| 17 | 5 | Cellular Response To Amino Acid Stimulus | 5.70E-07 | 0.028 | 8.5 | COL3A1, COL1A2, COL6A1, COL1A1, COL5A2 |
| 19 | 9 | Neurotransmitter Secretion | 1.59E-12 | 0.001 | 19 | SLC17A7, GLS2, RAB3A, SYT1, STX1A, GAD2, SYN1, SYN2, SNAP25 |
| 19 | 6 | Glutamate Secretion | 1.67E-11 | 0.001 | 13 | SLC17A7, GLS2, RAB3A, SYT1, STX1A, SNAP25 |
| 19 | 5 | Synaptic Vesicle Transport | 5.46E-07 | 0.028 | 10 | RAB3A, SYT1, STX1A, SNAP25, SH3GL2 |
| 20 | 10 | Neurotransmitter Transport | 2.72E-10 | 0.001 | 14 | SLC17A7, RAB3A, SYT1, GAD2, DOC2A, SYN1, SYN2, SNCA, SV2B, SNAP25 |
| 20 | 9 | Generation Of A Signal Involved In Cell-Cell Signaling | 1.81E-08 | 0.002 | 12 | SLC17A7, RAB3A, SYT1, GAD2, EPHB6, DOC2A, SYN1, SYN2, SNAP25 |
| 20 | 6 | Synaptic Vesicle Transport | 1.53E-07 | 0.01 | 8.2 | SYP, RAB3A, SYT1, DOC2A, SNCA, SNAP25 |
| 20 | 10 | Regulation Of Neurological System Process | 1.96E-07 | 0.014 | 14 | SYP, RAB3A, CCK, CCKBR, NCDN, SNCA, NEUROD2, TAC1, CAMK2A, HTR2A |
| 22 | 27 | Response To Bacterium | 8.40E-16 | 0.001 | 16 | FGR, IL18, TLR1, TLR2, SLC11A1, NOD2, TNFRSF1B, MYD88, FCER1G, CD4, SERPINA1, SYK, C5AR1, CEBPB, NCF2, SOCS3, NCF1, HCK, MYO1F, HLA-B, STAT1, SOD2, LILRB1, THBD, CCR5, PLCG2, CD14 |
| 22 | 22 | Immune Effector Process | 8.65E-15 | 0.001 | 13 | PTPRC, NCF1, HCK, TLR1, MYO1F, SERPING1, PRKCD, STAT6, LILRB1, C1QA, SLC11A1, C1QB, CD86, NOD2, MYD88, PLCG2, C1RL, FCER1G, CTSC, C2, CFD, SYK |
| 22 | 23 | Activation Of Immune Response | 2.11E-14 | 0.001 | 14 | FYB, PTPRC, HCK, TLR1, TLR2, NCKAP1L, SERPING1, TLR5, C1QA, C1QB, CD86, NOD2, MYD88, RPS6KA1, PLCG2, C1RL, FCER1G, CD4, C2, CFD, CD14, DUSP6, SYK |
| 22 | 16 | Adaptive Immune Response | 4.15E-13 | 0.001 | 9.8 | IL18, SERPING1, CTSS, PRKCD, CTSL1, STAT6, C1QA, SLC11A1, C1QB, NOD2, MYD88, C1RL, FCER1G, CTSC, C2, SYK |
| 22 | 20 | Immune Response-Regulating Signaling Pathway | 1.90E-12 | 0.001 | 12 | FYB, PTPRC, FGR, HCK, TLR1, TLR2, NCKAP1L, TLR5, HLA-G, LILRB1, CD86, NOD2, MYD88, RPS6KA1, PLCG2, FCER1G, CD4, CD14, DUSP6, SYK |
| 22 | 14 | Defense Response To Bacterium | 1.60E-11 | 0.001 | 8.5 | SLC11A1, NOD2, C5AR1, MYD88, CCR5, FGR, NCF1, HCK, TLR1, TLR2, FCER1G, MYO1F, CD4, SYK |
| 22 | 17 | Regulation Of Immune Effector Process | 3.99E-10 | 0.001 | 10 | PTPRC, FGR, AP1B1, HCK, TLR2, SERPING1, STAT6, LILRB1, CD86, NOD2, DOCK2, IL4R, FCER1G, CD4, C2, SASH3, SYK |
| 22 | 18 | Positive Regulation Of Cell Activation | 6.42E-10 | 0.001 | 11 | PTPRC, FGR, IL18, NCKAP1L, VAV1, HLA-G, STAT6, LILRB1, CD86, NOD2, MYD88, RPS6KA1, IL4R, MAP3K8, FCER1G, CD4, SASH3, SYK |
| 22 | 17 | Regulation Of Innate Immune Response | 6.68E-10 | 0.001 | 10 | FGR, PTPN2, SOCS3, HCK, TLR1, TLR2, MYO1F, SERPING1, TLR5, STAT1, LILRB1, CD86, NOD2, MYD88, RPS6KA1, CD14, DUSP6 |

| | | | | | | |
|---|---|---|---|---|---|---|
| 22 | 20 | Cytokine-Mediated Signaling Pathway | 2.61E-09 | 0.001 | 12 | PTPN2, SOCS3, HCK, CCR1, IFI30, HLA-C, HLA-B, STAT1, PRKCD, HLA-G, TRADD, STAT6, TNFRSF1B, MYD88, CCR5, IL10RA, IL4R, PYCARD, IL13RA1, CSF1R |
| 22 | 8 | Positive Regulation Of Tumor Necrosis Factor Production | 3.02E-09 | 0.001 | 4.9 | NOD2, MYD88, CCR5, IL18, TLR2, FCER1G, SASH3, CD14 |
| 22 | 10 | Regulation Of Interleukin-6 Production | 3.58E-09 | 0.001 | 6.1 | NOD2, MYD88, CEBPB, CCR5, MAPK13, TLR1, TLR2, FCER1G, NCKAP1L, HLA-B |
| 22 | 16 | Leukocyte Migration | 3.78E-09 | 0.001 | 9.8 | C5AR1, HCK, CCR1, NCKAP1L, ITGB2, ITGAM, SLC7A7, SLC16A3, THBD, CCR5, ITGA5, FCER1G, COL1A1, MERTK, SYK, SPP1 |
| 22 | 12 | Cellular Response To Biotic Stimulus | 4.31E-09 | 0.001 | 7.3 | LILRB1, TNFRSF1B, NOD2, MYD88, CCR5, HCK, IL18, TLR1, TLR2, STAT1, CD14, SYK |
| 22 | 15 | Positive Regulation Of Cytokine Production | 5.27E-09 | 0.001 | 9.1 | IL18, TLR2, TLR5, HLA-G, SLC11A1, NOD2, MYD88, CCR5, MAPK13, IL4R, PYCARD, FCER1G, SASH3, CD14, CSF1R |
| 22 | 8 | Defense Response To Gram-Positive Bacterium | 5.46E-09 | 0.002 | 4.9 | NOD2, C5AR1, MYD88, FGR, NCF1, HCK, TLR2, MYO1F |
| 22 | 9 | Positive Regulation Of Cytokine Secretion | 7.53E-09 | 0.002 | 5.5 | NOD2, FGR, CCR5, IL4R, TLR2, PYCARD, CD14, SYK, CSF1R |
| 22 | 8 | Regulation Of Phagocytosis | 2.06E-08 | 0.002 | 4.9 | SLC11A1, NOD2, FGR, HCK, FCER1G, NCKAP1L, MERTK, SYK |
| 22 | 11 | Response To Interferon-Gamma | 2.09E-08 | 0.002 | 6.7 | SLC11A1, PTPN2, SOCS3, HCK, IL18, IFI30, HLA-C, HLA-B, STAT1, PRKCD, HLA-G |
| 22 | 5 | Positive Regulation Of Gamma-Delta T Cell Activation | 6.08E-08 | 0.006 | 3 | LILRB1, PTPRC, NOD2, NCKAP1L, SYK |
| 22 | 10 | Antigen Processing And Presentation Of Exogenous Peptide Antigen Via Mhc Class I | 7.77E-08 | 0.006 | 6.1 | CYBB, NCF2, NCF1, NCF4, FCER1G, IFI30, HLA-C, HLA-B, CTSS, HLA-G |
| 22 | 10 | Regulation Of Multi-Organism Process | 3.22E-07 | 0.019 | 6.1 | LILRB1, DOCK2, NOD2, MYD88, CCR5, AP1B1, HCK, TLR2, CD4, TIMP1 |
| 22 | 8 | Regulation Of Alpha-Beta T Cell Activation | 4.51E-07 | 0.024 | 4.9 | LILRB1, PTPRC, CD86, IL4R, IL18, NCKAP1L, SASH3, SYK |
| 22 | 5 | Production Of Molecular Mediator Involved In Inflammatory Response | 5.91E-07 | 0.029 | 3 | IL4R, ALOX5AP, FCER1G, ALOX5, SYK |
| 22 | 5 | Respiratory Burst | 9.01E-07 | 0.043 | 3 | SLC11A1, CYBB, NCF2, NCF1, HCK |
| 26 | 16 | Regulation Of Neuron Differentiation | 8.92E-09 | 0.002 | 13 | PALM, RAP2A, CDK5R1, TNIK, NLGN1, SLIT1, TTC3, NTRK3, EDNRB, NOTCH1, MAPT, RAPGEF4, OLIG2, DBN1, TCF12, NKX2-2 |
| 26 | 7 | Regulation Of Dendrite Development | 7.16E-07 | 0.036 | 5.9 | RAP2A, PALM, CDK5R1, TNIK, NLGN1, RAPGEF4, DBN1 |
| 27 | 36 | M Phase Of Mitotic Cell Cycle | 1.17E-49 | 0.001 | 69 | KIF23, AURKA, AURKB, PTTG1, KIF2C, SPC25, CDCA8, CENPA, OIP5, NCAPG, CCNA2, ASPM, CDCA3, CENPN, CDK1, KIF11, DLGAP5, TPX2, NUSAP1, CENPF, CDC20, NDC80, BIRC5, CENPE, PBK, CDC25C, RACGAP1, UBE2C, MLF1IP, CCNB1, MAD2L1, CCNB2, SPAG5, BUB1B, UBE2S, KIF20A |

| | | | | | | |
|---|---|---|---|---|---|---|
| 27 | 15 | Mitotic Prometaphase | 3.11E-22 | 0.001 | 29 | CENPN, CENPF, BIRC5, NDC80, CENPE, CDC20, AURKB, MLF1IP, CCNB1, SPC25, KIF2C, CDCA8, MAD2L1, CENPA, BUB1B |
| 27 | 20 | Microtubule-Based Process | 2.16E-20 | 0.001 | 38 | KIF23, CDK1, KIF4A, KIF11, PRC1, DLGAP5, TTK, NUSAP1, NDC80, CENPE, AURKA, AURKB, UBE2C, RACGAP1, SPC25, KIF2C, SPAG5, CENPA, BUB1B, KIF20A |
| 27 | 15 | Chromosome Segregation | 6.72E-20 | 0.001 | 29 | CCNB1, KIF2C, SPC25, MAD2L1, SPAG5, NCAPG, DLGAP5, CENPF, NUSAP1, CENPE, NDC80, BIRC5, AURKB, PTTG1, TOP2A |
| 27 | 12 | Spindle Organization | 2.65E-17 | 0.001 | 23 | KIF23, SPC25, KIF11, PRC1, SPAG5, TTK, BUB1B, AURKA, NDC80, AURKB, RACGAP1, UBE2C |
| 27 | 16 | Regulation Of Cell Cycle Arrest | 6.82E-16 | 0.001 | 31 | CDK1, DTL, FOXM1, TTK, CENPF, BIRC5, CDC20, MCM2, UBE2C, CDC25C, CCNB1, MAD2L1, CCNB2, BUB1B, CCNA2, TOP2A |
| 27 | 16 | Regulation Of Mitotic Cell Cycle | 8.71E-15 | 0.001 | 31 | CDK1, DLGAP5, TPX2, TTK, NUSAP1, CENPF, BIRC5, AURKA, CDC20, UBE2C, CDC25C, CCNB1, MAD2L1, BUB1B, CCNA2, TOP2A |
| 27 | 8 | Mitotic Spindle Organization | 1.52E-13 | 0.001 | 15 | KIF23, SPC25, KIF11, PRC1, TTK, NDC80, AURKB, RACGAP1 |
| 27 | 8 | Spindle Checkpoint | 2.14E-13 | 0.001 | 15 | CCNB1, MAD2L1, TTK, CENPF, BUB1B, BIRC5, CDC20, UBE2C |
| 27 | 10 | Anaphase-Promoting Complex-Dependent Proteasomal Ubiquitin-Dependent Protein Catabolic Process | 4.18E-13 | 0.001 | 19 | CCNB1, CDK1, MAD2L1, BUB1B, AURKA, CDC20, PTTG1, AURKB, UBE2C, UBE2S |
| 27 | 10 | Regulation Of Nuclear Division | 4.18E-13 | 0.001 | 19 | MAD2L1, DLGAP5, TTK, CENPF, BUB1B, NUSAP1, AURKA, CDC20, UBE2C, CDC25C |
| 27 | 7 | Regulation Of Mitotic Metaphase/Anaphase Transition | 3.62E-11 | 0.001 | 13 | MAD2L1, DLGAP5, TTK, CENPF, BUB1B, CDC20, UBE2C |
| 27 | 9 | Dna Packaging | 4.86E-10 | 0.001 | 17 | CDK1, CENPN, NCAPG, CENPA, OIP5, NUSAP1, MCM2, TOP2A, MLF1IP |
| 27 | 9 | G2/M Transition Of Mitotic Cell Cycle | 1.76E-09 | 0.001 | 17 | CCNB1, CDK1, CCNB2, FOXM1, BIRC5, CDC25C, TOP2A, CCNA2, MELK |
| 27 | 8 | Protein-Dna Complex Assembly | 3.24E-09 | 0.001 | 15 | CENPN, CENPA, OIP5, CENPF, CENPE, MCM2, MLF1IP, RAD51 |
| 27 | 12 | Interphase Of Mitotic Cell Cycle | 3.25E-09 | 0.001 | 23 | CCNB1, CDK1, GINS2, CCNB2, FOXM1, CENPF, BIRC5, MCM2, CDC25C, TOP2A, CCNA2, MELK |
| 27 | 9 | Organelle Localization | 4.13E-09 | 0.001 | 17 | CCNB1, CENPA, DLGAP5, CENPF, NUSAP1, CENPE, NDC80, BIRC5, ASPM |
| 27 | 5 | Chromosome Localization | 7.64E-09 | 0.002 | 9.6 | CCNB1, DLGAP5, CENPF, CENPE, BIRC5 |
| 27 | 5 | Regulation Of Chromosome Segregation | 1.04E-08 | 0.002 | 9.6 | CCNB1, KIF2C, SPAG5, AURKB, RACGAP1 |
| 27 | 7 | Cytokinesis | 1.09E-08 | 0.002 | 13 | KIF23, PRC1, NUSAP1, BIRC5, AURKB, RACGAP1, KIF20A |
| 27 | 8 | Positive Regulation Of Cell Cycle Process | 1.50E-07 | 0.01 | 15 | KIF23, DLGAP5, NUSAP1, AURKA, BIRC5, AURKB, RACGAP1, UBE2C |
| 27 | 8 | Dna Replication | 4.31E-07 | 0.022 | 15 | CDK1, GINS2, DTL, CENPF, MCM2, CDC25C, TOP2A, RAD51 |
| 27 | 8 | Cellular Protein Complex Assembly | 4.69E-07 | 0.026 | 15 | KIF23, CENPA, CENPF, CENPE, AURKB, RACGAP1, UBE2C, UBE2S |

| | | | | | | |
|---|---|---|---|---|---|---|
| 27 | 4 | Chromatin Remodeling At Centromere | 5.58E-07 | 0.028 | 7.7 | CENPN, CENPA, OIP5, MLF1IP |
| 27 | 6 | Inositol Lipid-Mediated Signaling | 8.47E-07 | 0.04 | 12 | SPAG5, BUB1B, AURKA, NDC80, UBE2C, TOP2A |
| 28 | 10 | Response To Bacterium | 3.72E-07 | 0.021 | 19 | LILRB1, TNFRSF1B, C5AR1, HCK, TLR1, MYO1F, SERPINA1, CD4, TGFB1, SYK |
| 29 | 13 | Mitosis | 8.77E-15 | 0.001 | 43 | KIF2C, SPC25, CCNB2, NEK2, CENPA, OIP5, DLGAP5, KIF15, BUB1B, BIRC5, AURKB, MLF1IP, DSCC1 |
| 29 | 12 | Interphase Of Mitotic Cell Cycle | 2.94E-12 | 0.001 | 40 | CDC7, CDC45, CCNB2, POLE2, NEK2, CDKN2C, PCNA, BIRC5, ORC6, MCM2, CDKN3, TCF3 |
| 29 | 7 | Chromosome Segregation | 4.24E-09 | 0.001 | 23 | KIF2C, SPC25, NEK2, DLGAP5, BIRC5, AURKB, DSCC1 |
| 29 | 8 | Dna Replication | 5.20E-09 | 0.001 | 27 | CDC7, CDC45, POLE2, PCNA, ORC6, MCM2, RNASEH2A, DSCC1 |
| 29 | 9 | Microtubule-Based Process | 1.16E-08 | 0.002 | 30 | KIF2C, SPC25, NEK2, CENPA, DLGAP5, KIF15, TTK, BUB1B, AURKB |
| 29 | 8 | Cell Cycle Checkpoint | 2.90E-08 | 0.003 | 27 | CDC7, CDC45, CCNB2, TTK, BUB1B, BIRC5, ORC6, MCM2 |
| 29 | 5 | Spindle Organization | 4.85E-07 | 0.026 | 17 | SPC25, NEK2, TTK, BUB1B, AURKB |
| 30 | 33 | Positive Regulation Of Immune Response | 2.47E-25 | 0.001 | 26 | HLA-DQB1, C3AR1, C3, TLR1, TLR2, TLR5, C1S, HLA-DMB, HLA-DMA, CD74, SLC11A1, FCER1G, CD4, HLA-DPB1, C2, CFD, SYK, FYB, PTPRC, PTPN6, LYN, HCK, NCKAP1L, C1QA, C1QB, CD86, PLCG2, MNDA, HLA-DPA1, VSIG4, SASH3, HLA-DRA, LCP2 |
| 30 | 30 | Activation Of Immune Response | 3.80E-25 | 0.001 | 23 | HLA-DQB1, C3AR1, C3, TLR1, TLR2, TLR5, C1S, HLA-DMB, HLA-DMA, FCER1G, CD4, HLA-DPB1, C2, CFD, SYK, FYB, PTPRC, PTPN6, LYN, HCK, NCKAP1L, C1QA, C1QB, CD86, PLCG2, MNDA, HLA-DPA1, VSIG4, HLA-DRA, LCP2 |
| 30 | 24 | Immune Effector Process | 1.97E-19 | 0.001 | 19 | HLA-DQB1, PTPRC, PTPN6, LYN, C3, HCK, TLR1, MYO1F, C1S, HLA-DMA, CD74, C1QA, LILRB1, SLC11A1, C1QB, CD86, PLCG2, FCER1G, CTSC, C2, VSIG4, CFD, LCP1, SYK |
| 30 | 20 | Immune Response-Regulating Cell Surface Receptor Signaling Pathway | 2.88E-19 | 0.001 | 16 | FYB, HLA-DQB1, PTPRC, PTPN6, C3AR1, LYN, TLR2, NCKAP1L, HLA-DMB, HLA-DMA, LILRB1, PLCG2, MNDA, FCER1G, HLA-DPA1, CD4, HLA-DPB1, HLA-DRA, LCP2, SYK |
| 30 | 20 | Positive Regulation Of T Cell Activation | 1.09E-16 | 0.001 | 16 | HLA-DQB1, PTPRC, PTPN6, LYN, AIF1, IL18, NCKAP1L, HLA-DMB, HLA-DMA, VAV1, CD74, LILRB1, CD86, CORO1A, HLA-DPA1, CD4, HLA-DPB1, SASH3, HLA-DRA, SYK |
| 30 | 24 | Regulation Of Leukocyte Activation | 2.84E-15 | 0.001 | 19 | HLA-DQB1, PTPRC, PTPN6, LYN, AIF1, IL18, NCKAP1L, HLA-DMB, SAMSN1, HLA-DMA, VAV1, CD74, LILRB1, CD86, CORO1A, MNDA, FCER1G, HLA-DPA1, CD4, HLA-DPB1, VSIG4, SASH3, HLA-DRA, SYK |
| 30 | 9 | Antigen Processing And Presentation Of Peptide Or Polysaccharide Antigen Via Mhc Class Ii | 8.59E-15 | 0.001 | 7 | HLA-DQB1, FCER1G, IFI30, HLA-DPA1, HLA-DPB1, HLA-DMB, HLA-DMA, CD74, HLA-DRA |

| | | | | | | |
|---|---|---|---|---|---|---|
| 30 | 16 | Adaptive Immune Response | 9.32E-15 | 0.001 | 12 | HLA-DQB1, C3AR1, C3, IL18, CTSS, C1S, HLA-DMA, CD74, CTSL1, C1QA, SLC11A1, C1QB, FCER1G, CTSC, C2, SYK |
| 30 | 17 | Antigen Processing And Presentation | 2.86E-14 | 0.001 | 13 | HLA-DQB1, NCF2, NCF4, IFI30, CTSS, HLA-DMB, HLA-DMA, CD74, CTSL1, CYBA, SLC11A1, CYBB, FCGR1A, FCER1G, HLA-DPA1, HLA-DPB1, HLA-DRA |
| 30 | 13 | Response To Interferon-Gamma | 5.93E-12 | 0.001 | 10 | HLA-DQB1, PTPN6, SLC11A1, AIF1, HCK, IL18, FCGR1A, IFI30, HLA-DPA1, HLA-DPB1, HLA-DMB, HLA-DMA, HLA-DRA |
| 30 | 16 | Regulation Of Immune Effector Process | 9.11E-11 | 0.001 | 12 | PTPRC, PTPN6, LYN, C3, HCK, TLR2, CD74, LILRB1, CD86, CD37, DOCK2, FCER1G, CD4, C2, SASH3, SYK |
| 30 | 19 | Response To Bacterium | 1.29E-10 | 0.001 | 15 | C5AR1, LYN, NCF2, HCK, IL18, TLR1, LYZ, TLR2, MYO1F, LILRB1, SLC11A1, TNFRSF1B, CCR5, STAB1, PLCG2, FCER1G, SERPINA1, CD4, SYK |
| 30 | 12 | Defense Response To Bacterium | 1.84E-10 | 0.001 | 9.3 | SLC11A1, C5AR1, CCR5, STAB1, HCK, TLR1, LYZ, TLR2, MYO1F, FCER1G, CD4, SYK |
| 30 | 18 | Leukocyte Activation | 9.68E-10 | 0.001 | 14 | PTPRC, LYN, AIF1, TLR1, MYO1F, HLA-DMA, VAV1, CD74, LILRB1, SLC11A1, CD86, DOCK2, PLCG2, FCER1G, CD4, LCP1, LCP2, SYK |
| 30 | 18 | Cytokine-Mediated Signaling Pathway | 1.89E-09 | 0.001 | 14 | HLA-DQB1, PTPN6, HCK, CCR1, IFI30, HLA-DMB, HLA-DMA, CD74, TNFRSF1B, CCR5, IL10RA, FCGR1A, PYCARD, HLA-DPA1, HLA-DPB1, IL13RA1, HLA-DRA, CSF1R |
| 30 | 14 | Leukocyte Migration | 9.43E-09 | 0.002 | 11 | PTPN6, C5AR1, LYN, SELL, HCK, CCR1, NCKAP1L, ITGB2, ITGAM, SLC7A7, CORO1A, CCR5, FCER1G, SYK |
| 30 | 6 | T Cell Selection | 1.01E-07 | 0.008 | 4.7 | PTPRC, DOCK2, CD4, HLA-DMA, CD74, SYK |
| 30 | 12 | Positive Regulation Of Cytokine Production | 1.55E-07 | 0.01 | 9.3 | SLC11A1, C3AR1, CCR5, C3, IL18, TLR2, PYCARD, FCER1G, TLR5, CD74, SASH3, CSF1R |
| 30 | 5 | Production Of Molecular Mediator Involved In Inflammatory Response | 1.79E-07 | 0.013 | 3.9 | LYN, ALOX5AP, FCER1G, ALOX5, SYK |
| 30 | 9 | Humoral Immune Response | 2.28E-07 | 0.015 | 7 | HLA-DQB1, C1QA, C1QB, C3, C1S, C2, VSIG4, TREM2, CFD |
| 30 | 5 | Respiratory Burst | 2.75E-07 | 0.018 | 3.9 | CYBA, SLC11A1, CYBB, NCF2, HCK |
| 30 | 8 | Myeloid Leukocyte Activation | 3.01E-07 | 0.019 | 6.2 | SLC11A1, LYN, AIF1, TLR1, MYO1F, FCER1G, SYK, LCP2 |
| 30 | 8 | Regulation Of Cytokine Secretion | 3.41E-07 | 0.019 | 6.2 | LILRB1, LYN, CCR5, TLR2, PYCARD, SRGN, SYK, CSF1R |
| 30 | 9 | Cellular Response To Biotic Stimulus | 6.10E-07 | 0.03 | 7 | LILRB1, TNFRSF1B, LYN, CCR5, HCK, IL18, TLR1, TLR2, SYK |
| 37 | 24 | Mitosis | 3.01E-28 | 0.001 | 50 | KIF23, CDC6, KIF11, NEK2, KIF15, TPX2, CENPF, BIRC5, CENPE, AURKB, CDK2, KIF2C, CDCA8, NCAPH, MAD2L1, SPAG5, CENPA, OIP5, NCAPG, BUB1, BUB1B, CCNA2, ASPM, CDCA3 |
| 37 | 16 | Microtubule-Based Process | 3.10E-15 | 0.001 | 33 | KIF14, KIF23, KIF4A, KIF11, NEK2, KIF15, TTK, CENPE, AURKB, BRCA1, CDK2, KIF2C, PLK4, CENPA, SPAG5, BUB1B |

| | | | | | | |
|---|---|---|---|---|---|---|
| 37 | 12 | Chromosome Segregation | 3.62E-15 | 0.001 | 25 | KIF2C, NCAPH, MAD2L1, NEK2, SPAG5, NCAPG, BUB1, CENPF, CENPE, BIRC5, AURKB, BRCA1 |
| 37 | 7 | Regulation Of Chromosome Segregation | 3.81E-13 | 0.001 | 15 | CDC6, KIF2C, NEK2, SPAG5, BUB1, AURKB, ECT2 |
| 37 | 12 | Cell Cycle Checkpoint | 2.08E-11 | 0.001 | 25 | RFC5, CDC6, MAD2L1, BUB1, TTK, CENPF, BUB1B, CHEK1, BIRC5, CCNA2, CDK2, BRCA1 |
| 37 | 12 | Regulation Of Mitotic Cell Cycle | 3.99E-10 | 0.001 | 25 | CDC6, MAD2L1, NEK2, TPX2, BUB1, TTK, CENPF, BUB1B, CHEK1, BIRC5, CCNA2, CDK2 |
| 37 | 8 | Regulation Of Nuclear Division | 4.18E-10 | 0.001 | 17 | CDC6, MAD2L1, NEK2, BUB1, TTK, CENPF, BUB1B, CHEK1 |
| 37 | 8 | Negative Regulation Of Cell Cycle Process | 7.23E-10 | 0.001 | 17 | CDC6, MAD2L1, BUB1, TTK, CENPF, BUB1B, CHEK1, BRCA1 |
| 37 | 6 | Spindle Checkpoint | 1.21E-09 | 0.001 | 13 | MAD2L1, BUB1, TTK, CENPF, BUB1B, BIRC5 |
| 37 | 6 | Negative Regulation Of Mitosis | 1.51E-09 | 0.001 | 13 | MAD2L1, BUB1, TTK, CENPF, BUB1B, CHEK1 |
| 37 | 7 | Spindle Organization | 5.68E-09 | 0.002 | 15 | KIF23, KIF11, NEK2, SPAG5, TTK, BUB1B, AURKB |
| 37 | 7 | Regulation Of Microtubule Cytoskeleton Organization | 7.88E-09 | 0.002 | 15 | PLK4, NEK2, SPAG5, TPX2, CHEK1, ECT2, BRCA1 |
| 37 | 7 | Organelle Assembly | 1.45E-08 | 0.002 | 15 | KIF23, KIF11, NEK2, CENPA, CENPF, CENPE, AURKB |
| 37 | 5 | Centrosome Cycle | 4.59E-08 | 0.005 | 10 | PLK4, KIF11, NEK2, CDK2, BRCA1 |
| 37 | 8 | Positive Regulation Of Cell Cycle Process | 9.24E-08 | 0.006 | 17 | KIF23, CDC6, PLK4, BIRC5, AURKB, ECT2, CDK2, BRCA1 |
| 37 | 4 | Regulation Of Cell Cycle Cytokinesis | 1.71E-07 | 0.013 | 8.3 | KIF23, CDC6, AURKB, ECT2 |
| 40 | 23 | Cell Cycle Checkpoint | 6.49E-22 | 0.001 | 28 | CDC7, DTL, KNTC1, TTK, SOX4, CENPF, CHEK1, MCM2, MCM3, MCM4, BRCA1, CDK2, CDT1, RFC5, CCNE2, RFC4, MCM7, PLK1, BUB1, BUB1B, WAC, TOP2A, BUB3 |
| 40 | 21 | Dna Replication | 3.26E-21 | 0.001 | 25 | CDC7, SSRP1, DTL, NASP, CENPF, BRCA2, CHEK1, MCM2, MCM3, MCM4, BRCA1, CDK2, CDT1, POLD3, RFC5, RFC4, MCM7, POLD1, CHAF1A, TOP2A, FEN1 |
| 40 | 22 | Dna Repair | 4.48E-17 | 0.001 | 27 | EXO1, SSRP1, DTL, MSH5, TRIM28, BRCA2, CHEK1, RAD54L, SMC3, BRCA1, CDK2, POLD3, RFC5, RFC4, FANCI, POLD1, SFPQ, MUS81, CHAF1A, TOP2A, FEN1, FANCC |
| 40 | 20 | Mitosis | 2.67E-16 | 0.001 | 24 | KIF11, KIF15, TPX2, KNTC1, CENPF, NDC80, NCAPD3, SMC3, CDK2, KIF2C, CDCA8, NCAPH, TIMELESS, PLK1, SPAG5, NCAPG, BUB1, BUB1B, ASPM, BUB3 |
| 40 | 21 | Interphase Of Mitotic Cell Cycle | 7.54E-16 | 0.001 | 25 | CDC7, SKP2, CENPF, CHEK1, MCM2, MCM3, MCM4, CDK2, CDT1, POLD3, RFC5, CCNE2, PLK4, RFC4, MCM7, PLK1, POLD1, TUBG1, TOP2A, FEN1, MELK |
| 40 | 14 | Chromosome Segregation | 5.96E-15 | 0.001 | 17 | MSH5, BRCA2, CENPF, NDC80, NCAPD3, BRCA1, SMC3, KIF2C, NCAPH, NCAPG, SPAG5, BUB1, TOP2A, BUB3 |

| | | | | | | |
|---|---|---|---|---|---|---|
| 40 | 19 | Microtubule-Based Process | 1.68E-14 | 0.001 | 23 | KIF4A, KIF11, KIF5B, PRC1, KIF15, TTK, BRCA2, NDC80, TACC3, BRCA1, CDK2, SMC3, KIF2C, PLK4, PLK1, SPAG5, BUB1B, TUBG1, BUB3 |
| 40 | 9 | Dna Strand Elongation Involved In Dna Replication | 5.15E-13 | 0.001 | 11 | POLD3, RFC5, RFC4, MCM7, POLD1, MCM2, MCM3, MCM4, FEN1 |
| 40 | 12 | S Phase Of Mitotic Cell Cycle | 8.91E-12 | 0.001 | 14 | POLD3, RFC5, RFC4, MCM7, POLD1, SKP2, MCM2, MCM3, MCM4, CDK2, FEN1, CDT1 |
| 40 | 13 | Dna Recombination | 1.32E-11 | 0.001 | 16 | EXO1, MSH5, BRCA2, RAD54L, BRCA1, RFC5, POLD3, RFC4, POLD1, SFPQ, MUS81, TOP2A, FEN1 |
| 40 | 11 | Meiotic Cell Cycle | 8.49E-11 | 0.001 | 13 | EXO1, MKI67, PLK1, MSH5, BRCA2, TUBG1, TOP2A, RAD54L, CDK2, BUB3, SMC3 |
| 40 | 15 | Regulation Of Mitotic Cell Cycle | 3.44E-10 | 0.001 | 18 | TPX2, KNTC1, TTK, BRCA2, SOX4, CENPF, CHEK1, CDK2, CDT1, PLK1, POLD1, BUB1, BUB1B, TOP2A, BUB3 |
| 40 | 9 | Spindle Organization | 4.91E-10 | 0.001 | 11 | KIF11, PRC1, SPAG5, TTK, BUB1B, NDC80, TUBG1, TACC3, SMC3 |
| 40 | 9 | Negative Regulation Of Cell Cycle Process | 3.05E-09 | 0.001 | 11 | BUB1, TTK, CENPF, BUB1B, CHEK1, BUB3, BRCA1, SMC3, CDT1 |
| 40 | 6 | Regulation Of Mitotic Metaphase/Anaphase Transition | 5.39E-08 | 0.005 | 7.2 | PLK1, BUB1, TTK, CENPF, BUB1B, BUB3 |
| 40 | 6 | Centrosome Organization | 3.80E-07 | 0.021 | 7.2 | PLK4, KIF11, PLK1, BRCA2, CDK2, BRCA1 |
| 41 | 15 | Response To Bacterium | 2.60E-11 | 0.001 | 22 | IL6, CEBPB, C5AR1, S100A8, TLR2, SOD2, SLC11A1, NOD2, TNFRSF1B, THBD, CCL20, STAB1, PLA2G2A, TNFAIP3, CD14 |
| 41 | 5 | Response To Peptidoglycan | 1.52E-08 | 0.002 | 7.5 | IL6, NOD2, C5AR1, TLR2, TNFAIP3 |
| 41 | 10 | Regulation Of Inflammatory Response | 1.63E-08 | 0.002 | 15 | IER3, IL6, TNFRSF1B, NOD2, MAPK13, TLR2, PLA2G2A, C2, BIRC3, TNFAIP3 |
| 41 | 8 | Defense Response To Bacterium | 3.24E-08 | 0.004 | 12 | SLC11A1, IL6, NOD2, C5AR1, CCL20, STAB1, TLR2, PLA2G2A |
| 41 | 6 | Regulation Of Interleukin-6 Production | 6.31E-07 | 0.031 | 9 | IL6, NOD2, CEBPB, MAPK13, TLR2, TNFAIP3 |
| 43 | 24 | Mitosis | 1.17E-26 | 0.001 | 44 | KIF23, CENPF, CDC20, BIRC5, NDC80, AURKA, AURKB, PBK, PTTG1, UBE2C, RACGAP1, MLF1IP, CCNB1, KIF2C, SPC25, MAD2L1, CCNB2, CENPA, OIP5, NCAPG, BUB1B, FBXO5, ZWILCH, ASPM |
| 43 | 13 | Mitotic Prometaphase | 4.35E-18 | 0.001 | 24 | CCNB1, KIF2C, SPC25, MAD2L1, CENPA, BUB1B, CENPF, CDC20, NDC80, BIRC5, AURKB, ZWILCH, MLF1IP |
| 43 | 11 | Spindle Organization | 3.50E-15 | 0.001 | 20 | KIF23, SPC25, CKS2, FBXO5, TTK, BUB1B, AURKA, NDC80, AURKB, RACGAP1, UBE2C |
| 43 | 17 | Interphase Of Mitotic Cell Cycle | 8.31E-15 | 0.001 | 31 | GINS1, CKS1B, GINS2, DBF4, CENPF, BIRC5, MCM2, CDKN3, CCNB1, TYMS, RFC4, CCNB2, PCNA, FBXO5, KPNA2, TOP2A, MELK |
| 43 | 15 | Cell Cycle Checkpoint | 1.07E-14 | 0.001 | 28 | DTL, DBF4, TTK, CENPF, BIRC5, CDC20, MCM2, UBE2C, CCNB1, MAD2L1, CCNB2, RFC4, BUB1B, ZWILCH, TOP2A |
| 43 | 8 | Spindle Checkpoint | 3.47E-13 | 0.001 | 15 | CCNB1, MAD2L1, TTK, CENPF, BUB1B, BIRC5, CDC20, UBE2C |

| | | | | | | |
|---|---|---|---|---|---|---|
| 43 | 15 | Microtubule-Based Process | 4.79E-13 | 0.001 | 28 | KIF23, KIF4A, TTK, NDC80, AURKA, AURKB, UBE2C, RACGAP1, SPC25, KIF2C, CENPA, CKS2, BUB1B, FBXO5, KPNA2 |
| 43 | 11 | Chromosome Segregation | 6.27E-13 | 0.001 | 20 | CCNB1, KIF2C, SPC25, MAD2L1, NCAPG, CENPF, NDC80, BIRC5, PTTG1, AURKB, TOP2A |
| 43 | 12 | Dna Replication | 7.08E-12 | 0.001 | 22 | GINS1, GINS2, TYMS, RFC4, DTL, DBF4, PCNA, CENPF, MCM2, RNASEH2A, TOP2A, RAD51 |
| 43 | 7 | Negative Regulation Of Mitotic Metaphase/Anaphase Transition | 9.62E-12 | 0.001 | 13 | MAD2L1, FBXO5, TTK, CENPF, BUB1B, CDC20, UBE2C |
| 43 | 9 | Anaphase-Promoting Complex-Dependent Proteasomal Ubiquitin-Dependent Protein Catabolic Process | 3.17E-11 | 0.001 | 17 | CCNB1, MAD2L1, FBXO5, BUB1B, AURKA, CDC20, PTTG1, AURKB, UBE2C |
| 43 | 9 | Inositol Lipid-Mediated Signaling | 5.22E-11 | 0.001 | 17 | TYMS, RFC4, CKS2, PCNA, BUB1B, AURKA, NDC80, UBE2C, TOP2A |
| 43 | 12 | Regulation Of Mitotic Cell Cycle | 1.72E-09 | 0.001 | 22 | CCNB1, MAD2L1, FBXO5, TTK, CENPF, BUB1B, AURKA, BIRC5, CDC20, ZWILCH, UBE2C, TOP2A |
| 43 | 5 | Regulation Of Chromosome Segregation | 1.41E-08 | 0.002 | 9.3 | CCNB1, KIF2C, AURKB, RACGAP1, ECT2 |
| 43 | 7 | Protein-Dna Complex Assembly | 1.28E-07 | 0.009 | 13 | CENPA, OIP5, H2AFZ, CENPF, MCM2, MLF1IP, RAD51 |
| 43 | 5 | Dna Strand Elongation Involved In Dna Replication | 2.67E-07 | 0.018 | 9.3 | GINS1, GINS2, RFC4, PCNA, MCM2 |
| 43 | 4 | Regulation Of Cell Cycle Cytokinesis | 2.79E-07 | 0.018 | 7.4 | KIF23, AURKB, RACGAP1, ECT2 |
| 43 | 8 | Dna Conformation Change | 4.01E-07 | 0.021 | 15 | NCAPG, CENPA, OIP5, H2AFZ, MCM2, TOP2A, MLF1IP, RAD51 |
| 43 | 6 | Organelle Assembly | 8.69E-07 | 0.04 | 11 | KIF23, CENPA, FBXO5, CENPF, AURKB, RACGAP1 |
| 46 | 10 | Rna Splicing | 3.30E-07 | 0.019 | 17 | FUS, RBM4B, PRPF4B, USP39, PCBP2, RBM4, WBP11, DHX35, CSTF1, SRPK1 |
| 49 | 14 | Immune Response-Regulating Signaling Pathway | 9.25E-16 | 0.001 | 35 | FYB, PTPRC, HCK, TLR1, TLR2, TLR5, TLR7, HLA-DMA, LILRB1, CD86, MNDA, CD4, LCP2, SYK |
| 49 | 7 | Regulation Of Cytokine Biosynthetic Process | 7.47E-09 | 0.002 | 18 | LILRB1, CD86, TLR1, TLR2, CD4, TLR7, SYK |
| 49 | 9 | Immune Effector Process | 8.38E-09 | 0.002 | 23 | LILRB1, PTPRC, CD86, HCK, TLR1, MYO1F, HLA-DMA, TLR7, SYK |
| 49 | 10 | Leukocyte Activation | 1.81E-08 | 0.002 | 25 | LILRB1, PTPRC, CD86, TLR1, MYO1F, CD4, HLA-DMA, TLR7, SYK, LCP2 |
| 49 | 9 | Regulation Of Lymphocyte Activation | 9.45E-08 | 0.006 | 23 | LILRB1, PTPRC, CD86, IL18, MNDA, CD4, SAMSN1, HLA-DMA, SYK |
| 49 | 4 | Positive Regulation Of Interleukin-8 Production | 1.55E-07 | 0.01 | 10 | IL18, TLR2, TLR5, TLR7 |
| 49 | 7 | Regulation Of Peptidyl-Tyrosine Phosphorylation | 2.74E-07 | 0.018 | 18 | PTPRC, HCLS1, ITGB2, CD4, SAMSN1, SYK, CSF1R |
| 49 | 6 | Defense Response To Bacterium | 4.61E-07 | 0.024 | 15 | HCK, TLR1, TLR2, MYO1F, CD4, SYK |

| | | | | | | |
|---|---|---|---|---|---|---|
| 49 | 6 | Cellular Response To Biotic Stimulus | 5.65E-07 | 0.028 | 15 | LILRB1, HCK, IL18, TLR1, TLR2, SYK |
| 49 | 5 | Regulation Of Alpha-Beta T Cell Activation | 7.39E-07 | 0.037 | 13 | LILRB1, PTPRC, CD86, IL18, SYK |
| 49 | 4 | T Cell Selection | 8.13E-07 | 0.038 | 10 | PTPRC, CD4, HLA-DMA, SYK |
| 51 | 24 | Activation Of Immune Response | 4.42E-22 | 0.001 | 27 | FYB, PTPRC, C3AR1, LYN, LY96, HCK, TLR1, TLR2, NCKAP1L, HLA-DMB, TLR7, HLA-DQA1, C1QA, C1QB, CD86, RPS6KA1, MNDA, FCER1G, CD4, HLA-DPB1, VSIG4, CD14, LCP2, SYK |
| 51 | 22 | Immune Response-Regulating Signaling Pathway | 1.63E-20 | 0.001 | 25 | FYB, PTPRC, C3AR1, LYN, LY96, HCK, TLR1, TLR2, NCKAP1L, HLA-DMB, TLR7, HLA-DQA1, LILRB1, CD86, RPS6KA1, MNDA, FCER1G, CD4, HLA-DPB1, CD14, LCP2, SYK |
| 51 | 19 | Regulation Of Leukocyte Activation | 1.43E-13 | 0.001 | 22 | PTPRC, LYN, AIF1, IL18, NCKAP1L, HLA-DMB, SAMSN1, HLA-DQA1, LILRB1, CD86, CORO1A, HMOX1, MNDA, FCER1G, CD4, HLA-DPB1, VSIG4, SASH3, SYK |
| 51 | 17 | Positive Regulation Of Cell Activation | 1.90E-13 | 0.001 | 19 | PTPRC, LYN, PLEK, AIF1, IL18, NCKAP1L, HLA-DMB, HLA-DQA1, LILRB1, CD86, CORO1A, RPS6KA1, FCER1G, CD4, HLA-DPB1, SASH3, SYK |
| 51 | 15 | Immune Effector Process | 5.50E-12 | 0.001 | 17 | PIK3CG, PTPRC, LYN, HCK, TLR1, MYO1F, APOBEC3G, TLR7, LILRB1, C1QA, C1QB, CD86, FCER1G, VSIG4, SYK |
| 51 | 17 | Response To Bacterium | 1.50E-11 | 0.001 | 19 | TSPO, LYN, NCF2, RNASE3, LY96, HCK, IL18, TLR1, LYZ, TLR2, MYO1F, LILRB1, FCER1G, SERPINA1, CD4, CD14, SYK |
| 51 | 7 | Myeloid Cell Activation Involved In Immune Response | 6.15E-10 | 0.001 | 8 | PIK3CG, LYN, TLR1, MYO1F, FCER1G, TLR7, SYK |
| 51 | 9 | Myeloid Leukocyte Activation | 6.20E-10 | 0.001 | 10 | PIK3CG, LYN, AIF1, TLR1, MYO1F, FCER1G, TLR7, SYK, LCP2 |
| 51 | 13 | Regulation Of Immune Effector Process | 6.21E-10 | 0.001 | 15 | PTPRC, LYN, HCK, TLR2, APOBEC3G, LILRB1, CD86, DOCK2, HMOX1, FCER1G, CD4, SASH3, SYK |
| 51 | 10 | Cellular Response To Biotic Stimulus | 1.29E-09 | 0.001 | 11 | LILRB1, TSPO, LYN, LY96, HCK, IL18, TLR1, TLR2, CD14, SYK |
| 51 | 14 | Leukocyte Activation | 1.32E-08 | 0.002 | 16 | PIK3CG, LILRB1, PTPRC, DOCK2, CD86, LYN, AIF1, TLR1, FCER1G, MYO1F, CD4, TLR7, SYK, LCP2 |
| 51 | 8 | Regulation Of Tumor Necrosis Factor Production | 1.33E-08 | 0.002 | 9.1 | LILRB1, TSPO, IL18, TLR1, TLR2, FCER1G, SASH3, CD14 |
| 51 | 9 | Defense Response To Bacterium | 1.70E-08 | 0.002 | 10 | RNASE3, HCK, TLR1, LYZ, TLR2, MYO1F, FCER1G, CD4, SYK |
| 51 | 8 | Regulation Of Cytokine Secretion | 1.73E-08 | 0.002 | 9.1 | LILRB1, LYN, TLR2, PYCARD, CD14, SRGN, SYK, CSF1R |
| 51 | 10 | Antigen Processing And Presentation | 3.05E-08 | 0.003 | 11 | CYBA, CYBB, NCF2, FCGR1A, NCF4, FCER1G, CTSS, HLA-DPB1, HLA-DMB, HLA-DQA1 |
| 51 | 5 | Respiratory Burst | 4.04E-08 | 0.005 | 5.7 | PIK3CG, CYBA, CYBB, NCF2, HCK |
| 51 | 11 | Exocytosis | 8.79E-08 | 0.006 | 13 | PIK3CG, PLEK, LYN, HCK, CCR1, SCIN, MYO1F, FCER1G, SERPINA1, SRGN, SYK |
| 51 | 7 | Regulation Of Alpha-Beta T Cell Activation | 9.12E-08 | 0.006 | 8 | LILRB1, PTPRC, CD86, IL18, NCKAP1L, SASH3, SYK |
| 51 | 7 | Positive Regulation Of Cytokine Biosynthetic Process | 9.12E-08 | 0.006 | 8 | CD86, HMOX1, TLR1, TLR2, CD4, TLR7, SYK |

| | | | | | | |
|---|---|---|---|---|---|---|
| 51 | 4 | Positive Regulation Of Gamma-Delta T Cell Activation | 3.66E-07 | 0.021 | 4.5 | LILRB1, PTPRC, NCKAP1L, SYK |
| 51 | 9 | Positive Regulation Of Response To External Stimulus | 6.33E-07 | 0.031 | 10 | PIK3CG, C3AR1, AIF1, CCR1, IL18, TLR2, FCER1G, NCKAP1L, TLR7 |
| 51 | 8 | Adaptive Immune Response | 6.81E-07 | 0.034 | 9.1 | PIK3CG, C1QA, C1QB, C3AR1, IL18, FCER1G, CTSS, SYK |
| 51 | 7 | Leukocyte Chemotaxis | 8.26E-07 | 0.038 | 8 | PIK3CG, CORO1A, CCR1, FCER1G, NCKAP1L, ITGB2, SYK |
| 51 | 10 | Leukocyte Migration | 8.36E-07 | 0.039 | 11 | PIK3CG, CORO1A, LYN, HCK, CCR1, FCER1G, NCKAP1L, ITGB2, SYK, SLC7A7 |
