# Supplementary material for: A Computational Approach to Identifying Gene-microRNA Modules in Cancer
Source: PLoS Comput Biol. 2015 Jan 22;11(1):e1004042. doi: 10.1371/journal.pcbi.1004042 (PMC4303261; doi:10.1371/journal.pcbi.1004042)
Supplement: S25 Table — (PDF) [file pcbi.1004042.s032.pdf]

Table S25. Ovarian cancer specific genes and GBM specific genes in identified modules.

Module ID	Ovarian cancer specific genes
1	PTK2
5	PTK2
6	BARD1
7	PTK2
8	FN1
12	NBN
13	CDC42
14	MAP3K4
15	INSR
17	C11orf30
19	MAP3K3
20	MAD2L1,CDC20,AURKA
22	FN1
23	ARID4B
26	MAD2L1
27	SERPINF1,FN1
28	HMGA1
31	RAB25,SERPINF1,CTSD,ACVR2B
32	RAB25
33	AURKA,CDC20

Module ID	GBM specific genes
3	TSC2
8	PLCG2
22	RPS6KA1,PLCG2
26	HIPK2,SHC2
30	PLCG2
32	PDPK1
33	PDGFA
39	HIPK2
44	TSC2
46	TSC2
51	RPS6KA1
