# Supplementary material for: A Computational Approach to Identifying Gene-microRNA Modules in Cancer
Source: PLoS Comput Biol. 2015 Jan 22;11(1):e1004042. doi: 10.1371/journal.pcbi.1004042 (PMC4303261; doi:10.1371/journal.pcbi.1004042)
Supplement: S26 Table — (PDF) [file pcbi.1004042.s033.pdf]

Table S26. DNA copy number aberration regions that regulate gene expressions in ovarian cancer modules.

Module ID	DNA regions with copy number aberrations (CNAs) and PCC values between CNAs and genes
1	chr6:159800000-160099999(-0.201), chr6:160700000-160799999(-0.202), chr6:161100000-161199999(-0.201), chr6:161600000-163099999(-0.208), chr6:163200000-163299999(-0.204), chr6:163800000-165899999(-0.209), chr6:166100000-166599999(-0.209), chr6:166700000-166899999(-0.207), chr6:167300000-167499999(-0.209), chr6:167600000-168199999(-0.205), chr6:168400000-170999999(-0.224), chr8:75000000-145299999(0.401)
4	chr19:55800000-55999999(-0.201)
5	chr8:102300000-145299999(0.289)
7	chr8:76600000-76799999(0.201), chr8:77000000-77999999(0.205), chr8:78200000-78299999(0.203), chr8:78400000-78499999(0.202), chr8:78700000-78999999(0.201), chr8:79100000-79399999(0.201), chr8:80900000-80999999(0.203), chr8:82400000-82699999(0.202), chr8:83400000-83499999(0.201), chr8:83900000-84099999(0.202), chr8:84200000-84299999(0.2), chr8:84800000-84899999(0.2), chr8:87100000-145299999(0.365)
9	chr1:32100000-53399999(0.301), chr1:53600000-53699999(0.2), chr1:54100000-54199999(0.2), chr1:54300000-54599999(0.206), chr1:54800000-54899999(0.204), chr1:55000000-55099999(0.201)
10	chr12:800000-39599999(0.357), chr12:40400000-40499999(0.201), chr12:40700000-40799999(0.21), chr12:41600000-41699999(0.2)
11	chr16:600000-14699999(0.292), chr16:14800000-18799999(0.225)
12	chr6:1000000-1699999(0.204), chr6:2000000-26199999(0.231), chr6:26300000-27699999(0.208), chr6:28000000-28399999(0.202), chr6:28600000-29399999(0.205), chr6:29900000-30399999(0.204), chr6:31300000-32999999(0.206), chr6:33600000-34499999(0.205), chr6:35600000-35699999(0.204)
16	chr19:8200000-22299999(0.377), chr19:22500000-22799999(0.208), chr19:22900000-23199999(0.203)
17	chr12:39200000-39499999(0.204), chr12:40100000-40699999(0.213), chr12:41100000-43299999(0.212), chr12:47400000-47599999(0.207), chr12:47700000-73199999(0.264), chr12:73600000-73699999(0.201), chr12:73900000-74299999(0.203), chr12:74400000-74699999(0.202)
21	chr2:230300000-230399999(-0.201), chr4:156900000-156999999(0.203), chr4:157300000-159299999(0.206), chr4:159400000-159599999(0.205), chr22:40500000-41699999(0.213), chr22:45500000-45599999(0.202)
23	chr1:158200000-159599999(0.207), chr1:160300000-160799999(0.203), chr1:160900000-160999999(0.201), chr1:161500000-162199999(0.206), chr1:162500000-162699999(0.202), chr1:162800000-169299999(0.219), chr1:169600000-169899999(0.203), chr1:170000000-171299999(0.207), chr1:171600000-171799999(0.2), chr1:172200000-180399999(0.217), chr1:180600000-247899999(0.319)
28	chr19:8600000-8699999(0.203), chr19:8800000-21199999(0.263)
29	chr1:31900000-59099999(0.345), chr1:60000000-61899999(0.209), chr1:62000000-62299999(0.203), chr1:62700000-63499999(0.207)
30	chr6:1000000-70199999(0.366), chr6:70700000-71799999(0.206), chr6:72400000-72599999(0.201), chr6:73000000-73799999(0.207), chr6:74100000-74199999(0.201)
