# Supplementary material for: A Computational Approach to Identifying Gene-microRNA Modules in Cancer
Source: PLoS Comput Biol. 2015 Jan 22;11(1):e1004042. doi: 10.1371/journal.pcbi.1004042 (PMC4303261; doi:10.1371/journal.pcbi.1004042)
Supplement: S27 Table — (PDF) [file pcbi.1004042.s034.pdf]

Table S27. DNA copy number aberration regions that regulate gene expressions in GBM modules.

Module ID	DNA regions with copy number aberrations (CNAs) and PCC values between CNAs and genes
1	chr10:500000-1399999(0.211), chr10:1700000-1899999(0.205), chr10:2000000-2099999(0.205), chr10:2400000-3099999(0.209), chr10:3200000-3299999(0.219), chr10:3400000-3499999(0.209), chr10:3600000-3699999(0.204), chr10:3800000-3999999(0.203), chr10:4100000-6299999(0.214), chr10:6400000-6999999(0.219), chr10:7100000-7499999(0.218), chr10:7600000-8599999(0.219), chr10:8700000-11399999(0.213), chr10:11500000-12499999(0.215), chr10:12600000-12799999(0.205), chr10:12900000-13899999(0.212), chr10:14000000-14499999(0.213), chr10:14600000-14799999(0.202), chr10:14900000-15099999(0.211), chr10:15200000-16399999(0.208), chr10:16900000-17499999(0.203), chr10:17600000-18199999(0.203), chr10:22600000-22699999(0.2), chr10:29500000-29599999(0.201), chr11:1300000-1399999(-0.203), chr11:1900000-1999999(-0.204), chr11:6200000-6299999(-0.205), chr11:6600000-6699999(-0.201)
2	chr1:150000000-150199999(0.204), chr1:157100000-157199999(0.202), chr1:158000000-158399999(0.209), chr1:158500000-159199999(0.214), chr1:161500000-161899999(0.216), chr1:162000000-162799999(0.216), chr1:162900000-164599999(0.215), chr1:164700000-165199999(0.217), chr1:165300000-165499999(0.217), chr1:165600000-166199999(0.21), chr1:166300000-166999999(0.223), chr1:167100000-167599999(0.215), chr1:167700000-168099999(0.211), chr1:168200000-169899999(0.21), chr1:170000000-170399999(0.211), chr1:170500000-170699999(0.204), chr1:170800000-171399999(0.206), chr1:171500000-172999999(0.213), chr1:173200000-177599999(0.21), chr1:177700000-178999999(0.208), chr1:179100000-179299999(0.211), chr1:179400000-179499999(0.209), chr1:179800000-179999999(0.204), chr1:180100000-180399999(0.203), chr1:180600000-180699999(0.201), chr1:181000000-181099999(0.213), chr1:181200000-182199999(0.214), chr1:182300000-182399999(0.212), chr1:182600000-183299999(0.209), chr1:183400000-184899999(0.205), chr1:185000000-185199999(0.203), chr1:193800000-193899999(0.201)
4	chr7:55100000-55199999(0.201)
5	chr1:150000000-150199999(0.206), chr1:157100000-157199999(0.208), chr1:157600000-158399999(0.208), chr1:158500000-159199999(0.218), chr1:159300000-159499999(0.204), chr1:159700000-159799999(0.203), chr1:160900000-160999999(0.204), chr1:161100000-161899999(0.213), chr1:162000000-162799999(0.221), chr1:162900000-164599999(0.22), chr1:164700000-165199999(0.223), chr1:165300000-165499999(0.223), chr1:165600000-166199999(0.214), chr1:166300000-166999999(0.226), chr1:167100000-168099999(0.215), chr1:168200000-169799999(0.214), chr1:170000000-170399999(0.212), chr1:170500000-170699999(0.205), chr1:170800000-171399999(0.207), chr1:171500000-172999999(0.215), chr1:173100000-177599999(0.211), chr1:177700000-178999999(0.211), chr1:179100000-179699999(0.209), chr1:179800000-179999999(0.209), chr1:180100000-180399999(0.209), chr1:180600000-180799999(0.205), chr1:181000000-181099999(0.216), chr1:181200000-182199999(0.218), chr1:182300000-182399999(0.217), chr1:182500000-184899999(0.21), chr1:185000000-185199999(0.206), chr1:193800000-193899999(0.205), chr1:194600000-194699999(0.205), chr10:104700000-104799999(-0.203), chr10:106100000-106199999(-0.208), chr10:110700000-110799999(-0.2), chr10:114800000-114899999(-0.203), chr10:120000000-120099999(-0.209), chr10:122400000-122599999(-0.203), chr10:123800000-123999999(-0.203), chr10:124600000-124799999(-0.209), chr10:131600000-131699999(-0.2), chr10:134000000-134099999(-0.202), chr11:6200000-6299999(-0.203)

7	chr10:76300000-77099999(-0.205), chr10:77200000-78599999(-0.203), chr10:78700000-79399999(-0.205), chr10:82600000-82699999(-0.201), chr10:83500000-83799999(-0.203), chr10:84000000-84699999(-0.207), chr10:84800000-85499999(-0.207), chr10:85800000-85899999(-0.205), chr10:86000000-86999999(-0.205), chr10:87100000-87199999(-0.203), chr10:87300000-87999999(-0.216), chr10:88100000-88399999(-0.212), chr10:89100000-89199999(-0.202), chr10:91600000-91799999(-0.202), chr10:91900000-92399999(-0.208), chr10:92500000-92699999(-0.21), chr10:92800000-92899999(-0.205), chr10:93000000-93599999(-0.214), chr10:93700000-95299999(-0.213), chr10:95400000-97299999(-0.219), chr10:97400000-98899999(-0.22), chr10:99000000-99299999(-0.21), chr10:99400000-99599999(-0.204), chr10:99700000-100199999(-0.21), chr10:100300000-101199999(-0.214), chr10:101300000-101599999(-0.213), chr10:101700000-102499999(-0.215), chr10:102600000-102899999(-0.213), chr10:103000000-103299999(-0.215), chr10:103500000-108199999(-0.222), chr10:108500000-109099999(-0.215), chr10:109300000-109399999(-0.227), chr10:109800000-110799999(-0.22), chr10:110900000-110999999(-0.223), chr10:111100000-111499999(-0.218), chr10:111600000-111999999(-0.206), chr10:112100000-112199999(-0.207), chr10:112300000-112399999(-0.218), chr10:112600000-112699999(-0.21), chr10:112900000-112999999(-0.209), chr10:113100000-113299999(-0.206), chr10:113400000-113499999(-0.212), chr10:113600000-113899999(-0.211), chr10:114100000-114399999(-0.212), chr10:114500000-117599999(-0.223), chr10:117700000-120999999(-0.226), chr10:121300000-121799999(-0.22), chr10:121900000-122899999(-0.225), chr10:123400000-124399999(-0.23), chr10:124500000-125899999(-0.214), chr10:126400000-126499999(-0.21), chr10:126800000-126999999(-0.208), chr10:127100000-127599999(-0.219), chr10:127800000-127899999(-0.222), chr10:128000000-128599999(-0.218), chr10:128900000-128999999(-0.225), chr10:129200000-129399999(-0.214), chr10:129600000-129799999(-0.204), chr10:129900000-130199999(-0.206), chr10:130400000-131199999(-0.214), chr10:131300000-131499999(-0.22), chr10:131600000-131999999(-0.223), chr10:132100000-134099999(-0.221), chr10:134300000-135299999(-0.22)
9	chr4:55100000-55199999(0.203)
13	chr10:95600000-95699999(-0.201), chr10:96000000-96099999(-0.201), chr10:96200000-96499999(-0.201), chr10:96600000-96699999(-0.204), chr10:97800000-97899999(-0.201), chr10:98300000-98799999(-0.201), chr10:103600000-104399999(-0.206), chr10:104500000-106099999(-0.207), chr10:106200000-106299999(-0.2), chr10:106700000-106799999(-0.202), chr10:106900000-107699999(-0.204), chr10:107800000-108199999(-0.205), chr10:109300000-109399999(-0.208), chr10:109800000-109899999(-0.204), chr10:110200000-110299999(-0.2), chr10:110400000-110799999(-0.206), chr10:110900000-110999999(-0.203), chr10:111100000-111199999(-0.207), chr10:111300000-111399999(-0.2), chr10:114700000-115099999(-0.207), chr10:115200000-115299999(-0.204), chr10:115600000-116099999(-0.205), chr10:116200000-116299999(-0.203), chr10:116400000-116499999(-0.201), chr10:116800000-117199999(-0.206), chr10:117300000-117599999(-0.207), chr10:117900000-118899999(-0.211), chr10:119000000-119099999(-0.217), chr10:119300000-119399999(-0.208), chr10:119500000-119599999(-0.204), chr10:119700000-120199999(-0.208), chr10:120300000-120499999(-0.211), chr10:120600000-120999999(-0.209), chr10:121300000-121399999(-0.206), chr10:121500000-121799999(-0.208), chr10:121900000-122099999(-0.205), chr10:122200000-122899999(-0.213), chr10:123400000-124399999(-0.211), chr10:124500000-124799999(-0.209), chr10:127400000-127599999(-0.205), chr10:127800000-127899999(-0.205), chr10:128000000-128199999(-0.201), chr10:128300000-128499999(-0.208), chr10:128900000-128999999(-0.208), chr10:130600000-130699999(-0.201), chr10:130900000-130999999(-0.209), chr10:131100000-131199999(-0.209), chr10:131300000-131499999(-0.203), chr10:131600000-131999999(-0.204), chr10:132100000-132999999(-0.204), chr10:133100000-133799999(-0.205), chr10:134000000-134099999(-0.203), chr10:134300000-134599999(-0.203), chr10:135100000-135199999(-0.202)
16	chr1:150000000-150199999(0.212), chr1:157100000-157199999(0.201), chr1:157700000-157799999(0.2), chr1:157900000-158399999(0.21), chr1:158500000-159199999(0.218), chr1:159300000-159499999(0.207), chr1:159600000-159799999(0.204), chr1:160900000-160999999(0.207), chr1:161100000-161899999(0.215), chr1:162000000-162799999(0.222), chr1:162900000-164599999(0.222), chr1:164700000-165199999(0.224), chr1:165300000-165499999(0.225), chr1:165600000-166199999(0.218), chr1:166300000-166999999(0.227), chr1:167100000-168099999(0.216), chr1:168200000-169899999(0.218), chr1:170000000-177599999(0.217), chr1:177700000-178999999(0.218), chr1:179100000-179699999(0.215), chr1:179800000-179999999(0.214), chr1:180100000-180499999(0.21), chr1:180600000-180799999(0.211), chr1:181000000-182199999(0.219), chr1:182300000-184899999(0.212), chr1:185000000-185299999(0.207), chr1:193800000-193899999(0.204), chr1:194600000-194699999(0.204), chr11:6200000-6299999(-0.202)

27	chr1:62700000-62799999(0.203), chr1:150000000-150199999(0.222), chr1:153000000-153199999(0.209), chr1:154000000-154299999(0.208), chr1:155900000-156299999(0.208), chr1:157100000-157499999(0.216), chr1:157600000-158399999(0.227), chr1:158500000-159199999(0.242), chr1:159300000-159799999(0.223), chr1:160200000-160799999(0.212), chr1:160900000-160999999(0.229), chr1:161100000-161899999(0.237), chr1:162000000-162799999(0.245), chr1:162900000-164599999(0.242), chr1:164700000-165199999(0.245), chr1:165300000-165499999(0.247), chr1:165600000-166999999(0.241), chr1:167100000-169899999(0.236), chr1:170000000-178999999(0.236), chr1:179100000-179999999(0.232), chr1:180100000-180799999(0.227), chr1:180900000-184899999(0.231), chr1:185000000-186399999(0.21), chr1:186500000-187199999(0.205), chr1:187600000-187699999(0.203), chr1:192600000-192699999(0.201), chr1:193200000-193899999(0.21), chr1:194000000-195499999(0.209), chr1:195600000-195699999(0.203), chr1:195800000-195999999(0.206), chr10:76500000-77099999(-0.203), chr10:77200000-77399999(-0.203), chr10:77700000-77799999(-0.202), chr10:79000000-79099999(-0.202), chr10:79300000-79399999(-0.204), chr10:84000000-84099999(-0.21), chr10:84900000-84999999(-0.217), chr10:85400000-85599999(-0.201), chr10:87400000-87999999(-0.21), chr10:88100000-88399999(-0.203), chr10:89300000-89399999(-0.208), chr10:92500000-92699999(-0.201), chr10:92900000-93099999(-0.201), chr10:93300000-93399999(-0.219), chr10:93500000-93599999(-0.203), chr10:94500000-95299999(-0.207), chr10:95400000-96799999(-0.213), chr10:96900000-96999999(-0.204), chr10:97100000-97199999(-0.2), chr10:97400000-98899999(-0.207), chr10:100500000-100599999(-0.202), chr10:100800000-100999999(-0.206), chr10:101800000-101999999(-0.201), chr10:102100000-102199999(-0.211), chr10:102300000-102399999(-0.2), chr10:102600000-102699999(-0.201), chr10:103000000-103299999(-0.201), chr10:103500000-106199999(-0.215), chr10:106400000-108199999(-0.211), chr10:108500000-109099999(-0.206), chr10:109300000-109399999(-0.22), chr10:109800000-110099999(-0.211), chr10:110200000-110799999(-0.214), chr10:110900000-111399999(-0.21), chr10:112100000-112199999(-0.202), chr10:112300000-112399999(-0.209), chr10:113400000-113499999(-0.2), chr10:113600000-113899999(-0.204), chr10:114100000-114399999(-0.203), chr10:114600000-117599999(-0.214), chr10:117700000-120499999(-0.216), chr10:120600000-120899999(-0.22), chr10:121400000-121699999(-0.214), chr10:122000000-122799999(-0.218), chr10:123400000-124399999(-0.218), chr10:124500000-124899999(-0.223), chr10:127300000-127599999(-0.209), chr10:127800000-127899999(-0.211), chr10:128000000-128499999(-0.21), chr10:128900000-128999999(-0.213), chr10:129200000-129399999(-0.209), chr10:130600000-130999999(-0.206), chr10:131100000-131199999(-0.212), chr10:131300000-131499999(-0.208), chr10:131600000-131999999(-0.211), chr10:132100000-133799999(-0.209), chr10:133900000-134099999(-0.217), chr10:134300000-135099999(-0.21), chr13:49100000-49199999(-0.205)

29	chr1:150000000-150199999(0.216), chr1:153100000-153199999(0.206), chr1:154000000-154299999(0.203), chr1:155900000-156299999(0.205), chr1:157100000-157499999(0.209), chr1:157600000-158399999(0.221), chr1:158500000-159199999(0.233), chr1:159300000-159499999(0.221), chr1:159600000-159799999(0.219), chr1:160500000-160799999(0.21), chr1:160900000-160999999(0.22), chr1:161100000-161899999(0.228), chr1:162000000-162799999(0.236), chr1:162900000-164599999(0.233), chr1:164700000-165199999(0.236), chr1:165300000-165499999(0.238), chr1:165600000-166999999(0.233), chr1:167100000-169899999(0.231), chr1:170000000-178999999(0.23), chr1:179100000-179999999(0.224), chr1:180100000-180799999(0.22), chr1:180900000-182199999(0.23), chr1:182300000-184899999(0.223), chr1:185000000-185499999(0.212), chr1:185800000-185899999(0.2), chr1:193200000-193899999(0.205), chr1:194000000-195499999(0.205), chr1:195800000-195899999(0.206), chr10:76400000-77099999(-0.205), chr10:77200000-77399999(-0.203), chr10:79000000-79099999(-0.201), chr10:79300000-79399999(-0.207), chr10:84000000-84099999(-0.212), chr10:84300000-84699999(-0.202), chr10:84800000-85199999(-0.203), chr10:85300000-85599999(-0.202), chr10:85800000-85899999(-0.2), chr10:86000000-86299999(-0.201), chr10:86400000-86499999(-0.203), chr10:87300000-87999999(-0.213), chr10:88100000-88399999(-0.208), chr10:89300000-89399999(-0.211), chr10:92000000-92399999(-0.205), chr10:92500000-93599999(-0.208), chr10:93700000-95299999(-0.209), chr10:95400000-96999999(-0.217), chr10:97100000-97199999(-0.2), chr10:97400000-98899999(-0.214), chr10:99000000-99299999(-0.203), chr10:99700000-100199999(-0.202), chr10:100400000-101199999(-0.207), chr10:101300000-101599999(-0.207), chr10:101700000-102499999(-0.209), chr10:102600000-102899999(-0.206), chr10:103000000-103299999(-0.21), chr10:103500000-108199999(-0.221), chr10:108400000-109099999(-0.213), chr10:109200000-109399999(-0.214), chr10:109500000-109599999(-0.203), chr10:109700000-110799999(-0.219), chr10:110900000-111499999(-0.216), chr10:111600000-111999999(-0.207), chr10:112100000-112199999(-0.209), chr10:112300000-112399999(-0.217), chr10:112600000-112699999(-0.208), chr10:112900000-112999999(-0.205), chr10:113100000-113199999(-0.205), chr10:113400000-113499999(-0.209), chr10:113600000-113899999(-0.213), chr10:114100000-114399999(-0.211), chr10:114500000-117599999(-0.223), chr10:117700000-120899999(-0.226), chr10:121300000-121799999(-0.215), chr10:121900000-122799999(-0.226), chr10:123400000-124399999(-0.228), chr10:124500000-125399999(-0.219), chr10:125600000-125699999(-0.201), chr10:125800000-125899999(-0.201), chr10:126200000-126299999(-0.201), chr10:126400000-126499999(-0.207), chr10:126800000-126899999(-0.21), chr10:127100000-127599999(-0.215), chr10:127800000-127899999(-0.218), chr10:128000000-128599999(-0.215), chr10:128900000-128999999(-0.221), chr10:129200000-129399999(-0.216), chr10:129600000-129699999(-0.204), chr10:129900000-129999999(-0.207), chr10:130100000-130199999(-0.206), chr10:130500000-130999999(-0.212), chr10:131100000-131199999(-0.223), chr10:131300000-131499999(-0.218), chr10:131600000-131999999(-0.221), chr10:132100000-133799999(-0.218), chr10:133900000-134099999(-0.226), chr10:134300000-135099999(-0.218), chr10:135200000-135299999(-0.204)
30	chr9:21900000-22099999(0.203)
31	chr5:159200000-159299999(0.206), chr5:163000000-163899999(0.204), chr5:164300000-164399999(0.201), chr5:167000000-167299999(0.203)
33	chr5:121400000-121499999(0.202), chr5:132400000-132499999(0.203), chr5:159200000-159299999(0.208), chr7:54900000-55299999(0.217), chr7:75600000-75699999(0.201), chr7:77400000-77499999(0.203), chr7:78200000-78299999(0.215), chr10:500000-899999(-0.205), chr10:1100000-1499999(-0.208), chr10:1600000-3299999(-0.209), chr10:3400000-3499999(-0.204), chr10:4100000-6299999(-0.211), chr10:6400000-6999999(-0.221), chr10:7100000-7399999(-0.22), chr10:7600000-10699999(-0.219), chr10:10800000-11299999(-0.219), chr10:11500000-12799999(-0.217), chr10:12900000-16799999(-0.223), chr10:16900000-18199999(-0.228), chr10:18300000-24399999(-0.232), chr10:24500000-34099999(-0.229), chr10:34300000-34399999(-0.215), chr10:34600000-36699999(-0.209), chr10:36800000-36999999(-0.208), chr10:37100000-37199999(-0.212), chr10:37300000-38699999(-0.208), chr10:46100000-48399999(-0.205), chr10:51600000-52399999(-0.202), chr10:59900000-60099999(-0.201), chr10:60400000-60499999(-0.2), chr10:61000000-61099999(-0.205), chr10:61300000-61799999(-0.206), chr10:62000000-63299999(-0.208), chr10:63500000-64099999(-0.207), chr10:64200000-64499999(-0.206), chr10:64700000-64899999(-0.202), chr10:65000000-65199999(-0.206), chr10:65500000-65699999(-0.203), chr10:66100000-66199999(-0.201), chr10:66400000-66799999(-0.202), chr10:74100000-74599999(-0.2), chr10:74700000-74799999(-0.201), chr10:76200000-76499999(-0.206), chr10:76600000-77099999(-0.205), chr10:77200000-77399999(-0.201), chr11:400000-4399999(0.229), chr11:4500000-7599999(0.227), chr11:7900000-7999999(0.21), chr11:8100000-8199999(0.213), chr11:8300000-10599999(0.214), chr11:10700000-11399999(0.216), chr11:11500000-12099999(0.218), chr11:12200000-12499999(0.217), chr11:12600000-13799999(0.215), chr19:55000000-55099999(0.2), chr19:55300000-55399999(0.201), chr19:55500000-55599999(0.213), chr19:55800000-56599999(0.203), chr19:56700000-58899999(0.207)

37	chr1:150000000-150199999(0.224), chr1:153100000-153199999(0.207), chr1:154000000-154299999(0.206), chr1:155900000-156299999(0.207), chr1:157100000-157499999(0.213), chr1:157600000-158399999(0.224), chr1:158500000-159199999(0.237), chr1:159300000-159499999(0.223), chr1:159600000-159799999(0.22), chr1:160500000-160799999(0.211), chr1:160900000-160999999(0.223), chr1:161100000-161899999(0.232), chr1:162000000-162799999(0.24), chr1:162900000-164599999(0.239), chr1:164700000-165199999(0.241), chr1:165300000-165499999(0.242), chr1:165600000-166199999(0.234), chr1:166300000-169899999(0.234), chr1:170000000-178999999(0.23), chr1:179100000-179999999(0.225), chr1:180100000-180799999(0.221), chr1:180900000-182199999(0.232), chr1:182300000-184899999(0.226), chr1:185000000-186399999(0.206), chr1:186500000-187199999(0.201), chr1:187600000-187699999(0.2), chr1:192600000-192699999(0.2), chr1:193200000-193899999(0.209), chr1:194000000-195499999(0.208), chr1:195600000-195699999(0.201), chr1:195800000-195999999(0.206), chr10:93300000-93399999(-0.2), chr10:104700000-104799999(-0.208), chr10:106000000-106199999(-0.207), chr10:109300000-109399999(-0.2), chr10:110200000-110299999(-0.202), chr10:110700000-110799999(-0.206), chr10:114800000-115099999(-0.203), chr10:115200000-115499999(-0.205), chr10:115600000-115699999(-0.207), chr10:117300000-117399999(-0.201), chr10:117900000-118199999(-0.204), chr10:118400000-118899999(-0.205), chr10:119000000-119099999(-0.21), chr10:119300000-119399999(-0.2), chr10:119900000-120199999(-0.206), chr10:120300000-120499999(-0.2), chr10:120700000-120899999(-0.202), chr10:121500000-121599999(-0.2), chr10:122200000-122799999(-0.203), chr10:123700000-124099999(-0.205), chr10:124200000-124299999(-0.206), chr10:124600000-124799999(-0.214), chr10:131600000-131699999(-0.205), chr10:134000000-134099999(-0.212), chr10:134500000-134599999(-0.205)
40	chr1:158500000-158599999(0.204), chr1:161700000-161899999(0.202), chr1:162000000-162499999(0.204), chr1:162600000-162699999(0.203), chr1:162900000-162999999(0.203), chr1:163100000-163399999(0.202), chr1:163500000-163699999(0.201), chr1:164000000-164099999(0.203), chr1:164200000-164599999(0.203), chr1:164700000-164999999(0.205), chr1:165100000-165199999(0.202), chr1:165300000-165499999(0.201), chr1:165700000-165899999(0.2), chr1:166100000-166199999(0.202), chr1:166300000-166899999(0.206), chr1:167100000-167299999(0.2), chr1:167900000-167999999(0.2)
42	chr5:159200000-159299999(0.21), chr7:77400000-77499999(0.201), chr7:78200000-78299999(0.214), chr10:5300000-5399999(-0.204), chr10:5500000-5599999(-0.2), chr10:5700000-5799999(-0.2), chr10:6000000-6299999(-0.203), chr10:6400000-6899999(-0.202), chr10:7100000-7399999(-0.201), chr10:7600000-7899999(-0.205), chr10:8000000-8499999(-0.205), chr10:8700000-8999999(-0.201), chr10:9400000-9499999(-0.201), chr10:9600000-9999999(-0.201), chr10:11000000-11099999(-0.201), chr10:11200000-11299999(-0.2), chr10:11600000-11899999(-0.203), chr10:12000000-12499999(-0.203), chr10:12600000-12699999(-0.203), chr10:13000000-13899999(-0.206), chr10:14000000-14199999(-0.207), chr10:14300000-15799999(-0.21), chr10:15900000-16799999(-0.209), chr10:16900000-18199999(-0.212), chr10:18300000-18399999(-0.207), chr10:18500000-21899999(-0.218), chr10:22000000-23199999(-0.221), chr10:23300000-24399999(-0.216), chr10:24500000-26099999(-0.221), chr10:26200000-28999999(-0.214), chr10:29100000-29299999(-0.212), chr10:29400000-29599999(-0.212), chr10:29700000-29799999(-0.208), chr10:29900000-30099999(-0.208), chr10:30300000-30399999(-0.212), chr10:30500000-32499999(-0.209), chr10:62000000-62099999(-0.202), chr11:500000-1599999(0.215), chr11:1700000-3099999(0.214), chr11:3200000-4399999(0.213), chr11:4500000-5499999(0.219), chr11:5600000-5999999(0.223), chr11:6100000-6899999(0.214), chr11:7000000-7099999(0.209), chr11:8300000-8699999(0.202), chr11:9200000-9799999(0.202), chr11:11100000-11399999(0.214), chr11:11500000-11599999(0.201), chr11:11700000-11899999(0.205), chr11:12000000-12099999(0.203), chr11:12200000-12299999(0.212), chr11:12400000-12499999(0.211), chr11:12600000-12999999(0.208), chr11:13200000-13499999(0.207), chr19:55500000-55599999(0.206), chr19:56900000-56999999(0.214), chr19:57500000-57599999(0.203), chr19:57800000-57999999(0.2)

43	chr1:62700000-62799999(0.206), chr1:150000000-150199999(0.223), chr1:153000000-153199999(0.211), chr1:153700000-153799999(0.202), chr1:154000000-154299999(0.213), chr1:154400000-154599999(0.205), chr1:155600000-155699999(0.2), chr1:155800000-156299999(0.209), chr1:157100000-157499999(0.218), chr1:157600000-158399999(0.23), chr1:158500000-159199999(0.242), chr1:159300000-159799999(0.228), chr1:159900000-159999999(0.203), chr1:160200000-160799999(0.217), chr1:160900000-161899999(0.236), chr1:162000000-162799999(0.248), chr1:162900000-164599999(0.245), chr1:164700000-165499999(0.242), chr1:165600000-166999999(0.244), chr1:167100000-169899999(0.239), chr1:170000000-178999999(0.239), chr1:179100000-179999999(0.234), chr1:180100000-180799999(0.227), chr1:180900000-184899999(0.233), chr1:185000000-186399999(0.214), chr1:186500000-187199999(0.21), chr1:187600000-187699999(0.206), chr1:192600000-192699999(0.201), chr1:193200000-193899999(0.211), chr1:194000000-195999999(0.21), chr1:198400000-198499999(0.205), chr10:76400000-77099999(-0.204), chr10:77200000-77399999(-0.203), chr10:77700000-77799999(-0.201), chr10:79000000-79099999(-0.202), chr10:79300000-79399999(-0.208), chr10:84000000-84099999(-0.209), chr10:84300000-84599999(-0.201), chr10:84900000-84999999(-0.211), chr10:85100000-85199999(-0.201), chr10:85400000-85599999(-0.202), chr10:86400000-86499999(-0.201), chr10:87300000-87999999(-0.212), chr10:88100000-88399999(-0.206), chr10:89300000-89399999(-0.212), chr10:92000000-92099999(-0.202), chr10:92200000-92399999(-0.202), chr10:92500000-92699999(-0.204), chr10:92900000-95299999(-0.206), chr10:95400000-96999999(-0.214), chr10:97100000-97199999(-0.202), chr10:97400000-98899999(-0.21), chr10:99200000-99299999(-0.201), chr10:99800000-99899999(-0.201), chr10:100400000-101099999(-0.204), chr10:101300000-101599999(-0.202), chr10:101800000-102499999(-0.205), chr10:102600000-102799999(-0.202), chr10:103000000-103299999(-0.205), chr10:103500000-106299999(-0.216), chr10:106400000-106599999(-0.209), chr10:106700000-108199999(-0.215), chr10:108500000-109099999(-0.208), chr10:109300000-109399999(-0.221), chr10:109800000-110799999(-0.215), chr10:110900000-111499999(-0.211), chr10:111600000-111999999(-0.201), chr10:112100000-112199999(-0.205), chr10:112300000-112399999(-0.21), chr10:112600000-112699999(-0.202), chr10:113400000-113499999(-0.201), chr10:113600000-113799999(-0.206), chr10:114100000-114399999(-0.203), chr10:114600000-117599999(-0.217), chr10:117700000-120899999(-0.219), chr10:121400000-121699999(-0.216), chr10:122000000-122799999(-0.22), chr10:123400000-124399999(-0.22), chr10:124500000-124899999(-0.223), chr10:126800000-126899999(-0.2), chr10:127100000-127599999(-0.207), chr10:127800000-127899999(-0.212), chr10:128000000-128499999(-0.211), chr10:128900000-128999999(-0.215), chr10:129200000-129399999(-0.211), chr10:130600000-130999999(-0.207), chr10:131100000-131199999(-0.214), chr10:131300000-131499999(-0.209), chr10:131600000-131999999(-0.213), chr10:132100000-133799999(-0.21), chr10:133900000-134099999(-0.219), chr10:134300000-135099999(-0.21), chr13:49100000-49199999(-0.207)
44	chr20:39500000-40299999(0.203), chr20:40400000-40499999(0.201), chr20:41600000-41699999(0.201), chr20:41800000-41999999(0.2), chr20:42100000-42199999(0.2), chr20:42300000-42399999(0.2), chr20:42700000-42799999(0.2), chr20:42900000-43499999(0.212), chr20:51800000-51899999(0.201), chr20:52500000-52599999(0.201), chr20:53400000-53699999(0.203), chr20:53800000-55499999(0.205), chr20:55600000-55699999(0.207)
49	chr9:21900000-22099999(0.218)
